# Comprehensive analysis of cuproptosis-related lncRNAs to predict prognosis and immune infiltration characteristics in colorectal cancer

**DOI:** 10.3389/fgene.2022.984743

**Published:** 2022-11-17

**Authors:** Zhonglin Zhu, Qiuyan Zhao, Shanbao Li, Junyong Weng, Tianan Guo, Congcong Zhu, Ye Xu

**Affiliations:** ^1^ Department of Colorectal Surgery, Fudan University Shanghai Cancer Center, Shanghai, China; ^2^ Department of Oncology, Shanghai Medical College, Fudan University, Shanghai, China; ^3^ Department of Gastroenterology, Shanghai General Hospital, Shanghai Jiao Tong University School of Medicine, Shanghai, China; ^4^ Shanghai Key Laboratory of Pancreatic Diseases, Shanghai General Hospital, Shanghai Jiao Tong University School of Medicine, Shanghai, China; ^5^ Department of General Surgery, Shanghai General Hospital, Shanghai Jiao Tong University School of Medicine, Shanghai, China

**Keywords:** cuproptosis, long non-coding RNA, prognosis, immune infiltration, colorectal cancer

## Abstract

**Background:** Cuproptosis is a novel form of cell death discovered in recent. A great quantity of researches has confirmed the close relationships and crucial roles between long non-coding RNAs (lncRNAs) with the progression of colorectal cancer (CRC). However, the relationship between cuproptosis and lncRNAs remains unclear in CRC.

**Methods:** 1,111 co-expressed lncRNAs with 16 cuproptosis regulators were retrieved from CRC samples of The Cancer Genome Atlas (TCGA) database. Through univariate Cox and least absolute shrinkage and selection operator regression analysis, a prognosis model was constructed with 15 lncRNAs. The Kaplan-Meier, receiver operating characteristic curve, C-index and principal component analysis identified the prognostic power. Furthermore, a cuproptosis-related cluster was generated based on the 15 lncRNAs by unsupervised methods. The correlations between the cuproptosis-related signatures with immune cell infiltration and anti-tumor therapy were explored by multiple algorithms.

**Results:** A risk score and nomogram with great prediction ability were constructed for CRC prognosis evaluation. The immune activate pathways, immune infiltration cells, immune functions, immune score and immune activation genes were remarkably enriched in the high risk group. The cuproptosis-related cluster was generated, of which the cluster 2 showed longer overall survival. The immune cell infiltration analysis indicated the similar results of cluster 2 with the high risk group, implying a significant marker for “hot tumor.” The cluster 2 also presented high expression of immune checkpoint molecules, MSI-H status and higher susceptibility to multiple immunotherapy drugs.

**Conclusion:** We appraised a novel cuproptosis-related prognosis model and molecular signature associated with prognosis, immune infiltration and immunotherapy. The identification of cuproptosis-related lncRNAs improved our understanding of immune infiltration and provided a significant marker for prognosis and immunotherapy in CRC.

## Introduction

Copper is a necessary microelement for organic activities, and yet it changes to be cytotoxic when the concentration exceeds a certain threshold (Ruiz et al., 2021; Ge et al., 2022). It was lately reported that copper-induced cell death, named cuproptosis, is a novel type of cell death due to intracellular copper accumulation, distinct from any known format of cell death including apoptosis, autophagy, necrosis, ferroptosis and pyrotosis (Xi et al., 2016; Marshall et al., 2019; Koppula et al., 2022; Pan et al., 2022; Wang et al., 2022). Excessive copper participates in mitochondrial tricarboxylic acid (TCA) cycle through directly binding to lipoylated proteins, thus subsequent lipoylated protein aggregation and Fe-S deficiency result in proteotoxic stress and cell death (Tsvetkov et al., 2022). It was widely revealed that dysregulation of cell death mechanism was closely related with development and progression of various cancer types, and cell death related genes were promising targets for suppressing tumor growth and progression (Mao et al., 2021; Lei et al., 2022; Zhang et al., 2022). However, the correlations between cuproptosis and tumor progression remain unclear.

Long non-coding RNA (lncRNA) is a type of non-coding RNA with the length of more than 200 nt and without protein coding potential (Park et al., 2021; Winkle et al., 2021). LncRNAs can regulate genes’ expression and functions through multiple patterns, including signal, decoy, guide and scaffold (Schmitt and Chang, 2016; Liu et al., 2021). Increasing studies revealed the key roles of lncRNAs on tumor growth, progression, metastasis, chemoradiotherapy resistance and immunosuppression (Huang et al., 2018; Kim et al., 2018; Wong et al., 2018; Chen et al., 2019; Goodall and Wickramasinghe, 2021). LncRNA RMRP promoted MDM2-induced p53 ubiquitination and degradation *via* SNRPA1, thus promoting cell proliferation and preventing cell apoptosis (Chen et al., 2021). LncRNA LINC00336 inhibited ferroptosis through functioning as a ceRNA to promote the expression of cystathionine-β-synthase (CBS) in lung cancer (Wang et al., 2019). Nonetheless, the studies of cuproptosis-related lncRNA have not been retrieved up to now. Therefore, exploring cuproptosis-related lncRNAs facilitates our cognition of the crucial roles of cuproptosis and lncRNAs on the progression of colorectal cancer (CRC).

With the progression of biological immune system and tumor immune contexture, immunotherapy has been widely adapted in clinical patients (Best et al., 2022; Di Luccia and Colonna, 2022). However, the efficiency of immunotherapy differs greatly in individuals with divergent tumor immune contexture (Guillerey et al., 2016; Ciardiello et al., 2022; Liu et al., 2022). The interaction of tumor-immune system provided a large mount of foundation to construct a rational stratification of patients. Tumors have been classified into “hot tumor” and “cold tumor” according to the immune cell infiltration around tumors, and further regrouped into three types: immune-inflamed, immune-excluded and immune-desert (Galon and Bruni, 2019; Noman et al., 2020; Bruchard et al., 2022; Eggermont et al., 2022). Immune checkpoint blockades targeting PD-1 and PD-L1 have been approved for solid tumor with mismatch repair deficient (dMMR)/high microsatellite instability (MSI-H), including CRC (Homet Moreno and Ribas, 2015). With regard to the outcome, the overall prognosis of CRC patients was markedly affected by the enrichment of tumor immune cell infiltration (Anitei et al., 2014; Biller and Schrag, 2021). A prognostic and accuracy study showed that immunoscore was an indicator for immune reactions and prognosis evaluation of CRC patients (Pagès et al., 2018). Therefore, insight knowledge of the characteristics of the host immune system and fundamental mechanisms of tumor development and progression contributes to making a sweeping generalisation to patient stratification and provides potential targets for immunotherapy. Increasing evidence indicates that lncRNAs are involved in the regulation of tumor immune response (Li et al., 2021; Chen et al., 2022). LncRNA NKILA sensitizes T cells to activation-induced cell death through suppressing NF-κB activity, therefore promoting tumor immune evasion (Huang et al., 2018). Cancer cell-derived exosomes lncRNA SNHG16 upregulated CD73 expression *via* miR-16-5p/SMAD5 axis in Vδ1 T cells and subsequently converted Vδ1 T cells into CD73+ immunosuppressive phenotype in breast cancer (Ni et al., 2020). Consequently, taking a closer look of the regulations of lncRNAs on tumor immune microenvironment is of great significance.

In the study, we systemically integrated the RNA-seq data of 605 CRC samples from The Cancer Genome Atlas (TCGA) and 886 CRC samples from Gene Expression Omnibus (GEO) database. 1,111 cuproptosis-related lncRNAs were retrieved. Then, a prognosis model and molecular cluster with cuproptosis-related lncRNAs were constructed. Further analysis uncovered the close relationships between the cuproptosis molecular signatures with prognosis and immune cell infiltration, implying the great potential for the cuproptosis molecular signature as a biomarker of prognosis evaluation and a target for turning “cold tumor” into “hot tumor” in CRC.

## Materials and methods

### Preparation of RNA-seq data

RNA-sequencing data and clinical annotation of colon cancer and rectal cancer were downloaded from TCGA database and GEO database. Transcriptome profiles of 605 samples (43 normal samples and 562 cancer samples) in TCGA-colon adenocarcinoma/rectum adenocarcinoma (COAD/READ) were obtained and merged in the format of fragments per kilobase million (FPKM). The GEO data of GSE39582 (19 normal samples and 566 cancer samples) (Conesa et al., 2016), GSE17536 (177 colorectal cancer tissues) (Smith et al., 2010) and GSE72970 (124 colorectal cancer tissues) (Del Rio et al., 2017) were obtained for external validation of the prognosis model.

## Identification of cuproptosis-related lncRNAs

A list of 16 cuproptosis regulators were retrieved from lipoylated TCA cycle pathway of copper induced cell death (FDX1, LIPT1, LIAS, DLD, MTF1, GLS, CDKN2A, DLAT, PDHA1, PDHB, DBT, GCSH, and DLST) (Tsvetkov et al., 2022) and copper transport protein (SLC31A1, ATP7A, and ATP7B) (Graden and Winge, 1997; Lukanović et al., 2020). According to their roles in lipoylated TCA cycle pathway, these regulators were classified into 4 groups: 7 upregulators, 3 downregulators, 3 enzymes and 3 carriers. To find lncRNAs related with 16 cuproptosis regulators, pearson correlation analysis was conducted to analyze the expressions of lncRNAs and 16 cuproptosis regulators in the colorectal cancer tissues. Co-expressed lncRNAs with 16 cuproptosis regulators were identified by the standard of coefficients |Pearson R| >0.4 and *p* < 0.001. Next, differentially expressed lncRNAs between normal and cancer samples were screened by |Log2 fold change| >1 and FDR<0.05 with R package “limma” (3.50.3).

### Establishment of the prognosis model with cuproptosis-related lncRNAs

The samples were randomly divided into the train group and test group. According to the clinicopathological data and the expression of cuproptosis-related lncRNAs in the train group, univariate Cox regression analysis was performed to screen prognosis-related lncRNAs (*p* < 0.05). Next, LASSO analysis was employed to optimize the selected lncRNAs. Then, a best model was generated by multivariate Cox regression model analysis. The risk score was computed as follows (Song et al., 2022; Sotiriou et al., 2006):

$$risk\;score=\overset{n}{\underset{i=1}{\sum }}coef\left(lncRNA_{i}\right)*\exp\left(lncRNA_{i}\right)$$

Coef (lncRNA_i_) and exp (lncRNA_i_) represent the coefficient and expression of each lncRNAs, respectively.

### Assessment of the prognosis model and construction of nomogram

The samples were regrouped into high risk and low risk group based on the median of risk score. The Kaplan-Meier, principal component analysis (PCA), t-distributed stochastic neighbor embedding (t-SNE), uMAP, treceiver operating characteristic (ROC) curve and C-index were adapted to appraise the accuracy of the prognosis model. Then, the univariate and multivariate Cox regression analysis were employed to assess if the prognosis model could function as an independent prognostic indicator for CRC. Last, a nomogram was established to predict 1-, 3-, and 5-years overall survival rates combined risk score with age, gander, T, N, M, and stage. The calibration curves and decision curve analysis (DCA) were plotted to test the consistency and net benefits.

### Gene set enrichment analyses

GSEA was used to identify the significantly enriched biological behaviors and pathways in the two risk groups with the hallmark gene sets (v7.5.1) and KEGG gene sets (v7.5.1) (Liberzon et al., 2015; Wu et al., 2021).

### Investigation of the immune infiltration

The immune infiltration in TCGA samples was appraised by several algorithms including XCELL, TIMER, QUANTISEQ, MCPcounter, EPIC, and CIBERSORT. The enrichments of immune cells and immune functions were examined with single-sample gene set enrichment analysis (ssGSEA) (Charoentong et al., 2017). In addition, the stromal score and immune score of each sample were quantified by ESTIMATE algorithm.

### Unsupervised consensus cluster for cuproptosis-related lncRNAs

According to the expression of identified cuproptosis-related and significant prognosis-related lncRNAs, the unsupervised consensus clustering analysis was employed to classify samples into distinct molecular patterns with R package “ConsensusClusterPlus.”

### Somatic mutation and microsatellite instability analysis

The somatic mutation data of samples was obtained from the TCGA database by varscan file format. The significant mutated genes and tumor mutation burden (TMB) were calculated with R package “maftool.” The percentages of microsatellite stability (MSS), high MSI (MSI-H) and low MSI (MSI-L) were computed in different cuporptosis-related clusters.

### Significance of the cuproptosis-related signatures in chemotherapy and immunotherapy

To assess the efficiencies of different anti-tumor drugs on the patients with distinct cuproptosis-related signature, the “pRRophetic” package was adapted to calculate the half-maximal inhibitory concentration (IC50) of 251 common chemotherapy drugs, such as AKT inhibitor, Cisplatin, and Paclitaxel (Geeleher et al., 2014). The immune cell proportion score (IPS) data of TCGA samples was downloaded from The Cancer Immunome Altas (https://tcia.at/home). The IPS scores of anti-CTLA4, anti-PD-1 and anti-PD-L1 drugs were compared.

### Statistical analyses

All the data analysis was exerted by R software (version 4.1.2) and Strawberry Perl (version 5.3.0). *p*-value < 0.05 was set as statistical significance.

## Results

### Identification of cuproptosis-regulated lncRNAs

**FIGURE 1:**
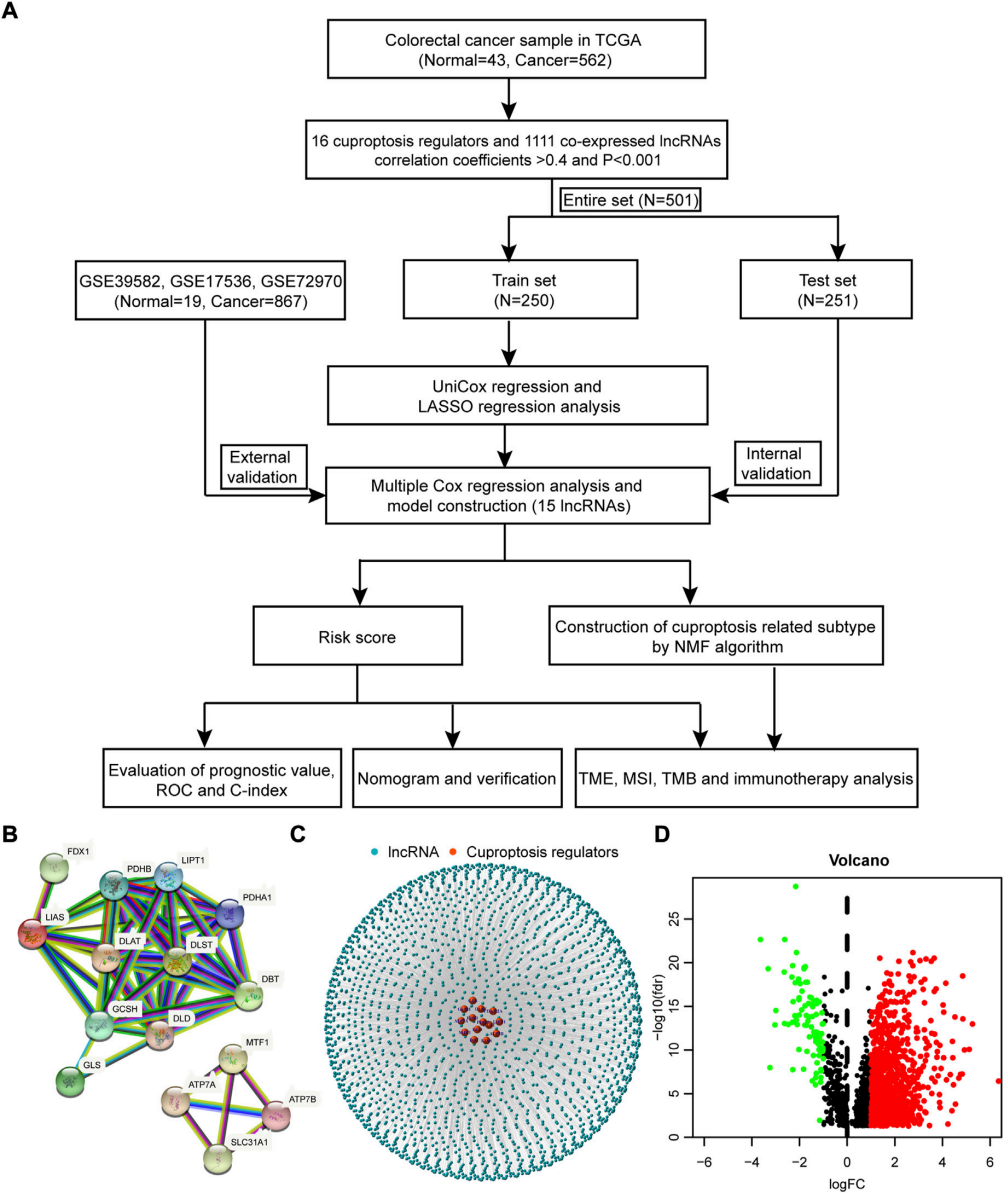
The process of this work and network of lnRNA-mRNA co-expression. **(A)** The process of this work. **(B)** The PPI network of 16 cuproptosis regulators analyzed by the STRING database. **(C)** The lncRNA-mRNA co-expression network. **(D)** The volcano plot of differentially expressed lncRNAs between normal and cancer samples.

The process of this work is exhibited in Figure 1A. RNA-sequencing data and clinical annotation of colon cancer and rectal cancer were downloaded from TCGA database, which consisted of 43 normal samples and 562 cancer samples. A total of 16 cuproptosis regulators were retrieved from lipoylated TCA cycle pathway of copper induced cell death in recent publication. The interaction of these genes was depicted with a PPI network, analyzed by the STRING database (Figure 1B, Supplementary Table S1). By pearson correlation analysis, 2,246 co-expressed lncRNAs with 16 cuproptosis regulators was identified (coefficients>0.4 and *p* < 0.001, Supplementary Table S2). Then, a lncRNA-mRNA co-expression network was generated to describe the interrelations (Figure 1C). Last, 1,111 differentially expressed lncRNAs (Log2 fold change >1 and FDR<0.05, Figure 1D, Supplementary Table S3) between normal and cancer samples were selected for further analysis.

### Construction of cuproptosis-related prognosis model

**FIGURE 2:**
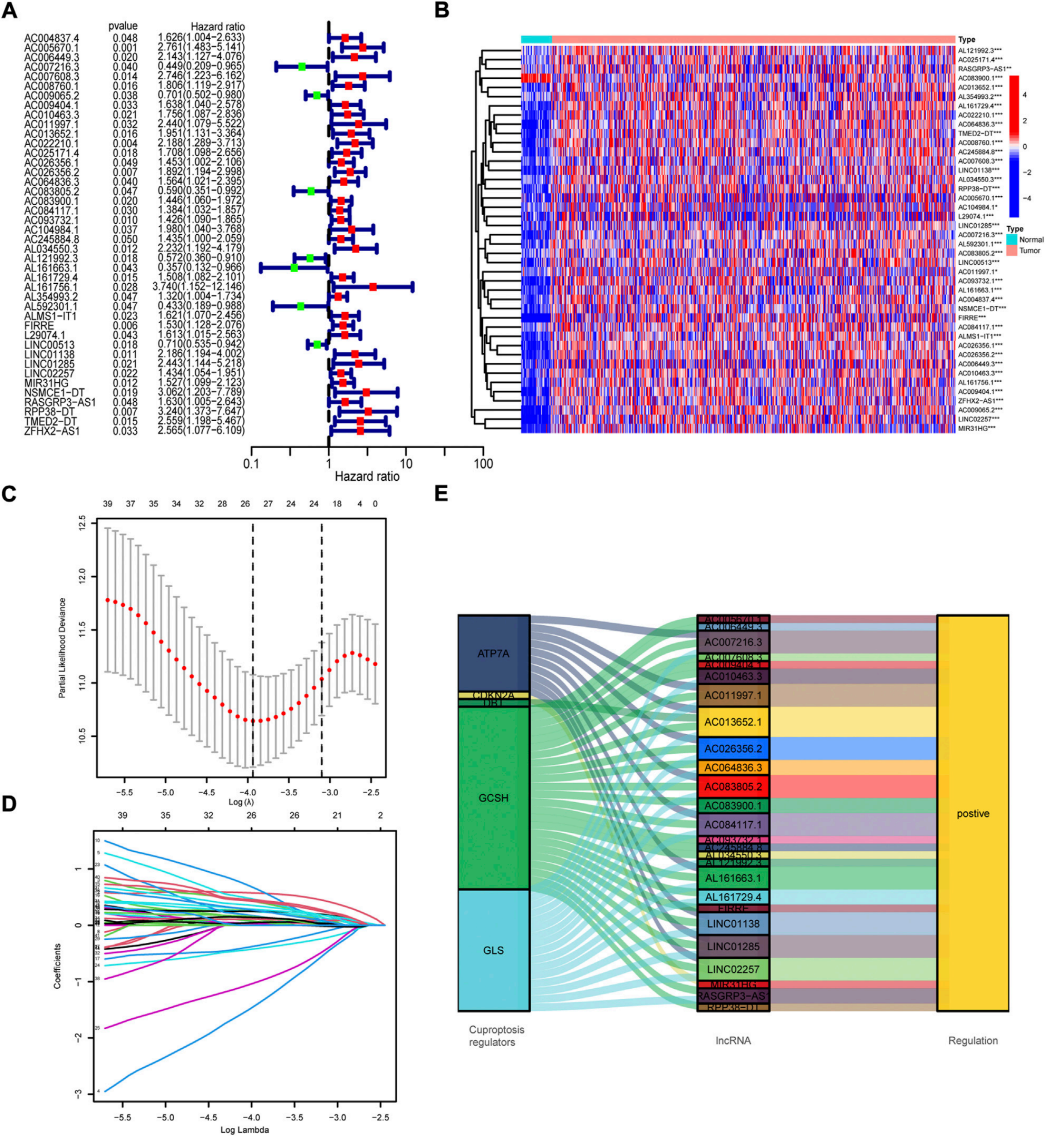
Screen of cuproptosis-related lncRNAs. **(A)** The results of univariate Cox regression analysis of prognosis-related lncRNAs. **(B)** The expression profiles of 42 prognosis-related lncRNAs. **(C)** The cross-validation in the LASSO model. **(D)** LASSO coefficient profile of prognosis-related lncRNAs. **(E)** Sankey diagram of cuproptosis regulators and prognosis-related lncRNAs.

To construct prognosis model, the samples were randomly divided into the train group and test group, of which the train group was used to generate model and the test group to validate model. Univariate Cox regression analysis of the above 1,111 cuproptosis-related lncRNAs was performed in the train group. We identified 42 prognosis-related lncRNAs (*p* < 0.05, Figure 2A) and made a heatmap to portray the expression of these lncRNAs in normal and cancer samples of TCGA COAD/READ (Figure 2B). To decrease the fitting of prognostic signatures, LASSO regression analysis was employed to optimize the prognosis-related lncRNAs (Figures 2C,D). 26 lncRNAs (Supplementary Table S4) were extracted for multivariate Cox regression model. Accidentally, we discovered that 26 prognosis-related lncRNAs all positively correlated with cuproptosis regulators (Figure 2E). Then, the multivariate Cox regression model analysis was performed and the optimal prognostic model was constructed with 15 lncRNAs (*p* < 0.05). 15 lncRNAs and their weighted coefficients were shown in Supplementary Table S5.

**FIGURE 3:**
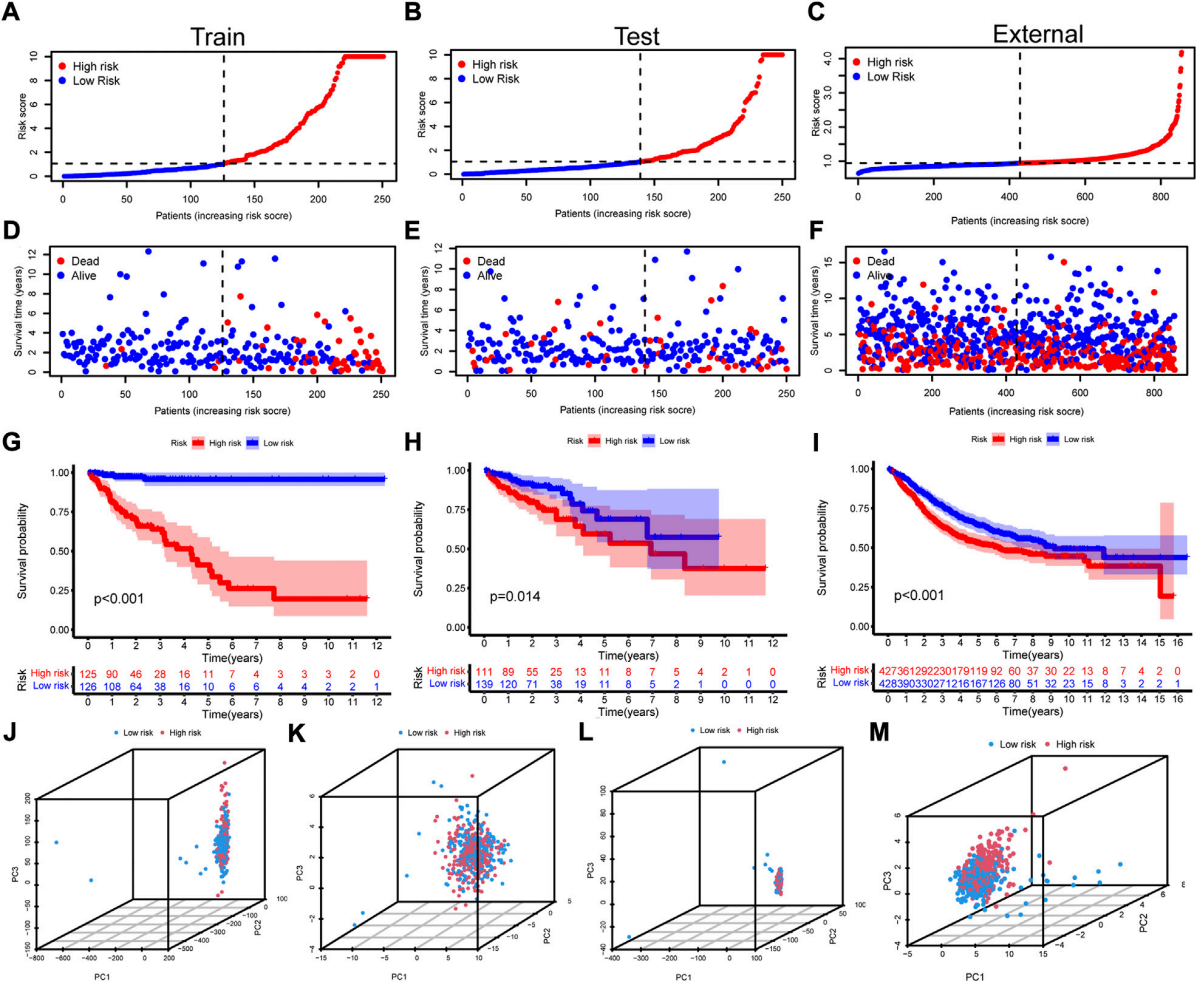
Construction of the cuproptosis-related prognosis model. **(A-C)** The scatterplot of risk score in the train set, test set, and the external validation set, respectively. **(D-F)** The distribution of survival time and survival status of high and low risk groups in the train set, test set, and the external validation set. **(G-I)** The Kaplan–Meier survival curves of overall survival between high and low risk groups in the train set, test set, and the external validation set. **(J-M)** The PCA with all genes, cuproptosis regulators, 1,111 cuproptosis-related lncRNAs and 15 model lncRNAs for all the samples.

With the median of risk score being the cut off, the samples were regrouped into high risk group and low risk group in the train group, the test group and the all samples (Supplementary Table S6). The scatterplot of risk score, survival time and survival status were drawn in the high and low risk groups of the train group, the test group and the external validation set (Figures 3A–I). The high risk group showed poorer overall survival. Besides, the high risk group also exhibited same survival disadvantage in patients with stratified gender, T, N, M, and stage (Supplementary Figure S1).

### Assessment of cuproptosis-related prognosis model

**FIGURE 4:**
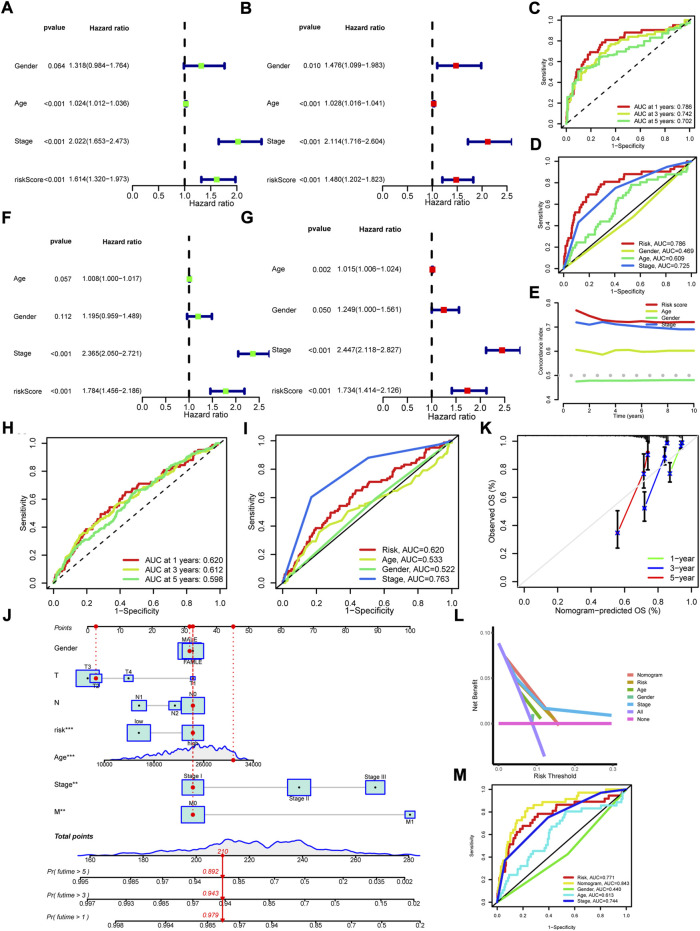
Assessment of cuproptosis-related prognosis model and construction of Nomogram. **(A,B)** The univariate and multivariate Cox regression analysis of overall survival in all samples. **(C)** The 1-, 3-, and 5-years ROC of risk score in all samples. **(D)** The 1-year ROC of risk score, age, gender and stage. **(E)** The C-index of risk score, age, gender and stage. **(F,G)** The univariate and multivariate Cox regression analysis of overall survival in the external validation set. **(H)** The 1-, 3-, and 5-years ROC of risk score in the external validation set. **(I)** The 1-year ROC of risk score, age, gender and stage in the external validation set. **(J)** The nomogram was constructed with risk score, age, gender, T, N, M, and stage to predict the overall survival rate of 1-, 3- and 5-years. **(K)** The calibration curves for predicting the probability of 1-, 3- and 5-years. **(L)** DCA curves for assessing the clinical utility of the nomogram. **(M)** The 1-year ROC of nomogram, risk score, age, gender and stage.

To detect the differences within the high and low risk groups, PCA was conducted. The results indicated that all genes (Figure 3J), cuproptosis regulators (Figure 3K) and 1,111 cuproptosis-related lncRNAs (Figure 3L) could not distinguish the samples, while lncRNAs in the risk model exhibited the best discrimination ability (Figure 3m). Then, the univariate (Figure 4A) and multivariate (Figure 4B) Cox regression analysis were employed to assess whether the risk score could act as an independent prognostic factor for CRC. The results identified the great prediction efficiency with HR value being 1.614 and 1.480 respectively. The area under curve of ROC were utilized to appraise the sensitivity and specificity of the risk score. The 1-, 3- and 5-years AUC of all the samples were 0.786, 0.742 and 0.702 (Figure 4C). Also, the 1-year AUC of risk score was higher than that of stage, age and gender (Figure 4D), implying the greater prediction efficiency. The C-index showed the same results (Figure 4E).

GEO data (GSE39582, GSE17536, and GSE72970) and clinical phenotypes were obtained for external validation of the prognosis model. The results indicated that patients in high risk group showed a shorter overall survival (Figures 3C,F,I, Supplementary Table S7). The univariate (Figure 4F) and multivariate (Figure 4G) Cox regression analysis identified the risk score as an independent prognostic factor for CRC. The 1-, 3-, and 5-years AUC were 0.620, 0.612, and 0.598 (Figure 4H). Although lower than the 1-year AUC of stage, the 1-year AUC of risk score presented fine sensitivity and specificity (Figure 4I). Taken together, the external validation confirmed the great prediction efficiency for prognosis in CRC.

### Construction and validation of nomogram

Combined risk score with clinicopathological features including age, gender, T, N, M, and stage, we constructed a nomogram to calculate the overall survival rate of 1-, 3-, and 5-years (Figure 4J). The calibration plots demonstrated a good concordance for the prediction efficiency of 1-, 3-, and 5-years overall survival (Figure 4K). Furthermore, the DCA curve also confirmed the prediction efficiency of nomogram and risk score (Figure 4L). The 1-year AUC of the nomogram was up to 0.843 (*p* < 0.05, Figure 4M), showing the predominant predicative ability.

### The correlations between clinicopathological features and immune cell infiltration with risk score

**FIGURE 5:**
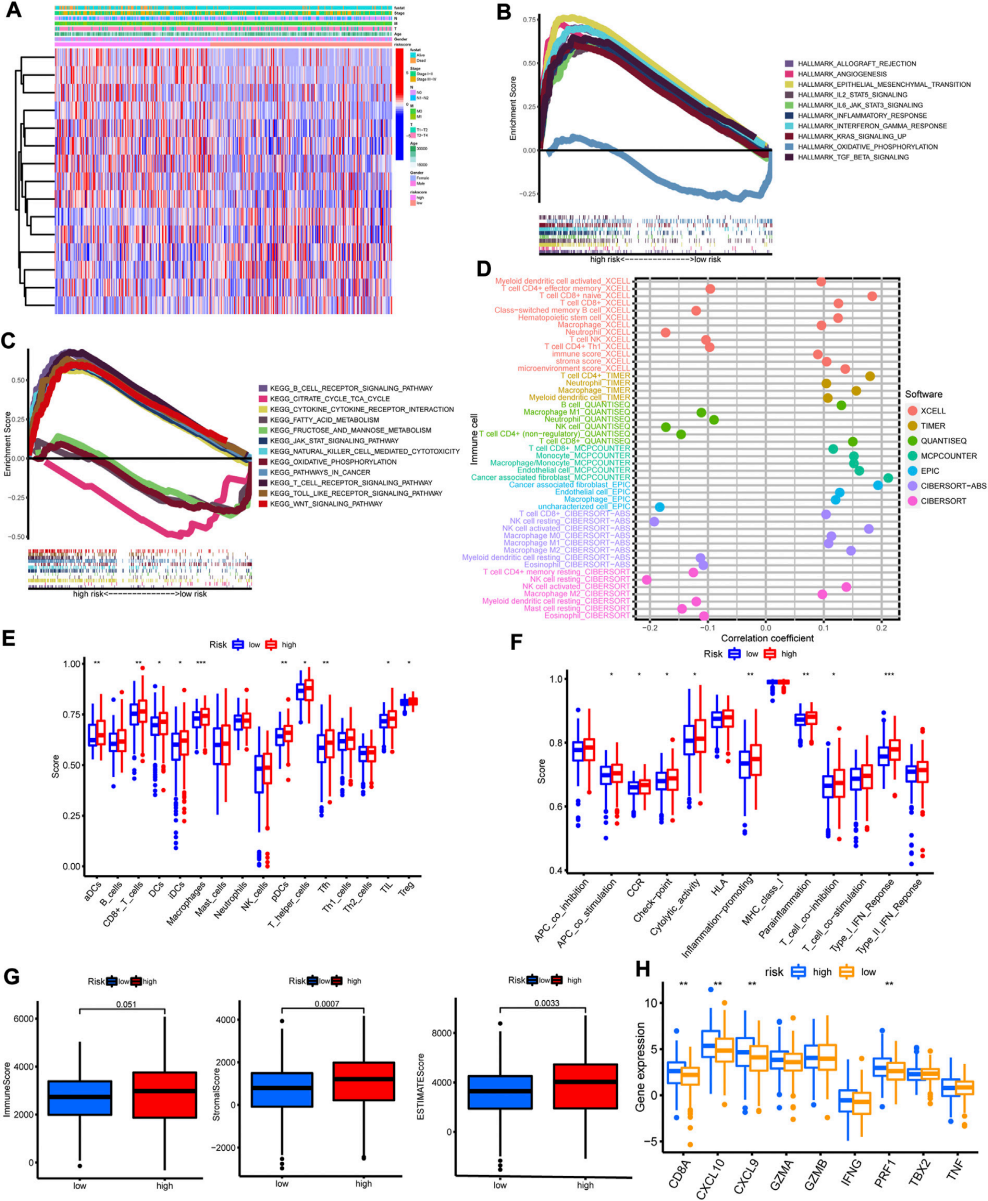
The correlations between clinicopathological features and immune cell infiltration with risk score. **(A)** The heatmap of 15 model lncRNAs expression and clinicopathological features in high and low risk group. **(B)** The heatmap of GSVA with hallmark sets between high and low risk group. **(C)** The heatmap of GSVA with KEGG sets between high and low risk group. **(D)** The immune cell bubble plot. **(E,F)** The enrichment of immune cells and immune functions in high and low risk group by ssGSVA algorithm. **(G)** The immune score, stromal score and ESTIMATE score in high and low risk group by ESTIMATE algorithm. **(H)** The expression of immune activated genes between high and low risk group. *p < 0.05; **p < 0.01; ***p < 0.001.

We analyzed the correlations between clinicopathological features with risk score. As shown in Figure 5A, high risk score was preferentially related to more M1, higher stage and dead status. GSEA results of hallmark gene sets indicated that high risk group was enriched with immune activation biological functions, including inflammatory response, IL2/STAT5 signaling, IL6/JAK/STAT3 signaling and interferon gamma (Figure 5B). GSEA analysis of KEGG also showed significant enrichment in high risk group with immune activated pathways (Figure 5C), such as B cell receptor signaling pathway, JAK/STAT signaling pathway, toll like receptor signaling pathway, natural killer cell mediated cytotoxicity and T cell receptor signaling pathway. The immune cells enrichment analysis by multiple algorithms demonstrated that high risk group was positively correlated with the enrichments of CD8+ T cell, CD4+ T cell, B cell, NK cell, macrophage and cancer associated fibroblast (Figure 5D). Furthermore, high risk score was positively related with the enrichment levels of NK cell, B cell, CD8+ T cell, CD4+ T cell, monocyte, macrophage and cancer associated fibroblast (Supplementary Figure S2). Besides, ssGSEA results indicated that high risk group was rich in immune cells (Figure 5E), including dendritic cell, B cell, CD8+ T cell, macrophage, NK cell and tumor infiltration lymphocyte, and immune functions (Figure 5F), including APC co-stimulation, CCR, check-point, cytolytic activity, inflammation promoting and type I IFN response. The stromal score and immune score of each sample were quantified with ESTIMATE algorithm (Supplementary Table S8). The high risk group presented higher immune score, stromal score and ESTIMATE score (Figure 5G). The immune activity related genes such as CD8A, CXLC10, CXCL9, GZMA, GZMB, IFNG, PRF1, TBX2A, and TNF, were upregulated in high risk group, especially CD8A, CXLC10, CXCL9, and PRF1 (Figure 5H). The above results demonstrated that high risk was closely correlated with progressed clinicopathological features and high immune cell infiltration status, implying “hot tumor” type.

### The investigation of risk score with TMB and the clinical treatment

Considering the close relationship between risk score with immune cell infiltration, we analyzed the levels of TMB and clinical treatment response between different risk groups in this part. Regretfully, no significant difference of somatic mutation and TMB between high and low risk group was observed (Supplementary Figures S3A,B). The Kaplan-Meier curves showed no significant difference of overall survival between high and low TMB group (Supplementary Figure S3C), but a trend of a shorter 5-years survival in high TMB group. Nonetheless, the differences of overall survival were obvious between groups with different TMB level and risk score (Supplementary Figure S3D). Then, we compared the expression of immune checkpoint genes in the high and low risk group, and found that all these genes showed high levels in high risk group (Supplementary Figure S3E). To examine the efficiency of immune checkpoint blockades, the IPS of TCGA samples was downloaded online (Supplementary Table S9, https://tcia.at/home). The high risk group presented high IPS with anti-CLTA4 drug, meaning better immunotherapy response (Supplementary Figure S3F). Last, we adapted the “pRRophetic” package to calculate the IC50 of 251 common chemotherapy drugs. The results showed that various anti-tumor drugs presented lower IC50 in high risk group, such as rapamycin, gemcitabine, paclitaxel (Supplementary Figures S3G–L, Supplementary Figure S4).

### Generation and immune cell infiltration characteristics of cuproptosis-related subtypes

**FIGURE 6:**
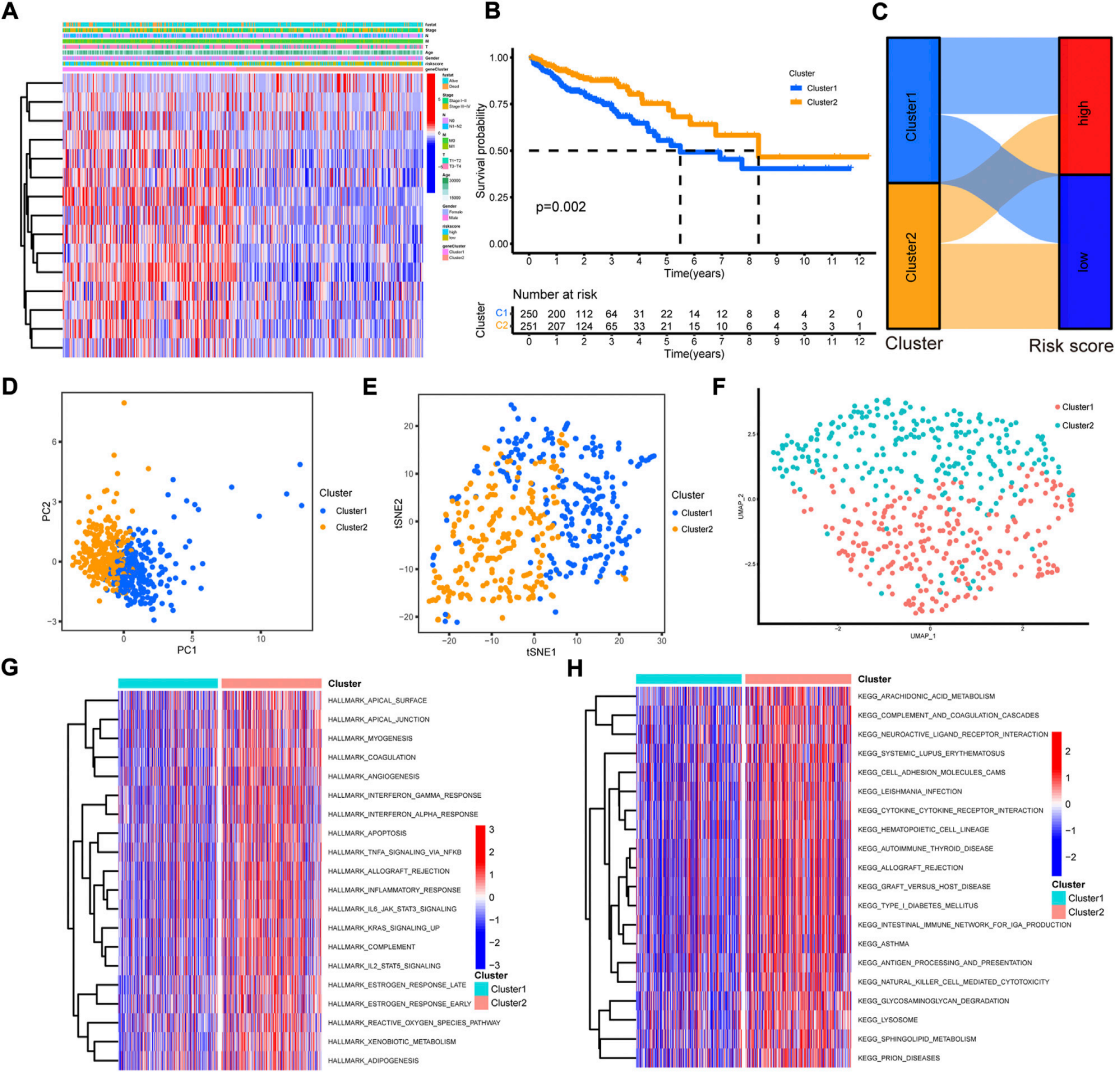
The construction and function annotation of cuproptosis-related cluster. **(A)** The heatmap of 15 model lncRNAs expression and clinicopathological features in cluster 1 and cluster 2. **(B)** The Kaplan–Meier survival curves of overall survival between cluster 1 and cluster 2. **(C)** The alluvial diagram showed the attribute changes from risk score to cluster subtype. **(D)** The PCA of the two clusters. **(E)** The t-SNE of the two clusters. **(F)** The uMAP analysis of the two clusters. **(G)** The heatmap of GSVA with hallmark sets between the two clusters. **(H)** The heatmap of GSVA with KEGG sets between the two clusters.

To draw a comprehensive picture of cuproptosis-related pattern, consensus clustering was performed with the 15 cuproptosis-related prognostic lncRNAs to group samples into different signature subtypes (Supplementary Figures S5A,B). Eventually, two cuproptosis-related phenotypes were generated, termed as cluster 1–2 (Supplementary Figures S5C,D, Supplementary Table S10). The correlations between clinicopathological features with cluster group were analyzed. The results showed that cluster 2 was preferentially related to higher T stage and more alive status (Figure 6A). The Kaplan-Meier curves indicated a better overall survival in cluster 2 than that in cluster 1 (Figure 6B). The alluvial diagram showed the majority of high risk samples was grouped into cluster 1, while most of low risk samples were classified into cluster 2 (Figure 6C). Then, we employed PCA to verify whether cluster group could distinguish the samples. The results showed a markedly difference between the two clusters (Figure 6D). In addition, t-SNE and uMAP analysis also indicated same result (Figures 6E,F).

**FIGURE 7:**
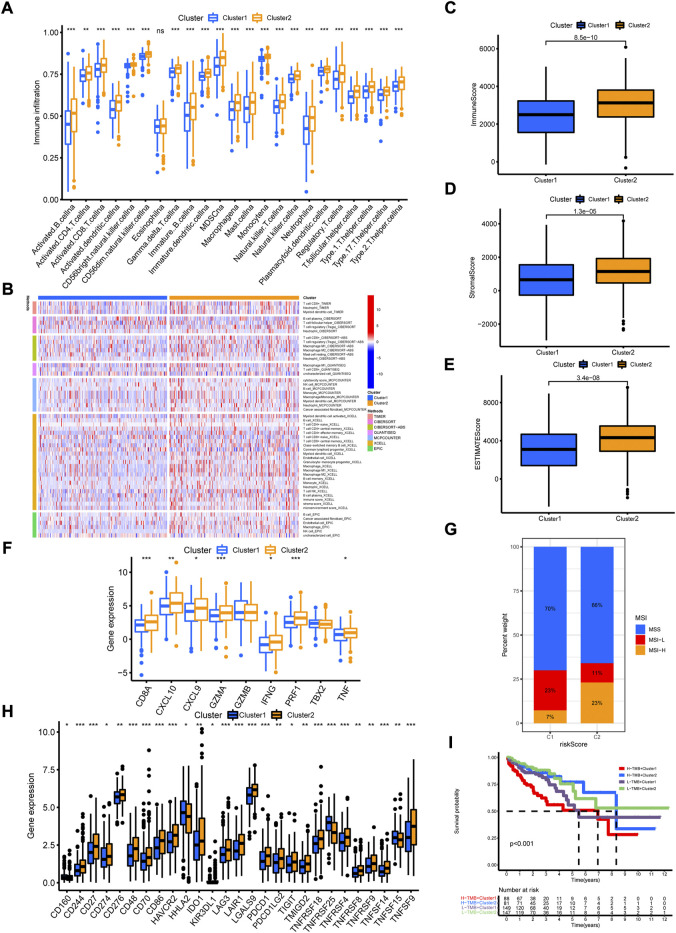
The immune cell infiltration characteristics of cuproptosis-related clusters. **(A)** The enrichment of immune cells in the two clusters by ssGSVA algorithm. **(B)** The immune cell bubble plot in the two clusters. **(C–E)** The immune score, stromal score and ESTIMATE score in the two clusters by ESTIMATE algorithm. **(F)** The expression of immune activated genes between the two clusters. **(G)** The expression of immune checkpoint genes between the two clusters. **(H)** The distribution of MSI status in the two clusters. **(I)** The Kaplan-Meier curves of overall survival in different groups of TMB combination with clusters. *p < 0.05; **p < 0.01; ***p < 0.001.

To evaluate the biological functions of the two cuproptosis-related clusters, GSVA was performed with hallmark gene sets and KEGG sets. Cluster 2 was markedly enriched in immune activation functions and pathways, such as allograft rejection, inflammatory response, interferon gamma response, interferon alpha response, IL2/STAT5 signaling, IL6/JAK/STAT3 signaling, antigen processing and presentation and natural killer cell mediated cytotoxicity (Figures 6G,H). The ssGSEA results indicated high enrichment of almost all the immune cells in cluster 2 group, including dendritic cell, B cell, CD4+ T cell, CD8+ T cell, macrophage and NK cells (Figure 7A). Then, the immune cells enrichment analysis was further analyzed by multiple algorithms. The heatmap showed the similarly high enrichment of almost all the immune cells in cluster 2 group (Figure 7B). In addition, the cluster 2 group showed higher immune score, stromal score and ESTIMATE score (Figures 7C–E). The immune activity related genes were upregulated in cluster 2, espically CD8A, CXLC10, CXCL9, GZMA, IFNG, TNF, and PRF1 (Figure 7F). The above results demonstrated that cluster 2 was significantly enriched in immune infiltration cells, conforming to “hot tumor” type, while cluster 1 exhibited with low immune cells infiltration, according with “cold tumor.”

### Relationship of cuproptosis-related subtypes with tumor somatic mutation and clinical treatment

**FIGURE 8:**
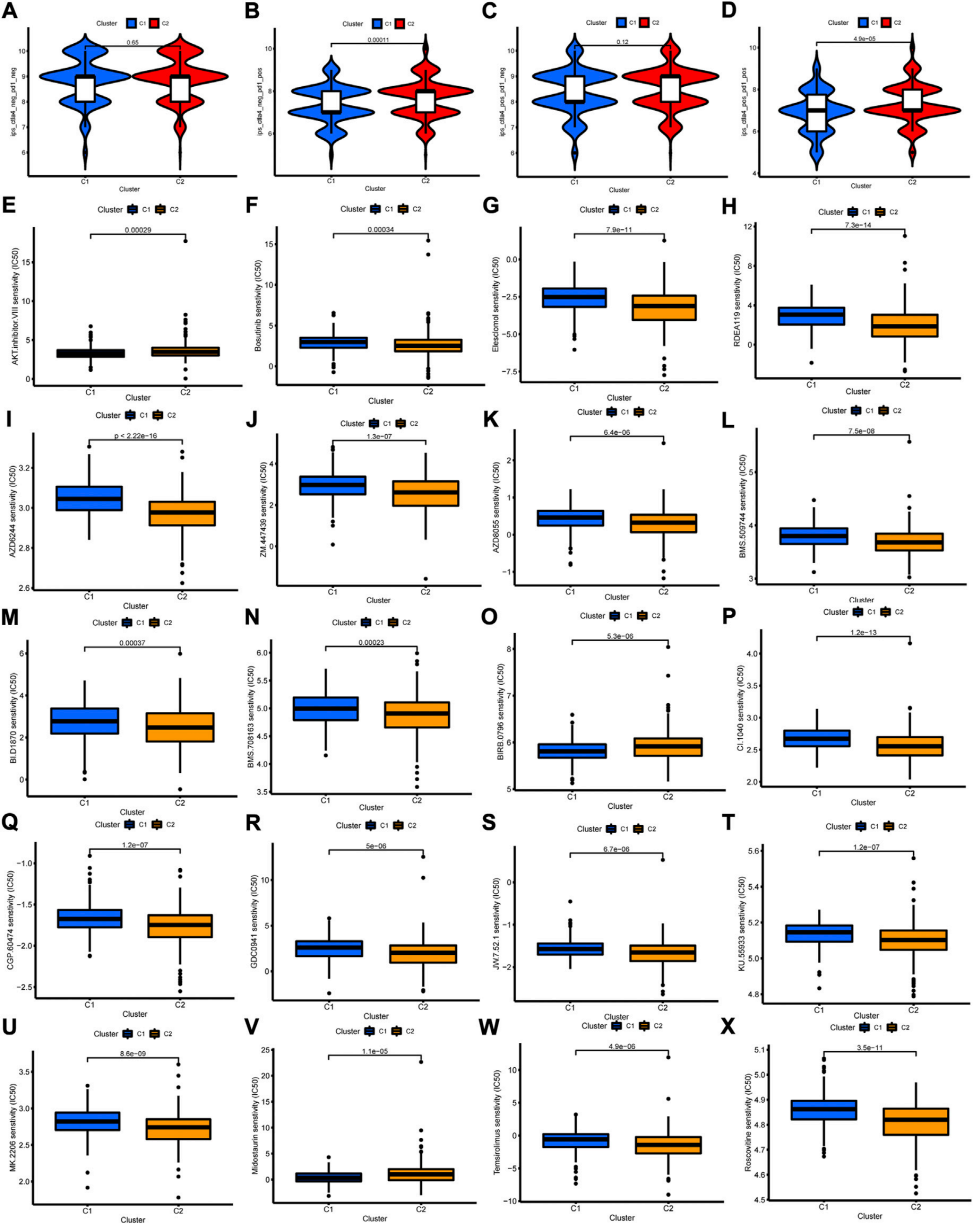
The Relationship of cuproptosis-related clusters with clinical treatment. **(A–D)** The IPS in the two clusters. **(E–X)** The IC50 of 20 anti-tumor drugs in the two clusters.

Given the close correlation between cuproptosis-related cluster with immune cell infiltration, we further explore whether cuproptosis-related cluster could affect immunotherapy response. First, we examined the expression of immune checkpoint genes, and found high levels of all these genes in cluster 2 (Figure 7G). Second, the status of MSI was compared between the two clusters. The cluster 2 showed high frequency of MSI-H and low frequency of MSS (Figure 7H). Third, it is a pity that no significant difference of somatic mutation and TMB between the two clusters was observed (Supplementary Figures S6A–C). However, the Kaplan-Meier curves showed obvious differences between groups with different TMB level and cluster (Figure 7I). Then, we examined the efficiency of immune checkpoint blockades, and found high IPS with anti-CLTA4 drug or combination of anti-CTLA4 and anti-PD-1 drug in cluster 2, meaning better immunotherapy response (Figures 8A–D). Last, we adapted the “pRRophetic” package to predict the IC50 of different chemotherapy drugs in the two clusters. The results indicated that multiple drugs presented lower IC50 in cluster 2 (Figures 8E–X).

## Discussion

In the past decades, intracellular copper toxicity has not been clearly elaborated (Oliveri, 2022). Although the drugs of copper ionophores have entered clinical trials, beneficial outcomes have not been acquired, which may result from failing to screen appropriate patient populations and understand the action of drugs’ mechanism (O'Day et al., 2013; Davis et al., 2020; Tsang et al., 2020). With the discovery of copper-induced cell death mechanism—cuproptosis in recent, it will improve our cognition of drugs targeting copper and help to construct suitable patient subgroup. In this study, we constructed a prognosis model with cuproptosis-related lncRNAs to predict prognosis and clinical efficiency of anti-tumor drugs for CRC patients. Furthermore, a cuproptosis-related cluster was established and closely correlated with clinicopathological features and immune cell infiltration. The cuproptosis-related patterns contribute to our understanding of mechanisms targeting copper drugs and tumor microenvironment, suggesting an available biomarker for distinguishing “hot tumor” and “cold tumor” and predicting efficiency of immunotherapy in CRC.

LncRNAs has been widely reported to participate in the development and progression of various cancers (Yang and Al-Hendy, 2022). However, there was no cuproptosis-related lncRNA reported up to now. In consideration of multi-dimensional regulation of lncRNAs in the levels of epigenetics, transcription, post-transcription, translation and post-translation, there are reasons to believe that lncRNAs are involved in the regulation of cuproptosis and the roles of cuproptosis on the progression of cancers. In the study, we identified 15 cuproptosis-related and prognosis-related lncRNAs, which may be conducive to further research of lncRNAs and cuproptosis. Based on the expression of the 15 lncRNAs, a cuproptosis-related prognosis model was constructed. The univariate and multivariate Cox regression analysis, ROC and C-index demonstrated the predominant efficiency of predicting prognosis of this model. A nomogram was constructed to compute the survival rate of 1-, 3-, and 5-years, whose accuracy was confirmed by ROC, calibration and DCA curves. Therefore, we established and verified a novel cuproptosis-related prognosis model and nomogram for CRC.

With the advance of tumor immunobiology and targeted drugs, immunotherapy has been widely adapted in clinic and archived beneficial outcomes (Zhang et al., 2021; Luo et al., 2022). Nonetheless, the outcome is highly heterogeneous in patient subgroup with different tumor microenvironment (Zeng et al., 2021). The tumor microenvironment has been demonstrated to affect the result of immunotherapy in various studies (Song et al., 2021). According to the immune cell infiltration in tumor microenvironment, the tumor is classified into two types: “hot tumor” and “cold tumor” (Zhao et al., 2021). “Hot tumor” is rich in immune cells and immune activated, therefore positive response to immunotherapy, while “cold tumor” is short of immune cells and immune deserted, thus no-response to immunotherapy. In this work, high risk group was enriched with immune activated functions and pathways and high immune score. The immune cell infiltration analysis showed high enrichment in high risk group and positive correlation of immune cell infiltration with risk score. Therefore, the high risk group was classified as “hot tumor,” while the low risk group was “cold tumor.” The drug sensitivity analysis indicated the low IC50 of various anti-tumor drugs in high risk group, which provided foundation for the selection of clinical treatment schedule. When we examined the efficiency of immune checkpoint blockades, only drugs anti-CTLA4 exhibited a positive response in high risk group. Various factors may intervene immunotherapy. Consequently, in-depth studies need to be taken to dissect the correlation of cuproptosis with tumor microenvironment.

To draw a synthetic picture of cuproptosis-related patterns, a cuproptosis-related cluster was constructed based on the 15 lncRNAs. The cuproptosis-related cluster was closely correlated with clinicopathological features and prognosis of CRC. GSVA and immune cell infiltration analysis showed high enrichment of immune activated pathways, such as B cell receptor signaling pathway, cytokine-cytokine receptor interaction, natural killer cell mediated cytotoxicity, T cell receptor signaling pathway and toll like receptor signaling pathway, and multiple immune cells including dendritic cell, B cell, CD4+ T cell, CD8+ T cell, macrophage and NK cells. Accordingly, cluster 2 was grouped into “hot tumor,” while cluster 1 was “cold tumor.” Similarly, the drug sensitivity analysis indicated the low IC50 of various anti-tumor drugs in cluster 2. Furthermore, we examined the expression of immune checkpoint genes in two clusters and found high expression in cluster 2. In addition, immune checkpoint blockades of anti-CTLA4 and anti-PD-1 exhibited a positive response in cluster 2. Taken together, the cuproptosis-related cluster was closely correlated with clinicopathological features and immune cell infiltration, and contributed to differentiating “hot tumor” and “cold tumor.” The generation of cuproptosis-related cluster is expected to be a significant biomarker for prognosis evaluation and a target for altering “cold tumor” into “hot tumor” in CRC.

## Conclusion

We comprehensively explored the cuproptosis-related pattern in CRC samples from different databases, and constructed a cuproptosis-related prognosis model and a cuproptosis-related cluster with 15 cuproptosis-related lncRNAs. The cuproptosis-related prognosis model and cluster were both closely correlated with clinicopathological features and immune cell infiltration, and conducive to distinguishing “hot tumor” and “cold tumor.” In a word, the systematic analysis emphasized the crucial roles of cuproptosis-related patterns in prognosis and immune cell infiltration of CRC, which contributed to our understanding of the interaction of cuproptosis and tumor microenvironment.

## Data Availability

The datasets presented in this study can be found in online repositories. The names of the repository/repositories and accession number(s) can be found in the article/Supplementary Material.
