# Supplementary material for: Comprehensive analysis of cuproptosis-related lncRNAs to predict prognosis and immune infiltration characteristics in colorectal cancer
Source: Front Genet. 2022 Nov 17;13:984743. doi: 10.3389/fgene.2022.984743 (PMC9712968; doi:10.3389/fgene.2022.984743)
Supplement: Supplementary file 1 [file DataSheet1.docx]

**Supplementary files:**

**Supplementary Figure S1**

**
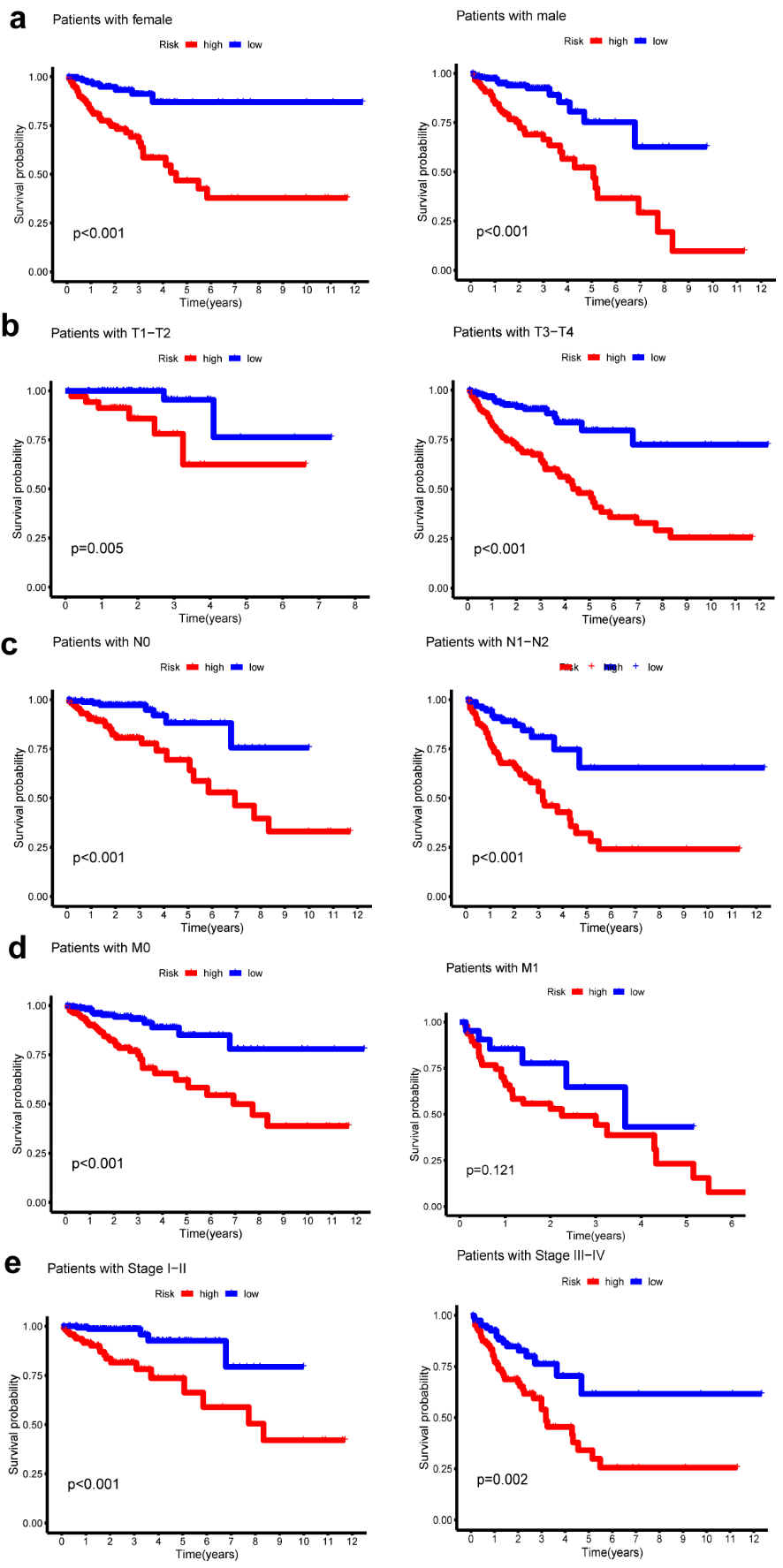
**

**Supplementary FIGURE S1** | The Kaplan-Meier curves of overall survival in the high and low risk group with different clinicopathological features. The Kaplan-Meier curves of high and low risk groups in TCGA samples with female and male (**a**),T1-T2 stages and T3-T4 stages (**b**), N0 stages and N1-N2 stages (**c**), M0 stages and M1 stages (**d**), TNM I-II stages and TNM III-IV stages (**e**), respectively.

**Supplementary FIGURE S2**

**
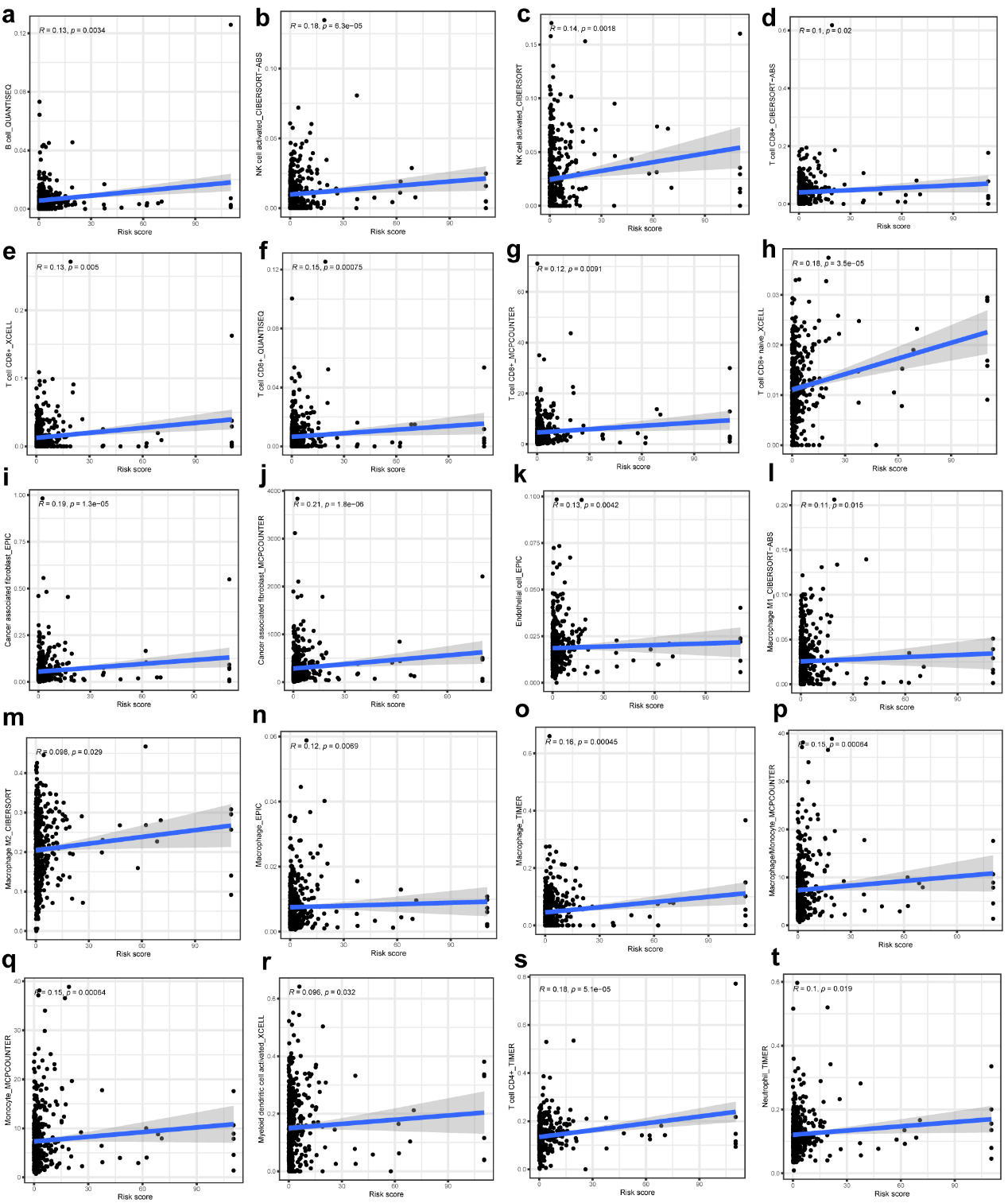
**

**Supplementary FIGURE S2** | The correlations between risk score and the enrichment level of immune cells. Pearson correlation analysis between risk score with the enrichment levels of immune cells by multiple algorithms (**a-t**).

**Supplementary FIGURE S3
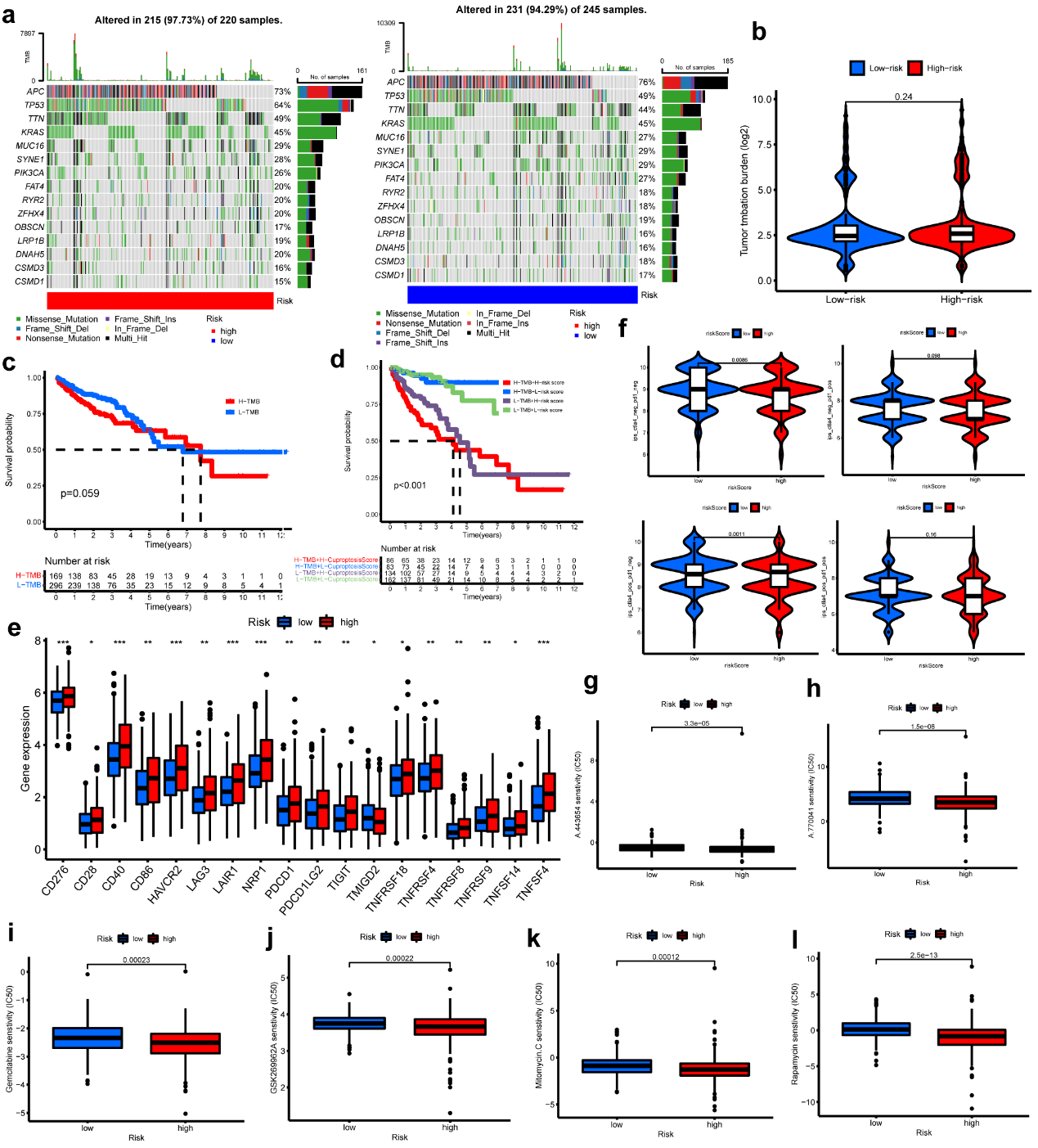
**

**Supplementary FIGURE S3** | The relationship of prognosis model with tumor somatic mutation and clinical treatment. **(a)** The waterfall plot of tumor somatic mutation in high risk group and low risk group of TCGA data. **(b)** The TMB of high and low risk group. **(c)** The Kaplan-Meier curves of overall survival in high and low TMB groups. **(d)** The Kaplan-Meier curves of overall survival in different groups of TMB combination with risk score. **(e)** The expression of immune checkpoint genes between the high and low risk group. **(f)** The IPS in the high and low risk group. **(g-l)** The IC50 of anti-tumor drugs in the high and low risk group. *P < 0.05; **P < 0.01; ***P < 0.001.

**Supplementary FIGURE S4**

**
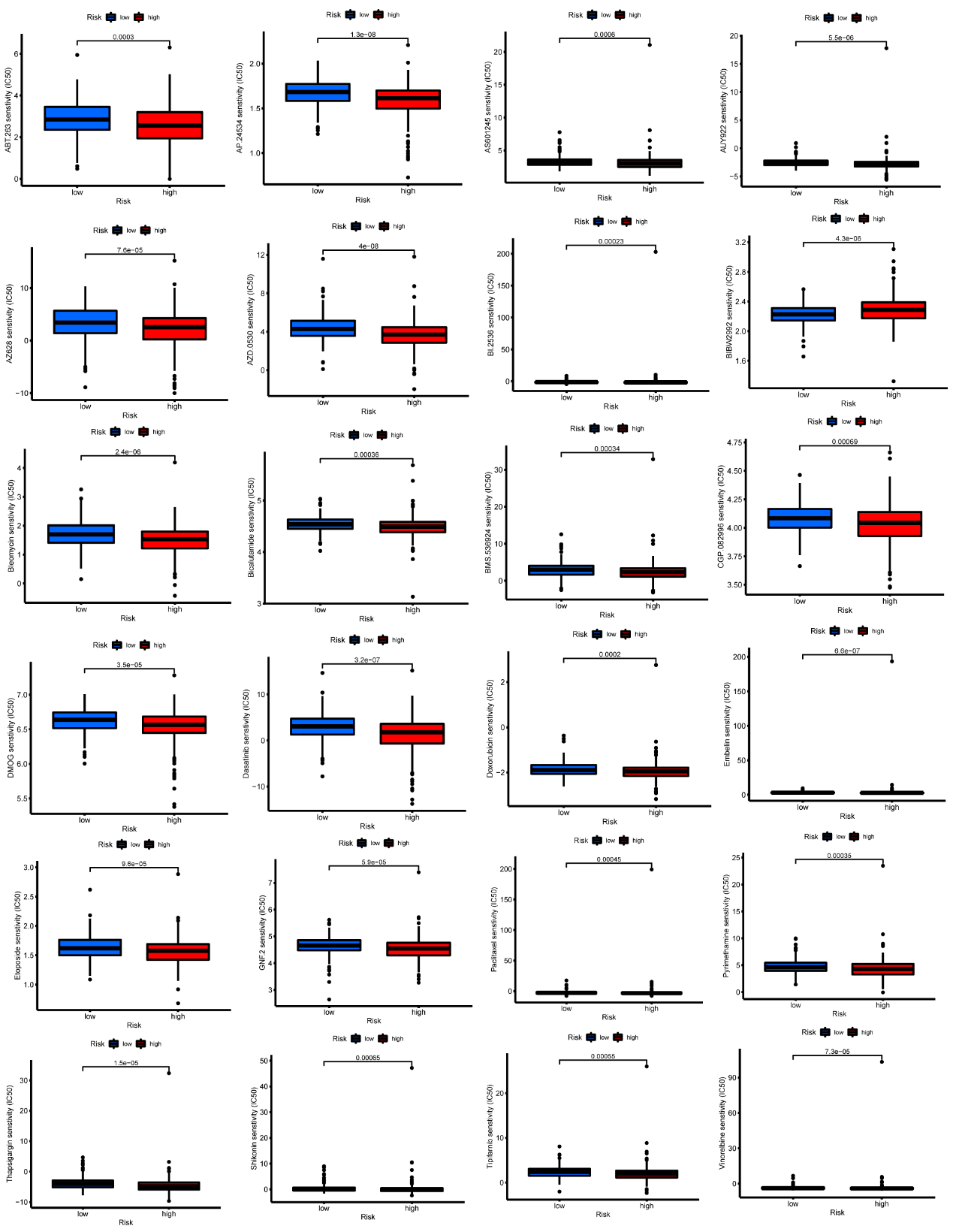
**

**Supplementary FIGURE S4** | The efficiency of clinical treatment in the high and low risk group. The IC50 of anti-tumor drugs in the high and low risk group.

**Supplementary FIGURE S5**

**
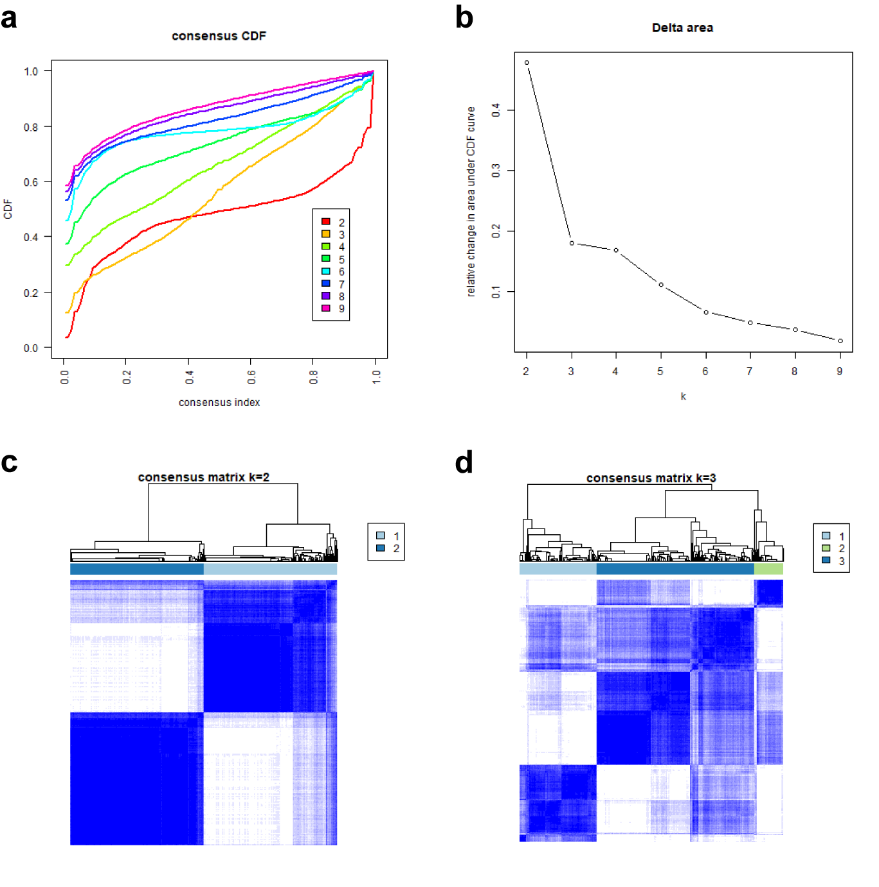
**

**Supplementary FIGURE S5** | Unsupervised consensus clustering of cuproptosis-related lncRNAs. **(a)** The relative change in area under the cumulative distribution function (CDF) curves. **(b)** The curves of consensus CDF. **(c-d)** The heat maps for consensus matrices (k=2-3).

**Supplementary FIGURE S6**

**
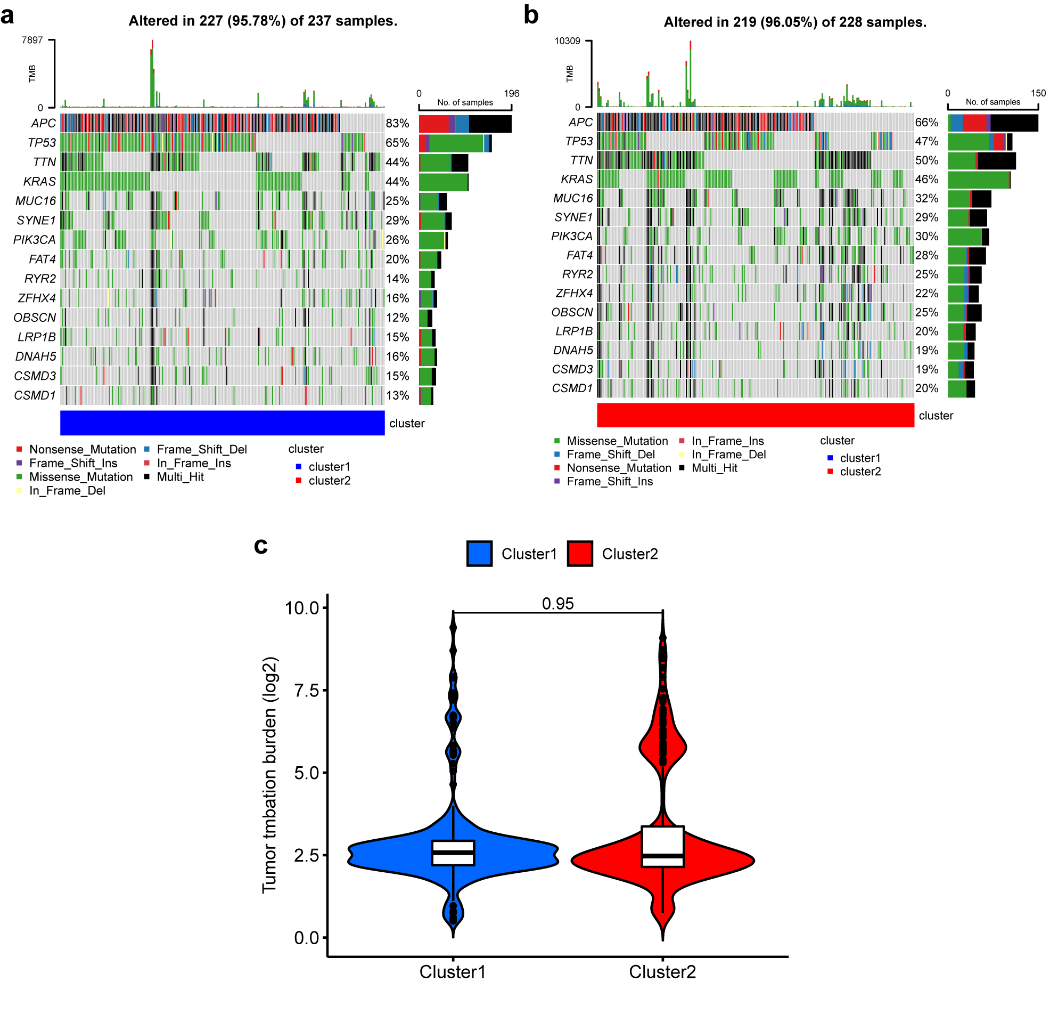
**

**Supplementary FIGURE S6** | The relationship of prognosis model with tumor somatic. **(a-b)** The waterfall plot of tumor somatic mutation in cluster 1 and cluster 2 of TCGA data. **(c)** The TMB of cluster 1 and cluster 2.

**Supplementary Table S1.** The data of PPI network analyzed by the STRING database.

| #node1 | node2 | coexpression | combined_score |
| --- | --- | --- | --- |
| ATP7A | ATP7B | 0 | 0.811 |
| ATP7A | MTF1 | 0.062 | 0.624 |
| ATP7A | SLC31A1 | 0.082 | 0.887 |
| ATP7B | MTF1 | 0.062 | 0.714 |
| ATP7B | SLC31A1 | 0.082 | 0.887 |
| DBT | DLD | 0.196 | 0.997 |
| DBT | DLAT | 0.143 | 0.697 |
| DBT | PDHB | 0.312 | 0.87 |
| DBT | GCSH | 0.062 | 0.947 |
| DBT | DLST | 0.138 | 0.854 |
| DBT | PDHA1 | 0.132 | 0.747 |
| DBT | LIPT1 | 0.066 | 0.963 |
| DLAT | DLD | 0.61 | 0.999 |
| DLAT | LIAS | 0.082 | 0.662 |
| DLAT | LIPT1 | 0.062 | 0.983 |
| DLAT | GCSH | 0.103 | 0.985 |
| DLAT | DLST | 0.449 | 0.988 |
| DLAT | PDHA1 | 0.775 | 0.999 |
| DLAT | PDHB | 0.889 | 0.999 |
| DLD | LIAS | 0.11 | 0.802 |
| DLD | GLS | 0.062 | 0.813 |
| DLD | LIPT1 | 0.083 | 0.975 |
| DLD | GCSH | 0.145 | 0.99 |
| DLD | PDHA1 | 0.439 | 0.998 |
| DLD | PDHB | 0.863 | 0.999 |
| DLD | DLST | 0.833 | 0.999 |
| DLST | LIAS | 0.066 | 0.743 |
| DLST | PDHB | 0.309 | 0.942 |
| DLST | GCSH | 0.088 | 0.983 |
| DLST | PDHA1 | 0.17 | 0.88 |
| DLST | LIPT1 | 0.065 | 0.959 |
| FDX1 | LIAS | 0.066 | 0.756 |
| GCSH | LIAS | 0.09 | 0.977 |
| GCSH | PDHB | 0.064 | 0.917 |
| GCSH | GLS | 0 | 0.809 |
| GCSH | PDHA1 | 0.085 | 0.92 |
| GCSH | LIPT1 | 0.063 | 0.993 |
| LIAS | PDHA1 | 0.067 | 0.412 |
| LIAS | PDHB | 0.09 | 0.542 |
| LIAS | LIPT1 | 0.066 | 0.991 |
| LIPT1 | PDHB | 0.066 | 0.964 |
| LIPT1 | PDHA1 | 0.064 | 0.942 |
| MTF1 | SLC31A1 | 0.062 | 0.703 |
| PDHA1 | PDHB | 0.98 | 0.999 |

**Supplementary Table S2.** The 2246 co-expressed lncRNAs with 16 cuproptosis regulators.

| ID |
| --- |
| A2M-AS1 |
| ABCA9-AS1 |
| AC000061.1 |
| AC000065.1 |
| AC000072.1 |
| AC000082.1 |
| AC000123.1 |
| AC001226.1 |
| AC002044.1 |
| AC002057.2 |
| AC002074.1 |
| AC002128.1 |
| AC002128.2 |
| AC002306.1 |
| AC002451.1 |
| AC002463.1 |
| AC002480.1 |
| AC002480.2 |
| AC002542.6 |
| AC002546.1 |
| AC002550.2 |
| AC002553.1 |
| AC002563.1 |
| AC003682.1 |
| AC003991.1 |
| AC003992.1 |
| AC004000.1 |
| AC004039.1 |
| AC004067.1 |
| AC004069.1 |
| AC004076.2 |
| AC004148.1 |
| AC004160.2 |
| AC004223.2 |
| AC004241.3 |
| AC004241.4 |
| AC004253.1 |
| AC004466.1 |
| AC004466.2 |
| AC004466.3 |
| AC004492.1 |
| AC004540.1 |
| AC004554.1 |
| AC004584.1 |
| AC004593.1 |
| AC004594.1 |
| AC004637.1 |
| AC004691.1 |
| AC004704.1 |
| AC004765.1 |
| AC004812.2 |
| AC004825.3 |
| AC004832.4 |
| AC004832.5 |
| AC004837.2 |
| AC004837.4 |
| AC004839.2 |
| AC004846.1 |
| AC004893.2 |
| AC004900.1 |
| AC004908.1 |
| AC004908.2 |
| AC004918.3 |
| AC004943.1 |
| AC004943.2 |
| AC004943.3 |
| AC004951.4 |
| AC004967.2 |
| AC004982.1 |
| AC005005.3 |
| AC005034.5 |
| AC005046.1 |
| AC005050.1 |
| AC005062.1 |
| AC005070.3 |
| AC005072.1 |
| AC005086.2 |
| AC005096.1 |
| AC005104.1 |
| AC005165.1 |
| AC005261.1 |
| AC005264.1 |
| AC005277.2 |
| AC005304.1 |
| AC005332.2 |
| AC005332.7 |
| AC005381.1 |
| AC005410.2 |
| AC005479.1 |
| AC005479.2 |
| AC005480.1 |
| AC005498.3 |
| AC005519.1 |
| AC005520.5 |
| AC005540.1 |
| AC005546.1 |
| AC005594.1 |
| AC005618.4 |
| AC005632.3 |
| AC005632.6 |
| AC005670.1 |
| AC005674.1 |
| AC005699.1 |
| AC005726.2 |
| AC005757.1 |
| AC005776.2 |
| AC005785.1 |
| AC005838.2 |
| AC005856.1 |
| AC005899.6 |
| AC005920.3 |
| AC006008.1 |
| AC006017.1 |
| AC006033.2 |
| AC006042.1 |
| AC006042.3 |
| AC006059.1 |
| AC006062.1 |
| AC006111.2 |
| AC006111.3 |
| AC006116.4 |
| AC006159.1 |
| AC006160.1 |
| AC006213.7 |
| AC006230.1 |
| AC006299.1 |
| AC006355.2 |
| AC006449.3 |
| AC006460.1 |
| AC006460.2 |
| AC006504.1 |
| AC006504.5 |
| AC006504.7 |
| AC006511.5 |
| AC006525.1 |
| AC006547.1 |
| AC006947.1 |
| AC006960.3 |
| AC007014.2 |
| AC007038.1 |
| AC007098.1 |
| AC007114.2 |
| AC007128.1 |
| AC007128.2 |
| AC007216.2 |
| AC007216.3 |
| AC007216.4 |
| AC007278.2 |
| AC007285.1 |
| AC007292.2 |
| AC007319.1 |
| AC007336.1 |
| AC007362.1 |
| AC007383.1 |
| AC007384.1 |
| AC007390.1 |
| AC007431.2 |
| AC007483.1 |
| AC007497.1 |
| AC007535.1 |
| AC007601.1 |
| AC007608.1 |
| AC007608.2 |
| AC007608.3 |
| AC007620.3 |
| AC007622.2 |
| AC007637.1 |
| AC007639.1 |
| AC007663.4 |
| AC007684.2 |
| AC007695.1 |
| AC007728.2 |
| AC007728.3 |
| AC007750.1 |
| AC007785.3 |
| AC007823.1 |
| AC007879.3 |
| AC007920.2 |
| AC007938.1 |
| AC007938.2 |
| AC007938.3 |
| AC008026.3 |
| AC008033.3 |
| AC008080.4 |
| AC008114.1 |
| AC008115.2 |
| AC008115.3 |
| AC008119.1 |
| AC008121.2 |
| AC008124.1 |
| AC008264.2 |
| AC008269.1 |
| AC008429.1 |
| AC008434.1 |
| AC008453.2 |
| AC008456.1 |
| AC008467.1 |
| AC008537.2 |
| AC008543.1 |
| AC008649.2 |
| AC008667.3 |
| AC008735.4 |
| AC008752.2 |
| AC008758.2 |
| AC008760.1 |
| AC008764.8 |
| AC008771.1 |
| AC008870.2 |
| AC008870.3 |
| AC008870.4 |
| AC008883.3 |
| AC008894.2 |
| AC008906.1 |
| AC008937.3 |
| AC008957.1 |
| AC008966.1 |
| AC008966.3 |
| AC009032.1 |
| AC009041.3 |
| AC009053.3 |
| AC009054.1 |
| AC009054.2 |
| AC009065.2 |
| AC009090.3 |
| AC009090.6 |
| AC009093.1 |
| AC009093.2 |
| AC009097.1 |
| AC009107.2 |
| AC009120.2 |
| AC009120.3 |
| AC009120.5 |
| AC009121.2 |
| AC009135.1 |
| AC009142.1 |
| AC009148.1 |
| AC009152.6 |
| AC009159.2 |
| AC009229.2 |
| AC009244.1 |
| AC009262.1 |
| AC009269.5 |
| AC009299.2 |
| AC009315.1 |
| AC009318.2 |
| AC009318.4 |
| AC009387.1 |
| AC009404.1 |
| AC009495.2 |
| AC009554.2 |
| AC009560.4 |
| AC009563.1 |
| AC009690.2 |
| AC009704.2 |
| AC009754.1 |
| AC009803.1 |
| AC009812.3 |
| AC009812.4 |
| AC009908.1 |
| AC009948.1 |
| AC009948.2 |
| AC009950.1 |
| AC009975.1 |
| AC009975.2 |
| AC009996.1 |
| AC010148.1 |
| AC010149.1 |
| AC010168.1 |
| AC010168.2 |
| AC010184.1 |
| AC010207.1 |
| AC010226.1 |
| AC010245.2 |
| AC010260.1 |
| AC010285.3 |
| AC010300.1 |
| AC010307.2 |
| AC010320.3 |
| AC010326.3 |
| AC010327.5 |
| AC010343.3 |
| AC010359.3 |
| AC010422.4 |
| AC010463.3 |
| AC010491.2 |
| AC010524.1 |
| AC010525.1 |
| AC010530.1 |
| AC010536.2 |
| AC010538.1 |
| AC010542.5 |
| AC010551.2 |
| AC010618.2 |
| AC010618.3 |
| AC010624.3 |
| AC010632.1 |
| AC010680.2 |
| AC010727.1 |
| AC010761.1 |
| AC010789.2 |
| AC010834.3 |
| AC010894.3 |
| AC010976.1 |
| AC010999.2 |
| AC011092.3 |
| AC011247.1 |
| AC011287.2 |
| AC011330.2 |
| AC011337.1 |
| AC011352.1 |
| AC011352.3 |
| AC011363.1 |
| AC011365.1 |
| AC011442.1 |
| AC011462.5 |
| AC011466.1 |
| AC011466.2 |
| AC011466.3 |
| AC011468.1 |
| AC011472.4 |
| AC011477.2 |
| AC011481.1 |
| AC011503.2 |
| AC011510.1 |
| AC011595.1 |
| AC011603.2 |
| AC011676.1 |
| AC011676.2 |
| AC011676.3 |
| AC011700.1 |
| AC011773.1 |
| AC011773.4 |
| AC011815.1 |
| AC011815.2 |
| AC011921.1 |
| AC011933.2 |
| AC011997.1 |
| AC012020.1 |
| AC012065.2 |
| AC012076.1 |
| AC012085.2 |
| AC012178.1 |
| AC012181.1 |
| AC012184.3 |
| AC012184.4 |
| AC012213.3 |
| AC012254.3 |
| AC012313.5 |
| AC012313.9 |
| AC012358.2 |
| AC012360.1 |
| AC012409.1 |
| AC012409.3 |
| AC012409.4 |
| AC012442.1 |
| AC012443.2 |
| AC012459.1 |
| AC012464.1 |
| AC012464.2 |
| AC012467.2 |
| AC012494.1 |
| AC012494.2 |
| AC012501.2 |
| AC012615.4 |
| AC012640.1 |
| AC012676.3 |
| AC012676.4 |
| AC013391.2 |
| AC013400.1 |
| AC013452.2 |
| AC013468.1 |
| AC013486.1 |
| AC013643.2 |
| AC013652.1 |
| AC013726.1 |
| AC013731.1 |
| AC015727.1 |
| AC015813.1 |
| AC015849.3 |
| AC015853.1 |
| AC015871.6 |
| AC015908.4 |
| AC015909.2 |
| AC015911.3 |
| AC015914.1 |
| AC015921.1 |
| AC015922.3 |
| AC015967.1 |
| AC015983.2 |
| AC015987.1 |
| AC016245.2 |
| AC016394.1 |
| AC016394.2 |
| AC016405.1 |
| AC016597.1 |
| AC016597.2 |
| AC016644.1 |
| AC016683.1 |
| AC016722.2 |
| AC016727.1 |
| AC016727.3 |
| AC016737.1 |
| AC016737.2 |
| AC016745.1 |
| AC016831.1 |
| AC016831.4 |
| AC016831.6 |
| AC016910.1 |
| AC016949.1 |
| AC017048.3 |
| AC017067.1 |
| AC017071.1 |
| AC017076.1 |
| AC017083.1 |
| AC017104.5 |
| AC017116.2 |
| AC018410.1 |
| AC018552.3 |
| AC018557.2 |
| AC018628.2 |
| AC018645.3 |
| AC018648.1 |
| AC018690.1 |
| AC018695.3 |
| AC018695.4 |
| AC018714.2 |
| AC018766.1 |
| AC018809.1 |
| AC018845.3 |
| AC018926.1 |
| AC018926.2 |
| AC018926.3 |
| AC018978.1 |
| AC019080.1 |
| AC019080.5 |
| AC019118.1 |
| AC019131.2 |
| AC019186.1 |
| AC019205.1 |
| AC019254.1 |
| AC019330.1 |
| AC020612.3 |
| AC020634.2 |
| AC020658.4 |
| AC020661.3 |
| AC020663.3 |
| AC020763.1 |
| AC020891.3 |
| AC020915.1 |
| AC020915.2 |
| AC020917.2 |
| AC020978.1 |
| AC020978.2 |
| AC020978.3 |
| AC020978.7 |
| AC021078.1 |
| AC021087.3 |
| AC021205.3 |
| AC021231.1 |
| AC021237.1 |
| AC021321.1 |
| AC021683.6 |
| AC021851.1 |
| AC022001.2 |
| AC022079.1 |
| AC022137.3 |
| AC022165.1 |
| AC022210.1 |
| AC022211.1 |
| AC022215.2 |
| AC022267.1 |
| AC022306.2 |
| AC022306.3 |
| AC022400.4 |
| AC022558.3 |
| AC022726.2 |
| AC022733.1 |
| AC022778.1 |
| AC022784.5 |
| AC022784.6 |
| AC022819.1 |
| AC022893.1 |
| AC022960.1 |
| AC022973.5 |
| AC023137.1 |
| AC023202.1 |
| AC023355.1 |
| AC023424.2 |
| AC023494.1 |
| AC023510.2 |
| AC023632.2 |
| AC023790.2 |
| AC023794.1 |
| AC023825.2 |
| AC023830.1 |
| AC023830.3 |
| AC023908.3 |
| AC024060.2 |
| AC024075.1 |
| AC024075.3 |
| AC024267.3 |
| AC024361.1 |
| AC024361.3 |
| AC024382.1 |
| AC024451.4 |
| AC024560.3 |
| AC024560.4 |
| AC024588.1 |
| AC024619.3 |
| AC024896.1 |
| AC024940.5 |
| AC025031.4 |
| AC025034.1 |
| AC025040.1 |
| AC025162.2 |
| AC025165.1 |
| AC025165.4 |
| AC025165.5 |
| AC025171.2 |
| AC025171.4 |
| AC025188.1 |
| AC025280.1 |
| AC025280.3 |
| AC025287.3 |
| AC025569.1 |
| AC025580.2 |
| AC025682.1 |
| AC025766.1 |
| AC025917.1 |
| AC026150.3 |
| AC026202.2 |
| AC026254.2 |
| AC026329.1 |
| AC026333.3 |
| AC026356.1 |
| AC026356.2 |
| AC026367.2 |
| AC026368.1 |
| AC026401.1 |
| AC026401.2 |
| AC026412.3 |
| AC026470.2 |
| AC026782.2 |
| AC026979.1 |
| AC027097.2 |
| AC027237.3 |
| AC027277.2 |
| AC027279.1 |
| AC027335.1 |
| AC027373.1 |
| AC027627.1 |
| AC027644.3 |
| AC027682.1 |
| AC027682.4 |
| AC027801.1 |
| AC027808.2 |
| AC027811.1 |
| AC034139.1 |
| AC034199.1 |
| AC034228.2 |
| AC034229.4 |
| AC036108.1 |
| AC036108.3 |
| AC036214.2 |
| AC037459.4 |
| AC037487.1 |
| AC040169.3 |
| AC040904.1 |
| AC040934.1 |
| AC046134.2 |
| AC048341.1 |
| AC048341.2 |
| AC048344.4 |
| AC048382.1 |
| AC053513.2 |
| AC055811.1 |
| AC058791.1 |
| AC060780.1 |
| AC061708.1 |
| AC061958.1 |
| AC061975.7 |
| AC061975.8 |
| AC063960.2 |
| AC064799.2 |
| AC064801.1 |
| AC064836.1 |
| AC064836.3 |
| AC064856.1 |
| AC066613.1 |
| AC067838.1 |
| AC068025.2 |
| AC068189.1 |
| AC068205.2 |
| AC068282.1 |
| AC068481.1 |
| AC068491.2 |
| AC068620.2 |
| AC068724.1 |
| AC068790.2 |
| AC068790.3 |
| AC068790.4 |
| AC068790.5 |
| AC068790.6 |
| AC068790.9 |
| AC068888.1 |
| AC068989.1 |
| AC069023.1 |
| AC069222.1 |
| AC069234.4 |
| AC069243.1 |
| AC069549.1 |
| AC072028.1 |
| AC073046.3 |
| AC073073.2 |
| AC073140.2 |
| AC073254.1 |
| AC073288.2 |
| AC073326.1 |
| AC073332.1 |
| AC073333.1 |
| AC073349.4 |
| AC073476.2 |
| AC073487.1 |
| AC073517.1 |
| AC073529.1 |
| AC073575.2 |
| AC073592.1 |
| AC073593.2 |
| AC073641.1 |
| AC073648.5 |
| AC073651.1 |
| AC073896.3 |
| AC073957.1 |
| AC073957.3 |
| AC074044.1 |
| AC074050.4 |
| AC074117.1 |
| AC074124.1 |
| AC078777.1 |
| AC078778.1 |
| AC078785.1 |
| AC078795.1 |
| AC078820.2 |
| AC078846.1 |
| AC078850.1 |
| AC078852.1 |
| AC078860.2 |
| AC078860.3 |
| AC078883.1 |
| AC078883.2 |
| AC078922.1 |
| AC078962.1 |
| AC078962.4 |
| AC078983.1 |
| AC079075.1 |
| AC079142.1 |
| AC079148.1 |
| AC079160.1 |
| AC079209.1 |
| AC079209.2 |
| AC079313.1 |
| AC079313.2 |
| AC079336.1 |
| AC079336.2 |
| AC079336.5 |
| AC079414.3 |
| AC079684.1 |
| AC079753.2 |
| AC079766.1 |
| AC079793.1 |
| AC079804.3 |
| AC079866.2 |
| AC079907.1 |
| AC080013.1 |
| AC080112.4 |
| AC080188.1 |
| AC083805.1 |
| AC083805.2 |
| AC083806.2 |
| AC083843.1 |
| AC083843.2 |
| AC083900.1 |
| AC083906.3 |
| AC084117.1 |
| AC084357.2 |
| AC084782.3 |
| AC084824.4 |
| AC084824.5 |
| AC084876.1 |
| AC087175.1 |
| AC087222.1 |
| AC087277.2 |
| AC087289.1 |
| AC087294.1 |
| AC087379.2 |
| AC087392.2 |
| AC087392.3 |
| AC087392.4 |
| AC087477.2 |
| AC087501.4 |
| AC087521.2 |
| AC087588.1 |
| AC087683.2 |
| AC087854.1 |
| AC089985.1 |
| AC090004.2 |
| AC090018.2 |
| AC090116.1 |
| AC090197.1 |
| AC090260.1 |
| AC090510.2 |
| AC090517.2 |
| AC090517.5 |
| AC090519.1 |
| AC090527.3 |
| AC090578.2 |
| AC090578.3 |
| AC090579.1 |
| AC090589.2 |
| AC090589.3 |
| AC090739.1 |
| AC090753.1 |
| AC090809.1 |
| AC090825.1 |
| AC090945.1 |
| AC090948.1 |
| AC090948.2 |
| AC091117.2 |
| AC091153.2 |
| AC091180.2 |
| AC091181.2 |
| AC091214.1 |
| AC091544.2 |
| AC091544.5 |
| AC091729.2 |
| AC091868.2 |
| AC091906.1 |
| AC092119.2 |
| AC092123.1 |
| AC092127.1 |
| AC092164.1 |
| AC092168.2 |
| AC092171.1 |
| AC092301.1 |
| AC092338.1 |
| AC092338.3 |
| AC092375.2 |
| AC092376.2 |
| AC092376.3 |
| AC092428.1 |
| AC092535.2 |
| AC092535.4 |
| AC092598.1 |
| AC092645.2 |
| AC092652.1 |
| AC092681.2 |
| AC092718.1 |
| AC092718.5 |
| AC092718.8 |
| AC092745.5 |
| AC092755.1 |
| AC092755.2 |
| AC092794.1 |
| AC092802.1 |
| AC092802.3 |
| AC092828.1 |
| AC092902.2 |
| AC092902.6 |
| AC092910.3 |
| AC092944.1 |
| AC093157.1 |
| AC093157.2 |
| AC093227.3 |
| AC093297.2 |
| AC093382.1 |
| AC093418.1 |
| AC093424.1 |
| AC093484.4 |
| AC093510.2 |
| AC093520.1 |
| AC093535.1 |
| AC093591.2 |
| AC093675.2 |
| AC093677.2 |
| AC093690.1 |
| AC093732.1 |
| AC093752.1 |
| AC093752.2 |
| AC093788.1 |
| AC093801.1 |
| AC093817.1 |
| AC093849.3 |
| AC095055.1 |
| AC096536.1 |
| AC096642.1 |
| AC096708.2 |
| AC096721.1 |
| AC096741.1 |
| AC096746.1 |
| AC096921.2 |
| AC096992.2 |
| AC097381.2 |
| AC097448.1 |
| AC097468.3 |
| AC097500.1 |
| AC097532.2 |
| AC097634.3 |
| AC097641.2 |
| AC097709.1 |
| AC097724.1 |
| AC098484.4 |
| AC098656.1 |
| AC098679.4 |
| AC098679.5 |
| AC098851.1 |
| AC098869.2 |
| AC099066.2 |
| AC099314.1 |
| AC099518.4 |
| AC099565.1 |
| AC099661.1 |
| AC099782.1 |
| AC099792.1 |
| AC099795.1 |
| AC099811.1 |
| AC100774.1 |
| AC100782.1 |
| AC100791.3 |
| AC100814.1 |
| AC100821.2 |
| AC103591.3 |
| AC103591.4 |
| AC103703.1 |
| AC103718.1 |
| AC103739.1 |
| AC103739.3 |
| AC103740.1 |
| AC103746.1 |
| AC103853.2 |
| AC103923.1 |
| AC103982.1 |
| AC104088.1 |
| AC104109.4 |
| AC104126.1 |
| AC104211.1 |
| AC104335.1 |
| AC104365.1 |
| AC104370.1 |
| AC104463.2 |
| AC104472.1 |
| AC104472.5 |
| AC104590.1 |
| AC104695.3 |
| AC104785.1 |
| AC104791.1 |
| AC104841.1 |
| AC104964.3 |
| AC104984.1 |
| AC104984.2 |
| AC105036.3 |
| AC105052.4 |
| AC105101.2 |
| AC105137.2 |
| AC105206.3 |
| AC105254.1 |
| AC105339.2 |
| AC105429.1 |
| AC105460.2 |
| AC106791.1 |
| AC106795.2 |
| AC106820.3 |
| AC106845.1 |
| AC106858.1 |
| AC106881.1 |
| AC106882.1 |
| AC106897.1 |
| AC107021.1 |
| AC107027.1 |
| AC107027.3 |
| AC107032.2 |
| AC107032.3 |
| AC107050.1 |
| AC107068.1 |
| AC107068.2 |
| AC107081.1 |
| AC107081.3 |
| AC107214.1 |
| AC107214.2 |
| AC107294.2 |
| AC107308.1 |
| AC107464.1 |
| AC107884.1 |
| AC107958.3 |
| AC107959.1 |
| AC108053.1 |
| AC108058.1 |
| AC108174.1 |
| AC108449.1 |
| AC108463.2 |
| AC108463.3 |
| AC108471.2 |
| AC108471.3 |
| AC108472.1 |
| AC108519.1 |
| AC108681.1 |
| AC108693.2 |
| AC108704.1 |
| AC108727.1 |
| AC109347.2 |
| AC109449.1 |
| AC109460.2 |
| AC109597.2 |
| AC109992.2 |
| AC110611.1 |
| AC110611.2 |
| AC110769.2 |
| AC110813.1 |
| AC112220.2 |
| AC112250.2 |
| AC112254.1 |
| AC112496.1 |
| AC112512.1 |
| AC112721.2 |
| AC112722.1 |
| AC113139.1 |
| AC113143.2 |
| AC113361.1 |
| AC114321.1 |
| AC114760.2 |
| AC114763.1 |
| AC114781.2 |
| AC114811.2 |
| AC114956.2 |
| AC114980.1 |
| AC115282.2 |
| AC115284.4 |
| AC116025.1 |
| AC116535.1 |
| AC116903.2 |
| AC116913.1 |
| AC118344.1 |
| AC119403.1 |
| AC120036.4 |
| AC120042.1 |
| AC120042.2 |
| AC120053.1 |
| AC120193.1 |
| AC122129.1 |
| AC122719.3 |
| AC123023.1 |
| AC123595.2 |
| AC123768.1 |
| AC123768.2 |
| AC124014.1 |
| AC124016.1 |
| AC124045.1 |
| AC124067.3 |
| AC124248.2 |
| AC124276.1 |
| AC124283.3 |
| AC124862.1 |
| AC125437.1 |
| AC125494.3 |
| AC126118.1 |
| AC126696.2 |
| AC126773.3 |
| AC127024.3 |
| AC127035.1 |
| AC127070.2 |
| AC127164.1 |
| AC127496.3 |
| AC127526.1 |
| AC127526.4 |
| AC128687.3 |
| AC129510.2 |
| AC130371.1 |
| AC130456.1 |
| AC130650.2 |
| AC131025.3 |
| AC131159.1 |
| AC131568.1 |
| AC131571.1 |
| AC131934.1 |
| AC131953.1 |
| AC131971.1 |
| AC132192.1 |
| AC132192.2 |
| AC132938.2 |
| AC133540.1 |
| AC133644.1 |
| AC134407.1 |
| AC135050.4 |
| AC135050.6 |
| AC135178.4 |
| AC135279.3 |
| AC137770.1 |
| AC137932.2 |
| AC137932.3 |
| AC138123.2 |
| AC138207.4 |
| AC138646.1 |
| AC138932.4 |
| AC138956.1 |
| AC138956.2 |
| AC139019.1 |
| AC139100.1 |
| AC139720.2 |
| AC139795.2 |
| AC139887.2 |
| AC141002.1 |
| AC145146.1 |
| AC145207.8 |
| AC145285.3 |
| AC145423.1 |
| AC145423.2 |
| AC146507.3 |
| AC211433.1 |
| AC211476.2 |
| AC215522.2 |
| AC232271.1 |
| AC234917.1 |
| AC239584.1 |
| AC243772.2 |
| AC243967.2 |
| AC244033.2 |
| AC244093.3 |
| AC244093.4 |
| AC244093.5 |
| AC244100.2 |
| AC244517.1 |
| AC244517.7 |
| AC245060.5 |
| AC245884.10 |
| AC245884.8 |
| AC246817.1 |
| AC246817.2 |
| AC254562.2 |
| ACBD3-AS1 |
| ACOXL-AS1 |
| ACSL3-AS1 |
| ACTA2-AS1 |
| AD001527.1 |
| ADAMTS9-AS1 |
| ADAMTS9-AS2 |
| ADD3-AS1 |
| ADNP-AS1 |
| AF111169.4 |
| AF117829.1 |
| AF127936.1 |
| AF129075.3 |
| AF165147.1 |
| AF230666.1 |
| AF230666.2 |
| AF235103.3 |
| AF241728.2 |
| AF287957.1 |
| AFDN-DT |
| AIRN |
| AJ239328.1 |
| AL008718.3 |
| AL008729.1 |
| AL020995.1 |
| AL020997.2 |
| AL021155.4 |
| AL021328.1 |
| AL021392.1 |
| AL021578.1 |
| AL021707.2 |
| AL021707.4 |
| AL021707.7 |
| AL021918.4 |
| AL021978.1 |
| AL022069.1 |
| AL022157.1 |
| AL022311.1 |
| AL022322.1 |
| AL023755.1 |
| AL023882.1 |
| AL024498.1 |
| AL031005.1 |
| AL031275.1 |
| AL031429.2 |
| AL031432.3 |
| AL031432.4 |
| AL031595.3 |
| AL031602.2 |
| AL031651.2 |
| AL031663.3 |
| AL031666.1 |
| AL031666.2 |
| AL031667.3 |
| AL031670.1 |
| AL031673.1 |
| AL031705.1 |
| AL031710.1 |
| AL031710.2 |
| AL031716.1 |
| AL031717.1 |
| AL031770.1 |
| AL031775.1 |
| AL031775.2 |
| AL031985.3 |
| AL032819.1 |
| AL033381.3 |
| AL033397.2 |
| AL033504.1 |
| AL033527.3 |
| AL033543.1 |
| AL034405.1 |
| AL034417.4 |
| AL034422.1 |
| AL034550.1 |
| AL034550.3 |
| AL035071.1 |
| AL035071.2 |
| AL035078.1 |
| AL035411.3 |
| AL035416.1 |
| AL035448.1 |
| AL035458.2 |
| AL035530.2 |
| AL035693.1 |
| AL049539.1 |
| AL049552.2 |
| AL049646.1 |
| AL049779.4 |
| AL049780.1 |
| AL049838.1 |
| AL049840.2 |
| AL049840.5 |
| AL049870.3 |
| AL050320.1 |
| AL050403.2 |
| AL078581.2 |
| AL078600.1 |
| AL078604.2 |
| AL080276.2 |
| AL080317.1 |
| AL080317.2 |
| AL096701.3 |
| AL096794.1 |
| AL109614.1 |
| AL109615.2 |
| AL109615.4 |
| AL109761.1 |
| AL109767.1 |
| AL109804.1 |
| AL109811.1 |
| AL109840.2 |
| AL109923.1 |
| AL109984.1 |
| AL110114.1 |
| AL110115.1 |
| AL110115.2 |
| AL117327.1 |
| AL117344.2 |
| AL117379.1 |
| AL117382.1 |
| AL121583.1 |
| AL121750.1 |
| AL121772.2 |
| AL121782.1 |
| AL121845.4 |
| AL121895.1 |
| AL121895.2 |
| AL121906.1 |
| AL121929.3 |
| AL121933.2 |
| AL121935.2 |
| AL121987.1 |
| AL121987.2 |
| AL121992.3 |
| AL122035.1 |
| AL122125.1 |
| AL132642.1 |
| AL132657.1 |
| AL132765.2 |
| AL132780.1 |
| AL133230.1 |
| AL133243.2 |
| AL133243.3 |
| AL133255.1 |
| AL133259.1 |
| AL133268.4 |
| AL133284.1 |
| AL133330.1 |
| AL133342.1 |
| AL133343.2 |
| AL133371.2 |
| AL133406.2 |
| AL133410.1 |
| AL133481.1 |
| AL133520.1 |
| AL133551.1 |
| AL135784.1 |
| AL135786.2 |
| AL135841.1 |
| AL135902.1 |
| AL135926.2 |
| AL135937.1 |
| AL136115.1 |
| AL136115.2 |
| AL136141.1 |
| AL136169.1 |
| AL136171.2 |
| AL136361.1 |
| AL136526.1 |
| AL136985.2 |
| AL136988.2 |
| AL137003.1 |
| AL137009.1 |
| AL137058.2 |
| AL137139.3 |
| AL137244.1 |
| AL137246.1 |
| AL137779.2 |
| AL137782.1 |
| AL137847.1 |
| AL137856.1 |
| AL138689.1 |
| AL138733.1 |
| AL138756.1 |
| AL138759.1 |
| AL138820.1 |
| AL138921.1 |
| AL138962.1 |
| AL138963.1 |
| AL138999.1 |
| AL138999.2 |
| AL139021.1 |
| AL139081.1 |
| AL139082.1 |
| AL139147.1 |
| AL139274.2 |
| AL139287.1 |
| AL139294.1 |
| AL139300.2 |
| AL139317.5 |
| AL139383.1 |
| AL139807.1 |
| AL157392.2 |
| AL157392.4 |
| AL157395.1 |
| AL157400.2 |
| AL157400.3 |
| AL157400.4 |
| AL157786.1 |
| AL157813.2 |
| AL157827.2 |
| AL157834.2 |
| AL157838.1 |
| AL157871.5 |
| AL157884.2 |
| AL157895.1 |
| AL157932.1 |
| AL157938.3 |
| AL158063.1 |
| AL158151.4 |
| AL158163.1 |
| AL158163.2 |
| AL158195.1 |
| AL158198.2 |
| AL158207.2 |
| AL158211.1 |
| AL158212.2 |
| AL158212.3 |
| AL158825.2 |
| AL158829.1 |
| AL160006.1 |
| AL160171.1 |
| AL160286.3 |
| AL160290.1 |
| AL160314.2 |
| AL161430.1 |
| AL161443.1 |
| AL161457.1 |
| AL161663.1 |
| AL161663.2 |
| AL161725.2 |
| AL161729.2 |
| AL161729.3 |
| AL161729.4 |
| AL161756.1 |
| AL161756.3 |
| AL161891.1 |
| AL162386.2 |
| AL162424.1 |
| AL162586.2 |
| AL162595.1 |
| AL162724.2 |
| AL162741.1 |
| AL353593.2 |
| AL353708.3 |
| AL353796.1 |
| AL353801.1 |
| AL353801.3 |
| AL353804.2 |
| AL354696.1 |
| AL354696.2 |
| AL354726.1 |
| AL354733.2 |
| AL354793.1 |
| AL354813.1 |
| AL354833.1 |
| AL354892.3 |
| AL354979.1 |
| AL354989.1 |
| AL354993.1 |
| AL354993.2 |
| AL355001.1 |
| AL355073.1 |
| AL355073.2 |
| AL355075.2 |
| AL355075.6 |
| AL355076.2 |
| AL355112.1 |
| AL355303.2 |
| AL355312.2 |
| AL355377.4 |
| AL355385.1 |
| AL355388.1 |
| AL355472.3 |
| AL355488.1 |
| AL355834.2 |
| AL355835.1 |
| AL355916.2 |
| AL355922.1 |
| AL355922.3 |
| AL355922.4 |
| AL355999.1 |
| AL356124.1 |
| AL356299.2 |
| AL356475.1 |
| AL356481.2 |
| AL356608.3 |
| AL356752.1 |
| AL356804.1 |
| AL356966.1 |
| AL357079.3 |
| AL357140.4 |
| AL357873.1 |
| AL357874.2 |
| AL358072.1 |
| AL358115.1 |
| AL358216.1 |
| AL358472.2 |
| AL358472.3 |
| AL358472.5 |
| AL359232.1 |
| AL359317.2 |
| AL359636.2 |
| AL359643.2 |
| AL359878.1 |
| AL359880.1 |
| AL359921.1 |
| AL359922.2 |
| AL359962.2 |
| AL360091.1 |
| AL360093.1 |
| AL360178.1 |
| AL360219.1 |
| AL360270.1 |
| AL360270.2 |
| AL360270.3 |
| AL365361.1 |
| AL365436.2 |
| AL390066.2 |
| AL390195.1 |
| AL390195.2 |
| AL390242.1 |
| AL390719.3 |
| AL390728.4 |
| AL390755.3 |
| AL390957.1 |
| AL390961.2 |
| AL391421.1 |
| AL391427.1 |
| AL391684.1 |
| AL391832.3 |
| AL391834.1 |
| AL392089.1 |
| AL442067.1 |
| AL442067.2 |
| AL442125.1 |
| AL442125.2 |
| AL442128.2 |
| AL445222.1 |
| AL445223.1 |
| AL445231.1 |
| AL445309.1 |
| AL445489.1 |
| AL449214.1 |
| AL450344.3 |
| AL450992.2 |
| AL450998.2 |
| AL451069.2 |
| AL451085.1 |
| AL451123.1 |
| AL512283.1 |
| AL512283.3 |
| AL512288.1 |
| AL512328.1 |
| AL512353.1 |
| AL512506.1 |
| AL512603.2 |
| AL512643.1 |
| AL512643.2 |
| AL512652.1 |
| AL512656.1 |
| AL513123.1 |
| AL513327.1 |
| AL513327.2 |
| AL513534.2 |
| AL513550.1 |
| AL583824.1 |
| AL589745.1 |
| AL589745.2 |
| AL590064.1 |
| AL590096.1 |
| AL590101.1 |
| AL590282.2 |
| AL590483.4 |
| AL590723.1 |
| AL590729.1 |
| AL590822.2 |
| AL591069.1 |
| AL591212.1 |
| AL591506.1 |
| AL591518.1 |
| AL591848.3 |
| AL592148.3 |
| AL592166.1 |
| AL592301.1 |
| AL592430.1 |
| AL592431.1 |
| AL592546.3 |
| AL596202.1 |
| AL596214.1 |
| AL596223.1 |
| AL596247.1 |
| AL596325.2 |
| AL604028.1 |
| AL606489.1 |
| AL606537.1 |
| AL606834.2 |
| AL607028.1 |
| AL645768.1 |
| AL683813.1 |
| AL691447.2 |
| AL713852.1 |
| AL731537.1 |
| AL731563.3 |
| AL731566.2 |
| AL731569.1 |
| AL731571.1 |
| AL732509.1 |
| AL807757.2 |
| AL929236.1 |
| ALG13-AS1 |
| ALG14-AS1 |
| ALKBH3-AS1 |
| ALMS1-IT1 |
| ANKRD10-IT1 |
| AOAH-IT1 |
| AP000253.1 |
| AP000254.2 |
| AP000331.1 |
| AP000350.6 |
| AP000442.1 |
| AP000487.1 |
| AP000593.3 |
| AP000640.2 |
| AP000662.1 |
| AP000679.1 |
| AP000692.1 |
| AP000704.1 |
| AP000763.3 |
| AP000786.1 |
| AP000813.1 |
| AP000844.1 |
| AP000866.1 |
| AP000866.6 |
| AP000873.2 |
| AP000919.1 |
| AP000977.1 |
| AP001001.1 |
| AP001029.2 |
| AP001037.1 |
| AP001094.2 |
| AP001107.1 |
| AP001107.4 |
| AP001160.1 |
| AP001160.4 |
| AP001267.3 |
| AP001269.1 |
| AP001347.1 |
| AP001372.2 |
| AP001429.1 |
| AP001442.1 |
| AP001469.2 |
| AP001469.3 |
| AP001528.1 |
| AP001619.1 |
| AP001885.3 |
| AP001893.1 |
| AP001922.3 |
| AP002026.1 |
| AP002336.2 |
| AP002449.1 |
| AP002490.1 |
| AP002748.6 |
| AP002784.1 |
| AP002812.3 |
| AP002907.1 |
| AP002982.2 |
| AP002985.1 |
| AP002993.1 |
| AP003059.2 |
| AP003086.1 |
| AP003110.1 |
| AP003170.4 |
| AP003352.1 |
| AP003354.1 |
| AP003392.1 |
| AP003392.4 |
| AP003419.2 |
| AP003498.1 |
| AP003548.1 |
| AP003783.1 |
| AP005059.1 |
| AP005131.1 |
| AP005230.1 |
| AP005482.2 |
| AP006259.1 |
| AP006333.2 |
| AP006545.1 |
| AP006545.2 |
| AP006545.3 |
| AP007216.2 |
| AP4B1-AS1 |
| ARAP1-AS2 |
| ARHGAP15-AS1 |
| ARHGAP27P1-BPTFP1-KPNA2P3 |
| ARHGAP42-AS1 |
| ARHGEF35-AS1 |
| ARHGEF38-IT1 |
| ARNTL2-AS1 |
| ARRDC3-AS1 |
| ASAP1-IT2 |
| ASH1L-AS1 |
| ASTN2-AS1 |
| ATP11A-AS1 |
| ATP1B3-AS1 |
| ATP2B1-AS1 |
| AUXG01000058.1 |
| B4GALT4-AS1 |
| BAALC-AS1 |
| BACE1-AS |
| BACH1-IT1 |
| BCDIN3D-AS1 |
| BCL2L1-AS1 |
| BDNF-AS |
| BMP7-AS1 |
| BMS1P4 |
| BNC2-AS1 |
| BX322234.1 |
| BX842570.1 |
| C15orf54 |
| C1orf147 |
| C1RL-AS1 |
| C2-AS1 |
| C2orf27A |
| C3orf35 |
| C5orf66 |
| C7orf69 |
| C8orf44 |
| C9orf147 |
| CAPN10-DT |
| CARMN |
| CARNMT1-AS1 |
| CARS1-AS1 |
| CASC15 |
| CASC18 |
| CASC19 |
| CASC2 |
| CASK-AS1 |
| CATIP-AS2 |
| CBR3-AS1 |
| CCAT2 |
| CCDC13-AS1 |
| CCDC18-AS1 |
| CCDC28A-AS1 |
| CCNT2-AS1 |
| CD44-AS1 |
| CDC42-AS1 |
| CDC42-IT1 |
| CEP83-DT |
| CERNA1 |
| CERS6-AS1 |
| CFAP44-AS1 |
| CFTR-AS1 |
| CHN2-AS1 |
| CHRM3-AS2 |
| CLDN10-AS1 |
| COL4A2-AS2 |
| COX10-AS1 |
| CRAT37 |
| CRNDE |
| CRPPA-AS1 |
| CRTC3-AS1 |
| CRYZL2P-SEC16B |
| CSE1L-AS1 |
| CSNK1G2-AS1 |
| CTBP1-AS |
| CYP1B1-AS1 |
| CYP2U1-AS1 |
| CYTOR |
| DACT3-AS1 |
| DANT2 |
| DCUN1D2-AS |
| DELEC1 |
| DEPDC1-AS1 |
| DGCR11 |
| DGUOK-AS1 |
| DIAPH2-AS1 |
| DLEU1 |
| DLEU2 |
| DLGAP4-AS1 |
| DNAJC3-DT |
| DNAJC9-AS1 |
| DNMBP-AS1 |
| DPH6-DT |
| DPYD-AS1 |
| DSG2-AS1 |
| DTX2P1-UPK3BP1-PMS2P11 |
| DUBR |
| EBLN3P |
| ECE1-AS1 |
| EDIL3-DT |
| EGOT |
| EHD4-AS1 |
| EIF1AX-AS1 |
| EIPR1-IT1 |
| ELOA-AS1 |
| ENTPD1-AS1 |
| ENTPD3-AS1 |
| EP300-AS1 |
| EPN2-AS1 |
| EPS15-AS1 |
| ERCC8-AS1 |
| EXTL3-AS1 |
| FAM106A |
| FAM111A-DT |
| FAM13A-AS1 |
| FAM160A1-DT |
| FAM218A |
| FAM53B-AS1 |
| FAM66C |
| FAM78B-AS1 |
| FAM87B |
| FARP1-AS1 |
| FBXO30-DT |
| FER1L6-AS1 |
| FER1L6-AS2 |
| FGD5-AS1 |
| FIRRE |
| FLNB-AS1 |
| FMR1-AS1 |
| FMR1-IT1 |
| FO393418.1 |
| FO680682.1 |
| FOCAD-AS1 |
| FOXN3-AS2 |
| FRMD6-AS1 |
| FRMD6-AS2 |
| FSIP2-AS1 |
| FSIP2-AS2 |
| FTX |
| G2E3-AS1 |
| GABPB1-AS1 |
| GARS1-DT |
| GAS1RR |
| GAS5-AS1 |
| GAS8-AS1 |
| GEMIN7-AS1 |
| GHRLOS |
| GK-AS1 |
| GK-IT1 |
| GLYCTK-AS1 |
| GNG12-AS1 |
| GORAB-AS1 |
| GRASLND |
| GRM8-AS1 |
| GRTP1-AS1 |
| GSN-AS1 |
| GSTCD-AS1 |
| GTF3C2-AS1 |
| H2AZ1-DT |
| HAS2-AS1 |
| HCFC1-AS1 |
| HCG18 |
| HCG25 |
| HCP5B |
| HDAC2-AS2 |
| HECW1-IT1 |
| HECW2-AS1 |
| HEXD-IT1 |
| HIF1A-AS3 |
| HLA-F-AS1 |
| HMGA2-AS1 |
| HOXA-AS3 |
| HYI-AS1 |
| HYMAI |
| IDI2-AS1 |
| IFNG-AS1 |
| INE1 |
| INHBA-AS1 |
| INTS6-AS1 |
| INTS9-AS1 |
| IQCH-AS1 |
| IRF1-AS1 |
| ISM1-AS1 |
| ITFG1-AS1 |
| ITGA6-AS1 |
| ITGA9-AS1 |
| ITGB5-AS1 |
| ITPK1-AS1 |
| ITPKB-AS1 |
| ITPKB-IT1 |
| ITPRIP-AS1 |
| JARID2-AS1 |
| JMJD1C-AS1 |
| JPX |
| KANSL1L-AS1 |
| KCNIP2-AS1 |
| KCNMA1-AS3 |
| KCNMB2-AS1 |
| KCNQ1OT1 |
| KDM4A-AS1 |
| KIAA1614-AS1 |
| KIF26B-AS1 |
| KIF9-AS1 |
| KLF7-IT1 |
| KLHL7-DT |
| KTN1-AS1 |
| L29074.1 |
| L3MBTL4-AS1 |
| LACTB2-AS1 |
| LAMC1-AS1 |
| LAMTOR5-AS1 |
| LCMT1-AS1 |
| LDLRAD4-AS1 |
| LEF1-AS1 |
| LEMD1-AS1 |
| LENG8-AS1 |
| LGR4-AS1 |
| LIMD1-AS1 |
| LINC-PINT |
| LINC00092 |
| LINC00189 |
| LINC00216 |
| LINC00240 |
| LINC00244 |
| LINC00271 |
| LINC00299 |
| LINC00339 |
| LINC00365 |
| LINC00412 |
| LINC00449 |
| LINC00467 |
| LINC00472 |
| LINC00476 |
| LINC00511 |
| LINC00513 |
| LINC00535 |
| LINC00539 |
| LINC00562 |
| LINC00571 |
| LINC00578 |
| LINC00622 |
| LINC00624 |
| LINC00626 |
| LINC00628 |
| LINC00630 |
| LINC00641 |
| LINC00654 |
| LINC00658 |
| LINC00662 |
| LINC00698 |
| LINC00861 |
| LINC00862 |
| LINC00863 |
| LINC00867 |
| LINC00893 |
| LINC00894 |
| LINC00910 |
| LINC00923 |
| LINC00937 |
| LINC00954 |
| LINC00968 |
| LINC00997 |
| LINC01004 |
| LINC01012 |
| LINC01036 |
| LINC01050 |
| LINC01055 |
| LINC01091 |
| LINC01106 |
| LINC01126 |
| LINC01138 |
| LINC01140 |
| LINC01176 |
| LINC01184 |
| LINC01191 |
| LINC01197 |
| LINC01210 |
| LINC01276 |
| LINC01285 |
| LINC01290 |
| LINC01303 |
| LINC01322 |
| LINC01338 |
| LINC01352 |
| LINC01355 |
| LINC01358 |
| LINC01359 |
| LINC01366 |
| LINC01376 |
| LINC01409 |
| LINC01410 |
| LINC01422 |
| LINC01424 |
| LINC01429 |
| LINC01473 |
| LINC01504 |
| LINC01537 |
| LINC01545 |
| LINC01572 |
| LINC01585 |
| LINC01607 |
| LINC01611 |
| LINC01622 |
| LINC01655 |
| LINC01705 |
| LINC01719 |
| LINC01748 |
| LINC01762 |
| LINC01772 |
| LINC01806 |
| LINC01811 |
| LINC01814 |
| LINC01829 |
| LINC01851 |
| LINC01967 |
| LINC02018 |
| LINC02027 |
| LINC02035 |
| LINC02042 |
| LINC02050 |
| LINC02100 |
| LINC02126 |
| LINC02156 |
| LINC02158 |
| LINC02163 |
| LINC02175 |
| LINC02198 |
| LINC02254 |
| LINC02257 |
| LINC02293 |
| LINC02294 |
| LINC02328 |
| LINC02334 |
| LINC02408 |
| LINC02416 |
| LINC02421 |
| LINC02428 |
| LINC02433 |
| LINC02435 |
| LINC02447 |
| LINC02532 |
| LINC02539 |
| LINC02542 |
| LINC02561 |
| LINC02569 |
| LINC02576 |
| LINC02584 |
| LINC02595 |
| LINC02603 |
| LINC02615 |
| LINC02633 |
| LINC02649 |
| LINC02656 |
| LINC02666 |
| LINC02669 |
| LINC02693 |
| LINC02718 |
| LINC02762 |
| LINC02768 |
| LINC02795 |
| LINC02831 |
| LINC02883 |
| LMCD1-AS1 |
| LMO7-AS1 |
| LMO7DN-IT1 |
| LNCOC1 |
| LNCSRLR |
| LRP1-AS |
| LRP4-AS1 |
| LRRC2-AS1 |
| LRRC3-DT |
| LRRC8C-DT |
| LRRK2-DT |
| LUCAT1 |
| LURAP1L-AS1 |
| LYRM4-AS1 |
| MACC1-AS1 |
| MAFTRR |
| MAGI1-IT1 |
| MAGI2-AS3 |
| MAILR |
| MAL2-AS1 |
| MALAT1 |
| MALINC1 |
| MAMDC2-AS1 |
| MANEA-DT |
| MAP3K2-DT |
| MAP3K5-AS1 |
| MAPKAPK5-AS1 |
| MAST4-AS1 |
| MBNL1-AS1 |
| MCCC1-AS1 |
| MCM3AP-AS1 |
| MCPH1-AS1 |
| MED4-AS1 |
| MED8-AS1 |
| MEF2C-AS1 |
| MEIS1-AS2 |
| MESTIT1 |
| MIATNB |
| MIR100HG |
| MIR133A1HG |
| MIR155HG |
| MIR17HG |
| MIR181A1HG |
| MIR181A2HG |
| MIR2052HG |
| MIR222HG |
| MIR223HG |
| MIR29B2CHG |
| MIR302CHG |
| MIR3150BHG |
| MIR31HG |
| MIR378D2HG |
| MIR3936HG |
| MIR4435-2HG |
| MIR4453HG |
| MIR646HG |
| MIR924HG |
| MIRLET7A1HG |
| MIS18A-AS1 |
| MKLN1-AS |
| MORF4L2-AS1 |
| MPRIP-AS1 |
| MRAP-AS1 |
| MRPS30-DT |
| MRPS9-AS1 |
| MSC-AS1 |
| MSH2-OT1 |
| MYB-AS1 |
| MYLK-AS1 |
| MYOSLID |
| N4BP2L2-IT2 |
| NAALADL2-AS3 |
| NADK2-AS1 |
| NARF-AS1 |
| NARF-IT1 |
| NCBP2-AS1 |
| NCK1-DT |
| NCKAP5-AS2 |
| NDUFV2-AS1 |
| NEAT1 |
| NECTIN1-AS1 |
| NFYC-AS1 |
| NHS-AS1 |
| NKILA |
| NNT-AS1 |
| NORAD |
| NPAS2-AS1 |
| NPSR1-AS1 |
| NR2F2-AS1 |
| NSMCE1-DT |
| NUTM2B-AS1 |
| NXT1-AS1 |
| ODF2-AS1 |
| OIP5-AS1 |
| OSBPL10-AS1 |
| OSGEPL1-AS1 |
| OTUD6B-AS1 |
| OVCH1-AS1 |
| OVOL1-AS1 |
| PABPC4-AS1 |
| PAN3-AS1 |
| PAXIP1-AS2 |
| PCAT1 |
| PCBP1-AS1 |
| PCBP2-OT1 |
| PCSK6-AS1 |
| PDC-AS1 |
| PDE9A-AS1 |
| PDXP-DT |
| PEF1-AS1 |
| PEG13 |
| PHACTR2-AS1 |
| PHC2-AS1 |
| PIK3IP1-DT |
| PITRM1-AS1 |
| PKP4-AS1 |
| PLBD1-AS1 |
| PLCE1-AS1 |
| PLCG1-AS1 |
| PLCL2-AS1 |
| PLS1-AS1 |
| PLS3-AS1 |
| POC1B-AS1 |
| PPM1F-AS1 |
| PPP1R12A-AS1 |
| PPP1R14B-AS1 |
| PPP3CB-AS1 |
| PRANCR |
| PRC1-AS1 |
| PRDX6-AS1 |
| PRICKLE2-AS2 |
| PRKCQ-AS1 |
| PRMT5-AS1 |
| PRNCR1 |
| PROSER2-AS1 |
| PRR34 |
| PRR7-AS1 |
| PSMD6-AS2 |
| PSPC1-AS2 |
| PTOV1-AS1 |
| PTPRG-AS1 |
| PURPL |
| PVT1 |
| RAB30-DT |
| RAP2C-AS1 |
| RASA3-IT1 |
| RASGRF2-AS1 |
| RASGRP3-AS1 |
| RB1-DT |
| RBFADN |
| RBM26-AS1 |
| RDH10-AS1 |
| RERE-AS1 |
| RERG-IT1 |
| RHOA-IT1 |
| RHOQ-AS1 |
| RNASEH2B-AS1 |
| RNF139-AS1 |
| RNF157-AS1 |
| RNF213-AS1 |
| RNF216-IT1 |
| RNF216P1 |
| RNF217-AS1 |
| RNF32-AS1 |
| RPARP-AS1 |
| RPL37A-DT |
| RPP38-DT |
| RPS6KA2-IT1 |
| RRN3P2 |
| RRS1-AS1 |
| RSF1-IT1 |
| RTCA-AS1 |
| RUSC1-AS1 |
| SAMD12-AS1 |
| SAP30-DT |
| SAP30L-AS1 |
| SBF2-AS1 |
| SCAMP1-AS1 |
| SCARNA9 |
| SCAT2 |
| SCG5-AS1 |
| SDK1-AS1 |
| SEC24B-AS1 |
| SEMA3F-AS1 |
| SEMA6A-AS1 |
| SEMA6A-AS2 |
| SEPSECS-AS1 |
| SEPTIN7-DT |
| SGMS1-AS1 |
| SH3TC2-DT |
| SHANK2-AS2 |
| SIDT1-AS1 |
| SKAP1-AS1 |
| SLC16A1-AS1 |
| SLC24A3-AS1 |
| SLC25A5-AS1 |
| SLC2A1-AS1 |
| SLC2A9-AS1 |
| SLC5A4-AS1 |
| SLC7A11-AS1 |
| SLC9A9-AS1 |
| SLFNL1-AS1 |
| SLIT2-IT1 |
| SMAD9-IT1 |
| SMC5-AS1 |
| SMG7-AS1 |
| SMILR |
| SMIM2-IT1 |
| SND1-IT1 |
| SNHG1 |
| SNHG14 |
| SNHG16 |
| SNHG22 |
| SNHG31 |
| SNHG32 |
| SNHG4 |
| SNRK-AS1 |
| SOS1-IT1 |
| SOX9-AS1 |
| SP2-AS1 |
| SPAG5-AS1 |
| SPON1-AS1 |
| SPRY4-AS1 |
| SREBF2-AS1 |
| SRI-AS1 |
| ST7-AS2 |
| ST7-OT4 |
| STAM-AS1 |
| STEAP2-AS1 |
| STEAP3-AS1 |
| STX17-AS1 |
| STX18-AS1 |
| STXBP5-AS1 |
| SUCLG2-AS1 |
| SUGCT-AS1 |
| SUGT1-DT |
| SYNJ2-IT1 |
| TAF1A-AS1 |
| TAX1BP1-AS1 |
| TBL1XR1-AS1 |
| TFAP2A-AS2 |
| THAP9-AS1 |
| THCAT158 |
| THOC7-AS1 |
| THORLNC |
| THUMPD3-AS1 |
| TLX1NB |
| TMC3-AS1 |
| TMCC1-AS1 |
| TMED2-DT |
| TMEM139-AS1 |
| TMEM202-AS1 |
| TMEM30A-DT |
| TMEM75 |
| TMEM9B-AS1 |
| TNFRSF10A-AS1 |
| TNS1-AS1 |
| TPM1-AS |
| TPT1-AS1 |
| TRAF3IP2-AS1 |
| TRG-AS1 |
| TRIM36-IT1 |
| TSC22D1-AS1 |
| TSPOAP1-AS1 |
| TTC21B-AS1 |
| TTC28-AS1 |
| TTC3-AS1 |
| TTC39C-AS1 |
| TTN-AS1 |
| U91328.1 |
| UBA6-AS1 |
| UBE2Q1-AS1 |
| UBOX5-AS1 |
| UBR5-AS1 |
| UBXN7-AS1 |
| UGDH-AS1 |
| USP3-AS1 |
| VAC14-AS1 |
| VIPR1-AS1 |
| VPS13B-DT |
| WAKMAR2 |
| WARS2-AS1 |
| WARS2-IT1 |
| WASHC5-AS1 |
| WASL-DT |
| WDFY3-AS2 |
| WNT5A-AS1 |
| YEATS2-AS1 |
| Z68871.1 |
| Z69666.1 |
| Z69733.1 |
| Z80897.1 |
| Z82217.1 |
| Z82243.1 |
| Z83843.1 |
| Z84485.1 |
| Z93022.1 |
| Z93930.3 |
| Z94721.1 |
| Z94721.2 |
| Z94721.3 |
| Z95118.2 |
| Z95331.1 |
| Z97192.1 |
| Z97200.1 |
| Z97832.2 |
| Z97989.1 |
| Z98884.2 |
| Z98885.3 |
| Z99127.3 |
| Z99289.2 |
| Z99289.3 |
| Z99496.1 |
| Z99572.1 |
| ZBED3-AS1 |
| ZBTB20-AS4 |
| ZBTB20-AS5 |
| ZDHHC20-IT1 |
| ZEB1-AS1 |
| ZFHX2-AS1 |
| ZFPM2-AS1 |
| ZKSCAN2-DT |
| ZKSCAN7-AS1 |
| ZMIZ1-AS1 |
| ZNF213-AS1 |
| ZNF252P-AS1 |
| ZNF32-AS1 |
| ZNF32-AS2 |
| ZNF337-AS1 |
| ZNF346-IT1 |
| ZNF433-AS1 |
| ZNF451-AS1 |
| ZNF460-AS1 |
| ZNF561-AS1 |
| ZNF571-AS1 |
| ZNF630-AS1 |
| ZNF674-AS1 |
| ZNF775-AS1 |
| ZNF790-AS1 |
| ZNF8-ERVK3-1 |
| ZNNT1 |
| ZNRF3-AS1 |
| ZNRF3-IT1 |
| ZRANB2-AS1 |
| ZRANB2-AS2 |

**Supplementary Table S3.** 1111 differentially expressed lncRNAs between normal and cancer samples of TCGA data.

| gene | conMean | treatMean | logFC | pValue | fdr |
| --- | --- | --- | --- | --- | --- |
| A2M-AS1 | 1.203726 | 0.560113 | -1.10372 | 4.17E-09 | 1.76E-08 |
| ABCA9-AS1 | 0.015716 | 0.147002 | 3.2255 | 3.48E-05 | 6.79E-05 |
| AC000061.1 | 0.842788 | 2.511557 | 1.57534 | 6.74E-05 | 0.000123 |
| AC000065.1 | 0.07286 | 0.530323 | 2.863664 | 3.20E-05 | 6.32E-05 |
| AC000072.1 | 0.056853 | 0.194834 | 1.776923 | 0.007965 | 0.009532 |
| AC000082.1 | 0.289756 | 0.088398 | -1.71276 | 1.20E-14 | 1.48E-13 |
| AC000123.1 | 2.263265 | 4.662818 | 1.042796 | 4.04E-07 | 1.14E-06 |
| AC001226.1 | 0.145809 | 0.293998 | 1.011726 | 0.029681 | 0.031121 |
| AC002306.1 | 0.071963 | 0.268851 | 1.901485 | 0.003781 | 0.004853 |
| AC002463.1 | 0.048302 | 0.146556 | 1.601286 | 0.003315 | 0.004328 |
| AC002542.6 | 0.085465 | 0.449823 | 2.39595 | 1.20E-06 | 3.10E-06 |
| AC002546.1 | 0.518379 | 0.182653 | -1.5049 | 3.55E-18 | 1.05E-16 |
| AC002563.1 | 0.112788 | 0.332974 | 1.561792 | 0.021934 | 0.023786 |
| AC003991.1 | 0.60274 | 0.286523 | -1.07288 | 1.33E-13 | 1.35E-12 |
| AC004000.1 | 0.37446 | 0.959363 | 1.357263 | 0.021901 | 0.023763 |
| AC004039.1 | 0.057667 | 0.23214 | 2.009166 | 3.89E-06 | 9.17E-06 |
| AC004076.2 | 0.3186 | 0.783221 | 1.297673 | 4.29E-10 | 2.16E-09 |
| AC004148.1 | 2.153402 | 4.339754 | 1.010995 | 6.00E-10 | 2.97E-09 |
| AC004223.2 | 0.13184 | 0.809615 | 2.618453 | 0.000897 | 0.001306 |
| AC004241.4 | 0.13767 | 0.384701 | 1.482527 | 1.39E-06 | 3.55E-06 |
| AC004253.1 | 1.072153 | 2.340888 | 1.126544 | 1.86E-07 | 5.52E-07 |
| AC004466.1 | 0.056223 | 0.113447 | 1.012779 | 0.005713 | 0.007067 |
| AC004466.2 | 0.173977 | 0.44315 | 1.3489 | 0.018256 | 0.020142 |
| AC004466.3 | 0.162449 | 0.565316 | 1.799072 | 1.29E-05 | 2.74E-05 |
| AC004554.1 | 0.333672 | 0.082464 | -2.0166 | 5.70E-16 | 9.78E-15 |
| AC004584.1 | 0.19237 | 0.411444 | 1.096814 | 0.000135 | 0.000232 |
| AC004593.1 | 0.115716 | 0.317953 | 1.45822 | 0.000325 | 0.000516 |
| AC004594.1 | 0.116328 | 0.345157 | 1.569055 | 0.001644 | 0.002274 |
| AC004637.1 | 1.058584 | 0.383446 | -1.46504 | 1.09E-07 | 3.44E-07 |
| AC004691.1 | 0.088151 | 0.464984 | 2.39913 | 0.008361 | 0.00996 |
| AC004765.1 | 0.225847 | 0.78522 | 1.797754 | 0.005433 | 0.006753 |
| AC004825.3 | 0.043074 | 0.237277 | 2.46167 | 0.007572 | 0.009121 |
| AC004832.4 | 0.437556 | 0.876396 | 1.002116 | 0.042986 | 0.043628 |
| AC004832.5 | 0.37554 | 1.167954 | 1.636946 | 1.15E-05 | 2.47E-05 |
| AC004837.2 | 0.498963 | 2.099598 | 2.073109 | 1.46E-08 | 5.47E-08 |
| AC004837.4 | 0.173528 | 0.597646 | 1.784123 | 1.54E-07 | 4.64E-07 |
| AC004893.2 | 0.095323 | 0.301412 | 1.660839 | 4.19E-08 | 1.45E-07 |
| AC004908.2 | 1.166098 | 2.636057 | 1.176693 | 1.01E-08 | 3.92E-08 |
| AC004943.1 | 0.357365 | 0.884017 | 1.306675 | 2.17E-07 | 6.39E-07 |
| AC004943.3 | 0.160677 | 0.32951 | 1.03616 | 0.015995 | 0.017829 |
| AC004967.2 | 0.546402 | 1.492175 | 1.449381 | 6.78E-13 | 6.02E-12 |
| AC005046.1 | 1.243747 | 2.54303 | 1.031856 | 5.05E-05 | 9.47E-05 |
| AC005050.1 | 0.083702 | 0.222269 | 1.408964 | 0.001261 | 0.001773 |
| AC005062.1 | 0.255063 | 0.661536 | 1.374967 | 1.74E-06 | 4.36E-06 |
| AC005072.1 | 0.076509 | 0.433695 | 2.502974 | 0.021345 | 0.023201 |
| AC005096.1 | 0.067695 | 0.711551 | 3.393838 | 7.35E-07 | 1.97E-06 |
| AC005261.1 | 9.528642 | 19.31624 | 1.019472 | 1.01E-13 | 1.04E-12 |
| AC005264.1 | 0.024937 | 0.145499 | 2.544638 | 2.49E-12 | 1.97E-11 |
| AC005277.2 | 1.015405 | 0.412999 | -1.29784 | 1.23E-08 | 4.66E-08 |
| AC005479.1 | 0.30073 | 0.607225 | 1.013762 | 0.000411 | 0.000638 |
| AC005479.2 | 0.232937 | 0.836167 | 1.843851 | 1.01E-07 | 3.21E-07 |
| AC005480.1 | 0.087795 | 0.178981 | 1.027587 | 0.027429 | 0.028925 |
| AC005519.1 | 0.570981 | 1.24403 | 1.123506 | 1.35E-07 | 4.14E-07 |
| AC005540.1 | 0.140074 | 0.45109 | 1.687221 | 1.77E-05 | 3.67E-05 |
| AC005546.1 | 0.430121 | 0.947764 | 1.139786 | 0.033566 | 0.034817 |
| AC005594.1 | 0.062656 | 0.167618 | 1.419657 | 0.00255 | 0.0034 |
| AC005632.6 | 0.276558 | 0.715306 | 1.370979 | 0.008391 | 0.00999 |
| AC005670.1 | 0.070795 | 0.328131 | 2.212547 | 1.13E-05 | 2.43E-05 |
| AC005699.1 | 0.266453 | 0.114981 | -1.21249 | 5.91E-13 | 5.32E-12 |
| AC005726.2 | 0.056484 | 0.240886 | 2.092446 | 1.85E-05 | 3.82E-05 |
| AC005757.1 | 0.078944 | 0.394094 | 2.319635 | 0.005225 | 0.00653 |
| AC005776.2 | 0.248237 | 0.561355 | 1.177194 | 3.73E-08 | 1.31E-07 |
| AC005785.1 | 0.283184 | 0.75205 | 1.409091 | 3.33E-10 | 1.73E-09 |
| AC005920.3 | 0.076758 | 0.189242 | 1.301841 | 9.72E-05 | 0.000172 |
| AC006017.1 | 0.28924 | 0.808866 | 1.483636 | 7.32E-06 | 1.64E-05 |
| AC006042.1 | 4.916002 | 10.90347 | 1.14923 | 1.35E-07 | 4.14E-07 |
| AC006062.1 | 0.014467 | 0.157389 | 3.443454 | 4.11E-12 | 3.13E-11 |
| AC006111.2 | 0.317637 | 1.232484 | 1.956117 | 7.29E-16 | 1.19E-14 |
| AC006160.1 | 0.27927 | 0.628213 | 1.169594 | 7.74E-08 | 2.52E-07 |
| AC006230.1 | 0.413228 | 1.149274 | 1.475713 | 4.42E-14 | 4.92E-13 |
| AC006299.1 | 0.047249 | 0.108531 | 1.199751 | 0.000556 | 0.000844 |
| AC006449.3 | 0.057014 | 0.302636 | 2.408198 | 6.12E-07 | 1.67E-06 |
| AC006460.1 | 0.081335 | 0.452216 | 2.475065 | 0.00055 | 0.000837 |
| AC006460.2 | 0.153349 | 0.347441 | 1.179951 | 0.000322 | 0.000512 |
| AC006511.5 | 0.022433 | 0.114305 | 2.349222 | 0.000266 | 0.000431 |
| AC006525.1 | 0.20826 | 0.479375 | 1.202767 | 3.49E-05 | 6.81E-05 |
| AC006960.3 | 0.111614 | 0.357047 | 1.677598 | 3.56E-05 | 6.90E-05 |
| AC007014.2 | 0.275293 | 0.906263 | 1.718962 | 6.31E-07 | 1.72E-06 |
| AC007038.1 | 1.162926 | 2.504806 | 1.10694 | 4.50E-07 | 1.26E-06 |
| AC007128.1 | 0.194935 | 1.778673 | 3.189737 | 2.64E-19 | 1.13E-17 |
| AC007128.2 | 0.049884 | 0.569004 | 3.511798 | 2.92E-15 | 4.29E-14 |
| AC007216.2 | 0.326626 | 1.036036 | 1.665364 | 1.72E-05 | 3.56E-05 |
| AC007216.3 | 0.314295 | 1.084806 | 1.787245 | 7.26E-12 | 5.35E-11 |
| AC007216.4 | 0.558326 | 1.205595 | 1.110566 | 1.40E-05 | 2.96E-05 |
| AC007285.1 | 0.274079 | 0.941546 | 1.78044 | 3.37E-08 | 1.19E-07 |
| AC007292.2 | 0.27394 | 0.584727 | 1.093906 | 1.62E-09 | 7.30E-09 |
| AC007336.1 | 0.113593 | 0.410604 | 1.853873 | 0.000139 | 0.000238 |
| AC007390.1 | 0.359821 | 0.754155 | 1.067581 | 0.001832 | 0.00251 |
| AC007483.1 | 0.020709 | 0.628331 | 4.923175 | 1.44E-11 | 9.86E-11 |
| AC007497.1 | 0.188928 | 0.71331 | 1.916694 | 3.33E-10 | 1.73E-09 |
| AC007601.1 | 0.029105 | 0.124195 | 2.09329 | 0.040435 | 0.041358 |
| AC007608.1 | 0.101495 | 1.307611 | 3.687448 | 1.42E-07 | 4.30E-07 |
| AC007608.2 | 0.010244 | 1.388258 | 7.082326 | 1.52E-07 | 4.60E-07 |
| AC007608.3 | 0.057119 | 0.173796 | 1.605364 | 1.19E-05 | 2.55E-05 |
| AC007637.1 | 3.232651 | 1.554354 | -1.0564 | 2.98E-16 | 5.42E-15 |
| AC007639.1 | 0.171633 | 0.903698 | 2.396517 | 9.40E-11 | 5.57E-10 |
| AC007684.2 | 0.39853 | 1.207451 | 1.599203 | 0.030795 | 0.032215 |
| AC007728.3 | 0.157584 | 0.613476 | 1.960889 | 6.56E-05 | 0.00012 |
| AC007750.1 | 0.020453 | 0.173362 | 3.083365 | 7.49E-09 | 2.97E-08 |
| AC007785.3 | 0.055072 | 0.239888 | 2.122965 | 5.34E-12 | 4.03E-11 |
| AC007879.3 | 0.096726 | 0.210816 | 1.124015 | 0.022521 | 0.024264 |
| AC007920.2 | 1.093479 | 0.224558 | -2.28377 | 4.27E-09 | 1.80E-08 |
| AC007938.1 | 0.094616 | 0.194708 | 1.041152 | 0.000128 | 0.000221 |
| AC007938.2 | 0.031612 | 0.12745 | 2.011404 | 3.53E-05 | 6.86E-05 |
| AC007938.3 | 0.545498 | 1.494666 | 1.454178 | 1.81E-10 | 9.88E-10 |
| AC008026.3 | 0.072988 | 0.317346 | 2.120318 | 0.000834 | 0.00122 |
| AC008080.4 | 0.018779 | 0.154629 | 3.04161 | 0.022228 | 0.024048 |
| AC008114.1 | 0.112188 | 0.38258 | 1.769839 | 2.41E-05 | 4.86E-05 |
| AC008115.3 | 2.514086 | 5.792075 | 1.204046 | 4.44E-11 | 2.78E-10 |
| AC008119.1 | 0.249947 | 0.690644 | 1.466323 | 8.10E-06 | 1.80E-05 |
| AC008121.2 | 0.200237 | 0.467069 | 1.221924 | 0.002366 | 0.003172 |
| AC008264.2 | 0.042595 | 0.143113 | 1.74839 | 1.40E-08 | 5.30E-08 |
| AC008434.1 | 0.271472 | 0.563063 | 1.052492 | 0.0005 | 0.000767 |
| AC008453.2 | 0.424784 | 0.211299 | -1.00744 | 7.28E-15 | 9.77E-14 |
| AC008456.1 | 0.140558 | 0.983773 | 2.807158 | 1.32E-07 | 4.07E-07 |
| AC008543.1 | 0.149414 | 0.317517 | 1.08752 | 1.96E-07 | 5.82E-07 |
| AC008649.2 | 0.396505 | 2.434736 | 2.618356 | 0.001319 | 0.001849 |
| AC008758.2 | 0.20834 | 0.504567 | 1.276108 | 0.000647 | 0.00097 |
| AC008760.1 | 1.718812 | 3.715124 | 1.111999 | 4.41E-11 | 2.78E-10 |
| AC008764.8 | 0.555149 | 1.115676 | 1.006972 | 8.88E-10 | 4.23E-09 |
| AC008771.1 | 0.172579 | 0.443314 | 1.361072 | 6.27E-16 | 1.06E-14 |
| AC008870.2 | 0.848602 | 2.063638 | 1.282029 | 7.52E-11 | 4.53E-10 |
| AC008883.3 | 0.112047 | 0.330406 | 1.560142 | 3.27E-05 | 6.42E-05 |
| AC008966.3 | 0.079328 | 0.338833 | 2.094674 | 2.71E-07 | 7.85E-07 |
| AC009032.1 | 0.593288 | 3.543194 | 2.578245 | 0.049895 | 0.049895 |
| AC009053.3 | 0.316786 | 0.658365 | 1.055378 | 4.69E-10 | 2.35E-09 |
| AC009065.2 | 4.749342 | 16.31782 | 1.780649 | 6.33E-19 | 2.33E-17 |
| AC009093.1 | 0.111602 | 0.640764 | 2.521425 | 6.03E-13 | 5.40E-12 |
| AC009093.2 | 0.136509 | 0.370138 | 1.439062 | 2.92E-05 | 5.80E-05 |
| AC009120.5 | 0.073886 | 0.238183 | 1.688699 | 6.90E-06 | 1.55E-05 |
| AC009121.2 | 0.252293 | 0.921241 | 1.868478 | 4.57E-13 | 4.24E-12 |
| AC009148.1 | 0.213026 | 0.559465 | 1.39302 | 9.39E-09 | 3.66E-08 |
| AC009262.1 | 0.115353 | 0.631884 | 2.453598 | 1.13E-10 | 6.50E-10 |
| AC009269.5 | 0.409795 | 1.433862 | 1.806931 | 0.000174 | 0.000292 |
| AC009299.2 | 0.102356 | 0.290156 | 1.503237 | 0.000212 | 0.000351 |
| AC009318.4 | 0.488312 | 1.06638 | 1.126847 | 0.032985 | 0.034292 |
| AC009404.1 | 0.810672 | 2.030422 | 1.324589 | 1.35E-14 | 1.64E-13 |
| AC009690.2 | 0.054598 | 0.141777 | 1.376709 | 4.46E-06 | 1.04E-05 |
| AC009704.2 | 0.396635 | 0.937639 | 1.241221 | 0.000301 | 0.000482 |
| AC009948.1 | 0.113305 | 0.262414 | 1.211639 | 0.035046 | 0.036311 |
| AC009948.2 | 0.202242 | 0.422844 | 1.064045 | 0.017351 | 0.019213 |
| AC009975.1 | 0.035533 | 0.914039 | 4.685042 | 4.85E-08 | 1.65E-07 |
| AC009975.2 | 0.001233 | 0.235999 | 7.580982 | 1.18E-08 | 4.52E-08 |
| AC009996.1 | 0.419912 | 1.397352 | 1.734538 | 1.81E-08 | 6.65E-08 |
| AC010148.1 | 0.092953 | 0.333299 | 1.842235 | 5.38E-12 | 4.05E-11 |
| AC010168.1 | 0.21064 | 0.652496 | 1.631193 | 0.001523 | 0.002112 |
| AC010184.1 | 0.055949 | 0.194151 | 1.794997 | 0.00031 | 0.000494 |
| AC010285.3 | 0.151263 | 0.332607 | 1.13676 | 8.99E-05 | 0.00016 |
| AC010320.3 | 0.26497 | 0.805567 | 1.604176 | 0.000753 | 0.001114 |
| AC010343.3 | 0.11327 | 0.251624 | 1.151509 | 0.042129 | 0.042923 |
| AC010359.3 | 0.069293 | 0.664288 | 3.261028 | 0.000804 | 0.001182 |
| AC010422.4 | 0.46844 | 1.054767 | 1.17099 | 0.001349 | 0.001881 |
| AC010463.3 | 0.663193 | 1.381129 | 1.058347 | 2.35E-07 | 6.89E-07 |
| AC010491.2 | 0.143451 | 0.429237 | 1.581215 | 0.025109 | 0.026755 |
| AC010524.1 | 0.13006 | 0.52383 | 2.009915 | 0.030484 | 0.031908 |
| AC010536.2 | 0.961063 | 2.317006 | 1.269559 | 0.006004 | 0.007397 |
| AC010538.1 | 0.512435 | 1.191877 | 1.217795 | 3.60E-15 | 5.13E-14 |
| AC010542.5 | 1.804272 | 4.947409 | 1.455256 | 1.39E-13 | 1.40E-12 |
| AC010632.1 | 0.066602 | 0.197469 | 1.567983 | 0.018018 | 0.019904 |
| AC010680.2 | 0.043586 | 0.142011 | 1.704068 | 0.025836 | 0.027434 |
| AC010727.1 | 0.031623 | 0.399959 | 3.660796 | 3.03E-09 | 1.30E-08 |
| AC010761.1 | 1.690744 | 4.823528 | 1.51243 | 5.09E-17 | 1.08E-15 |
| AC010834.3 | 1.450698 | 3.546262 | 1.289552 | 4.82E-07 | 1.34E-06 |
| AC010976.1 | 0.374847 | 0.905844 | 1.272963 | 4.73E-08 | 1.62E-07 |
| AC010999.2 | 0.120856 | 0.299249 | 1.308059 | 0.00423 | 0.005366 |
| AC011247.1 | 0.459616 | 1.272838 | 1.469547 | 0.016671 | 0.018527 |
| AC011365.1 | 0.2737 | 0.096626 | -1.50211 | 1.95E-14 | 2.33E-13 |
| AC011442.1 | 0.344656 | 0.82526 | 1.259693 | 0.027358 | 0.028867 |
| AC011466.3 | 0.120884 | 0.415089 | 1.779802 | 1.21E-08 | 4.62E-08 |
| AC011472.4 | 2.587142 | 1.20295 | -1.10478 | 4.01E-08 | 1.40E-07 |
| AC011603.2 | 0.121049 | 0.329011 | 1.442545 | 4.86E-13 | 4.47E-12 |
| AC011676.1 | 0.328016 | 1.96464 | 2.582425 | 5.32E-16 | 9.22E-15 |
| AC011700.1 | 0.085688 | 0.431669 | 2.332755 | 8.76E-13 | 7.63E-12 |
| AC011773.1 | 0.100867 | 0.50442 | 2.322165 | 5.01E-07 | 1.39E-06 |
| AC011773.4 | 0.063995 | 0.217916 | 1.767731 | 4.61E-06 | 1.07E-05 |
| AC011815.2 | 0.120753 | 0.277766 | 1.201807 | 0.016006 | 0.017831 |
| AC011997.1 | 0.067481 | 0.173987 | 1.366419 | 0.020223 | 0.022096 |
| AC012020.1 | 0.116442 | 0.321398 | 1.464749 | 3.80E-06 | 8.97E-06 |
| AC012065.2 | 0.525179 | 1.356584 | 1.369097 | 6.86E-05 | 0.000125 |
| AC012085.2 | 4.651588 | 0.56876 | -3.03183 | 1.04E-14 | 1.32E-13 |
| AC012184.3 | 0.418784 | 0.96367 | 1.202333 | 1.84E-10 | 1.00E-09 |
| AC012213.3 | 0.197119 | 0.792132 | 2.006677 | 9.37E-10 | 4.41E-09 |
| AC012360.1 | 0.1308 | 0.380954 | 1.542253 | 2.98E-06 | 7.17E-06 |
| AC012443.2 | 0.313607 | 0.801615 | 1.353952 | 1.48E-10 | 8.21E-10 |
| AC012459.1 | 0.400123 | 0.80798 | 1.013875 | 0.03089 | 0.032296 |
| AC012494.1 | 0.015491 | 0.419149 | 4.75799 | 1.65E-08 | 6.12E-08 |
| AC012615.4 | 0.342121 | 1.055253 | 1.625011 | 2.78E-06 | 6.75E-06 |
| AC012640.1 | 0.079979 | 0.320206 | 2.001304 | 2.65E-08 | 9.51E-08 |
| AC012676.4 | 0.25144 | 0.704105 | 1.48558 | 1.95E-06 | 4.82E-06 |
| AC013400.1 | 0.078214 | 0.266042 | 1.766158 | 2.87E-05 | 5.72E-05 |
| AC013452.2 | 0.095779 | 0.283274 | 1.564418 | 2.27E-05 | 4.59E-05 |
| AC013652.1 | 0.152493 | 0.531151 | 1.800378 | 2.31E-07 | 6.79E-07 |
| AC013731.1 | 0.366591 | 0.958352 | 1.386385 | 1.41E-10 | 7.81E-10 |
| AC015813.1 | 2.615979 | 7.402631 | 1.500687 | 3.74E-11 | 2.39E-10 |
| AC015849.3 | 3.03926 | 6.492458 | 1.095044 | 3.55E-06 | 8.44E-06 |
| AC015853.1 | 0.087784 | 0.373362 | 2.088548 | 3.37E-05 | 6.59E-05 |
| AC015871.6 | 0.130442 | 0.55424 | 2.087104 | 6.26E-05 | 0.000115 |
| AC015908.4 | 0.342353 | 0.101235 | -1.75778 | 2.34E-22 | 2.87E-20 |
| AC015921.1 | 0.017716 | 0.10751 | 2.601318 | 0.00322 | 0.004216 |
| AC015922.3 | 17.11733 | 7.033735 | -1.2831 | 2.04E-17 | 4.87E-16 |
| AC015983.2 | 0.011263 | 0.167894 | 3.897914 | 0.00024 | 0.000392 |
| AC015987.1 | 0.164547 | 0.487056 | 1.565591 | 4.10E-05 | 7.83E-05 |
| AC016394.1 | 0.667307 | 2.130694 | 1.674901 | 2.45E-16 | 4.55E-15 |
| AC016405.1 | 0.112463 | 0.660672 | 2.554487 | 0.017485 | 0.019349 |
| AC016597.1 | 0.055777 | 0.209377 | 1.908368 | 9.11E-05 | 0.000162 |
| AC016722.2 | 0.21747 | 0.509725 | 1.228904 | 2.90E-06 | 7.00E-06 |
| AC016727.3 | 0.220447 | 0.777804 | 1.818979 | 0.00094 | 0.001358 |
| AC016737.1 | 0.462837 | 1.57925 | 1.770663 | 7.76E-13 | 6.82E-12 |
| AC016745.1 | 0.096674 | 0.662508 | 2.776732 | 3.71E-11 | 2.37E-10 |
| AC016831.1 | 0.343381 | 1.219447 | 1.828344 | 2.48E-21 | 2.07E-19 |
| AC016831.4 | 0.261605 | 1.545617 | 2.562723 | 0.000409 | 0.000636 |
| AC016831.6 | 0.094284 | 0.406742 | 2.109035 | 9.90E-19 | 3.43E-17 |
| AC016949.1 | 0.399198 | 1.240672 | 1.635947 | 0.00101 | 0.001446 |
| AC017071.1 | 0.299358 | 0.94503 | 1.658488 | 0.04608 | 0.046409 |
| AC017076.1 | 0.130765 | 0.317919 | 1.281683 | 0.005092 | 0.00638 |
| AC017083.1 | 0.199972 | 0.687059 | 1.780636 | 9.43E-14 | 9.84E-13 |
| AC017116.2 | 0.15233 | 0.310398 | 1.026916 | 0.015833 | 0.017691 |
| AC018410.1 | 0.391105 | 1.026998 | 1.392807 | 0.00079 | 0.001164 |
| AC018552.3 | 0.086947 | 0.582376 | 2.74375 | 0.000165 | 0.000278 |
| AC018557.2 | 0.067516 | 0.498786 | 2.885115 | 8.55E-06 | 1.89E-05 |
| AC018628.2 | 0.139884 | 0.959238 | 2.77766 | 0.005147 | 0.006436 |
| AC018648.1 | 0.337305 | 0.705819 | 1.065245 | 0.002191 | 0.002953 |
| AC018695.3 | 0.101919 | 0.319715 | 1.649367 | 5.43E-05 | 0.000101 |
| AC018695.4 | 0.664151 | 1.674703 | 1.334322 | 1.70E-08 | 6.28E-08 |
| AC018714.2 | 0.080942 | 0.819409 | 3.339627 | 6.29E-11 | 3.88E-10 |
| AC018766.1 | 0.279086 | 0.578393 | 1.05134 | 0.015726 | 0.017594 |
| AC018809.1 | 0.712126 | 1.677414 | 1.236035 | 1.72E-06 | 4.32E-06 |
| AC018926.2 | 0.575395 | 1.519866 | 1.401318 | 6.21E-09 | 2.50E-08 |
| AC019118.1 | 0.06073 | 0.311184 | 2.357282 | 3.50E-05 | 6.81E-05 |
| AC019186.1 | 0.214005 | 0.493818 | 1.206338 | 0.000277 | 0.000447 |
| AC020612.3 | 0.196672 | 0.640322 | 1.703004 | 0.001145 | 0.001624 |
| AC020658.4 | 0.167886 | 0.491482 | 1.549657 | 5.02E-06 | 1.16E-05 |
| AC020663.3 | 0.289009 | 0.737657 | 1.351835 | 1.34E-06 | 3.45E-06 |
| AC020763.1 | 0.11546 | 0.316814 | 1.456237 | 1.27E-05 | 2.70E-05 |
| AC020915.1 | 0.786305 | 2.00843 | 1.352908 | 6.36E-06 | 1.44E-05 |
| AC020978.1 | 0.16567 | 0.473411 | 1.514784 | 0.048342 | 0.0485 |
| AC020978.2 | 0.122426 | 0.275593 | 1.170633 | 0.026942 | 0.028477 |
| AC020978.3 | 0.537051 | 1.46579 | 1.448546 | 0.000231 | 0.000379 |
| AC020978.7 | 0.161349 | 0.340208 | 1.076233 | 3.94E-05 | 7.55E-05 |
| AC021321.1 | 0.359826 | 0.751149 | 1.061802 | 0.000116 | 0.000201 |
| AC021851.1 | 0.450495 | 1.119356 | 1.313085 | 3.94E-14 | 4.44E-13 |
| AC022079.1 | 0.147188 | 0.577198 | 1.971403 | 0.010172 | 0.011849 |
| AC022137.3 | 0.404265 | 1.147876 | 1.505593 | 9.20E-08 | 2.94E-07 |
| AC022165.1 | 0.073886 | 0.300448 | 2.023743 | 0.000193 | 0.000321 |
| AC022210.1 | 1.084465 | 2.344883 | 1.112532 | 2.30E-12 | 1.84E-11 |
| AC022733.1 | 0.561977 | 0.158266 | -1.82816 | 3.68E-09 | 1.56E-08 |
| AC022784.6 | 0.06723 | 0.560288 | 3.058987 | 5.91E-09 | 2.39E-08 |
| AC022819.1 | 0.065681 | 0.144778 | 1.140283 | 0.000547 | 0.000832 |
| AC022973.5 | 0.296981 | 0.777052 | 1.387639 | 3.69E-05 | 7.13E-05 |
| AC023202.1 | 0.239365 | 0.110496 | -1.11522 | 1.59E-07 | 4.78E-07 |
| AC023355.1 | 0.108972 | 0.229056 | 1.071744 | 7.48E-05 | 0.000136 |
| AC023424.2 | 0.149549 | 0.805247 | 2.428815 | 0.001799 | 0.002471 |
| AC023510.2 | 0.675553 | 0.293116 | -1.2046 | 2.72E-12 | 2.15E-11 |
| AC023794.1 | 0.587593 | 0.233781 | -1.32966 | 1.15E-13 | 1.18E-12 |
| AC023825.2 | 0.153753 | 0.814551 | 2.405387 | 1.39E-08 | 5.25E-08 |
| AC023830.1 | 0.091553 | 0.246179 | 1.427019 | 0.001066 | 0.001518 |
| AC024060.2 | 5.561042 | 11.15485 | 1.004244 | 5.49E-11 | 3.42E-10 |
| AC024361.3 | 0.241507 | 0.69123 | 1.5171 | 4.32E-05 | 8.20E-05 |
| AC024382.1 | 0.094916 | 0.231392 | 1.285614 | 0.035195 | 0.036425 |
| AC024451.4 | 0.173426 | 0.541574 | 1.642843 | 0.0011 | 0.001563 |
| AC024560.3 | 0.619779 | 1.562591 | 1.334114 | 1.52E-05 | 3.18E-05 |
| AC024560.4 | 0.787521 | 1.889348 | 1.262498 | 4.81E-09 | 2.01E-08 |
| AC024588.1 | 0.10447 | 0.305694 | 1.549005 | 6.83E-08 | 2.25E-07 |
| AC024940.5 | 0.232912 | 0.467845 | 1.006247 | 0.001892 | 0.002584 |
| AC025031.4 | 0.250423 | 0.619927 | 1.30773 | 1.15E-07 | 3.60E-07 |
| AC025162.2 | 0.209642 | 0.429015 | 1.033103 | 0.002488 | 0.003321 |
| AC025171.4 | 0.511779 | 1.031274 | 1.010835 | 0.000225 | 0.000371 |
| AC026254.2 | 0.2341 | 0.529171 | 1.17661 | 1.56E-05 | 3.26E-05 |
| AC026356.1 | 0.620274 | 2.49249 | 2.006609 | 2.38E-16 | 4.52E-15 |
| AC026356.2 | 0.235758 | 0.923819 | 1.970303 | 2.40E-14 | 2.79E-13 |
| AC026368.1 | 0.362907 | 1.850006 | 2.349858 | 1.12E-14 | 1.40E-13 |
| AC026401.2 | 0.074872 | 0.367778 | 2.296336 | 0.00317 | 0.004154 |
| AC026470.2 | 0.45664 | 1.267728 | 1.473118 | 0.004142 | 0.005266 |
| AC026979.1 | 0.053384 | 0.221839 | 2.055041 | 7.25E-05 | 0.000132 |
| AC027277.2 | 0.229605 | 0.754909 | 1.717151 | 0.008735 | 0.010352 |
| AC027279.1 | 0.087679 | 0.378363 | 2.109465 | 0.022421 | 0.024214 |
| AC027373.1 | 0.392935 | 0.892321 | 1.183273 | 4.95E-09 | 2.05E-08 |
| AC027644.3 | 0.1262 | 0.296508 | 1.232359 | 0.010136 | 0.011814 |
| AC027682.4 | 0.201063 | 0.47315 | 1.234651 | 2.11E-05 | 4.30E-05 |
| AC027801.1 | 0.48503 | 1.092499 | 1.171486 | 1.41E-06 | 3.59E-06 |
| AC027808.2 | 0.068342 | 0.526989 | 2.94693 | 4.51E-05 | 8.52E-05 |
| AC027811.1 | 0.071809 | 0.184429 | 1.360826 | 0.002679 | 0.003559 |
| AC034139.1 | 1.492307 | 0.42641 | -1.80723 | 3.15E-22 | 3.61E-20 |
| AC034199.1 | 0.057533 | 0.208802 | 1.859686 | 4.64E-05 | 8.75E-05 |
| AC034229.4 | 0.391198 | 1.023953 | 1.388179 | 0.000474 | 0.000731 |
| AC036108.3 | 3.446374 | 0.556428 | -2.63081 | 6.80E-15 | 9.19E-14 |
| AC037459.4 | 0.445881 | 1.048537 | 1.233646 | 2.03E-05 | 4.16E-05 |
| AC037487.1 | 0.089823 | 0.295074 | 1.715916 | 0.000457 | 0.000707 |
| AC040169.3 | 0.65553 | 1.438219 | 1.133549 | 8.63E-08 | 2.78E-07 |
| AC040934.1 | 0.248109 | 0.703457 | 1.503487 | 0.000234 | 0.000384 |
| AC048341.2 | 1.752212 | 4.335849 | 1.307138 | 7.48E-11 | 4.52E-10 |
| AC048344.4 | 0.304853 | 1.628849 | 2.417665 | 1.31E-15 | 2.02E-14 |
| AC048382.1 | 0.087272 | 0.2509 | 1.523518 | 0.000206 | 0.000342 |
| AC055811.1 | 0.127479 | 0.398214 | 1.643283 | 0.002226 | 0.002998 |
| AC058791.1 | 0.285447 | 1.765442 | 2.628737 | 4.85E-06 | 1.12E-05 |
| AC060780.1 | 1.823284 | 4.473212 | 1.294772 | 8.50E-15 | 1.11E-13 |
| AC061975.7 | 0.114065 | 0.272325 | 1.255469 | 0.001769 | 0.002434 |
| AC061975.8 | 0.163742 | 0.364357 | 1.153928 | 3.97E-05 | 7.60E-05 |
| AC064836.1 | 0.07374 | 0.348074 | 2.238883 | 1.82E-05 | 3.76E-05 |
| AC064836.3 | 0.784656 | 3.134445 | 1.998078 | 1.02E-17 | 2.80E-16 |
| AC068189.1 | 1.314344 | 0.267076 | -2.29902 | 4.85E-15 | 6.75E-14 |
| AC068205.2 | 0.424437 | 1.191548 | 1.489214 | 7.32E-14 | 7.98E-13 |
| AC068282.1 | 0.085047 | 0.695726 | 3.032196 | 0.027892 | 0.029379 |
| AC068724.1 | 0.054526 | 0.231173 | 2.083965 | 0.000193 | 0.000322 |
| AC068790.2 | 0.446147 | 1.080551 | 1.276177 | 0.02049 | 0.022338 |
| AC068790.3 | 0.5639 | 1.342205 | 1.251094 | 0.000279 | 0.00045 |
| AC068790.4 | 0.314095 | 1.124457 | 1.839955 | 0.001428 | 0.001987 |
| AC068790.5 | 0.596026 | 1.503425 | 1.334807 | 0.000208 | 0.000345 |
| AC068790.6 | 0.308851 | 1.079541 | 1.805435 | 0.010603 | 0.012281 |
| AC069222.1 | 0.082049 | 0.409822 | 2.320442 | 3.50E-14 | 4.00E-13 |
| AC073046.3 | 0.443581 | 1.226669 | 1.467476 | 9.48E-05 | 0.000168 |
| AC073140.2 | 0.094458 | 0.553778 | 2.55156 | 2.00E-14 | 2.37E-13 |
| AC073288.2 | 0.123723 | 0.346477 | 1.485641 | 0.000315 | 0.000502 |
| AC073326.1 | 0.120107 | 0.58585 | 2.286212 | 8.83E-05 | 0.000158 |
| AC073333.1 | 0.41223 | 0.92258 | 1.162223 | 0.000397 | 0.00062 |
| AC073349.4 | 0.535709 | 1.299351 | 1.278269 | 0.012497 | 0.014248 |
| AC073487.1 | 0.822588 | 1.733816 | 1.075709 | 8.50E-06 | 1.88E-05 |
| AC073517.1 | 0.188621 | 0.397528 | 1.075567 | 0.026125 | 0.027692 |
| AC073529.1 | 0.190679 | 0.56294 | 1.561835 | 5.94E-15 | 8.08E-14 |
| AC073592.1 | 0.105723 | 0.536978 | 2.344571 | 7.62E-06 | 1.70E-05 |
| AC073593.2 | 0.066177 | 0.214964 | 1.699697 | 8.89E-07 | 2.34E-06 |
| AC073957.3 | 1.942814 | 4.377635 | 1.172004 | 7.83E-08 | 2.54E-07 |
| AC074044.1 | 0.116481 | 0.346797 | 1.573993 | 3.94E-06 | 9.26E-06 |
| AC074050.4 | 0.25633 | 0.615453 | 1.263646 | 4.06E-06 | 9.52E-06 |
| AC074117.1 | 2.75956 | 6.295086 | 1.189788 | 2.27E-16 | 4.38E-15 |
| AC078778.1 | 0.3138 | 0.819774 | 1.38538 | 0.0065 | 0.00795 |
| AC078795.1 | 0.246051 | 0.595583 | 1.275344 | 4.60E-07 | 1.29E-06 |
| AC078820.2 | 0.074258 | 0.196646 | 1.404983 | 8.28E-05 | 0.000148 |
| AC078850.1 | 0.075653 | 0.183067 | 1.274891 | 0.005006 | 0.006281 |
| AC078852.1 | 0.099021 | 0.488215 | 2.301711 | 0.00197 | 0.002682 |
| AC078860.2 | 0.019807 | 0.19277 | 3.282798 | 3.18E-10 | 1.66E-09 |
| AC078860.3 | 0.135105 | 1.338125 | 3.308064 | 9.03E-12 | 6.61E-11 |
| AC078883.2 | 0.308281 | 0.821223 | 1.413526 | 0.001069 | 0.00152 |
| AC078962.1 | 0.295626 | 1.167467 | 1.981539 | 0.024904 | 0.026567 |
| AC078983.1 | 0.034484 | 0.167099 | 2.276714 | 2.89E-06 | 7.00E-06 |
| AC079160.1 | 0.127877 | 0.312454 | 1.288889 | 0.001513 | 0.002099 |
| AC079313.2 | 1.930447 | 0.204279 | -3.24032 | 2.34E-09 | 1.04E-08 |
| AC079684.1 | 1.002942 | 2.913889 | 1.538708 | 3.24E-11 | 2.08E-10 |
| AC079753.2 | 0.050028 | 0.177344 | 1.825745 | 4.91E-08 | 1.66E-07 |
| AC079766.1 | 0.350165 | 0.710463 | 1.020723 | 0.000651 | 0.000975 |
| AC079804.3 | 0.354358 | 1.169434 | 1.72253 | 4.20E-07 | 1.18E-06 |
| AC083805.1 | 0.049309 | 0.137831 | 1.482966 | 2.43E-05 | 4.89E-05 |
| AC083805.2 | 0.331374 | 1.067098 | 1.687159 | 0.000276 | 0.000446 |
| AC083806.2 | 0.097612 | 0.269195 | 1.463527 | 0.000199 | 0.00033 |
| AC083900.1 | 3.106635 | 1.228322 | -1.33866 | 8.21E-14 | 8.77E-13 |
| AC083906.3 | 0.0288 | 0.337788 | 3.551979 | 4.08E-06 | 9.56E-06 |
| AC084117.1 | 0.892537 | 2.189804 | 1.294817 | 2.50E-05 | 5.02E-05 |
| AC084876.1 | 0.097812 | 0.270622 | 1.468202 | 0.000879 | 0.001281 |
| AC087175.1 | 0.115167 | 0.322718 | 1.48654 | 3.18E-09 | 1.36E-08 |
| AC087222.1 | 0.39176 | 1.668319 | 2.090351 | 5.19E-08 | 1.75E-07 |
| AC087277.2 | 4.930426 | 2.150792 | -1.19684 | 8.21E-13 | 7.18E-12 |
| AC087289.1 | 0.066547 | 0.199758 | 1.58582 | 0.000759 | 0.001123 |
| AC087379.2 | 0.95426 | 0.095676 | -3.31815 | 4.54E-22 | 4.91E-20 |
| AC087392.4 | 0.303763 | 0.632801 | 1.058806 | 0.002371 | 0.003177 |
| AC087521.2 | 0.244095 | 0.599727 | 1.296861 | 0.000411 | 0.000638 |
| AC087683.2 | 0.330242 | 0.870555 | 1.398412 | 0.032599 | 0.033949 |
| AC090004.2 | 1.296833 | 0.259652 | -2.32034 | 6.49E-21 | 4.42E-19 |
| AC090018.2 | 0.224914 | 0.576565 | 1.35811 | 1.05E-11 | 7.62E-11 |
| AC090116.1 | 0.219816 | 3.263441 | 3.892024 | 3.83E-12 | 2.93E-11 |
| AC090197.1 | 0.078605 | 0.262854 | 1.741576 | 9.19E-06 | 2.02E-05 |
| AC090527.3 | 0.164002 | 0.423406 | 1.368324 | 0.001854 | 0.002535 |
| AC090578.2 | 0.15086 | 0.676796 | 2.165507 | 0.002169 | 0.002928 |
| AC090578.3 | 0.054858 | 0.307434 | 2.486497 | 0.000123 | 0.000212 |
| AC090739.1 | 1.268242 | 3.284736 | 1.372947 | 2.81E-05 | 5.63E-05 |
| AC090825.1 | 0.365356 | 0.154894 | -1.23802 | 1.82E-13 | 1.78E-12 |
| AC090945.1 | 0.097556 | 0.543297 | 2.477441 | 2.63E-06 | 6.41E-06 |
| AC091181.2 | 0.107823 | 0.346571 | 1.684483 | 2.99E-07 | 8.63E-07 |
| AC091729.2 | 0.110298 | 0.375693 | 1.768151 | 0.002598 | 0.003458 |
| AC092119.2 | 1.470081 | 3.294546 | 1.164184 | 1.53E-09 | 6.91E-09 |
| AC092127.1 | 0.027753 | 0.273525 | 3.300933 | 0.001047 | 0.001494 |
| AC092168.2 | 0.242977 | 1.907841 | 2.973051 | 2.98E-07 | 8.61E-07 |
| AC092171.1 | 0.280386 | 1.075648 | 1.93972 | 1.19E-11 | 8.50E-11 |
| AC092301.1 | 0.400867 | 0.953772 | 1.250518 | 2.01E-05 | 4.11E-05 |
| AC092338.1 | 0.410395 | 1.625448 | 1.985751 | 0.003802 | 0.004877 |
| AC092338.3 | 0.611002 | 1.836227 | 1.587495 | 4.91E-15 | 6.78E-14 |
| AC092428.1 | 0.163623 | 0.421387 | 1.364767 | 0.044313 | 0.044874 |
| AC092645.2 | 0.424126 | 1.057412 | 1.317974 | 5.51E-08 | 1.85E-07 |
| AC092652.1 | 1.563626 | 0.125347 | -3.6409 | 3.67E-26 | 2.25E-23 |
| AC092681.2 | 0.071707 | 0.302654 | 2.077483 | 7.94E-05 | 0.000143 |
| AC092718.1 | 0.044777 | 0.159761 | 1.835096 | 0.000573 | 0.000869 |
| AC092718.5 | 0.336088 | 0.877467 | 1.384505 | 1.41E-09 | 6.45E-09 |
| AC092718.8 | 0.068514 | 0.170254 | 1.313221 | 6.98E-10 | 3.38E-09 |
| AC092755.2 | 0.190812 | 0.586614 | 1.620263 | 0.00811 | 0.009693 |
| AC092828.1 | 0.110891 | 0.6777 | 2.611508 | 0.003582 | 0.004628 |
| AC092910.3 | 0.928426 | 2.203407 | 1.246878 | 1.36E-13 | 1.37E-12 |
| AC093382.1 | 0.173923 | 0.352014 | 1.017182 | 0.007001 | 0.008512 |
| AC093424.1 | 0.300042 | 0.643173 | 1.100043 | 0.025866 | 0.02745 |
| AC093520.1 | 0.054323 | 0.194937 | 1.843367 | 6.13E-07 | 1.67E-06 |
| AC093591.2 | 0.043207 | 0.143256 | 1.729258 | 0.000604 | 0.000908 |
| AC093675.2 | 0.090021 | 0.381389 | 2.08293 | 0.015668 | 0.01754 |
| AC093690.1 | 0.145719 | 0.381627 | 1.388979 | 0.007218 | 0.008741 |
| AC093732.1 | 0.132728 | 1.690322 | 3.670754 | 1.51E-13 | 1.50E-12 |
| AC093752.2 | 0.132419 | 0.32706 | 1.304451 | 0.005416 | 0.006741 |
| AC093788.1 | 0.816507 | 2.112837 | 1.371644 | 1.05E-08 | 4.04E-08 |
| AC096642.1 | 0.209988 | 0.476393 | 1.181844 | 0.000106 | 0.000186 |
| AC097500.1 | 0.519923 | 1.390512 | 1.419245 | 0.006665 | 0.008135 |
| AC097532.2 | 0.048781 | 0.127374 | 1.384668 | 0.001318 | 0.001848 |
| AC098656.1 | 2.566819 | 1.251147 | -1.03673 | 1.85E-11 | 1.24E-10 |
| AC099066.2 | 0.073544 | 0.208709 | 1.50481 | 0.000246 | 0.000401 |
| AC099314.1 | 0.086958 | 0.208658 | 1.26275 | 0.001029 | 0.00147 |
| AC099518.4 | 0.085244 | 0.195751 | 1.199348 | 0.001306 | 0.001832 |
| AC099565.1 | 0.423156 | 1.058081 | 1.322189 | 0.000951 | 0.001372 |
| AC099792.1 | 0.04563 | 0.757828 | 4.053808 | 2.53E-16 | 4.64E-15 |
| AC099811.1 | 0.20226 | 0.628812 | 1.636414 | 0.000159 | 0.000269 |
| AC100821.2 | 0.253114 | 0.599431 | 1.243808 | 0.001157 | 0.00164 |
| AC103591.3 | 1.378807 | 4.674407 | 1.761363 | 6.54E-07 | 1.77E-06 |
| AC103703.1 | 0.119426 | 0.477236 | 1.99859 | 0.004428 | 0.005598 |
| AC103718.1 | 0.01527 | 0.118977 | 2.961938 | 6.88E-14 | 7.62E-13 |
| AC103739.1 | 0.352747 | 0.908547 | 1.364929 | 0.022985 | 0.024692 |
| AC103739.3 | 0.104626 | 0.272409 | 1.380536 | 0.001258 | 0.001771 |
| AC103740.1 | 1.041549 | 0.305426 | -1.76984 | 3.99E-17 | 8.73E-16 |
| AC103923.1 | 0.954212 | 0.391345 | -1.28587 | 7.03E-18 | 2.02E-16 |
| AC104088.1 | 0.03537 | 0.146803 | 2.05329 | 2.36E-07 | 6.90E-07 |
| AC104109.4 | 0.103353 | 0.239127 | 1.210192 | 0.002622 | 0.003487 |
| AC104211.1 | 0.382386 | 0.084332 | -2.18087 | 3.77E-20 | 1.98E-18 |
| AC104335.1 | 0.292035 | 0.670443 | 1.198974 | 0.002322 | 0.00312 |
| AC104365.1 | 0.14274 | 1.155782 | 3.017412 | 8.34E-06 | 1.84E-05 |
| AC104370.1 | 0.029209 | 0.62481 | 4.418918 | 1.20E-12 | 1.03E-11 |
| AC104472.1 | 0.104926 | 0.215864 | 1.040755 | 7.58E-09 | 2.99E-08 |
| AC104472.5 | 0.162358 | 0.53346 | 1.716201 | 0.013608 | 0.015383 |
| AC104590.1 | 0.069237 | 0.176513 | 1.350153 | 5.81E-05 | 0.000108 |
| AC104695.3 | 1.122286 | 4.630643 | 2.044772 | 1.12E-05 | 2.43E-05 |
| AC104785.1 | 0.490035 | 1.444924 | 1.560037 | 2.26E-12 | 1.82E-11 |
| AC104791.1 | 0.052535 | 0.374944 | 2.835329 | 1.43E-06 | 3.63E-06 |
| AC104841.1 | 0.498649 | 1.517417 | 1.605521 | 0.000157 | 0.000266 |
| AC104964.3 | 0.449723 | 1.096125 | 1.285303 | 0.006418 | 0.007855 |
| AC104984.1 | 0.099537 | 0.245005 | 1.299502 | 0.013316 | 0.015099 |
| AC105101.2 | 0.503114 | 0.184307 | -1.44877 | 5.09E-17 | 1.08E-15 |
| AC105137.2 | 0.329295 | 0.90728 | 1.462166 | 3.01E-12 | 2.36E-11 |
| AC105339.2 | 0.082077 | 0.25679 | 1.645545 | 1.06E-11 | 7.67E-11 |
| AC105429.1 | 0.136767 | 0.781886 | 2.515233 | 1.27E-17 | 3.19E-16 |
| AC106791.1 | 0.372044 | 0.120951 | -1.62105 | 1.94E-17 | 4.68E-16 |
| AC106820.3 | 0.685537 | 1.804597 | 1.39637 | 6.32E-10 | 3.11E-09 |
| AC106881.1 | 0.4095 | 0.154915 | -1.40239 | 1.05E-17 | 2.83E-16 |
| AC107032.2 | 0.369758 | 0.907454 | 1.295242 | 8.96E-08 | 2.87E-07 |
| AC107032.3 | 0.034965 | 0.117634 | 1.750312 | 0.00029 | 0.000463 |
| AC107050.1 | 0.153707 | 1.063041 | 2.789943 | 0.014369 | 0.016164 |
| AC107081.1 | 0.200347 | 0.732703 | 1.870731 | 3.22E-12 | 2.50E-11 |
| AC107081.3 | 0.403395 | 1.079055 | 1.419502 | 7.07E-12 | 5.24E-11 |
| AC107308.1 | 0.223465 | 0.833197 | 1.898608 | 3.71E-05 | 7.14E-05 |
| AC107464.1 | 0.033509 | 0.258873 | 2.949611 | 0.000153 | 0.000261 |
| AC107959.1 | 0.929167 | 0.440651 | -1.0763 | 1.51E-11 | 1.03E-10 |
| AC108058.1 | 0.872291 | 1.859983 | 1.092408 | 0.005106 | 0.006393 |
| AC108463.2 | 0.142681 | 0.611434 | 2.0994 | 1.04E-06 | 2.71E-06 |
| AC108463.3 | 0.054879 | 0.421635 | 2.941667 | 7.78E-09 | 3.06E-08 |
| AC108681.1 | 0.023667 | 0.322331 | 3.767568 | 1.16E-10 | 6.62E-10 |
| AC108727.1 | 0.4776 | 1.428583 | 1.58071 | 0.014192 | 0.016005 |
| AC109992.2 | 0.091535 | 0.230804 | 1.334272 | 0.016069 | 0.017879 |
| AC110769.2 | 0.213365 | 0.506496 | 1.247226 | 2.91E-05 | 5.79E-05 |
| AC110813.1 | 1.381716 | 0.457443 | -1.5948 | 1.08E-15 | 1.68E-14 |
| AC112496.1 | 0.612821 | 2.588437 | 2.078544 | 0.000101 | 0.000177 |
| AC112512.1 | 0.052151 | 0.369067 | 2.823112 | 0.021059 | 0.022914 |
| AC112721.2 | 0.187656 | 0.681663 | 1.86097 | 6.31E-08 | 2.09E-07 |
| AC113139.1 | 0.460828 | 1.272486 | 1.46535 | 5.89E-05 | 0.000109 |
| AC113143.2 | 0.552744 | 0.222078 | -1.31554 | 1.33E-12 | 1.13E-11 |
| AC113361.1 | 0.125581 | 0.32346 | 1.364963 | 0.007476 | 0.009018 |
| AC114763.1 | 0.419544 | 0.942976 | 1.168399 | 5.52E-08 | 1.85E-07 |
| AC114811.2 | 0.084007 | 0.352648 | 2.069647 | 4.12E-10 | 2.09E-09 |
| AC115282.2 | 0.031565 | 0.122736 | 1.959157 | 7.81E-05 | 0.000141 |
| AC115284.4 | 0.087765 | 0.267551 | 1.608096 | 1.86E-09 | 8.33E-09 |
| AC116025.1 | 0.067991 | 0.355732 | 2.387382 | 0.000673 | 0.001007 |
| AC116903.2 | 0.029179 | 0.212111 | 2.861811 | 0.008843 | 0.010467 |
| AC118344.1 | 0.324019 | 0.746704 | 1.204459 | 0.001791 | 0.002463 |
| AC120036.4 | 0.307665 | 0.13545 | -1.1836 | 6.88E-11 | 4.20E-10 |
| AC120042.2 | 0.100312 | 0.53168 | 2.406068 | 5.22E-06 | 1.20E-05 |
| AC122719.3 | 0.048753 | 0.234268 | 2.264585 | 2.26E-12 | 1.82E-11 |
| AC123023.1 | 0.450891 | 5.98291 | 3.729998 | 2.28E-19 | 9.97E-18 |
| AC123768.1 | 0.159233 | 0.391183 | 1.296707 | 1.68E-05 | 3.49E-05 |
| AC124016.1 | 0.396681 | 0.827061 | 1.060013 | 4.16E-10 | 2.10E-09 |
| AC124067.3 | 0.315509 | 1.034387 | 1.713021 | 5.87E-08 | 1.96E-07 |
| AC124248.2 | 0.060874 | 0.2129 | 1.806266 | 6.56E-08 | 2.17E-07 |
| AC124283.3 | 0.291167 | 1.045684 | 1.844525 | 0.001188 | 0.001679 |
| AC124862.1 | 0.062872 | 0.219994 | 1.806972 | 0.018554 | 0.020422 |
| AC125494.3 | 0.073695 | 0.216521 | 1.554863 | 0.011018 | 0.012722 |
| AC126773.3 | 0.071081 | 0.202983 | 1.513816 | 0.011599 | 0.013351 |
| AC127024.3 | 0.186802 | 0.940789 | 2.332359 | 5.09E-05 | 9.54E-05 |
| AC127035.1 | 0.085114 | 0.42864 | 2.332298 | 0.039874 | 0.040876 |
| AC127164.1 | 0.05033 | 0.248847 | 2.305761 | 1.50E-09 | 6.80E-09 |
| AC131025.3 | 0.080549 | 0.218744 | 1.441305 | 0.001669 | 0.002305 |
| AC131159.1 | 0.510095 | 1.066119 | 1.063529 | 0.0156 | 0.017474 |
| AC131568.1 | 0.202753 | 0.809561 | 1.997413 | 0.00612 | 0.007531 |
| AC131571.1 | 0.035737 | 0.152108 | 2.089594 | 0.001985 | 0.002701 |
| AC131934.1 | 0.169091 | 0.53242 | 1.654767 | 0.004306 | 0.005456 |
| AC131953.1 | 0.133284 | 0.365406 | 1.455001 | 0.03341 | 0.034674 |
| AC131971.1 | 0.558928 | 1.573704 | 1.49343 | 6.86E-07 | 1.85E-06 |
| AC132192.1 | 0.29084 | 0.958083 | 1.719928 | 2.61E-07 | 7.60E-07 |
| AC132192.2 | 0.801179 | 2.165285 | 1.43436 | 7.55E-14 | 8.16E-13 |
| AC132938.2 | 0.178737 | 0.362512 | 1.020188 | 0.009397 | 0.011058 |
| AC133540.1 | 0.087323 | 0.794788 | 3.186133 | 5.93E-07 | 1.62E-06 |
| AC133644.1 | 2.687367 | 1.017225 | -1.40155 | 8.47E-14 | 8.99E-13 |
| AC134407.1 | 0.116174 | 0.484005 | 2.05873 | 0.000601 | 0.000906 |
| AC135050.4 | 0.133923 | 0.459202 | 1.777724 | 6.85E-07 | 1.85E-06 |
| AC135178.4 | 0.28514 | 0.724837 | 1.345989 | 5.86E-07 | 1.60E-06 |
| AC135279.3 | 0.060974 | 0.186009 | 1.609094 | 1.41E-05 | 2.97E-05 |
| AC137770.1 | 0.236984 | 0.508702 | 1.102032 | 0.022478 | 0.024261 |
| AC137932.2 | 0.119351 | 0.519194 | 2.12106 | 6.94E-10 | 3.37E-09 |
| AC137932.3 | 0.49894 | 1.364099 | 1.451012 | 2.85E-14 | 3.27E-13 |
| AC138123.2 | 0.026477 | 0.175494 | 2.728622 | 0.001805 | 0.002475 |
| AC138932.4 | 0.542949 | 1.185578 | 1.126703 | 0.00283 | 0.003742 |
| AC141002.1 | 0.759751 | 1.641153 | 1.111111 | 1.26E-05 | 2.68E-05 |
| AC145207.8 | 0.579563 | 1.347013 | 1.216727 | 0.00098 | 0.001408 |
| AC145423.1 | 0.432179 | 1.83492 | 2.086016 | 5.69E-13 | 5.15E-12 |
| AC145423.2 | 0.437647 | 1.214224 | 1.472196 | 4.75E-09 | 1.99E-08 |
| AC146507.3 | 0.307937 | 0.717246 | 1.219831 | 0.001165 | 0.00165 |
| AC211433.1 | 0.146628 | 0.694759 | 2.244353 | 5.36E-07 | 1.48E-06 |
| AC211476.2 | 0.177658 | 0.8909 | 2.326159 | 2.94E-08 | 1.05E-07 |
| AC215522.2 | 0.040744 | 0.148861 | 1.869298 | 0.00215 | 0.002906 |
| AC239584.1 | 0.09583 | 1.320677 | 3.784652 | 2.35E-11 | 1.53E-10 |
| AC243772.2 | 0.031577 | 0.110941 | 1.812862 | 1.10E-05 | 2.38E-05 |
| AC243967.2 | 0.332956 | 1.845957 | 2.470966 | 6.60E-08 | 2.18E-07 |
| AC244093.4 | 0.210963 | 0.692053 | 1.713893 | 0.009324 | 0.010987 |
| AC244093.5 | 0.179749 | 0.592072 | 1.71979 | 2.42E-06 | 5.90E-06 |
| AC244100.2 | 0.283477 | 0.115313 | -1.29767 | 4.80E-16 | 8.40E-15 |
| AC244517.7 | 0.385998 | 1.393868 | 1.85243 | 0.014872 | 0.016699 |
| AC245060.5 | 0.652786 | 1.459203 | 1.160499 | 5.68E-06 | 1.30E-05 |
| AC245884.10 | 0.141558 | 0.963844 | 2.767405 | 0.000369 | 0.000579 |
| AC245884.8 | 1.109381 | 3.584081 | 1.691848 | 2.05E-14 | 2.41E-13 |
| ACBD3-AS1 | 0.629667 | 1.593247 | 1.339308 | 2.60E-06 | 6.34E-06 |
| ACSL3-AS1 | 0.208284 | 0.54709 | 1.393227 | 0.000131 | 0.000225 |
| ADAMTS9-AS1 | 1.763288 | 0.317668 | -2.47268 | 8.96E-15 | 1.17E-13 |
| ADAMTS9-AS2 | 0.579081 | 0.142127 | -2.02658 | 5.76E-18 | 1.68E-16 |
| ADD3-AS1 | 0.3069 | 0.97565 | 1.668595 | 2.08E-11 | 1.37E-10 |
| ADNP-AS1 | 2.178191 | 4.648554 | 1.093652 | 1.21E-09 | 5.60E-09 |
| AF111169.4 | 0.078209 | 0.2145 | 1.455568 | 0.001382 | 0.001927 |
| AF117829.1 | 0.568237 | 1.423833 | 1.325215 | 1.18E-10 | 6.71E-10 |
| AF127936.1 | 0.329414 | 0.160069 | -1.04121 | 1.25E-11 | 8.82E-11 |
| AF165147.1 | 0.260428 | 0.088192 | -1.56216 | 2.75E-18 | 8.82E-17 |
| AF230666.1 | 0.184479 | 0.438017 | 1.247531 | 0.031636 | 0.03302 |
| AF230666.2 | 0.053621 | 0.307138 | 2.518019 | 3.75E-05 | 7.21E-05 |
| AF241728.2 | 0.158765 | 0.33307 | 1.068932 | 0.002784 | 0.003684 |
| AF287957.1 | 0.526995 | 0.256311 | -1.03989 | 1.43E-12 | 1.20E-11 |
| AJ239328.1 | 0.050742 | 0.193593 | 1.931782 | 0.000578 | 0.000875 |
| AL008718.3 | 0.374833 | 0.855461 | 1.190456 | 0.00764 | 0.009191 |
| AL020997.2 | 0.3751 | 1.178103 | 1.651119 | 0.003776 | 0.004851 |
| AL021392.1 | 0.081223 | 0.34815 | 2.099744 | 1.26E-14 | 1.53E-13 |
| AL021578.1 | 0.586221 | 2.472399 | 2.076395 | 9.85E-13 | 8.54E-12 |
| AL021918.4 | 0.084495 | 0.302297 | 1.839022 | 6.86E-06 | 1.55E-05 |
| AL021978.1 | 0.754133 | 1.744875 | 1.210234 | 1.10E-11 | 7.92E-11 |
| AL022157.1 | 0.51103 | 1.152587 | 1.173396 | 0.000196 | 0.000327 |
| AL022311.1 | 0.160016 | 0.411341 | 1.362115 | 2.78E-18 | 8.82E-17 |
| AL022322.1 | 2.133205 | 5.001966 | 1.229473 | 2.03E-09 | 9.01E-09 |
| AL031005.1 | 0.103249 | 0.381194 | 1.8844 | 0.015901 | 0.017746 |
| AL031275.1 | 0.278872 | 1.79085 | 2.682969 | 7.07E-05 | 0.000129 |
| AL031595.3 | 0.0452 | 0.180307 | 1.996065 | 7.20E-05 | 0.000131 |
| AL031651.2 | 0.529005 | 1.146416 | 1.115778 | 0.041585 | 0.042439 |
| AL031666.2 | 0.157328 | 0.45886 | 1.54428 | 3.32E-05 | 6.51E-05 |
| AL031667.3 | 0.246405 | 1.06861 | 2.116634 | 4.05E-09 | 1.71E-08 |
| AL031670.1 | 1.134658 | 2.326717 | 1.036038 | 0.000981 | 0.001408 |
| AL031673.1 | 1.655167 | 3.79347 | 1.196541 | 1.94E-08 | 7.10E-08 |
| AL031705.1 | 0.226777 | 0.572392 | 1.335731 | 1.27E-05 | 2.70E-05 |
| AL031710.2 | 0.207947 | 1.102304 | 2.406238 | 1.43E-16 | 2.91E-15 |
| AL031716.1 | 1.04517 | 2.778751 | 1.410699 | 1.93E-10 | 1.05E-09 |
| AL031717.1 | 0.588316 | 1.253963 | 1.091831 | 7.90E-05 | 0.000142 |
| AL031770.1 | 0.098381 | 0.422537 | 2.102619 | 4.47E-05 | 8.46E-05 |
| AL031985.3 | 1.497707 | 3.795999 | 1.341724 | 3.89E-17 | 8.71E-16 |
| AL032819.1 | 0.053749 | 0.364963 | 2.763444 | 1.12E-05 | 2.41E-05 |
| AL033381.3 | 0.009367 | 0.358926 | 5.259886 | 7.93E-15 | 1.06E-13 |
| AL033543.1 | 0.012349 | 0.112461 | 3.186978 | 1.87E-09 | 8.33E-09 |
| AL034405.1 | 0.050993 | 0.210913 | 2.048276 | 0.028477 | 0.029944 |
| AL034422.1 | 0.152635 | 0.345648 | 1.179218 | 3.23E-05 | 6.36E-05 |
| AL034550.1 | 1.06136 | 2.370852 | 1.159491 | 2.13E-05 | 4.34E-05 |
| AL034550.3 | 0.240135 | 0.511131 | 1.089849 | 3.57E-06 | 8.48E-06 |
| AL035071.1 | 5.828198 | 11.93316 | 1.033854 | 3.21E-10 | 1.68E-09 |
| AL035071.2 | 0.311198 | 1.470543 | 2.240446 | 3.83E-06 | 9.02E-06 |
| AL035416.1 | 0.085844 | 0.577743 | 2.750636 | 0.000325 | 0.000516 |
| AL035458.2 | 0.225005 | 0.845704 | 1.910198 | 1.09E-07 | 3.44E-07 |
| AL049539.1 | 0.250272 | 1.873683 | 2.904308 | 7.90E-14 | 8.49E-13 |
| AL049646.1 | 0.052753 | 0.212041 | 2.007004 | 0.000606 | 0.00091 |
| AL050403.2 | 0.214623 | 0.100548 | -1.09392 | 1.36E-11 | 9.49E-11 |
| AL078604.2 | 0.105186 | 0.298044 | 1.502584 | 0.000157 | 0.000266 |
| AL080317.1 | 0.614702 | 1.57542 | 1.357777 | 1.92E-11 | 1.28E-10 |
| AL096701.3 | 0.22524 | 0.45842 | 1.025211 | 0.004101 | 0.005224 |
| AL109614.1 | 1.087181 | 3.991061 | 1.87618 | 9.02E-09 | 3.52E-08 |
| AL109615.2 | 0.017616 | 0.167756 | 3.251387 | 1.28E-20 | 8.09E-19 |
| AL109615.4 | 1.049086 | 2.277253 | 1.118162 | 5.50E-09 | 2.26E-08 |
| AL109804.1 | 0.061942 | 0.271332 | 2.131072 | 3.01E-05 | 5.98E-05 |
| AL109811.1 | 0.07724 | 0.166928 | 1.111816 | 8.42E-07 | 2.24E-06 |
| AL109840.2 | 0.052409 | 0.13439 | 1.358526 | 0.000287 | 0.00046 |
| AL109984.1 | 0.326612 | 0.945662 | 1.533749 | 0.000479 | 0.000736 |
| AL110115.1 | 0.207519 | 1.069391 | 2.365477 | 2.11E-05 | 4.30E-05 |
| AL110115.2 | 0.1687 | 0.340302 | 1.012355 | 0.029557 | 0.031009 |
| AL117327.1 | 0.090812 | 0.34027 | 1.905731 | 1.26E-09 | 5.81E-09 |
| AL117379.1 | 1.49494 | 4.897991 | 1.712103 | 3.73E-12 | 2.87E-11 |
| AL117382.1 | 5.129802 | 22.12367 | 2.108616 | 1.33E-11 | 9.30E-11 |
| AL121583.1 | 0.672572 | 1.487396 | 1.145028 | 5.97E-06 | 1.36E-05 |
| AL121782.1 | 0.244321 | 0.593673 | 1.280891 | 6.24E-05 | 0.000115 |
| AL121845.4 | 0.45594 | 1.327689 | 1.542003 | 0.000148 | 0.000253 |
| AL121895.1 | 0.111644 | 0.645163 | 2.530755 | 3.87E-08 | 1.36E-07 |
| AL121987.1 | 0.373293 | 0.164669 | -1.18074 | 1.31E-11 | 9.17E-11 |
| AL121987.2 | 0.218916 | 0.454264 | 1.053152 | 7.49E-06 | 1.67E-05 |
| AL121992.3 | 0.639986 | 1.42457 | 1.154414 | 1.74E-05 | 3.61E-05 |
| AL132642.1 | 2.233016 | 0.357376 | -2.64348 | 1.22E-21 | 1.18E-19 |
| AL132765.2 | 0.222686 | 0.487699 | 1.130981 | 0.027072 | 0.028598 |
| AL133230.1 | 0.178405 | 0.639449 | 1.841676 | 5.72E-09 | 2.33E-08 |
| AL133243.2 | 1.492009 | 3.59238 | 1.267683 | 1.52E-05 | 3.19E-05 |
| AL133243.3 | 0.548365 | 1.142635 | 1.059156 | 7.10E-06 | 1.59E-05 |
| AL133284.1 | 0.030823 | 0.11206 | 1.862184 | 0.006361 | 0.00779 |
| AL133330.1 | 0.460637 | 1.936613 | 2.071833 | 1.55E-12 | 1.30E-11 |
| AL133410.1 | 0.705695 | 1.676887 | 1.248668 | 6.93E-11 | 4.22E-10 |
| AL133520.1 | 2.372837 | 5.118681 | 1.109159 | 1.27E-09 | 5.84E-09 |
| AL135902.1 | 0.110112 | 0.373187 | 1.760933 | 0.000237 | 0.000388 |
| AL135926.2 | 0.069912 | 0.442836 | 2.663167 | 0.000328 | 0.000519 |
| AL136115.1 | 0.39896 | 1.119991 | 1.489169 | 0.001961 | 0.002672 |
| AL136115.2 | 0.311272 | 1.775614 | 2.51207 | 3.60E-05 | 6.96E-05 |
| AL136171.2 | 0.044888 | 0.139996 | 1.640975 | 0.028726 | 0.030172 |
| AL136526.1 | 0.067277 | 0.151825 | 1.174227 | 0.004786 | 0.006022 |
| AL136988.2 | 0.285753 | 0.724432 | 1.34208 | 0.000289 | 0.000462 |
| AL137058.2 | 0.050898 | 0.116323 | 1.192464 | 0.010957 | 0.012667 |
| AL137244.1 | 0.356693 | 1.406254 | 1.979102 | 4.12E-11 | 2.62E-10 |
| AL137246.1 | 0.049219 | 0.209591 | 2.090298 | 0.003529 | 0.004569 |
| AL137782.1 | 0.245547 | 0.984193 | 2.002945 | 5.68E-12 | 4.26E-11 |
| AL137847.1 | 0.065372 | 0.14055 | 1.104333 | 0.01193 | 0.013697 |
| AL138689.1 | 0.935435 | 3.334636 | 1.83382 | 3.22E-06 | 7.72E-06 |
| AL138820.1 | 0.187656 | 0.479318 | 1.352894 | 0.001604 | 0.002221 |
| AL138921.1 | 0.174753 | 0.399024 | 1.191155 | 1.54E-06 | 3.90E-06 |
| AL138963.1 | 0.293921 | 2.452263 | 3.060614 | 0.000313 | 0.000499 |
| AL138999.1 | 0.343172 | 1.047962 | 1.610583 | 0.000115 | 0.0002 |
| AL138999.2 | 0.073744 | 0.52687 | 2.836845 | 1.12E-06 | 2.90E-06 |
| AL139082.1 | 0.216793 | 0.462057 | 1.091751 | 0.000581 | 0.000878 |
| AL139147.1 | 0.022109 | 0.516438 | 4.54587 | 8.93E-05 | 0.000159 |
| AL139274.2 | 0.101126 | 0.212346 | 1.070272 | 1.98E-05 | 4.05E-05 |
| AL139317.5 | 0.028053 | 0.116825 | 2.058096 | 0.023016 | 0.024711 |
| AL157395.1 | 0.117695 | 0.48833 | 2.052798 | 0.008881 | 0.010499 |
| AL157400.2 | 0.043572 | 0.1485 | 1.76899 | 3.40E-07 | 9.67E-07 |
| AL157400.4 | 0.180658 | 0.366966 | 1.022384 | 0.010034 | 0.011713 |
| AL157786.1 | 1.108277 | 2.327999 | 1.070772 | 0.024454 | 0.026103 |
| AL157827.2 | 0.07677 | 0.204572 | 1.413996 | 1.19E-06 | 3.08E-06 |
| AL157838.1 | 0.77777 | 1.664825 | 1.097955 | 1.95E-05 | 4.01E-05 |
| AL157895.1 | 0.306181 | 0.133699 | -1.19539 | 1.05E-12 | 9.04E-12 |
| AL157938.3 | 0.080598 | 0.295321 | 1.873475 | 0.006621 | 0.008087 |
| AL158063.1 | 0.40976 | 1.273399 | 1.635831 | 9.24E-08 | 2.95E-07 |
| AL158151.4 | 0.215891 | 0.635611 | 1.557842 | 0.000113 | 0.000196 |
| AL158198.2 | 0.216479 | 0.096778 | -1.16148 | 3.61E-13 | 3.38E-12 |
| AL158211.1 | 0.3756 | 0.144783 | -1.3753 | 2.49E-13 | 2.41E-12 |
| AL158212.2 | 0.232849 | 0.562591 | 1.272693 | 0.021708 | 0.023582 |
| AL158825.2 | 0.530867 | 1.146454 | 1.110755 | 0.001005 | 0.00144 |
| AL160171.1 | 0.161219 | 0.40664 | 1.334735 | 1.14E-05 | 2.46E-05 |
| AL161430.1 | 0.323472 | 1.586076 | 2.293748 | 1.20E-05 | 2.58E-05 |
| AL161457.1 | 0.335402 | 0.095487 | -1.81251 | 4.82E-22 | 4.92E-20 |
| AL161663.1 | 0.050312 | 0.35079 | 2.801642 | 6.28E-09 | 2.52E-08 |
| AL161729.2 | 0.117558 | 0.865213 | 2.879681 | 0.000117 | 0.000202 |
| AL161729.3 | 0.291244 | 0.712354 | 1.290366 | 2.00E-05 | 4.11E-05 |
| AL161729.4 | 0.511721 | 2.460314 | 2.265413 | 9.98E-14 | 1.04E-12 |
| AL161756.1 | 0.094447 | 0.23442 | 1.311528 | 2.39E-09 | 1.05E-08 |
| AL161891.1 | 0.494626 | 1.783561 | 1.850352 | 3.11E-16 | 5.59E-15 |
| AL162424.1 | 2.874879 | 0.839838 | -1.77532 | 1.15E-14 | 1.43E-13 |
| AL162595.1 | 2.055884 | 6.125326 | 1.575028 | 5.87E-20 | 2.76E-18 |
| AL162724.2 | 0.839902 | 1.957763 | 1.220913 | 5.01E-05 | 9.40E-05 |
| AL162741.1 | 0.161635 | 0.355175 | 1.135793 | 1.69E-07 | 5.06E-07 |
| AL353708.3 | 0.32403 | 0.910756 | 1.490937 | 2.63E-10 | 1.39E-09 |
| AL353796.1 | 0.861121 | 2.19254 | 1.348316 | 1.10E-14 | 1.39E-13 |
| AL353801.1 | 1.617744 | 0.33089 | -2.28956 | 1.59E-22 | 2.21E-20 |
| AL353801.3 | 0.185147 | 0.518668 | 1.486145 | 6.21E-05 | 0.000115 |
| AL353804.2 | 0.227021 | 1.447952 | 2.673116 | 4.93E-05 | 9.27E-05 |
| AL354696.1 | 1.804835 | 4.035698 | 1.160951 | 3.06E-11 | 1.97E-10 |
| AL354726.1 | 0.218465 | 0.991244 | 2.181837 | 0.010192 | 0.011865 |
| AL354733.2 | 0.050221 | 0.136609 | 1.443689 | 0.005295 | 0.006603 |
| AL354813.1 | 0.048765 | 0.20878 | 2.098059 | 0.000167 | 0.000281 |
| AL354993.1 | 0.109942 | 0.430426 | 1.969026 | 0.001229 | 0.001732 |
| AL354993.2 | 0.580019 | 1.967069 | 1.761876 | 0.00068 | 0.001014 |
| AL355001.1 | 0.216523 | 0.556245 | 1.361199 | 0.003717 | 0.004784 |
| AL355075.2 | 1.173805 | 2.498889 | 1.090094 | 6.33E-06 | 1.44E-05 |
| AL355075.6 | 0.687607 | 1.898208 | 1.464982 | 1.62E-10 | 8.94E-10 |
| AL355112.1 | 0.039484 | 0.248041 | 2.651251 | 2.58E-07 | 7.52E-07 |
| AL355312.2 | 0.10903 | 0.331747 | 1.605354 | 1.82E-06 | 4.53E-06 |
| AL355377.4 | 0.215651 | 0.453839 | 1.073481 | 0.018237 | 0.020133 |
| AL355385.1 | 0.287381 | 0.796293 | 1.470332 | 6.40E-07 | 1.74E-06 |
| AL355388.1 | 0.505481 | 1.067844 | 1.078971 | 5.81E-08 | 1.94E-07 |
| AL355472.3 | 0.339928 | 0.748321 | 1.138429 | 0.005458 | 0.00678 |
| AL355488.1 | 2.37676 | 4.976784 | 1.066217 | 6.51E-07 | 1.77E-06 |
| AL355834.2 | 0.116698 | 0.469074 | 2.00704 | 0.000423 | 0.000656 |
| AL355999.1 | 0.336114 | 0.812103 | 1.272712 | 1.86E-09 | 8.33E-09 |
| AL356124.1 | 0.074644 | 0.235427 | 1.657178 | 0.006272 | 0.007691 |
| AL356299.2 | 0.41597 | 2.541519 | 2.61114 | 1.36E-18 | 4.53E-17 |
| AL356804.1 | 0.174772 | 0.521601 | 1.577472 | 4.77E-08 | 1.63E-07 |
| AL356966.1 | 0.069937 | 0.213019 | 1.606847 | 9.79E-07 | 2.56E-06 |
| AL357079.3 | 1.469553 | 3.06035 | 1.058319 | 3.10E-08 | 1.10E-07 |
| AL357140.4 | 0.15017 | 0.372513 | 1.310696 | 0.007333 | 0.008856 |
| AL357874.2 | 0.112351 | 0.322634 | 1.521885 | 0.012025 | 0.01379 |
| AL358115.1 | 0.382272 | 0.924368 | 1.273867 | 1.58E-06 | 3.98E-06 |
| AL358472.5 | 0.208453 | 0.570943 | 1.453621 | 2.31E-10 | 1.23E-09 |
| AL359232.1 | 0.153909 | 0.464626 | 1.593989 | 8.16E-18 | 2.31E-16 |
| AL359636.2 | 0.115326 | 0.318642 | 1.466223 | 0.011456 | 0.013211 |
| AL359880.1 | 0.226405 | 1.244668 | 2.458785 | 0.002918 | 0.003854 |
| AL359921.1 | 0.208649 | 0.506255 | 1.278787 | 0.000925 | 0.001339 |
| AL359922.2 | 0.260635 | 0.970638 | 1.896904 | 1.10E-14 | 1.39E-13 |
| AL360093.1 | 0.062209 | 0.324441 | 2.382756 | 0.0017 | 0.002343 |
| AL365361.1 | 4.074658 | 1.0963 | -1.89404 | 4.65E-19 | 1.82E-17 |
| AL390195.1 | 0.397416 | 0.996249 | 1.325855 | 1.58E-08 | 5.90E-08 |
| AL390719.3 | 0.216502 | 0.560103 | 1.37131 | 7.34E-06 | 1.64E-05 |
| AL390755.3 | 0.058949 | 0.204574 | 1.795085 | 5.52E-05 | 0.000103 |
| AL390957.1 | 0.089286 | 0.324933 | 1.863637 | 2.41E-10 | 1.28E-09 |
| AL390961.2 | 0.121309 | 0.343336 | 1.500932 | 0.00805 | 0.009627 |
| AL391421.1 | 0.055588 | 0.249263 | 2.164814 | 0.000335 | 0.000529 |
| AL391427.1 | 0.157067 | 0.450622 | 1.520534 | 0.004544 | 0.005741 |
| AL391832.3 | 0.159677 | 0.326242 | 1.030787 | 0.007184 | 0.008711 |
| AL392089.1 | 0.170519 | 0.650205 | 1.930965 | 0.003732 | 0.004801 |
| AL442067.1 | 0.365842 | 2.254282 | 2.623376 | 4.90E-09 | 2.04E-08 |
| AL442067.2 | 0.280684 | 1.201077 | 2.097311 | 4.15E-05 | 7.91E-05 |
| AL442125.2 | 0.567067 | 1.587196 | 1.484888 | 0.000349 | 0.00055 |
| AL442128.2 | 0.232198 | 0.609715 | 1.392782 | 1.02E-07 | 3.23E-07 |
| AL445222.1 | 0.597707 | 2.02615 | 1.761231 | 2.52E-13 | 2.42E-12 |
| AL450344.3 | 0.23297 | 0.795331 | 1.771413 | 0.027557 | 0.029044 |
| AL450992.2 | 0.047279 | 0.148969 | 1.655737 | 0.000186 | 0.000312 |
| AL512283.3 | 0.279472 | 0.087691 | -1.6722 | 3.17E-20 | 1.72E-18 |
| AL512506.1 | 0.274607 | 1.472033 | 2.42237 | 1.27E-06 | 3.28E-06 |
| AL512643.2 | 0.015535 | 0.22843 | 3.878171 | 5.45E-05 | 0.000102 |
| AL512652.1 | 0.271093 | 1.136353 | 2.067551 | 9.26E-11 | 5.50E-10 |
| AL512656.1 | 0.220033 | 0.555148 | 1.335156 | 0.004209 | 0.005343 |
| AL513123.1 | 0.113005 | 1.089283 | 3.268925 | 5.38E-13 | 4.91E-12 |
| AL513327.1 | 0.696926 | 1.553061 | 1.156038 | 4.39E-06 | 1.02E-05 |
| AL513327.2 | 0.185251 | 0.514767 | 1.474437 | 0.005404 | 0.00673 |
| AL583824.1 | 0.045316 | 0.231637 | 2.353763 | 0.019528 | 0.021404 |
| AL590064.1 | 3.903516 | 10.35559 | 1.407564 | 1.13E-09 | 5.28E-09 |
| AL590096.1 | 0.297184 | 0.866856 | 1.544436 | 5.55E-09 | 2.27E-08 |
| AL590101.1 | 0.424705 | 0.897225 | 1.079011 | 0.000247 | 0.000402 |
| AL590282.2 | 0.2076 | 0.796247 | 1.93941 | 0.003545 | 0.004583 |
| AL590483.4 | 0.043181 | 0.761579 | 4.140512 | 4.42E-20 | 2.25E-18 |
| AL590723.1 | 0.625342 | 1.785923 | 1.513953 | 0.000444 | 0.000687 |
| AL590729.1 | 0.223114 | 0.549335 | 1.299906 | 0.002428 | 0.003247 |
| AL590822.2 | 0.057774 | 0.136168 | 1.236888 | 0.013547 | 0.015323 |
| AL591069.1 | 0.056384 | 0.794335 | 3.816396 | 2.51E-08 | 9.01E-08 |
| AL592166.1 | 0.131128 | 0.314182 | 1.260626 | 1.72E-07 | 5.13E-07 |
| AL592301.1 | 0.253491 | 0.809803 | 1.675638 | 5.11E-08 | 1.73E-07 |
| AL592546.3 | 0.070453 | 0.297251 | 2.076939 | 0.029965 | 0.031401 |
| AL596223.1 | 1.7587 | 0.586291 | -1.58482 | 3.30E-13 | 3.13E-12 |
| AL596247.1 | 0.296988 | 1.06433 | 1.841467 | 0.006949 | 0.008454 |
| AL606537.1 | 0.032009 | 0.266989 | 3.060217 | 0.00023 | 0.000378 |
| AL645768.1 | 0.019265 | 0.211304 | 3.455259 | 0.044768 | 0.045236 |
| AL731563.3 | 0.302695 | 0.647284 | 1.096533 | 7.05E-09 | 2.81E-08 |
| AL731569.1 | 0.450986 | 1.055372 | 1.226597 | 1.89E-13 | 1.85E-12 |
| AL807757.2 | 0.123251 | 0.681721 | 2.467579 | 8.32E-05 | 0.000149 |
| AL929236.1 | 0.105314 | 0.3445 | 1.709809 | 0.000578 | 0.000875 |
| ALG13-AS1 | 1.225247 | 2.928128 | 1.256907 | 0.0302 | 0.031629 |
| ALMS1-IT1 | 0.362253 | 1.195966 | 1.723105 | 3.09E-12 | 2.41E-11 |
| ANKRD10-IT1 | 14.21804 | 34.08677 | 1.261489 | 1.87E-06 | 4.64E-06 |
| AOAH-IT1 | 0.254167 | 0.874263 | 1.782288 | 0.004074 | 0.005195 |
| AP000331.1 | 0.111144 | 0.912055 | 3.036689 | 8.82E-07 | 2.33E-06 |
| AP000487.1 | 0.332367 | 0.803897 | 1.274231 | 1.37E-15 | 2.10E-14 |
| AP000640.2 | 0.045156 | 0.138959 | 1.62168 | 0.00018 | 0.000302 |
| AP000679.1 | 0.037937 | 0.112726 | 1.571132 | 0.000861 | 0.00126 |
| AP000692.1 | 0.531567 | 1.326331 | 1.319116 | 4.50E-06 | 1.05E-05 |
| AP000704.1 | 0.204547 | 0.451678 | 1.142865 | 1.21E-12 | 1.03E-11 |
| AP000763.3 | 0.159284 | 0.451032 | 1.501632 | 0.013963 | 0.015775 |
| AP000786.1 | 0.703453 | 1.807475 | 1.361449 | 0.014766 | 0.01659 |
| AP000866.6 | 0.422693 | 1.202599 | 1.508473 | 1.39E-07 | 4.25E-07 |
| AP001001.1 | 0.198233 | 0.464235 | 1.227661 | 0.011747 | 0.013495 |
| AP001029.2 | 0.071544 | 0.235625 | 1.719587 | 1.97E-08 | 7.19E-08 |
| AP001160.1 | 0.123967 | 0.601335 | 2.278207 | 1.25E-14 | 1.53E-13 |
| AP001160.4 | 0.526737 | 1.297798 | 1.300911 | 0.000546 | 0.000832 |
| AP001269.1 | 0.337705 | 0.167097 | -1.01508 | 1.27E-11 | 8.97E-11 |
| AP001429.1 | 0.427195 | 2.557587 | 2.581815 | 6.58E-07 | 1.78E-06 |
| AP001442.1 | 0.133767 | 0.572028 | 2.09636 | 0.009448 | 0.011105 |
| AP001469.2 | 0.681707 | 1.509192 | 1.146553 | 1.59E-07 | 4.78E-07 |
| AP001469.3 | 1.073098 | 3.003201 | 1.48472 | 1.45E-18 | 4.75E-17 |
| AP001619.1 | 0.216544 | 0.684933 | 1.661302 | 1.12E-05 | 2.42E-05 |
| AP001893.1 | 0.610956 | 1.29992 | 1.089283 | 0.003346 | 0.004365 |
| AP002336.2 | 0.670519 | 1.635838 | 1.286681 | 3.74E-10 | 1.92E-09 |
| AP002490.1 | 0.138407 | 0.29387 | 1.086261 | 0.000178 | 0.000298 |
| AP002748.6 | 0.061535 | 0.137129 | 1.156059 | 0.001334 | 0.001866 |
| AP002812.3 | 0.176174 | 0.55125 | 1.645702 | 0.006179 | 0.007582 |
| AP002907.1 | 0.49496 | 1.634783 | 1.723714 | 9.37E-08 | 2.98E-07 |
| AP002982.2 | 0.139291 | 0.295714 | 1.086105 | 0.022553 | 0.024274 |
| AP003086.1 | 0.086602 | 0.329401 | 1.927365 | 0.000156 | 0.000264 |
| AP003170.4 | 0.10696 | 0.263483 | 1.30063 | 0.010105 | 0.011786 |
| AP003352.1 | 0.860433 | 2.594787 | 1.592482 | 3.44E-17 | 7.79E-16 |
| AP003419.2 | 0.3345 | 0.796305 | 1.251314 | 0.03333 | 0.034611 |
| AP003548.1 | 0.37527 | 0.092621 | -2.01852 | 3.56E-18 | 1.05E-16 |
| AP003783.1 | 0.029356 | 0.192816 | 2.715508 | 0.000595 | 0.000897 |
| AP005230.1 | 0.012423 | 0.202317 | 4.025503 | 2.99E-17 | 6.94E-16 |
| AP006545.1 | 0.333505 | 0.869212 | 1.382001 | 1.07E-07 | 3.37E-07 |
| AP006545.3 | 0.12464 | 0.263884 | 1.082141 | 0.003933 | 0.005035 |
| AP007216.2 | 0.119795 | 0.386675 | 1.690549 | 0.001805 | 0.002475 |
| ARAP1-AS2 | 0.146926 | 0.618498 | 2.073683 | 7.85E-05 | 0.000141 |
| ARHGAP15-AS1 | 0.660772 | 0.147756 | -2.16093 | 1.06E-32 | 1.95E-29 |
| ARHGAP27P1-BPTFP1-KPNA2P3 | 0.482428 | 1.237327 | 1.358842 | 1.57E-12 | 1.30E-11 |
| ARHGEF38-IT1 | 0.289128 | 1.88841 | 2.707392 | 2.84E-10 | 1.50E-09 |
| ASAP1-IT2 | 0.050877 | 0.315756 | 2.63373 | 0.008353 | 0.009957 |
| BACH1-IT1 | 0.423495 | 0.94343 | 1.155569 | 0.021757 | 0.023621 |
| BCL2L1-AS1 | 0.275712 | 2.373795 | 3.105964 | 0.008251 | 0.009842 |
| BX322234.1 | 0.275493 | 0.796519 | 1.531694 | 0.000176 | 0.000296 |
| BX842570.1 | 0.8325 | 2.952231 | 1.826284 | 9.86E-18 | 2.74E-16 |
| C15orf54 | 0.010498 | 0.188714 | 4.16806 | 8.09E-08 | 2.61E-07 |
| C1orf147 | 0.208451 | 0.520962 | 1.32147 | 1.37E-07 | 4.20E-07 |
| C2-AS1 | 0.116086 | 0.87476 | 2.913693 | 2.53E-14 | 2.93E-13 |
| C2orf27A | 0.472628 | 1.484287 | 1.650993 | 3.02E-17 | 6.94E-16 |
| C3orf35 | 0.204209 | 0.523355 | 1.357741 | 9.52E-12 | 6.94E-11 |
| C5orf66 | 0.241284 | 0.599138 | 1.312157 | 3.86E-16 | 6.82E-15 |
| C9orf147 | 0.255116 | 0.520042 | 1.027473 | 7.57E-08 | 2.47E-07 |
| CAPN10-DT | 0.91057 | 2.378205 | 1.385032 | 2.47E-17 | 5.82E-16 |
| CARMN | 6.102021 | 2.424628 | -1.33152 | 3.36E-07 | 9.58E-07 |
| CARNMT1-AS1 | 0.399786 | 0.944753 | 1.240709 | 0.003903 | 0.005 |
| CARS1-AS1 | 0.032867 | 0.135422 | 2.042728 | 0.002017 | 0.002741 |
| CASC15 | 0.123563 | 0.524647 | 2.086103 | 1.60E-11 | 1.08E-10 |
| CASC18 | 0.406133 | 0.094817 | -2.09873 | 9.42E-15 | 1.21E-13 |
| CASC19 | 0.453005 | 5.694448 | 3.651958 | 1.16E-23 | 3.06E-21 |
| CASK-AS1 | 0.149865 | 0.459281 | 1.615713 | 1.94E-05 | 4.00E-05 |
| CATIP-AS2 | 0.123509 | 0.283902 | 1.200773 | 0.03519 | 0.036425 |
| CCAT2 | 0.065019 | 0.598023 | 3.201276 | 1.48E-09 | 6.72E-09 |
| CD44-AS1 | 0.613337 | 3.081022 | 2.328656 | 1.94E-11 | 1.29E-10 |
| CDC42-IT1 | 0.140223 | 0.702692 | 2.325168 | 0.000791 | 0.001164 |
| CERS6-AS1 | 0.041698 | 0.113352 | 1.442766 | 2.34E-08 | 8.42E-08 |
| CFAP44-AS1 | 0.082049 | 0.279782 | 1.769748 | 0.006133 | 0.007536 |
| CHN2-AS1 | 0.166 | 2.229105 | 3.74721 | 1.63E-12 | 1.35E-11 |
| CLDN10-AS1 | 0.038702 | 1.350213 | 5.124623 | 1.17E-11 | 8.37E-11 |
| COL4A2-AS2 | 0.028665 | 0.203833 | 2.830022 | 4.17E-05 | 7.96E-05 |
| CRAT37 | 0.078879 | 0.925948 | 3.553217 | 5.04E-08 | 1.71E-07 |
| CRNDE | 0.159895 | 1.845749 | 3.529006 | 4.15E-23 | 6.93E-21 |
| CRPPA-AS1 | 0.037863 | 0.105368 | 1.476584 | 0.026549 | 0.028094 |
| CRYZL2P-SEC16B | 0.091733 | 0.319653 | 1.801002 | 1.63E-10 | 8.94E-10 |
| CSE1L-AS1 | 0.067912 | 0.15107 | 1.153489 | 0.044435 | 0.044973 |
| CSNK1G2-AS1 | 0.103414 | 0.373532 | 1.852802 | 3.21E-05 | 6.34E-05 |
| CYP1B1-AS1 | 0.277405 | 0.12293 | -1.17415 | 1.19E-17 | 3.08E-16 |
| CYTOR | 0.434779 | 1.326608 | 1.609388 | 4.04E-19 | 1.69E-17 |
| DCUN1D2-AS | 0.465053 | 1.06092 | 1.189847 | 0.000483 | 0.000742 |
| DELEC1 | 0.116747 | 0.346171 | 1.568106 | 0.000289 | 0.000462 |
| DIAPH2-AS1 | 0.130849 | 0.763907 | 2.545495 | 1.33E-07 | 4.09E-07 |
| DLEU1 | 0.46854 | 1.624333 | 1.793605 | 5.67E-20 | 2.74E-18 |
| DLEU2 | 0.458509 | 1.578868 | 1.783867 | 1.37E-14 | 1.65E-13 |
| DLGAP4-AS1 | 0.187467 | 0.651133 | 1.796313 | 1.24E-10 | 6.97E-10 |
| EDIL3-DT | 0.610665 | 0.183193 | -1.73702 | 3.11E-20 | 1.72E-18 |
| EGOT | 0.067884 | 0.451751 | 2.734389 | 3.13E-08 | 1.11E-07 |
| EIF1AX-AS1 | 0.3184 | 1.28342 | 2.011081 | 0.010467 | 0.012146 |
| EIPR1-IT1 | 0.165879 | 0.55286 | 1.736782 | 8.12E-10 | 3.91E-09 |
| ELOA-AS1 | 0.452947 | 1.012423 | 1.160399 | 7.14E-14 | 7.86E-13 |
| EPN2-AS1 | 0.103374 | 0.37543 | 1.860664 | 0.003314 | 0.004328 |
| EXTL3-AS1 | 0.068681 | 0.302695 | 2.139873 | 5.49E-15 | 7.52E-14 |
| FAM218A | 0.565163 | 0.205593 | -1.45888 | 8.75E-16 | 1.41E-14 |
| FAM78B-AS1 | 1.008109 | 0.376977 | -1.4191 | 6.82E-11 | 4.18E-10 |
| FIRRE | 0.173665 | 1.214202 | 2.805629 | 5.61E-21 | 4.12E-19 |
| FMR1-AS1 | 0.032084 | 0.112996 | 1.816359 | 1.68E-05 | 3.50E-05 |
| FMR1-IT1 | 1.10733 | 2.305226 | 1.057822 | 0.001024 | 0.001464 |
| FO393418.1 | 1.051486 | 0.423708 | -1.31129 | 6.05E-13 | 5.40E-12 |
| FO680682.1 | 0.278679 | 0.735994 | 1.40109 | 0.046381 | 0.046634 |
| FOCAD-AS1 | 0.354363 | 0.865206 | 1.287817 | 8.32E-07 | 2.21E-06 |
| FRMD6-AS2 | 0.690149 | 0.112127 | -2.62178 | 2.40E-26 | 2.21E-23 |
| FSIP2-AS1 | 0.105691 | 0.430889 | 2.027468 | 9.69E-08 | 3.08E-07 |
| FSIP2-AS2 | 0.112321 | 0.683735 | 2.60581 | 3.12E-07 | 8.94E-07 |
| FTX | 1.26696 | 3.948332 | 1.639872 | 6.73E-08 | 2.22E-07 |
| GABPB1-AS1 | 1.530172 | 3.541754 | 1.21077 | 4.36E-08 | 1.51E-07 |
| GAS1RR | 0.547151 | 0.134437 | -2.02501 | 1.24E-11 | 8.80E-11 |
| GAS8-AS1 | 0.195928 | 0.672744 | 1.779735 | 9.31E-10 | 4.40E-09 |
| GK-AS1 | 0.750719 | 2.814313 | 1.906438 | 9.76E-05 | 0.000173 |
| GK-IT1 | 0.27153 | 1.439253 | 2.406136 | 7.49E-06 | 1.67E-05 |
| GNG12-AS1 | 1.473986 | 0.716335 | -1.04102 | 8.05E-15 | 1.06E-13 |
| GRASLND | 0.11773 | 0.33489 | 1.508203 | 6.50E-08 | 2.15E-07 |
| GSTCD-AS1 | 0.111788 | 0.477338 | 2.09424 | 0.002428 | 0.003247 |
| GTF3C2-AS1 | 0.053653 | 0.227928 | 2.086837 | 3.85E-10 | 1.97E-09 |
| HCG25 | 0.182026 | 0.416504 | 1.194189 | 3.78E-10 | 1.94E-09 |
| HDAC2-AS2 | 0.17794 | 0.416396 | 1.226571 | 1.41E-11 | 9.69E-11 |
| HECW2-AS1 | 1.405123 | 0.702022 | -1.00111 | 1.74E-10 | 9.56E-10 |
| HIF1A-AS3 | 0.754044 | 3.344211 | 2.148945 | 0.00019 | 0.000318 |
| HMGA2-AS1 | 0.127565 | 0.661166 | 2.373779 | 8.70E-11 | 5.21E-10 |
| HYMAI | 0.077884 | 0.287944 | 1.886394 | 0.000333 | 0.000526 |
| IFNG-AS1 | 0.289526 | 0.136357 | -1.0863 | 6.27E-09 | 2.52E-08 |
| INE1 | 2.496216 | 5.044106 | 1.014856 | 4.46E-08 | 1.54E-07 |
| INHBA-AS1 | 0.017495 | 0.148068 | 3.081213 | 9.06E-11 | 5.41E-10 |
| INTS6-AS1 | 0.148426 | 0.304846 | 1.03834 | 3.44E-07 | 9.76E-07 |
| INTS9-AS1 | 0.080902 | 0.222525 | 1.459711 | 0.008973 | 0.010594 |
| ISM1-AS1 | 0.086991 | 0.285965 | 1.716905 | 0.003007 | 0.00396 |
| ITFG1-AS1 | 0.237472 | 0.497328 | 1.066439 | 0.022929 | 0.024646 |
| ITPK1-AS1 | 0.068681 | 0.259458 | 1.91751 | 0.02529 | 0.026932 |
| ITPRIP-AS1 | 0.077851 | 0.594548 | 2.933004 | 4.13E-08 | 1.44E-07 |
| JARID2-AS1 | 0.377949 | 1.051039 | 1.475553 | 2.29E-05 | 4.64E-05 |
| JPX | 0.173202 | 0.726102 | 2.067714 | 2.45E-09 | 1.08E-08 |
| KANSL1L-AS1 | 0.342012 | 1.0876 | 1.669031 | 2.56E-07 | 7.46E-07 |
| KCNIP2-AS1 | 0.174307 | 0.425117 | 1.286231 | 2.77E-09 | 1.20E-08 |
| KCNMB2-AS1 | 0.133112 | 0.64746 | 2.282155 | 1.40E-07 | 4.27E-07 |
| KCNQ1OT1 | 0.111172 | 0.669441 | 2.590163 | 3.97E-15 | 5.61E-14 |
| KIAA1614-AS1 | 0.068453 | 0.145523 | 1.088056 | 0.009181 | 0.010833 |
| KLF7-IT1 | 0.26216 | 1.08109 | 2.043964 | 0.00167 | 0.002305 |
| L29074.1 | 0.056056 | 0.287952 | 2.360894 | 0.000122 | 0.000211 |
| LACTB2-AS1 | 0.082802 | 0.230507 | 1.477066 | 1.08E-06 | 2.81E-06 |
| LEF1-AS1 | 0.033263 | 0.178819 | 2.426518 | 3.01E-18 | 9.37E-17 |
| LEMD1-AS1 | 0.01136 | 0.188206 | 4.050216 | 1.39E-10 | 7.73E-10 |
| LENG8-AS1 | 2.589523 | 6.695838 | 1.370578 | 1.86E-16 | 3.64E-15 |
| LINC-PINT | 0.89167 | 2.65482 | 1.574033 | 2.08E-10 | 1.12E-09 |
| LINC00092 | 0.592428 | 0.143447 | -2.04612 | 1.14E-20 | 7.47E-19 |
| LINC00216 | 0.482753 | 1.030271 | 1.093665 | 0.004885 | 0.006133 |
| LINC00240 | 0.068835 | 0.149116 | 1.11522 | 1.22E-05 | 2.61E-05 |
| LINC00244 | 0.312791 | 0.767253 | 1.294505 | 0.019359 | 0.021245 |
| LINC00299 | 0.083623 | 0.201446 | 1.268418 | 1.59E-08 | 5.91E-08 |
| LINC00365 | 0.44303 | 1.48245 | 1.742506 | 6.07E-11 | 3.75E-10 |
| LINC00412 | 0.238305 | 1.092923 | 2.197313 | 1.54E-08 | 5.76E-08 |
| LINC00449 | 0.158814 | 0.371099 | 1.224465 | 3.51E-05 | 6.83E-05 |
| LINC00513 | 1.654891 | 5.111373 | 1.626975 | 1.46E-09 | 6.66E-09 |
| LINC00562 | 0.20014 | 0.575665 | 1.524222 | 3.89E-08 | 1.36E-07 |
| LINC00622 | 0.357807 | 0.914364 | 1.353587 | 7.30E-05 | 0.000132 |
| LINC00624 | 0.154547 | 0.449122 | 1.539067 | 4.41E-06 | 1.03E-05 |
| LINC00628 | 0.430574 | 1.006938 | 1.22564 | 2.24E-10 | 1.20E-09 |
| LINC00630 | 0.274281 | 0.64038 | 1.223271 | 5.59E-13 | 5.09E-12 |
| LINC00658 | 0.02483 | 0.187743 | 2.91859 | 0.000126 | 0.000218 |
| LINC00698 | 0.018826 | 0.191959 | 3.350034 | 1.42E-12 | 1.20E-11 |
| LINC00862 | 0.04057 | 0.208807 | 2.363693 | 2.39E-09 | 1.05E-08 |
| LINC00867 | 0.027677 | 0.124969 | 2.17482 | 0.000307 | 0.00049 |
| LINC00893 | 0.210958 | 0.561527 | 1.412399 | 9.39E-07 | 2.46E-06 |
| LINC00894 | 0.340056 | 1.280447 | 1.912804 | 1.72E-12 | 1.41E-11 |
| LINC00937 | 0.049949 | 0.11834 | 1.24442 | 0.000782 | 0.001153 |
| LINC00954 | 0.2605 | 0.744672 | 1.515321 | 0.00037 | 0.00058 |
| LINC01012 | 0.539051 | 1.452947 | 1.430488 | 3.23E-18 | 9.88E-17 |
| LINC01036 | 0.015565 | 0.291991 | 4.229535 | 0.028677 | 0.030137 |
| LINC01050 | 0.025726 | 0.592077 | 4.524508 | 1.06E-15 | 1.66E-14 |
| LINC01055 | 0.67983 | 0.148246 | -2.19718 | 7.33E-16 | 1.19E-14 |
| LINC01091 | 0.210767 | 0.691612 | 1.71431 | 1.85E-12 | 1.50E-11 |
| LINC01126 | 0.360335 | 0.778935 | 1.112165 | 1.33E-05 | 2.82E-05 |
| LINC01138 | 0.504414 | 1.167775 | 1.211082 | 2.39E-16 | 4.52E-15 |
| LINC01140 | 0.255847 | 0.125171 | -1.03138 | 2.05E-11 | 1.36E-10 |
| LINC01176 | 0.834035 | 1.848168 | 1.147916 | 0.000939 | 0.001358 |
| LINC01191 | 0.05626 | 0.158988 | 1.498728 | 3.74E-05 | 7.20E-05 |
| LINC01197 | 0.219295 | 0.107705 | -1.02579 | 7.77E-12 | 5.71E-11 |
| LINC01276 | 0.054909 | 0.294408 | 2.422693 | 1.78E-11 | 1.20E-10 |
| LINC01285 | 0.090305 | 0.236886 | 1.391323 | 5.02E-07 | 1.39E-06 |
| LINC01303 | 0.016205 | 0.114215 | 2.817276 | 6.60E-10 | 3.23E-09 |
| LINC01338 | 0.0061 | 0.122521 | 4.328078 | 1.79E-08 | 6.59E-08 |
| LINC01352 | 0.615686 | 0.18736 | -1.71638 | 6.72E-16 | 1.11E-14 |
| LINC01355 | 1.26084 | 2.843183 | 1.173122 | 3.05E-08 | 1.08E-07 |
| LINC01359 | 0.132035 | 0.365674 | 1.469638 | 0.014248 | 0.016057 |
| LINC01376 | 0.356337 | 0.750048 | 1.07374 | 9.20E-06 | 2.02E-05 |
| LINC01410 | 0.357498 | 0.815811 | 1.190301 | 7.50E-05 | 0.000136 |
| LINC01424 | 0.233228 | 0.660291 | 1.501361 | 4.53E-10 | 2.27E-09 |
| LINC01429 | 0.00716 | 0.157935 | 4.463133 | 3.33E-07 | 9.52E-07 |
| LINC01473 | 0.468884 | 1.447763 | 1.626523 | 3.53E-10 | 1.82E-09 |
| LINC01537 | 0.516212 | 0.137301 | -1.91063 | 1.22E-16 | 2.53E-15 |
| LINC01545 | 0.144209 | 0.465716 | 1.691287 | 4.57E-06 | 1.06E-05 |
| LINC01572 | 0.136551 | 0.336953 | 1.303106 | 7.28E-17 | 1.52E-15 |
| LINC01607 | 0.0931 | 0.205313 | 1.140973 | 1.36E-06 | 3.47E-06 |
| LINC01622 | 0.042928 | 0.182913 | 2.091168 | 5.13E-07 | 1.42E-06 |
| LINC01655 | 0.026888 | 0.581598 | 4.434968 | 1.39E-10 | 7.73E-10 |
| LINC01705 | 0.115781 | 1.459115 | 3.655618 | 5.06E-19 | 1.94E-17 |
| LINC01719 | 0.212428 | 0.483461 | 1.186427 | 1.47E-07 | 4.46E-07 |
| LINC01748 | 0.143149 | 1.351909 | 3.23941 | 4.31E-14 | 4.82E-13 |
| LINC01762 | 0.11736 | 0.301712 | 1.362224 | 2.57E-05 | 5.15E-05 |
| LINC01811 | 0.046167 | 1.329672 | 4.84805 | 4.41E-21 | 3.37E-19 |
| LINC01829 | 0.346056 | 0.155366 | -1.15533 | 0.00961 | 0.011265 |
| LINC02100 | 0.494493 | 1.331179 | 1.428682 | 5.13E-06 | 1.18E-05 |
| LINC02126 | 0.044386 | 0.166011 | 1.903098 | 0.000525 | 0.000804 |
| LINC02156 | 0.067035 | 0.577279 | 3.106286 | 3.06E-20 | 1.72E-18 |
| LINC02163 | 0.192519 | 1.332715 | 2.791299 | 6.05E-19 | 2.27E-17 |
| LINC02198 | 0.052356 | 0.183353 | 1.808201 | 0.000645 | 0.000968 |
| LINC02254 | 0.0065 | 0.184562 | 4.827519 | 1.51E-08 | 5.65E-08 |
| LINC02257 | 0.032177 | 0.932475 | 4.856975 | 1.83E-15 | 2.76E-14 |
| LINC02334 | 0.031198 | 0.130442 | 2.063898 | 0.000271 | 0.000438 |
| LINC02408 | 0.302579 | 0.125743 | -1.26684 | 8.88E-14 | 9.32E-13 |
| LINC02416 | 0.122972 | 0.416684 | 1.760622 | 5.01E-05 | 9.40E-05 |
| LINC02428 | 0.003263 | 0.267254 | 6.355959 | 1.17E-07 | 3.65E-07 |
| LINC02435 | 0.06997 | 0.24189 | 1.78955 | 0.003408 | 0.004431 |
| LINC02447 | 0.338956 | 0.148178 | -1.19377 | 2.54E-13 | 2.43E-12 |
| LINC02542 | 0.952293 | 2.576346 | 1.435849 | 0.000218 | 0.00036 |
| LINC02561 | 0.150942 | 0.509193 | 1.754218 | 3.22E-05 | 6.36E-05 |
| LINC02595 | 0.162949 | 1.613803 | 3.307973 | 1.62E-23 | 3.72E-21 |
| LINC02603 | 0.088998 | 0.204665 | 1.201423 | 0.003311 | 0.004328 |
| LINC02615 | 0.409391 | 0.915425 | 1.160963 | 2.25E-10 | 1.20E-09 |
| LINC02649 | 0.150167 | 0.534896 | 1.832686 | 5.66E-09 | 2.31E-08 |
| LINC02656 | 0.186723 | 0.524214 | 1.489253 | 0.000265 | 0.000429 |
| LINC02669 | 0.06774 | 0.15489 | 1.193173 | 1.50E-06 | 3.79E-06 |
| LINC02718 | 0.052147 | 0.146746 | 1.492678 | 0.003041 | 0.003998 |
| LINC02795 | 0.063202 | 0.181791 | 1.524235 | 1.41E-05 | 2.97E-05 |
| LINC02883 | 0.120249 | 0.372423 | 1.63092 | 0.000258 | 0.000419 |
| LMCD1-AS1 | 0.052802 | 0.168879 | 1.677317 | 8.75E-14 | 9.23E-13 |
| LMO7-AS1 | 0.218465 | 1.265667 | 2.534423 | 2.59E-15 | 3.83E-14 |
| LNCOC1 | 0.045928 | 0.107323 | 1.224521 | 8.43E-06 | 1.86E-05 |
| LRP1-AS | 0.181272 | 0.417009 | 1.201923 | 3.04E-07 | 8.74E-07 |
| LRRC2-AS1 | 0.045072 | 0.166789 | 1.887714 | 2.10E-08 | 7.63E-08 |
| LUCAT1 | 0.061674 | 0.58417 | 3.243644 | 1.60E-17 | 3.91E-16 |
| LURAP1L-AS1 | 0.103628 | 0.473297 | 2.191333 | 0.000589 | 0.000889 |
| MAL2-AS1 | 0.220156 | 1.040646 | 2.240883 | 2.87E-05 | 5.73E-05 |
| MALAT1 | 50.79079 | 345.1749 | 2.764689 | 1.25E-09 | 5.77E-09 |
| MANEA-DT | 0.278649 | 0.578833 | 1.054698 | 0.000908 | 0.001319 |
| MAP3K5-AS1 | 0.362633 | 1.09576 | 1.595352 | 0.000153 | 0.000261 |
| MBNL1-AS1 | 6.084405 | 1.260004 | -2.27169 | 1.09E-17 | 2.91E-16 |
| MCCC1-AS1 | 0.450347 | 1.081763 | 1.264277 | 2.28E-07 | 6.71E-07 |
| MCM3AP-AS1 | 0.693905 | 1.478285 | 1.091115 | 9.06E-15 | 1.17E-13 |
| MED4-AS1 | 0.11007 | 0.268877 | 1.288527 | 0.007204 | 0.008729 |
| MED8-AS1 | 0.677302 | 1.62802 | 1.265247 | 5.77E-09 | 2.34E-08 |
| MEF2C-AS1 | 0.330814 | 0.084346 | -1.97164 | 4.99E-20 | 2.48E-18 |
| MESTIT1 | 0.111991 | 0.283021 | 1.33753 | 5.90E-09 | 2.39E-08 |
| MIR17HG | 0.838167 | 4.062558 | 2.277078 | 1.14E-17 | 3.00E-16 |
| MIR181A2HG | 0.499405 | 1.883402 | 1.91506 | 2.90E-08 | 1.03E-07 |
| MIR2052HG | 0.005984 | 0.126146 | 4.397905 | 0.000604 | 0.000908 |
| MIR222HG | 2.09904 | 4.630918 | 1.141569 | 1.01E-08 | 3.90E-08 |
| MIR3150BHG | 1.301123 | 0.296257 | -2.13484 | 2.45E-15 | 3.65E-14 |
| MIR31HG | 0.097584 | 1.142663 | 3.549616 | 1.19E-13 | 1.22E-12 |
| MIR378D2HG | 0.041667 | 0.179099 | 2.103761 | 1.63E-06 | 4.09E-06 |
| MIR4435-2HG | 0.257137 | 1.155889 | 2.168392 | 4.13E-23 | 6.93E-21 |
| MPRIP-AS1 | 0.086605 | 0.418449 | 2.272536 | 0.00272 | 0.003606 |
| MRPS30-DT | 1.091742 | 0.436327 | -1.32315 | 4.30E-08 | 1.49E-07 |
| MYOSLID | 0.035642 | 0.156991 | 2.13904 | 6.60E-10 | 3.23E-09 |
| N4BP2L2-IT2 | 0.84957 | 2.542741 | 1.58158 | 4.69E-06 | 1.08E-05 |
| NADK2-AS1 | 0.538651 | 1.32834 | 1.302201 | 0.039248 | 0.040301 |
| NARF-AS1 | 0.12363 | 0.309842 | 1.325503 | 8.95E-05 | 0.000159 |
| NARF-IT1 | 0.545677 | 1.165198 | 1.094456 | 4.27E-05 | 8.12E-05 |
| NCBP2-AS1 | 0.72124 | 1.767277 | 1.292978 | 5.61E-07 | 1.54E-06 |
| NEAT1 | 24.79635 | 86.19648 | 1.797501 | 0.000158 | 0.000268 |
| NKILA | 0.850323 | 3.279748 | 1.947502 | 3.76E-14 | 4.27E-13 |
| NPAS2-AS1 | 0.035028 | 0.115968 | 1.727154 | 0.00123 | 0.001732 |
| NPSR1-AS1 | 0.124923 | 0.526447 | 2.075245 | 1.43E-17 | 3.55E-16 |
| NSMCE1-DT | 0.165326 | 0.34901 | 1.07796 | 8.37E-10 | 3.99E-09 |
| NXT1-AS1 | 0.123937 | 0.419386 | 1.758669 | 3.67E-09 | 1.56E-08 |
| ODF2-AS1 | 0.107505 | 0.451939 | 2.071728 | 0.000546 | 0.000832 |
| OSBPL10-AS1 | 0.044979 | 0.229579 | 2.351667 | 0.001023 | 0.001464 |
| PABPC4-AS1 | 0.339909 | 0.767948 | 1.175858 | 0.001849 | 0.002531 |
| PAN3-AS1 | 1.509019 | 3.389208 | 1.167338 | 3.09E-10 | 1.62E-09 |
| PCAT1 | 0.137598 | 0.929363 | 2.755786 | 1.69E-22 | 2.21E-20 |
| PCBP2-OT1 | 0.105428 | 0.535504 | 2.34464 | 4.86E-09 | 2.03E-08 |
| PDE9A-AS1 | 2.01334 | 0.252783 | -2.99362 | 1.51E-16 | 3.06E-15 |
| PEF1-AS1 | 0.199337 | 0.538248 | 1.433059 | 8.04E-07 | 2.14E-06 |
| PHACTR2-AS1 | 0.143002 | 0.547146 | 1.935888 | 0.034315 | 0.035574 |
| PHC2-AS1 | 0.220453 | 0.517272 | 1.230449 | 0.019524 | 0.021404 |
| PIK3IP1-DT | 0.661879 | 1.543357 | 1.221433 | 3.90E-10 | 1.99E-09 |
| PKP4-AS1 | 0.075551 | 0.270664 | 1.840977 | 8.26E-10 | 3.97E-09 |
| PLCG1-AS1 | 0.154295 | 0.494912 | 1.681477 | 2.10E-10 | 1.13E-09 |
| PLS3-AS1 | 0.0815 | 0.298814 | 1.874376 | 1.11E-07 | 3.47E-07 |
| POC1B-AS1 | 0.573272 | 1.438651 | 1.327424 | 3.95E-17 | 8.73E-16 |
| PPP1R14B-AS1 | 0.31217 | 1.975683 | 2.661948 | 2.28E-20 | 1.35E-18 |
| PRMT5-AS1 | 0.040281 | 0.155661 | 1.950226 | 1.07E-10 | 6.23E-10 |
| PRNCR1 | 0.018693 | 0.146962 | 2.974871 | 2.12E-08 | 7.68E-08 |
| PROSER2-AS1 | 0.079828 | 0.186531 | 1.224453 | 0.000134 | 0.00023 |
| PRR7-AS1 | 0.290886 | 1.846064 | 2.665927 | 5.27E-23 | 8.07E-21 |
| PSPC1-AS2 | 1.106712 | 2.613116 | 1.239492 | 8.79E-06 | 1.94E-05 |
| PTPRG-AS1 | 0.065079 | 0.229586 | 1.818772 | 1.50E-05 | 3.14E-05 |
| PURPL | 0.10597 | 0.845846 | 2.996743 | 0.00214 | 0.002895 |
| PVT1 | 0.406174 | 2.751435 | 2.760013 | 1.64E-24 | 6.94E-22 |
| RASGRP3-AS1 | 0.272558 | 0.6196 | 1.184772 | 0.005114 | 0.0064 |
| RB1-DT | 0.111877 | 0.233091 | 1.058985 | 0.000284 | 0.000457 |
| RDH10-AS1 | 0.108051 | 0.53801 | 2.315917 | 4.75E-07 | 1.32E-06 |
| RERG-IT1 | 0.561672 | 0.174249 | -1.68858 | 2.18E-11 | 1.42E-10 |
| RHOA-IT1 | 0.668856 | 1.368134 | 1.032443 | 0.000829 | 0.001215 |
| RHOQ-AS1 | 0.248565 | 0.942933 | 1.923532 | 0.00741 | 0.008943 |
| RNASEH2B-AS1 | 0.077842 | 0.301343 | 1.952788 | 8.86E-07 | 2.34E-06 |
| RNF139-AS1 | 0.384335 | 1.117352 | 1.539647 | 1.75E-15 | 2.65E-14 |
| RNF157-AS1 | 0.1366 | 0.316445 | 1.211997 | 1.88E-06 | 4.67E-06 |
| RNF213-AS1 | 0.386419 | 0.783681 | 1.020103 | 1.94E-06 | 4.78E-06 |
| RNF32-AS1 | 0.144586 | 0.642854 | 2.152562 | 7.45E-19 | 2.63E-17 |
| RPP38-DT | 0.179677 | 0.423252 | 1.236113 | 1.10E-09 | 5.16E-09 |
| RPS6KA2-IT1 | 0.153844 | 0.345194 | 1.165939 | 0.021068 | 0.022914 |
| RSF1-IT1 | 0.160833 | 0.395194 | 1.297002 | 0.031469 | 0.032865 |
| RUSC1-AS1 | 1.984353 | 5.591484 | 1.494562 | 2.23E-14 | 2.60E-13 |
| SAP30L-AS1 | 0.302191 | 0.609912 | 1.013142 | 8.60E-07 | 2.28E-06 |
| SCARNA9 | 0.411079 | 3.190042 | 2.956088 | 4.71E-07 | 1.31E-06 |
| SCAT2 | 0.759067 | 2.558127 | 1.752788 | 7.34E-14 | 7.98E-13 |
| SDK1-AS1 | 0.345402 | 0.143856 | -1.26366 | 5.92E-10 | 2.93E-09 |
| SEMA6A-AS2 | 0.510879 | 0.11687 | -2.12807 | 1.89E-24 | 6.94E-22 |
| SH3TC2-DT | 0.026444 | 0.168707 | 2.673495 | 2.13E-11 | 1.39E-10 |
| SKAP1-AS1 | 0.08714 | 0.329096 | 1.91711 | 0.001272 | 0.001788 |
| SLC16A1-AS1 | 0.291065 | 1.10526 | 1.924972 | 1.24E-17 | 3.17E-16 |
| SLC2A1-AS1 | 0.118767 | 0.438573 | 1.884677 | 8.34E-10 | 3.99E-09 |
| SLC2A9-AS1 | 1.547353 | 0.493832 | -1.64771 | 3.34E-15 | 4.84E-14 |
| SLC5A4-AS1 | 0.087688 | 0.236865 | 1.433608 | 1.70E-07 | 5.08E-07 |
| SLC7A11-AS1 | 0.041447 | 0.338214 | 3.028615 | 4.17E-09 | 1.76E-08 |
| SLFNL1-AS1 | 0.239556 | 0.524383 | 1.13026 | 7.85E-08 | 2.54E-07 |
| SMG7-AS1 | 0.278028 | 0.619048 | 1.154821 | 1.49E-11 | 1.02E-10 |
| SNHG1 | 13.1289 | 41.74477 | 1.66885 | 1.65E-21 | 1.51E-19 |
| SNHG16 | 6.400516 | 16.53181 | 1.368985 | 1.01E-23 | 3.06E-21 |
| SNHG22 | 0.23036 | 0.550461 | 1.256748 | 5.33E-05 | 9.95E-05 |
| SNHG31 | 0.110635 | 0.294185 | 1.410919 | 0.001141 | 0.001619 |
| SNHG32 | 11.7903 | 40.06899 | 1.764885 | 2.14E-20 | 1.31E-18 |
| SNHG4 | 3.675681 | 13.37006 | 1.862922 | 8.76E-20 | 4.02E-18 |
| SOX9-AS1 | 0.190237 | 0.454452 | 1.25633 | 4.84E-08 | 1.65E-07 |
| SPRY4-AS1 | 0.115784 | 0.511899 | 2.144428 | 2.21E-21 | 1.94E-19 |
| STAM-AS1 | 0.236872 | 0.577242 | 1.285069 | 1.15E-09 | 5.34E-09 |
| STEAP2-AS1 | 0.024537 | 0.166132 | 2.759289 | 7.10E-08 | 2.33E-07 |
| STX18-AS1 | 0.438433 | 0.890458 | 1.022193 | 2.42E-16 | 4.54E-15 |
| SUGCT-AS1 | 0.171295 | 0.540875 | 1.658809 | 7.06E-11 | 4.28E-10 |
| SUGT1-DT | 1.333695 | 4.575389 | 1.778465 | 6.70E-16 | 1.11E-14 |
| TAX1BP1-AS1 | 0.067577 | 0.163573 | 1.275339 | 0.00442 | 0.005592 |
| TFAP2A-AS2 | 0.065602 | 0.546574 | 3.058598 | 2.82E-23 | 5.76E-21 |
| THOC7-AS1 | 0.098626 | 0.467394 | 2.244607 | 0.028225 | 0.029713 |
| THORLNC | 0.169788 | 0.361609 | 1.090694 | 2.32E-07 | 6.82E-07 |
| THUMPD3-AS1 | 3.137844 | 6.741304 | 1.103254 | 1.81E-12 | 1.48E-11 |
| TLX1NB | 0.088133 | 0.671622 | 2.929902 | 4.67E-13 | 4.31E-12 |
| TMCC1-AS1 | 0.386479 | 0.794686 | 1.039995 | 1.22E-07 | 3.80E-07 |
| TMED2-DT | 0.505342 | 1.0513 | 1.056843 | 2.36E-13 | 2.29E-12 |
| TMEM75 | 0.21027 | 0.983171 | 2.225201 | 1.00E-10 | 5.86E-10 |
| TPT1-AS1 | 1.464526 | 2.974675 | 1.022299 | 6.01E-08 | 2.00E-07 |
| TSPOAP1-AS1 | 1.433226 | 7.215061 | 2.331746 | 6.57E-19 | 2.37E-17 |
| TTC3-AS1 | 0.146774 | 0.31315 | 1.093253 | 0.007693 | 0.009243 |
| TTN-AS1 | 0.09824 | 0.204201 | 1.055615 | 0.000513 | 0.000787 |
| UBE2Q1-AS1 | 0.425074 | 1.015364 | 1.25621 | 0.000284 | 0.000457 |
| UBOX5-AS1 | 0.253772 | 0.579715 | 1.191811 | 9.15E-07 | 2.41E-06 |
| USP3-AS1 | 0.206358 | 0.49958 | 1.275567 | 0.000761 | 0.001125 |
| VAC14-AS1 | 0.049105 | 0.297563 | 2.599264 | 4.22E-21 | 3.37E-19 |
| WASHC5-AS1 | 0.262644 | 0.906585 | 1.787333 | 4.14E-06 | 9.68E-06 |
| WNT5A-AS1 | 1.395047 | 2.998526 | 1.10394 | 0.00841 | 0.010006 |
| YEATS2-AS1 | 0.229033 | 0.628488 | 1.456333 | 2.89E-12 | 2.27E-11 |
| Z68871.1 | 0.872142 | 1.845073 | 1.081043 | 2.67E-08 | 9.55E-08 |
| Z69666.1 | 0.150842 | 0.459199 | 1.606084 | 3.12E-05 | 6.18E-05 |
| Z69733.1 | 0.182521 | 0.4459 | 1.288659 | 3.25E-05 | 6.39E-05 |
| Z82217.1 | 0.059102 | 0.292018 | 2.304769 | 2.37E-06 | 5.79E-06 |
| Z82243.1 | 0.635288 | 2.582488 | 2.023278 | 5.90E-12 | 4.41E-11 |
| Z83843.1 | 2.221821 | 8.885941 | 1.999782 | 9.50E-06 | 2.08E-05 |
| Z84485.1 | 0.342281 | 1.099996 | 1.684244 | 1.70E-13 | 1.68E-12 |
| Z94721.1 | 0.75666 | 1.785404 | 1.238533 | 1.19E-07 | 3.71E-07 |
| Z94721.2 | 0.041547 | 0.121247 | 1.545152 | 0.00072 | 0.001069 |
| Z94721.3 | 0.341709 | 0.749911 | 1.13395 | 0.007238 | 0.008753 |
| Z95331.1 | 0.068519 | 0.193106 | 1.494822 | 1.15E-10 | 6.57E-10 |
| Z97192.1 | 0.019491 | 0.158753 | 3.025923 | 0.003386 | 0.004408 |
| Z97200.1 | 0.026514 | 0.40748 | 3.941904 | 3.00E-09 | 1.29E-08 |
| Z99289.2 | 0.019619 | 0.287419 | 3.87286 | 5.54E-06 | 1.27E-05 |
| ZDHHC20-IT1 | 0.385016 | 1.735111 | 2.172036 | 1.16E-11 | 8.35E-11 |
| ZFHX2-AS1 | 0.133702 | 0.325384 | 1.28312 | 7.59E-10 | 3.67E-09 |
| ZFPM2-AS1 | 0.079823 | 0.466512 | 2.547034 | 2.40E-10 | 1.28E-09 |
| ZKSCAN2-DT | 1.304391 | 3.153537 | 1.273595 | 5.01E-11 | 3.13E-10 |
| ZMIZ1-AS1 | 0.28253 | 0.639403 | 1.178321 | 3.04E-09 | 1.30E-08 |
| ZNF252P-AS1 | 0.280584 | 0.675217 | 1.26692 | 1.07E-08 | 4.09E-08 |
| ZNF337-AS1 | 0.587544 | 1.333407 | 1.182348 | 1.31E-08 | 4.97E-08 |
| ZNF346-IT1 | 0.410223 | 1.067615 | 1.37991 | 2.97E-06 | 7.15E-06 |
| ZNF433-AS1 | 0.72447 | 1.466832 | 1.017706 | 9.21E-16 | 1.46E-14 |
| ZNF451-AS1 | 0.204698 | 0.492434 | 1.266435 | 0.000883 | 0.001286 |
| ZNF775-AS1 | 0.41003 | 1.320675 | 1.687473 | 7.07E-07 | 1.90E-06 |
| ZNF8-ERVK3-1 | 0.173244 | 0.360574 | 1.057489 | 1.14E-10 | 6.53E-10 |
| ZNRF3-AS1 | 0.049512 | 0.312774 | 2.659281 | 3.75E-06 | 8.87E-06 |

**Supplementary Table S4.** LncRNAs in the results of LASSO regression analysis.

| id |
| --- |
| AC005670.1 |
| AC006449.3 |
| AC007216.3 |
| AC007608.3 |
| AC009404.1 |
| AC010463.3 |
| AC011997.1 |
| AC013652.1 |
| AC026356.2 |
| AC064836.3 |
| AC083805.2 |
| AC083900.1 |
| AC084117.1 |
| AC093732.1 |
| AC245884.8 |
| AL034550.3 |
| AL121992.3 |
| AL161663.1 |
| AL161729.4 |
| FIRRE |
| LINC01138 |
| LINC01285 |
| LINC02257 |
| MIR31HG |
| RASGRP3-AS1 |
| RPP38-DT |

**Supplementary Table S5.** The results of the multivariate Cox regression model analysis.

| id | coef |
| --- | --- |
| AC006449.3 | 1.079662 |
| AC007216.3 | -2.73834 |
| AC007608.3 | 1.186543 |
| AC011997.1 | 1.699957 |
| AC026356.2 | 0.868694 |
| AC064836.3 | 0.777473 |
| AC083805.2 | -0.78117 |
| AC083900.1 | 0.436184 |
| AL034550.3 | 0.853048 |
| AL121992.3 | -0.77061 |
| AL161663.1 | -2.01544 |
| FIRRE | 0.44101 |
| LINC02257 | 0.519502 |
| MIR31HG | 0.605015 |
| RPP38-DT | 0.915453 |

**Supplementary Table S6.** The risk score and risk group of TCGA samples.

| id | riskScore | risk |
| --- | --- | --- |
| TCGA-AA-3818 | 5.455779 | high |
| TCGA-AA-3543 | 1.159464 | high |
| TCGA-AA-A00R | 1.450201 | high |
| TCGA-AA-A01S | 0.4708 | low |
| TCGA-AA-3496 | 4.424339 | high |
| TCGA-AA-3494 | 0.551999 | low |
| TCGA-AG-A020 | 0.062084 | low |
| TCGA-AG-3883 | 9.425115 | high |
| TCGA-AG-3896 | 8.201923 | high |
| TCGA-AZ-4323 | 128.5928 | high |
| TCGA-F4-6704 | 2.956798 | high |
| TCGA-AG-A026 | 9.472633 | high |
| TCGA-AA-3966 | 15.41931 | high |
| TCGA-AA-3930 | 70.6423 | high |
| TCGA-AG-3574 | 11.41588 | high |
| TCGA-EF-5830 | 0.436229 | low |
| TCGA-AG-4021 | 126.8401 | high |
| TCGA-4N-A93T | 1.898718 | high |
| TCGA-D5-6537 | 2.329761 | high |
| TCGA-AA-A02J | 3.607296 | high |
| TCGA-AZ-4616 | 7.238892 | high |
| TCGA-AZ-6605 | 0.929875 | low |
| TCGA-AZ-4614 | 26.58461 | high |
| TCGA-AD-6899 | 17.51287 | high |
| TCGA-AG-A00C | 2.093012 | high |
| TCGA-CI-6619 | 0.696536 | low |
| TCGA-F4-6570 | 11.24788 | high |
| TCGA-AA-3511 | 0.327201 | low |
| TCGA-AA-3663 | 0.888781 | low |
| TCGA-D5-6898 | 0.857456 | low |
| TCGA-DM-A280 | 0.150248 | low |
| TCGA-AG-3726 | 1.806079 | high |
| TCGA-F4-6459 | 8.05132 | high |
| TCGA-EI-6882 | 0.473861 | low |
| TCGA-AA-3519 | 0.05106 | low |
| TCGA-D5-6927 | 0.159931 | low |
| TCGA-NH-A6GA | 68.58562 | high |
| TCGA-CM-6679 | 0.52709 | low |
| TCGA-AA-3811 | 13.31495 | high |
| TCGA-CA-6718 | 37.62581 | high |
| TCGA-AD-6964 | 9.165208 | high |
| TCGA-NH-A50U | 37.35757 | high |
| TCGA-DC-6158 | 15.53426 | high |
| TCGA-CM-6172 | 15.73013 | high |
| TCGA-CM-6678 | 0.173 | low |
| TCGA-AA-3680 | 9.828139 | high |
| TCGA-CM-6677 | 0.454364 | low |
| TCGA-F4-6461 | 5.714304 | high |
| TCGA-D5-6932 | 12.10052 | high |
| TCGA-EI-6883 | 0.748881 | low |
| TCGA-D5-6928 | 19.27984 | high |
| TCGA-AZ-6606 | 5.074667 | high |
| TCGA-EI-7002 | 0.748951 | low |
| TCGA-CM-6162 | 0.417915 | low |
| TCGA-D5-6931 | 3.745135 | high |
| TCGA-CM-6680 | 4.446488 | high |
| TCGA-AD-5900 | 0.661761 | low |
| TCGA-CA-6716 | 1.736311 | high |
| TCGA-CA-5256 | 0.029844 | low |
| TCGA-F5-6864 | 3.219537 | high |
| TCGA-CA-5797 | 1.203393 | high |
| TCGA-CA-6715 | 0.469574 | low |
| TCGA-CM-6168 | 0.679485 | low |
| TCGA-AA-3939 | 5.27385 | high |
| TCGA-CM-4752 | 0.640885 | low |
| TCGA-CM-6169 | 7.174162 | high |
| TCGA-AG-3892 | 0.063766 | low |
| TCGA-CM-6675 | 2.149676 | high |
| TCGA-AA-3973 | 2.062424 | high |
| TCGA-F4-6809 | 10.32017 | high |
| TCGA-DM-A1D7 | 0.990363 | low |
| TCGA-A6-2682 | 1.341848 | high |
| TCGA-CL-4957 | 6.062102 | high |
| TCGA-AA-A02K | 47.53126 | high |
| TCGA-AA-3667 | 0.174983 | low |
| TCGA-CM-6171 | 0.536124 | low |
| TCGA-AG-4005 | 0.777898 | low |
| TCGA-D5-6924 | 0.686434 | low |
| TCGA-CA-6719 | 3.415918 | high |
| TCGA-AU-3779 | 2.660561 | high |
| TCGA-A6-4105 | 8.640404 | high |
| TCGA-AA-3844 | 2.558948 | high |
| TCGA-CM-6167 | 2.422057 | high |
| TCGA-AA-A017 | 0.085085 | low |
| TCGA-CM-5861 | 0.812402 | low |
| TCGA-D5-6535 | 0.998649 | low |
| TCGA-D5-6541 | 0.215698 | low |
| TCGA-NH-A6GB | 0.278474 | low |
| TCGA-EI-6511 | 2.355104 | high |
| TCGA-AA-3833 | 0.170183 | low |
| TCGA-AG-A014 | 0.148446 | low |
| TCGA-CM-6165 | 0.459088 | low |
| TCGA-D5-6540 | 2.960265 | high |
| TCGA-A6-A565 | 3.9722 | high |
| TCGA-EI-6514 | 0.490008 | low |
| TCGA-EI-6513 | 7.571963 | high |
| TCGA-AD-A5EK | 1.98087 | high |
| TCGA-NH-A8F8 | 5.575368 | high |
| TCGA-EI-6509 | 0.885614 | low |
| TCGA-CK-6751 | 1.073423 | high |
| TCGA-AG-3890 | 0.51943 | low |
| TCGA-D5-6538 | 1.801569 | high |
| TCGA-AY-A54L | 0.258927 | low |
| TCGA-EI-6917 | 11.4264 | high |
| TCGA-AH-6549 | 0.294529 | low |
| TCGA-CK-4947 | 0.016143 | low |
| TCGA-D5-6531 | 5.24153 | high |
| TCGA-D5-6536 | 6.69449 | high |
| TCGA-AA-3554 | 1.29505 | high |
| TCGA-AA-3831 | 0.069778 | low |
| TCGA-AA-A00J | 1.394461 | high |
| TCGA-NH-A50T | 4.854845 | high |
| TCGA-DM-A1D6 | 19.14291 | high |
| TCGA-AY-A8YK | 0.678366 | low |
| TCGA-AA-A00D | 12.02386 | high |
| TCGA-AA-3688 | 0.069731 | low |
| TCGA-AA-3715 | 5.246044 | high |
| TCGA-AA-3713 | 2.810586 | high |
| TCGA-AA-3526 | 0.176302 | low |
| TCGA-AY-A71X | 0.301444 | low |
| TCGA-A6-6781 | 0.003327 | low |
| TCGA-EI-6507 | 6.249001 | high |
| TCGA-AA-3562 | 0.467551 | low |
| TCGA-AA-3560 | 0.863042 | low |
| TCGA-CM-4744 | 2.019581 | high |
| TCGA-A6-6780 | 0.002358 | low |
| TCGA-D5-6529 | 0.978839 | low |
| TCGA-D5-6530 | 0.291557 | low |
| TCGA-EI-6506 | 0.66317 | low |
| TCGA-A6-6650 | 0.000574 | low |
| TCGA-AA-3549 | 0.086777 | low |
| TCGA-AD-6548 | 0.208685 | low |
| TCGA-AY-6197 | 3.242091 | high |
| TCGA-AA-A00Z | 0.024018 | low |
| TCGA-AA-3968 | 2.822793 | high |
| TCGA-AA-A02R | 12.8318 | high |
| TCGA-A6-5665 | 0.000493 | low |
| TCGA-CM-5348 | 5.435575 | high |
| TCGA-AA-3556 | 5.43202 | high |
| TCGA-CM-4743 | 0.991459 | low |
| TCGA-A6-6654 | 5.894601 | high |
| TCGA-AA-3553 | 1.326279 | high |
| TCGA-AA-3979 | 0.268624 | low |
| TCGA-AG-3584 | 12.11697 | high |
| TCGA-AA-3520 | 2.632361 | high |
| TCGA-A6-6140 | 0.335786 | low |
| TCGA-DM-A0XD | 37.80794 | high |
| TCGA-A6-A566 | 17.05778 | high |
| TCGA-AA-3977 | 0.032286 | low |
| TCGA-AA-3819 | 4.045061 | high |
| TCGA-D5-6533 | 1.302314 | high |
| TCGA-CM-4748 | 0.329593 | low |
| TCGA-G4-6588 | 1.241448 | high |
| TCGA-G4-6320 | 0.160058 | low |
| TCGA-CK-6747 | 0.077821 | low |
| TCGA-AA-3982 | 2.103144 | high |
| TCGA-G4-6294 | 0.202703 | low |
| TCGA-AA-3534 | 0.0329 | low |
| TCGA-AA-3532 | 0.07112 | low |
| TCGA-CM-5341 | 0.532948 | low |
| TCGA-A6-5660 | 0.098375 | low |
| TCGA-QG-A5YW | 6.728992 | high |
| TCGA-AF-4110 | 1.734121 | high |
| TCGA-AG-3728 | 0.54286 | low |
| TCGA-AA-3861 | 0.718358 | low |
| TCGA-AA-A01I | 0.020395 | low |
| TCGA-AA-3877 | 0.76423 | low |
| TCGA-DC-5869 | 0.092839 | low |
| TCGA-AA-3858 | 0.467469 | low |
| TCGA-AA-3860 | 3.533665 | high |
| TCGA-A6-5657 | 1.960984 | high |
| TCGA-F4-6460 | 2.095651 | high |
| TCGA-AA-A01F | 0.595885 | low |
| TCGA-A6-5666 | 5.8236 | high |
| TCGA-A6-3809 | 0.002294 | low |
| TCGA-AZ-4615 | 4.368211 | high |
| TCGA-CI-6620 | 0.586979 | low |
| TCGA-AA-3975 | 0.132361 | low |
| TCGA-F4-6805 | 5.860504 | high |
| TCGA-A6-3807 | 0.475773 | low |
| TCGA-AA-A010 | 1.793528 | high |
| TCGA-AA-3502 | 0.648006 | low |
| TCGA-AA-3812 | 0.885548 | low |
| TCGA-A6-2680 | 0.0184 | low |
| TCGA-F4-6856 | 0.549667 | low |
| TCGA-AA-3854 | 0.602517 | low |
| TCGA-AA-3970 | 0.237764 | low |
| TCGA-AG-A015 | 0.123993 | low |
| TCGA-A6-3810 | 0.028675 | low |
| TCGA-AA-3841 | 0.727216 | low |
| TCGA-AG-3887 | 0.031639 | low |
| TCGA-A6-2686 | 15.21169 | high |
| TCGA-AA-3842 | 0.082114 | low |
| TCGA-AA-A01Z | 0.17756 | low |
| TCGA-AA-3495 | 1.104863 | high |
| TCGA-AA-3522 | 2.972739 | high |
| TCGA-A6-2684 | 0.001613 | low |
| TCGA-A6-2685 | 0.528295 | low |
| TCGA-AF-3911 | 0.983004 | low |
| TCGA-AA-A00L | 0.12598 | low |
| TCGA-AA-A01P | 4.631921 | high |
| TCGA-DM-A0XF | 9.188134 | high |
| TCGA-RU-A8FL | 1.056441 | low |
| TCGA-AG-A02G | 4.019595 | high |
| TCGA-5M-AATE | 2.204811 | high |
| TCGA-AA-A02F | 3.813171 | high |
| TCGA-AA-A03J | 0.151272 | low |
| TCGA-AG-A02X | 0.734065 | low |
| TCGA-A6-2678 | 0.326286 | low |
| TCGA-F5-6571 | 0.270274 | low |
| TCGA-F4-6807 | 2.774085 | high |
| TCGA-A6-2674 | 0.152027 | low |
| TCGA-DM-A1DB | 20.54618 | high |
| TCGA-G4-6306 | 1.25202 | high |
| TCGA-A6-2681 | 0.206489 | low |
| TCGA-AG-4022 | 1.841561 | high |
| TCGA-A6-2672 | 0.000248 | low |
| TCGA-AA-3678 | 0.534162 | low |
| TCGA-AA-3675 | 0.267783 | low |
| TCGA-AG-3898 | 0.011109 | low |
| TCGA-AG-A025 | 0.755261 | low |
| TCGA-AA-3673 | 0.387112 | low |
| TCGA-AA-3972 | 1.263673 | high |
| TCGA-CK-5913 | 0.227888 | low |
| TCGA-DY-A1DG | 12.87192 | high |
| TCGA-AG-A023 | 14.04473 | high |
| TCGA-G4-6304 | 0.099406 | low |
| TCGA-D5-5538 | 2.289096 | high |
| TCGA-G4-6307 | 0.599124 | low |
| TCGA-D5-5541 | 6.888863 | high |
| TCGA-DM-A28G | 1.160032 | high |
| TCGA-AA-3655 | 0.628439 | low |
| TCGA-A6-A567 | 6.510579 | high |
| TCGA-G4-6310 | 0.642137 | low |
| TCGA-AA-3510 | 0.647728 | low |
| TCGA-G4-6303 | 11.54596 | high |
| TCGA-CK-4951 | 5.74178 | high |
| TCGA-G4-6627 | 0.384923 | low |
| TCGA-G4-6299 | 10.69302 | high |
| TCGA-G4-6628 | 1.810402 | high |
| TCGA-G4-6297 | 2.908549 | high |
| TCGA-G4-6625 | 0.157122 | low |
| TCGA-DM-A1D4 | 1.354727 | high |
| TCGA-DM-A28M | 0.517865 | low |
| TCGA-AG-A036 | 0.220963 | low |
| TCGA-DM-A28E | 0.189234 | low |
| TCGA-DY-A1DE | 1.330182 | high |
| TCGA-G4-6293 | 0.761846 | low |
| TCGA-DM-A1HB | 1.359011 | high |
| TCGA-DM-A282 | 2.658414 | high |
| TCGA-CK-4948 | 0.38807 | low |
| TCGA-AA-3856 | 1.783749 | high |
| TCGA-AG-3878 | 0.27215 | low |
| TCGA-AA-3518 | 1.761365 | high |
| TCGA-AA-3821 | 0.035736 | low |
| TCGA-AA-A01V | 0.024303 | low |
| TCGA-AA-A01Q | 0.180469 | low |
| TCGA-AA-3514 | 0.301869 | low |
| TCGA-AG-A01J | 0.155746 | low |
| TCGA-5M-AAT4 | 0.953924 | low |
| TCGA-AZ-6608 | 2.185508 | high |
| TCGA-AA-3952 | 4.448015 | high |
| TCGA-AA-A02H | 2.003845 | high |
| TCGA-CK-6748 | 1.426146 | high |
| TCGA-AA-3666 | 0.183086 | low |
| TCGA-AA-A02E | 7.379173 | high |
| TCGA-AZ-6607 | 417.5337 | high |
| TCGA-AA-A00N | 2.474286 | high |
| TCGA-EF-5831 | 0.686307 | low |
| TCGA-CM-5862 | 3.273514 | high |
| TCGA-AA-3696 | 0.022177 | low |
| TCGA-DM-A285 | 9.990663 | high |
| TCGA-AA-3681 | 6.830592 | high |
| TCGA-AA-3662 | 1.592651 | high |
| TCGA-AZ-6599 | 3.558053 | high |
| TCGA-AA-3489 | 1.060559 | high |
| TCGA-DM-A1DA | 1.899747 | high |
| TCGA-AA-3980 | 0.393725 | low |
| TCGA-AA-3989 | 0.569193 | low |
| TCGA-G4-6295 | 5.332029 | high |
| TCGA-A6-6141 | 0.448798 | low |
| TCGA-D5-6926 | 0.835195 | low |
| TCGA-AG-A016 | 0.524192 | low |
| TCGA-5M-AAT6 | 6.777913 | high |
| TCGA-A6-A5ZU | 4.35511 | high |
| TCGA-AA-3848 | 0.020026 | low |
| TCGA-D5-6922 | 16.57951 | high |
| TCGA-D5-7000 | 6.544893 | high |
| TCGA-EI-6884 | 0.905731 | low |
| TCGA-CM-6676 | 0.534209 | low |
| TCGA-F5-6863 | 57.66065 | high |
| TCGA-T9-A92H | 0.909561 | low |
| TCGA-AA-A01G | 1.07126 | high |
| TCGA-AG-3575 | 351.9208 | high |
| TCGA-AZ-6600 | 3.087122 | high |
| TCGA-CA-5255 | 6.330993 | high |
| TCGA-CA-5796 | 0.160976 | low |
| TCGA-D5-6920 | 0.135327 | low |
| TCGA-D5-6923 | 0.835704 | low |
| TCGA-D5-6539 | 0.122244 | low |
| TCGA-DM-A1D8 | 12.48277 | high |
| TCGA-DT-5265 | 62.05377 | high |
| TCGA-4T-AA8H | 0.981126 | low |
| TCGA-CA-5254 | 0.610703 | low |
| TCGA-CA-6717 | 0.262747 | low |
| TCGA-NH-A6GC | 0.636681 | low |
| TCGA-CM-6674 | 1.931428 | high |
| TCGA-AA-3542 | 0.532401 | low |
| TCGA-AA-3516 | 0.692694 | low |
| TCGA-AA-3552 | 0.048069 | low |
| TCGA-D5-6930 | 1.975689 | high |
| TCGA-D5-6929 | 3.308408 | high |
| TCGA-AF-2692 | 0.040525 | low |
| TCGA-EI-6885 | 3.388981 | high |
| TCGA-G4-6323 | 0.300269 | low |
| TCGA-CI-6621 | 0.722372 | low |
| TCGA-AA-A004 | 0.499704 | low |
| TCGA-AA-3561 | 0.781331 | low |
| TCGA-AG-A008 | 0.531747 | low |
| TCGA-AA-3544 | 0.312024 | low |
| TCGA-AG-3894 | 0.148948 | low |
| TCGA-CM-6163 | 0.323799 | low |
| TCGA-DM-A288 | 0.412963 | low |
| TCGA-AA-A00W | 1.61714 | high |
| TCGA-CM-6170 | 0.808572 | low |
| TCGA-CM-5863 | 0.582936 | low |
| TCGA-AA-3679 | 0.319371 | low |
| TCGA-AA-A01C | 1.12712 | high |
| TCGA-CM-6161 | 3.002108 | high |
| TCGA-CM-5864 | 0.867703 | low |
| TCGA-AD-6888 | 2.865193 | high |
| TCGA-3L-AA1B | 1.400138 | high |
| TCGA-CK-4952 | 0.522118 | low |
| TCGA-AG-3608 | 0.025973 | low |
| TCGA-AA-3971 | 0.325518 | low |
| TCGA-AY-4070 | 6.831922 | high |
| TCGA-EI-6881 | 0.545796 | low |
| TCGA-A6-2683 | 0.110867 | low |
| TCGA-CM-5868 | 1.409059 | high |
| TCGA-AA-A00U | 0.517348 | low |
| TCGA-AA-3846 | 1.009626 | low |
| TCGA-AA-3866 | 0.420866 | low |
| TCGA-AF-2690 | 2.498445 | high |
| TCGA-EI-6512 | 1.455512 | high |
| TCGA-AY-6386 | 0.181158 | low |
| TCGA-NH-A8F7 | 0.644074 | low |
| TCGA-AY-A69D | 0.552445 | low |
| TCGA-AG-3885 | 3.311944 | high |
| TCGA-AA-A00K | 0.136176 | low |
| TCGA-AA-3875 | 1.292887 | high |
| TCGA-D5-6532 | 1.947068 | high |
| TCGA-G5-6233 | 0.707781 | low |
| TCGA-EI-6510 | 1.813786 | high |
| TCGA-AA-3530 | 7.60004 | high |
| TCGA-AA-3986 | 1.109751 | high |
| TCGA-NH-A50V | 0.27937 | low |
| TCGA-D5-5539 | 0.451373 | low |
| TCGA-AG-3612 | 0.36437 | low |
| TCGA-A6-6782 | 1.134261 | high |
| TCGA-EI-6508 | 0.268732 | low |
| TCGA-AA-3955 | 0.221957 | low |
| TCGA-AG-A002 | 0.450796 | low |
| TCGA-CK-5916 | 1.236392 | high |
| TCGA-BM-6198 | 3.121589 | high |
| TCGA-A6-6651 | 2.589884 | high |
| TCGA-QL-A97D | 0.376093 | low |
| TCGA-CK-5914 | 0.977172 | low |
| TCGA-CM-6166 | 1.48038 | high |
| TCGA-CM-5344 | 4.645358 | high |
| TCGA-G4-6321 | 0.490299 | low |
| TCGA-A6-5664 | 0.13548 | low |
| TCGA-AD-6901 | 15.21315 | high |
| TCGA-A6-6138 | 1.314785 | high |
| TCGA-AG-A00Y | 0.459296 | low |
| TCGA-A6-5662 | 19.07287 | high |
| TCGA-AA-3941 | 1.073032 | high |
| TCGA-AA-3950 | 3.738617 | high |
| TCGA-AA-3867 | 0.968785 | low |
| TCGA-A6-6649 | 4.170098 | high |
| TCGA-A6-2677 | 0.016194 | low |
| TCGA-A6-6653 | 0.539943 | low |
| TCGA-AD-6890 | 0.390136 | low |
| TCGA-A6-6652 | 1.619062 | high |
| TCGA-CM-4747 | 1.907964 | high |
| TCGA-AD-6895 | 0.776999 | low |
| TCGA-A6-6142 | 1.989057 | high |
| TCGA-A6-6648 | 0.775877 | low |
| TCGA-AG-A00H | 0.343268 | low |
| TCGA-AA-3538 | 0.038761 | low |
| TCGA-AA-A01X | 0.216411 | low |
| TCGA-AA-3949 | 0.798714 | low |
| TCGA-G4-6322 | 0.834372 | low |
| TCGA-DM-A28A | 62.28573 | high |
| TCGA-AD-6965 | 1.467288 | high |
| TCGA-AA-3710 | 20.92382 | high |
| TCGA-AA-A00O | 0.722406 | low |
| TCGA-AA-3994 | 0.416075 | low |
| TCGA-AA-3869 | 2.593962 | high |
| TCGA-CM-4751 | 11.55112 | high |
| TCGA-A6-6137 | 0.84939 | low |
| TCGA-AU-6004 | 0.418713 | low |
| TCGA-AD-6963 | 1.973675 | high |
| TCGA-CM-6164 | 4.816961 | high |
| TCGA-A6-5667 | 3.169926 | high |
| TCGA-AZ-6603 | 1.377516 | high |
| TCGA-AA-3555 | 1.403957 | high |
| TCGA-AA-3870 | 2.031835 | high |
| TCGA-AA-A00E | 0.250137 | low |
| TCGA-AA-3862 | 0.439506 | low |
| TCGA-CM-5349 | 3.39908 | high |
| TCGA-A6-5659 | 0.001417 | low |
| TCGA-AA-A01K | 0.270549 | low |
| TCGA-AG-A01N | 1.508827 | high |
| TCGA-QG-A5Z2 | 2.71343 | high |
| TCGA-CM-5860 | 1.664286 | high |
| TCGA-AA-3855 | 0.682689 | low |
| TCGA-A6-4107 | 0.116168 | low |
| TCGA-DY-A1H8 | 0.291833 | low |
| TCGA-A6-5656 | 0.140497 | low |
| TCGA-QG-A5YX | 1.900421 | high |
| TCGA-AY-5543 | 0.325641 | low |
| TCGA-AA-3947 | 25.98241 | high |
| TCGA-AA-3815 | 0.36334 | low |
| TCGA-AA-A01T | 0.024861 | low |
| TCGA-AA-3851 | 8.131743 | high |
| TCGA-A6-3808 | 0.853916 | low |
| TCGA-A6-5661 | 0.037547 | low |
| TCGA-F4-6808 | 0.95497 | low |
| TCGA-AA-3548 | 2.377044 | high |
| TCGA-AA-3956 | 0.956995 | low |
| TCGA-AA-A00F | 0.217815 | low |
| TCGA-AA-3531 | 0.388685 | low |
| TCGA-AA-A01R | 1.958536 | high |
| TCGA-F4-6569 | 4.663872 | high |
| TCGA-F4-6463 | 9.471977 | high |
| TCGA-G4-6586 | 0.640387 | low |
| TCGA-G4-6314 | 11.85606 | high |
| TCGA-DM-A28F | 1.288619 | high |
| TCGA-AA-3692 | 1.10228 | high |
| TCGA-G4-6317 | 0.88971 | low |
| TCGA-AA-3524 | 0.23268 | low |
| TCGA-CM-4746 | 2.669056 | high |
| TCGA-AG-3731 | 4.241708 | high |
| TCGA-AG-A011 | 0.075407 | low |
| TCGA-AA-3685 | 1.053733 | low |
| TCGA-F5-6814 | 1.156696 | high |
| TCGA-AA-A00A | 0.239438 | low |
| TCGA-AG-A032 | 0.634901 | low |
| TCGA-F5-6861 | 0.013537 | low |
| TCGA-AA-3837 | 0.5391 | low |
| TCGA-AA-3517 | 0.586598 | low |
| TCGA-AA-A024 | 0.359342 | low |
| TCGA-G4-6311 | 6.294997 | high |
| TCGA-AA-A02Y | 0.30266 | low |
| TCGA-AA-A02W | 9.590855 | high |
| TCGA-F4-6806 | 0.933817 | low |
| TCGA-AA-A00Q | 2.258077 | high |
| TCGA-QG-A5YV | 1.430524 | high |
| TCGA-A6-2676 | 0.574283 | low |
| TCGA-D5-6534 | 6.016217 | high |
| TCGA-A6-2675 | 0.382203 | low |
| TCGA-A6-2671 | 0.171935 | low |
| TCGA-CI-6622 | 0.220264 | low |
| TCGA-A6-2679 | 0.013397 | low |
| TCGA-D5-5537 | 6.745512 | high |
| TCGA-AG-3587 | 3.482662 | high |
| TCGA-F4-6855 | 0.751464 | low |
| TCGA-CI-6623 | 0.193302 | low |
| TCGA-F4-6703 | 2.900598 | high |
| TCGA-CI-6624 | 0.806229 | low |
| TCGA-CK-5912 | 0.409192 | low |
| TCGA-AZ-6598 | 6.138348 | high |
| TCGA-AA-A029 | 0.048238 | low |
| TCGA-AA-3864 | 0.454745 | low |
| TCGA-AA-3664 | 0.037661 | low |
| TCGA-D5-5540 | 1.598057 | high |
| TCGA-A6-A56B | 0.641508 | low |
| TCGA-AA-3506 | 0.451832 | low |
| TCGA-AZ-4315 | 0.212715 | low |
| TCGA-SS-A7HO | 183.1079 | high |
| TCGA-G4-6315 | 0.741079 | low |
| TCGA-AG-A02N | 0.188477 | low |
| TCGA-AZ-5403 | 1.166098 | high |
| TCGA-AA-3509 | 0.223483 | low |
| TCGA-WS-AB45 | 2.368016 | high |
| TCGA-AZ-4313 | 0.75306 | low |
| TCGA-AA-3660 | 0.529931 | low |
| TCGA-DM-A28C | 0.418368 | low |
| TCGA-AD-6889 | 2.526466 | high |
| TCGA-AA-3697 | 1.041739 | low |
| TCGA-CK-4950 | 2.91044 | high |
| TCGA-DM-A1HA | 110.4125 | high |
| TCGA-G4-6309 | 0.166269 | low |
| TCGA-AZ-5407 | 0.531751 | low |
| TCGA-DM-A28K | 0.613663 | low |
| TCGA-AZ-6601 | 3.034588 | high |
| TCGA-AZ-4308 | 1.91143 | high |
| TCGA-DM-A28H | 0.110318 | low |
| TCGA-DM-A0X9 | 3.987791 | high |
| TCGA-DM-A1D0 | 1.135971 | high |
| TCGA-DM-A1D9 | 1.884918 | high |

**Supplementary Table S7.** The risk score and risk group of GSE39582 samples.

id riskScore risk

GSM1875897 0.945151848 high

GSM1875898 0.820425188 low

GSM1875899 0.701422192 low

GSM1875900 1.012014669 high

GSM1875901 1.184616804 high

GSM1875902 0.72234785 low

GSM1875903 1.469101823 high

GSM1875904 0.863197207 low

GSM1875905 0.851755493 low

GSM1875906 0.81213006 low

GSM1875907 1.074368811 high

GSM1875908 0.959831653 high

GSM1875909 1.386663334 high

GSM1875910 1.031755043 high

GSM1875911 1.589263391 high

GSM1875912 1.193161304 high

GSM1875913 0.851755493 low

GSM1875914 1.143036256 high

GSM1875915 1.000025737 high

GSM1875916 1.095731557 high

GSM1875917 1.138663112 high

GSM1875918 0.791259515 low

GSM1875919 0.933887868 low

GSM1875920 0.897952285 low

GSM1875921 0.94907195 high

GSM1875922 0.898710036 low

GSM1875923 0.908077269 low

GSM1875924 0.862994598 low

GSM1875925 1.198884026 high

GSM1875926 1.147868832 high

GSM1875927 1.1536836 high

GSM1875928 0.911390324 low

GSM1875929 1.022109953 high

GSM1875930 1.394036409 high

GSM1875931 0.726813352 low

GSM1875932 0.900613817 low

GSM1875933 0.769413477 low

GSM1875934 1.215572311 high

GSM1875935 0.771947387 low

GSM1875936 0.707514597 low

GSM1875937 0.811705663 low

GSM1875938 0.841054753 low

GSM1875939 0.822696414 low

GSM1875940 0.982704438 high

GSM1875941 0.864020437 low

GSM1875942 1.246063014 high

GSM1875943 0.87745924 low

GSM1875944 1.215283353 high

GSM1875945 2.112834083 high

GSM1875946 0.843212808 low

GSM1875947 1.055970972 high

GSM1875948 1.120090343 high

GSM1875949 1.303974475 high

GSM1875950 1.189360717 high

GSM1875951 1.24528035 high

GSM1875952 1.024326968 high

GSM1875953 1.20319456 high

GSM1875954 1.272721211 high

GSM1875955 1.192471465 high

GSM1875956 0.891411478 low

GSM1875957 0.815656943 low

GSM1875958 1.040161109 high

GSM1875959 1.456948716 high

GSM1875960 0.828399209 low

GSM1875961 1.095960353 high

GSM1875962 0.825129729 low

GSM1875963 0.896065184 low

GSM1875964 1.154448429 high

GSM1875965 0.888007349 low

GSM1875966 0.976432548 high

GSM1875967 0.888681425 low

GSM1875968 1.136197631 high

GSM1875969 0.79881361 low

GSM1875970 1.016049857 high

GSM1875971 0.954735723 high

GSM1875972 0.801843614 low

GSM1875973 0.905612696 low

GSM1875974 1.12844299 high

GSM1875975 0.950807446 high

GSM1875976 1.047516082 high

GSM1875977 0.827714505 low

GSM1875978 1.943969288 high

GSM1875979 0.825471883 low

GSM1875980 1.27312821 high

GSM1875981 0.876912097 low

GSM1875982 1.075082891 high

GSM1875983 0.878548687 low

GSM1875984 1.052912198 high

GSM1875985 0.968142805 high

GSM1875986 2.72568261 high

GSM1875987 0.80162836 low

GSM1875988 0.780902728 low

GSM1875989 0.828399209 low

GSM1875990 0.933163794 low

GSM1875991 0.998341458 high

GSM1875992 0.864964502 low

GSM1875993 0.855336758 low

GSM1875994 0.863482009 low

GSM1875995 0.852977766 low

GSM1875996 1.489373144 high

GSM1875997 0.847545185 low

GSM1875998 0.953852833 high

GSM1875999 0.95642429 high

GSM1876000 1.448795475 high

GSM1876001 1.157839771 high

GSM1876002 1.601359641 high

GSM1876003 0.838111878 low

GSM1876004 0.761577448 low

GSM1876005 0.805051616 low

GSM1876006 1.064146529 high

GSM1876007 0.897175041 low

GSM1876008 0.73192623 low

GSM1876009 0.819588159 low

GSM1876010 0.862180348 low

GSM1876011 0.90979698 low

GSM1876012 0.943646246 high

GSM1876013 0.830880019 low

GSM1876014 0.723726224 low

GSM1876015 0.793063308 low

GSM1876016 0.8313009 low

GSM1876017 0.871946926 low

GSM1876018 2.774959869 high

GSM1876019 0.804550906 low

GSM1876020 2.099998875 high

GSM437093 0.951416571 high

GSM437094 0.802816363 low

GSM437095 0.900419081 low

GSM437096 1.176038866 high

GSM437097 0.808743312 low

GSM437098 0.799644063 low

GSM437099 0.987393423 high

GSM437100 0.832537699 low

GSM437101 0.785396428 low

GSM437102 1.104333924 high

GSM437103 0.761863649 low

GSM437104 1.09312627 high

GSM437105 0.873227886 low

GSM437106 0.755915541 low

GSM437107 0.870292608 low

GSM437108 1.198809692 high

GSM437109 0.872545521 low

GSM437110 0.792854218 low

GSM437111 0.914496981 low

GSM437112 0.945134414 high

GSM437113 0.916162463 low

GSM437114 1.255345364 high

GSM437115 0.795240383 low

GSM437116 0.811487204 low

GSM437117 0.963550503 high

GSM437118 1.669631692 high

GSM437119 1.136449836 high

GSM437120 1.014049548 high

GSM437121 0.87384794 low

GSM437122 0.906044075 low

GSM437123 0.887731399 low

GSM437124 1.690744442 high

GSM437125 1.697513419 high

GSM437126 1.222703552 high

GSM437127 0.919456028 low

GSM437128 0.922028836 low

GSM437129 0.916826291 low

GSM437130 0.98441506 high

GSM437131 1.439568554 high

GSM437132 0.830514732 low

GSM437133 1.697750545 high

GSM437134 1.161060233 high

GSM437135 1.242404406 high

GSM437136 0.852230304 low

GSM437137 0.812378099 low

GSM437138 2.029911065 high

GSM437139 0.848204023 low

GSM437140 1.098937907 high

GSM437141 0.95437511 high

GSM437142 0.967022713 high

GSM437143 1.004040528 high

GSM437144 0.891848739 low

GSM437145 1.058688647 high

GSM437146 0.773016328 low

GSM437147 1.499715166 high

GSM437148 0.987178775 high

GSM437149 0.85399782 low

GSM437150 1.307100455 high

GSM437151 0.846876477 low

GSM437152 1.262638499 high

GSM437153 0.875802387 low

GSM437154 0.767478433 low

GSM437155 0.850782307 low

GSM437156 0.993960849 high

GSM437157 1.022603062 high

GSM437158 2.02459808 high

GSM437159 0.871837822 low

GSM437160 0.998432615 high

GSM437161 0.855406886 low

GSM437162 0.767679957 low

GSM437163 0.897040242 low

GSM437164 0.821767464 low

GSM437165 0.849942479 low

GSM437166 0.871606019 low

GSM437167 1.166106554 high

GSM437168 1.07593236 high

GSM437169 0.836362003 low

GSM437170 0.892841019 low

GSM437171 0.89594112 low

GSM437172 0.89270504 low

GSM437173 1.708568529 high

GSM437174 1.132570854 high

GSM437175 0.892102611 low

GSM437176 0.974511881 high

GSM437177 0.801822874 low

GSM437178 1.024267801 high

GSM437179 0.832160743 low

GSM437180 1.373637112 high

GSM437181 0.924071978 low

GSM437182 0.796379133 low

GSM437183 0.839767085 low

GSM437184 0.976461416 high

GSM437185 0.809527408 low

GSM437186 0.920233042 low

GSM437187 0.998576064 high

GSM437188 0.935893331 low

GSM437189 0.958610213 high

GSM437190 1.274769056 high

GSM437191 0.731105111 low

GSM437192 0.756916068 low

GSM437193 0.944643343 high

GSM437194 0.871008958 low

GSM437195 0.914374056 low

GSM437196 0.875093338 low

GSM437197 2.267815951 high

GSM437198 0.815003146 low

GSM437199 0.830400848 low

GSM437200 1.30464163 high

GSM437201 1.704767572 high

GSM437202 1.769414301 high

GSM437203 1.05266923 high

GSM437204 1.043257103 high

GSM437205 0.940914358 low

GSM437206 0.908043138 low

GSM437207 0.864747263 low

GSM437208 1.207639097 high

GSM437209 1.436070587 high

GSM437210 1.02482506 high

GSM437211 1.012316699 high

GSM437212 1.588990795 high

GSM437213 1.153457392 high

GSM437214 0.772477723 low

GSM437215 1.037117171 high

GSM437216 0.968182258 high

GSM437217 0.8991982 low

GSM437218 1.201323332 high

GSM437219 0.93100358 low

GSM437220 0.860689783 low

GSM437221 0.879023153 low

GSM437222 0.953470084 high

GSM437223 0.964494792 high

GSM437225 1.398248391 high

GSM437226 0.971032496 high

GSM437227 1.281004897 high

GSM437228 0.849537265 low

GSM437229 0.848717661 low

GSM437230 0.91032414 low

GSM437231 0.93782158 low

GSM437232 0.901742063 low

GSM437233 1.438790204 high

GSM437234 1.049864655 high

GSM437235 1.285180887 high

GSM437236 0.881074706 low

GSM437237 0.867651154 low

GSM437238 0.958478298 high

GSM437239 1.19711442 high

GSM437240 0.79858421 low

GSM437241 0.772909261 low

GSM437243 0.895813383 low

GSM437244 0.798844411 low

GSM437245 0.854451605 low

GSM437246 0.958478298 high

GSM437247 0.76833279 low

GSM437248 0.851610052 low

GSM437249 0.930630694 low

GSM437250 0.822937384 low

GSM437251 1.337801734 high

GSM437252 0.865003857 low

GSM437253 4.186429704 high

GSM437254 0.878659157 low

GSM437255 0.928483614 low

GSM437256 0.979812139 high

GSM437257 0.920613311 low

GSM437258 0.851024347 low

GSM437259 0.843012089 low

GSM437260 0.94619764 high

GSM437261 0.986418517 high

GSM437262 0.98441506 high

GSM437263 0.924934018 low

GSM437264 0.870036254 low

GSM437265 1.113334881 high

GSM437266 1.388718509 high

GSM437267 0.975923804 high

GSM437268 0.711300542 low

GSM437269 0.879617089 low

GSM971957 0.886810656 low

GSM971958 1.003240857 high

GSM971959 1.002363459 high

GSM971960 0.831343227 low

GSM971961 0.971755304 high

GSM971962 1.011489168 high

GSM971963 1.214441727 high

GSM971964 0.918631762 low

GSM971965 0.917008108 low

GSM971966 0.975039243 high

GSM971967 1.32734516 high

GSM971968 1.072892949 high

GSM971969 0.881833589 low

GSM971970 1.061627444 high

GSM971971 0.948675341 high

GSM971972 0.800621091 low

GSM971973 0.908243754 low

GSM971974 1.097536846 high

GSM971975 0.794486527 low

GSM971976 0.966330532 high

GSM971977 0.96500574 high

GSM971978 1.085826808 high

GSM971979 0.889071914 low

GSM971980 1.566831802 high

GSM971981 1.060204167 high

GSM971982 2.59113445 high

GSM971983 0.807792613 low

GSM971984 0.872659782 low

GSM971985 1.350406129 high

GSM971986 0.72561605 low

GSM971987 0.783424943 low

GSM971988 2.060696552 high

GSM971989 1.456568577 high

GSM971990 0.96646405 high

GSM971991 0.988683264 high

GSM971992 0.879910343 low

GSM971993 0.939128641 low

GSM971994 0.873117904 low

GSM971995 0.797643897 low

GSM971996 1.155893419 high

GSM971997 0.871629941 low

GSM971998 0.771264772 low

GSM971999 0.816274495 low

GSM972000 0.965785847 high

GSM972001 0.800513949 low

GSM972002 1.294694536 high

GSM972003 1.042911745 high

GSM972004 0.862822752 low

GSM972005 1.518239939 high

GSM972006 2.033604557 high

GSM972007 1.767071681 high

GSM972008 2.161424591 high

GSM972009 1.169747494 high

GSM972010 0.971145748 high

GSM972011 1.224785638 high

GSM972012 0.790376055 low

GSM972013 0.859704786 low

GSM972014 0.835814452 low

GSM972015 1.436945804 high

GSM972016 0.8599345 low

GSM972017 0.88557337 low

GSM972018 0.93877153 low

GSM972019 2.199723974 high

GSM972020 0.897987535 low

GSM972021 0.804966828 low

GSM972022 0.981054306 high

GSM972023 0.900063127 low

GSM972024 0.810891133 low

GSM972025 1.084367842 high

GSM972026 1.509551539 high

GSM972027 0.781547366 low

GSM972028 0.808850043 low

GSM972029 0.81015403 low

GSM972030 0.973589522 high

GSM972031 0.933453066 low

GSM972032 0.845417836 low

GSM972033 1.055207591 high

GSM972034 0.91642553 low

GSM972035 0.896268582 low

GSM972036 1.081324917 high

GSM972037 1.215476114 high

GSM972038 0.823581753 low

GSM972039 1.571922261 high

GSM972040 1.033428485 high

GSM972041 1.05407426 high

GSM972042 0.875341089 low

GSM972043 0.917932273 low

GSM972044 0.944644994 high

GSM972045 1.148269657 high

GSM972046 0.928234869 low

GSM972047 0.900624991 low

GSM972048 0.896494263 low

GSM972049 1.044831341 high

GSM972050 0.937593145 low

GSM972051 1.072498407 high

GSM972052 0.883761649 low

GSM972053 1.127012063 high

GSM972054 1.308393176 high

GSM972055 0.989642532 high

GSM972056 1.582027178 high

GSM972057 0.915146775 low

GSM972058 1.048904383 high

GSM972059 1.039731461 high

GSM972060 0.902825533 low

GSM972061 0.952718299 high

GSM972062 1.964308462 high

GSM972063 0.797969587 low

GSM972064 1.171313497 high

GSM972065 0.992451737 high

GSM972066 0.87084571 low

GSM972067 0.998610345 high

GSM972068 1.107942833 high

GSM972069 0.78837277 low

GSM972070 0.766746853 low

GSM972071 0.863290206 low

GSM972072 1.17617684 high

GSM972073 1.029698993 high

GSM972074 1.088725779 high

GSM972075 0.952718299 high

GSM972076 0.867360196 low

GSM972077 1.05740931 high

GSM972078 0.841455172 low

GSM972079 1.612880169 high

GSM972080 0.875579202 low

GSM972081 0.958856286 high

GSM972082 1.545313038 high

GSM972083 1.134078909 high

GSM972084 1.080395933 high

GSM972085 0.995407307 high

GSM972086 1.005232283 high

GSM972087 0.989168335 high

GSM972088 0.933920358 low

GSM972089 0.886468164 low

GSM972090 1.055993087 high

GSM972091 0.825604157 low

GSM972092 0.917125788 low

GSM972093 0.992321762 high

GSM972094 1.152004378 high

GSM972095 0.869373727 low

GSM972096 0.977353068 high

GSM972097 1.328622644 high

GSM972098 0.890347041 low

GSM972099 0.734068469 low

GSM972100 0.88614457 low

GSM972101 0.727673717 low

GSM972102 0.816708471 low

GSM972103 0.784155508 low

GSM972104 0.769647623 low

GSM972105 0.891154841 low

GSM972106 0.677379538 low

GSM972107 1.584947009 high

GSM972108 0.674775714 low

GSM972109 0.77229174 low

GSM972110 1.032479238 high

GSM972111 0.670780722 low

GSM972112 1.755830214 high

GSM972113 1.689488313 high

GSM972114 1.075930076 high

GSM972115 0.97908146 high

GSM972116 0.92509127 low

GSM972117 0.797543378 low

GSM972118 1.038072305 high

GSM972119 0.695440928 low

GSM972120 1.152853175 high

GSM972121 1.799707805 high

GSM972122 0.781229729 low

GSM972123 0.671239577 low

GSM972124 0.657939441 low

GSM972125 0.77585457 low

GSM972126 0.866458544 low

GSM972127 0.904676502 low

GSM972128 0.80443471 low

GSM972129 0.781229729 low

GSM972130 0.942341565 low

GSM972131 2.232470674 high

GSM972132 0.668820414 low

GSM972133 0.945932779 high

GSM972134 1.160828513 high

GSM972135 1.324198021 high

GSM972136 1.111987069 high

GSM972137 0.794486527 low

GSM972138 0.977959464 high

GSM972139 0.905256632 low

GSM972140 1.055207591 high

GSM972141 0.894447907 low

GSM972142 0.83332272 low

GSM972143 0.817028396 low

GSM972144 1.008619578 high

GSM972145 1.037428447 high

GSM972146 1.205336857 high

GSM972147 0.864998357 low

GSM972148 0.945351294 high

GSM972149 0.853464229 low

GSM972150 0.830895079 low

GSM972151 0.858030564 low

GSM972152 0.769053558 low

GSM972153 0.936409913 low

GSM972154 1.103113246 high

GSM972155 1.955176971 high

GSM972156 0.761303664 low

GSM972157 0.903394656 low

GSM972158 1.026141641 high

GSM972159 0.955716198 high

GSM972160 0.893544741 low

GSM972161 0.975896744 high

GSM972162 0.900624991 low

GSM972163 0.954739754 high

GSM972164 0.829240791 low

GSM972165 0.868359682 low

GSM972166 0.893435351 low

GSM972167 0.908018203 low

GSM972168 1.036003793 high

GSM972169 1.02411522 high

GSM972170 0.850704231 low

GSM972171 1.243601065 high

GSM972172 0.998010119 high

GSM972173 1.176323604 high

GSM972174 0.726778281 low

GSM972175 0.69608531 low

GSM972176 1.357594854 high

GSM972177 1.237529644 high

GSM972178 0.764133005 low

GSM972179 1.180356325 high

GSM972180 0.976976496 high

GSM972181 1.462619482 high

GSM972182 0.950692225 high

GSM972183 0.922157326 low

GSM972184 3.713543766 high

GSM972185 0.993931763 high

GSM972186 1.289633262 high

GSM972187 0.948561612 high

GSM972188 0.829240791 low

GSM972189 0.981791772 high

GSM972190 0.800307515 low

GSM972191 0.653318923 low

GSM972192 0.798708554 low

GSM972193 1.064270715 high

GSM972194 0.7684644 low

GSM972195 0.827090106 low

GSM972196 0.763428038 low

GSM972197 0.957773312 high

GSM972198 0.930369127 low

GSM972199 0.824644211 low

GSM972200 0.779559743 low

GSM972201 1.321074693 high

GSM972202 0.917125788 low

GSM972203 0.906063244 low

GSM972204 0.951277234 high

GSM972205 0.846712379 low

GSM972206 1.007360058 high

GSM972207 0.813796933 low

GSM972208 0.889997156 low

GSM972209 0.911090981 low

GSM972210 0.857690773 low

GSM972211 0.956924056 high

GSM972212 0.898456477 low

GSM972213 1.341431704 high

GSM972214 0.808850043 low

GSM972215 0.830895079 low

GSM972216 0.83917686 low

GSM972217 0.829354616 low

GSM972218 2.477337241 high

GSM972219 0.936289042 low

GSM972220 1.236957896 high

GSM972221 0.941279983 low

GSM972222 0.938651341 low

GSM972223 1.051095384 high

GSM972224 1.127439312 high

GSM972225 0.948214152 high

GSM972226 0.993931763 high

GSM972227 0.985725904 high

GSM972228 0.907902386 low

GSM972230 0.848472168 low

GSM972231 0.918287461 low

GSM972232 0.958976057 high

GSM972233 0.845716313 low

GSM972234 0.884208581 low

GSM972235 0.899948589 low

GSM972236 0.888187376 low

GSM972237 0.911647482 low

GSM972238 1.018086211 high

GSM972239 0.910642661 low

GSM972240 1.089799901 high

GSM972241 1.015653072 high

GSM972242 0.885128502 low

GSM972243 0.911426125 low

GSM972244 0.967055204 high

GSM972247 0.908584618 low

GSM972248 0.856559766 low

GSM972249 0.851795908 low

GSM972250 0.89546237 low

GSM972251 0.897864307 low

GSM972252 1.003844658 high

GSM972253 0.928234869 low

GSM972254 0.863406523 low

GSM972255 0.895687137 low

GSM972256 1.024738901 high

GSM972257 0.971639363 high

GSM972259 0.977833309 high

GSM972260 0.996893785 high

GSM972261 0.979830121 high

GSM972262 0.946534859 high

GSM972263 0.935568952 low

GSM972264 1.026659766 high

GSM972265 0.886362216 low

GSM972266 0.996893785 high

GSM972267 0.912106498 low

GSM972268 0.96103592 high

GSM972269 1.353904392 high

GSM972270 0.90203046 low

GSM972271 0.856559766 low

GSM972272 0.930369127 low

GSM972273 0.952111597 high

GSM972274 1.120587855 high

GSM972275 3.170091012 high

GSM972276 2.320489354 high

GSM972277 0.940439339 low

GSM972278 1.042787636 high

GSM972279 1.001856738 high

GSM972280 0.999125319 high

GSM972281 0.96646405 high

GSM972282 1.084642708 high

GSM972283 1.275942464 high

GSM972284 0.863631897 low

GSM972285 0.930369127 low

GSM972286 1.019097403 high

GSM972287 1.027800544 high

GSM972288 0.981540834 high

GSM972289 0.9624688 high

GSM972290 1.04633434 high

GSM972291 1.059932436 high

GSM972292 0.862822752 low

GSM972293 1.057287681 high

GSM972294 0.983498944 high

GSM972295 0.987210337 high

GSM972296 1.376923611 high

GSM972297 0.966937204 high

GSM972298 1.635665887 high

GSM972299 0.878437673 low

GSM972300 1.483117744 high

GSM972301 0.917008108 low

GSM972302 0.856446861 low

GSM972303 1.247514582 high

GSM972304 0.962958919 high

GSM972305 1.177890957 high

GSM972306 1.008744962 high

GSM972307 0.905943266 low

GSM972308 4.115036087 high

GSM972309 0.715422706 low

GSM972310 0.954138643 high

GSM972311 0.833758609 low

GSM972312 0.82871717 low

GSM972313 1.293004518 high

GSM972314 0.958252134 high

GSM972315 0.69608531 low

GSM972316 1.251425746 high

GSM972317 1.021002525 high

GSM972318 0.94271779 high

GSM972319 0.883648718 low

GSM972320 0.854805572 low

GSM972321 1.274113397 high

GSM972322 0.906178952 low

GSM972323 0.897075251 low

GSM972324 0.950450436 high

GSM972325 0.955841815 high

GSM972326 1.28869804 high

GSM972327 1.579887902 high

GSM972328 0.905372294 low

GSM972329 0.839506078 low

GSM972330 1.467475137 high

GSM972331 0.921789646 low

GSM972332 0.974544861 high

GSM972333 0.9513974 high

GSM972334 1.078935083 high

GSM972335 0.862822752 low

GSM972336 1.045441994 high

GSM972337 0.997266183 high

GSM972338 0.950571245 high

GSM972339 0.797857889 low

GSM972340 1.010750189 high

GSM972341 0.900287531 low

GSM972342 0.927526276 low

GSM972343 0.822621753 low

GSM972344 1.159304688 high

GSM972345 1.755236402 high

GSM972346 0.985601405 high

GSM972347 0.765655172 low

GSM972348 0.755263393 low

GSM972349 1.50798239 high

GSM972350 0.832326543 low

GSM972351 0.974667922 high

GSM972352 1.019611075 high

GSM972353 1.082910601 high

GSM972354 1.29874302 high

GSM972355 1.101605261 high

GSM972356 0.949984738 high

GSM972357 1.056253734 high

GSM972358 0.977353068 high

GSM972359 0.926614691 low

GSM972360 0.780489585 low

GSM972361 3.625156874 high

GSM972362 0.873909601 low

GSM972363 0.880587167 low

GSM972364 1.021870359 high

GSM972365 2.930542039 high

GSM972366 1.067456881 high

GSM972367 0.854365012 low

GSM972368 0.894113346 low

GSM972369 0.925449742 low

GSM972370 0.879910343 low

GSM972371 0.903394656 low

GSM972372 0.938418255 low

GSM972373 0.810044023 low

GSM972374 0.834942671 low

GSM972375 0.976490912 high

GSM972376 0.762011556 low

GSM972377 0.841887832 low

GSM972378 0.876394327 low

GSM972379 0.84716716 low

GSM972380 0.976490912 high

GSM972381 0.935341671 low

GSM972382 0.836887832 low

GSM972383 0.775447275 low

GSM972384 0.990123616 high

GSM972385 0.644871467 low

GSM972386 0.826661069 low

GSM972387 0.904791225 low

GSM972388 0.745265858 low

GSM972389 1.128259883 high

GSM972390 1.270772661 high

GSM972391 0.843744478 low

GSM972392 0.98299724 high

GSM972393 0.70889165 low

GSM972394 1.168609512 high

GSM972395 0.791323189 low

GSM972396 1.093945378 high

GSM972397 0.89879394 low

GSM972398 1.13931401 high

GSM972399 0.841455172 low

GSM972400 0.966084707 high

GSM972402 1.037817719 high

GSM972403 1.10904868 high

GSM972404 0.82134151 low

GSM972405 1.108633772 high

GSM972406 0.833535416 low

GSM972407 0.891496494 low

GSM972408 1.297664482 high

GSM972409 1.018210364 high

GSM972410 0.835485581 low

GSM972411 0.913734156 low

GSM972412 1.060989888 high

GSM972413 1.125218338 high

GSM972414 0.942968407 high

GSM972415 0.80906461 low

GSM972416 0.957773312 high

GSM972417 0.810891133 low

GSM972418 0.908804312 low

GSM972419 0.741488891 low

GSM972420 1.398525561 high

GSM972421 0.989878759 high

GSM972422 1.455576513 high

GSM972423 0.718641512 low

GSM972424 0.912457337 low

GSM972425 1.15688097 high

GSM972426 0.940673143 low

GSM972427 0.921789646 low

GSM972428 1.049801375 high

GSM972429 0.906860887 low

GSM972430 2.493202078 high

GSM972431 0.930130955 low

GSM972432 1.75264072 high

GSM972433 0.71836609 low

GSM972434 0.804654746 low

GSM972435 0.832326543 low

GSM972436 0.753848495 low

GSM972437 1.171744219 high

GSM972438 1.48120351 high

GSM972439 1.229479693 high

GSM972440 1.094081268 high

GSM972441 1.012627257 high

GSM972442 0.824539718 low

GSM972443 0.851915521 low

GSM972444 2.077851729 high

GSM972445 3.222568558 high

GSM972447 1.166202688 high

GSM972449 0.928234869 low

GSM972450 0.880024496 low

GSM972451 0.71449027 low

GSM972452 0.834620981 low

GSM972453 1.036003793 high

GSM972454 0.881377541 low

GSM972455 1.13749414 high

GSM972457 1.331893643 high

GSM972458 1.023493198 high

GSM972459 1.453211923 high

GSM972460 0.983371233 high

GSM972461 0.801255274 low

GSM972462 0.954980909 high

GSM972463 0.821132176 low

GSM972464 0.738554209 low

GSM972465 0.902490924 low

GSM972466 0.824864939 low

GSM972467 1.006350739 high

GSM972468 0.857690773 low

GSM972469 1.080650926 high

GSM972470 0.904676502 low

GSM972472 0.950107392 high

GSM972473 0.821547577 low

GSM972474 0.8590387 low

GSM972475 0.768566113 low

GSM972476 0.781763413 low

GSM972477 1.298120556 high

GSM972478 0.883306219 low

GSM972479 1.620085622 high

GSM972480 1.052512113 high

GSM972481 0.910642661 low

GSM972482 1.184519018 high

GSM972483 2.113817266 high

GSM972484 0.990619835 high

GSM972485 1.182203598 high

GSM972486 0.863968393 low

GSM972487 0.937593145 low

GSM972488 1.086490227 high

GSM972489 0.993069125 high

GSM972490 0.8241222 low

GSM972491 0.984104501 high

GSM972492 1.214441727 high

GSM972493 0.896617748 low

GSM972494 1.262749126 high

GSM972495 0.977833309 high

GSM972496 0.907552293 low

GSM972497 0.952474924 high

GSM972498 1.021129573 high

GSM972499 1.915711326 high

GSM972501 0.855248306 low

GSM972502 1.10229617 high

GSM972503 1.106209181 high

GSM972504 0.858030564 low

GSM972505 1.058600253 high

GSM972506 0.788577309 low

GSM972507 0.905030055 low

GSM972508 0.899948589 low

GSM972509 0.837993234 low

GSM972510 0.890347041 low

GSM972511 1.476356003 high

GSM972512 2.068779579 high

GSM972513 0.894447907 low

GSM972514 0.923695728 low

GSM972515 0.954621221 high

GSM972516 0.926042199 low

GSM972517 0.945351294 high

GSM972518 0.987686373 high

GSM972519 0.904676502 low

GSM972520 1.640718113 high

GSM972521 0.765145426 low

GSM972522 1.532352354 high

**Supplementary Table S8.** The stromal score and immune score of TCGA samples by ESTIMATE algorithm.

| Sample | StromalScore | ImmuneScore | ESTIMATEScore |
| --- | --- | --- | --- |
| TCGA_TCGA-DM-A288 | -1064.36 | 561.9183 | -502.44 |
| TCGA_TCGA-QL-A97D | -288.84 | 3139.759 | 2850.92 |
| TCGA_TCGA-CM-6164 | 858.998 | 2553.859 | 3412.857 |
| TCGA_TCGA-G4-6299 | 1707.619 | 3735.973 | 5443.592 |
| TCGA_TCGA-F4-6463 | 1913.894 | 2580.694 | 4494.588 |
| TCGA_TCGA-AZ-4615 | 1779.649 | 4122.623 | 5902.272 |
| TCGA_TCGA-AA-3549 | 591.5384 | 2883.142 | 3474.681 |
| TCGA_TCGA-AY-4071 | 1456.977 | 4115.45 | 5572.427 |
| TCGA_TCGA-CM-4752 | 1490.017 | 3634.081 | 5124.097 |
| TCGA_TCGA-DM-A1D9 | -453.759 | 968.8853 | 515.126 |
| TCGA_TCGA-AA-3688 | 496.4294 | 2599.463 | 3095.892 |
| TCGA_TCGA-AA-3854 | 6.278004 | 2172.351 | 2178.629 |
| TCGA_TCGA-A6-3809 | 1908.267 | 4445.169 | 6353.437 |
| TCGA_TCGA-CM-6165 | 2104.664 | 3127.329 | 5231.993 |
| TCGA_TCGA-CM-4751 | 1424.006 | 3642.721 | 5066.728 |
| TCGA_TCGA-A6-5659 | 868.7894 | 1197.642 | 2066.431 |
| TCGA_TCGA-AA-3494 | 189.3199 | 2441.201 | 2630.521 |
| TCGA_TCGA-CM-4750 | 405.6487 | 2372.008 | 2777.657 |
| TCGA_TCGA-AZ-4682 | 44.32294 | 1971.402 | 2015.725 |
| TCGA_TCGA-G4-6625 | 2076.499 | 4577.872 | 6654.371 |
| TCGA_TCGA-DM-A0XF | 549.7634 | 2163.414 | 2713.178 |
| TCGA_TCGA-AA-3529 | 259.3841 | 2158.636 | 2418.02 |
| TCGA_TCGA-AA-3949 | 2311.098 | 5039.506 | 7350.604 |
| TCGA_TCGA-AA-3848 | 48.56644 | 2354.055 | 2402.621 |
| TCGA_TCGA-CA-6715 | -631.006 | 662.3765 | 31.37057 |
| TCGA_TCGA-AA-3818 | 373.9722 | 2342.699 | 2716.672 |
| TCGA_TCGA-AA-3710 | 1656.421 | 4988.427 | 6644.848 |
| TCGA_TCGA-AA-3950 | 2506.206 | 4737.946 | 7244.152 |
| TCGA_TCGA-AA-A00N | 2199.159 | 3138.845 | 5338.004 |
| TCGA_TCGA-A6-6138 | 2274.187 | 4420.467 | 6694.655 |
| TCGA_TCGA-DM-A28E | -1832.56 | 640.124 | -1192.44 |
| TCGA_TCGA-D5-6538 | -929.892 | -17.1101 | -947.002 |
| TCGA_TCGA-AA-A00K | 411.6968 | 2485.168 | 2896.865 |
| TCGA_TCGA-AA-3715 | 2498.546 | 4663.922 | 7162.468 |
| TCGA_TCGA-AA-3506 | 1148.319 | 3481.811 | 4630.13 |
| TCGA_TCGA-AU-3779 | 1603.47 | 3750.239 | 5353.709 |
| TCGA_TCGA-CK-5916 | 1790.147 | 4309.864 | 6100.011 |
| TCGA_TCGA-QG-A5Z1 | 1108.569 | 1608.098 | 2716.667 |
| TCGA_TCGA-AD-6890 | 695.8661 | 2306.896 | 3002.762 |
| TCGA_TCGA-A6-2685 | 2850.221 | 4178.013 | 7028.234 |
| TCGA_TCGA-AA-A00W | -1882.11 | 1744.821 | -137.291 |
| TCGA_TCGA-CK-5914 | 882.3306 | 2200.421 | 3082.752 |
| TCGA_TCGA-A6-2686 | 2194.031 | 4861.706 | 7055.737 |
| TCGA_TCGA-CA-5255 | -1430.2 | 1122.287 | -307.916 |
| TCGA_TCGA-A6-A5ZU | 1982.47 | 3442.567 | 5425.037 |
| TCGA_TCGA-NH-A50V | 1757.931 | 3104.136 | 4862.066 |
| TCGA_TCGA-QG-A5YX | -1476.29 | 1301.792 | -174.493 |
| TCGA_TCGA-A6-2677 | 411.5947 | 1228.682 | 1640.276 |
| TCGA_TCGA-CA-6717 | 2850.036 | 4361.813 | 7211.849 |
| TCGA_TCGA-A6-6649 | 1626.739 | 3362.693 | 4989.433 |
| TCGA_TCGA-NH-A6GC | 973.6192 | 1578.777 | 2552.397 |
| TCGA_TCGA-AA-3562 | 1218.532 | 2938.323 | 4156.855 |
| TCGA_TCGA-A6-4105 | 1865.106 | 3978.922 | 5844.028 |
| TCGA_TCGA-A6-6141 | 795.5752 | 3459.508 | 4255.083 |
| TCGA_TCGA-AA-A00R | 1296.225 | 4906.603 | 6202.828 |
| TCGA_TCGA-AZ-4684 | 1704.287 | 3595.628 | 5299.914 |
| TCGA_TCGA-CK-6747 | 823.3702 | 2599.849 | 3423.22 |
| TCGA_TCGA-AU-6004 | 1709.252 | 3916.645 | 5625.897 |
| TCGA_TCGA-A6-2672 | 1793.039 | 4642.878 | 6435.916 |
| TCGA_TCGA-QG-A5Z2 | -1343.84 | 3305.552 | 1961.709 |
| TCGA_TCGA-A6-6780 | 1673.664 | 4745.989 | 6419.653 |
| TCGA_TCGA-F4-6459 | 2448.92 | 2673.64 | 5122.56 |
| TCGA_TCGA-AA-3970 | 481.9335 | 3391.091 | 3873.025 |
| TCGA_TCGA-NH-A8F7 | -1676.01 | -143.893 | -1819.91 |
| TCGA_TCGA-G4-6321 | -715.97 | 3304.966 | 2588.995 |
| TCGA_TCGA-AY-6196 | 4160.101 | 5814.485 | 9974.585 |
| TCGA_TCGA-AA-3524 | 563.048 | 2539.842 | 3102.89 |
| TCGA_TCGA-DM-A1D0 | -1850.59 | -145.501 | -1996.09 |
| TCGA_TCGA-G4-6307 | -1433.24 | 685.959 | -747.282 |
| TCGA_TCGA-DM-A1HB | -370.035 | 1407.91 | 1037.875 |
| TCGA_TCGA-AA-3697 | 516.6708 | 2810.48 | 3327.151 |
| TCGA_TCGA-CM-6679 | 2146.168 | 3217.193 | 5363.361 |
| TCGA_TCGA-AY-6197 | -442.119 | 2571.354 | 2129.235 |
| TCGA_TCGA-AA-3672 | 1191.662 | 4219.55 | 5411.212 |
| TCGA_TCGA-AA-3684 | 2539.548 | 4012.849 | 6552.398 |
| TCGA_TCGA-A6-2684 | 2863.898 | 3661.491 | 6525.389 |
| TCGA_TCGA-AZ-4323 | 2224.951 | 5042.211 | 7267.161 |
| TCGA_TCGA-AA-3971 | 886.4402 | 3803.782 | 4690.222 |
| TCGA_TCGA-AA-3989 | 1631.867 | 3657.745 | 5289.611 |
| TCGA_TCGA-AA-A00D | 1798.821 | 4617.797 | 6416.618 |
| TCGA_TCGA-AD-6548 | 1695.108 | 3710.69 | 5405.798 |
| TCGA_TCGA-D5-6539 | 452.3279 | 2938.149 | 3390.477 |
| TCGA_TCGA-G4-6302 | 3182.871 | 3760.118 | 6942.989 |
| TCGA_TCGA-A6-2679 | 890.9722 | 3692.691 | 4583.663 |
| TCGA_TCGA-A6-2674 | 3288.425 | 4665.528 | 7953.953 |
| TCGA_TCGA-AZ-4614 | -1411.37 | 1511.927 | 100.5539 |
| TCGA_TCGA-NH-A5IV | 1182.562 | 3693.554 | 4876.116 |
| TCGA_TCGA-A6-5664 | 2581.377 | 4011.216 | 6592.593 |
| TCGA_TCGA-AZ-4315 | 938.2714 | 3031.332 | 3969.603 |
| TCGA_TCGA-F4-6806 | 886.2118 | 2569.841 | 3456.053 |
| TCGA_TCGA-AA-3510 | 937.2972 | 3659.348 | 4596.645 |
| TCGA_TCGA-AY-A71X | -2760.52 | 212.9654 | -2547.55 |
| TCGA_TCGA-AA-3488 | -393.09 | 2161.864 | 1768.773 |
| TCGA_TCGA-DM-A28F | -701.328 | 1335.842 | 634.5148 |
| TCGA_TCGA-G4-6293 | 631.443 | 3694.599 | 4326.042 |
| TCGA_TCGA-5M-AAT4 | -692.599 | 768.6221 | 76.02298 |
| TCGA_TCGA-D5-6533 | 834.7543 | 2393.897 | 3228.651 |
| TCGA_TCGA-A6-5657 | 1907.35 | 3312.291 | 5219.642 |
| TCGA_TCGA-AA-3941 | -254.468 | 1905.355 | 1650.887 |
| TCGA_TCGA-DM-A28K | -822.785 | 1256.286 | 433.501 |
| TCGA_TCGA-CM-5344 | 1536.238 | 1817.599 | 3353.837 |
| TCGA_TCGA-AA-3517 | 920.6732 | 2902.013 | 3822.687 |
| TCGA_TCGA-G4-6309 | -370.152 | 2050.925 | 1680.773 |
| TCGA_TCGA-AY-6386 | 46.86245 | 2930.596 | 2977.459 |
| TCGA_TCGA-AA-3678 | 414.2429 | 3381.155 | 3795.398 |
| TCGA_TCGA-AA-A01X | 43.57533 | 1702.93 | 1746.505 |
| TCGA_TCGA-AA-3821 | 1439.899 | 2990.256 | 4430.154 |
| TCGA_TCGA-CM-6677 | 1612.897 | 2914.26 | 4527.156 |
| TCGA_TCGA-CM-5861 | -293.279 | 1688.83 | 1395.551 |
| TCGA_TCGA-D5-6923 | 1924.969 | 2545.844 | 4470.813 |
| TCGA_TCGA-F4-6703 | 4082.294 | 5497.649 | 9579.943 |
| TCGA_TCGA-AZ-6605 | 2981.279 | 4153.22 | 7134.499 |
| TCGA_TCGA-NH-A6GB | 143.3552 | 2842.865 | 2986.221 |
| TCGA_TCGA-CM-5349 | 1771.518 | 3201.415 | 4972.934 |
| TCGA_TCGA-NH-A50U | 451.0088 | 1467.211 | 1918.22 |
| TCGA_TCGA-AA-3530 | -103.879 | 2885.429 | 2781.55 |
| TCGA_TCGA-G4-6627 | 2298.068 | 4033.718 | 6331.786 |
| TCGA_TCGA-DM-A28G | -536.228 | 1573.302 | 1037.074 |
| TCGA_TCGA-D5-6537 | -929.924 | 1872.464 | 942.5403 |
| TCGA_TCGA-D5-5539 | 1914.864 | 3228.486 | 5143.35 |
| TCGA_TCGA-CM-6163 | 1801.229 | 3629.376 | 5430.605 |
| TCGA_TCGA-CM-4744 | -532.471 | 2979.733 | 2447.262 |
| TCGA_TCGA-AA-A004 | 777.3857 | 3211.652 | 3989.038 |
| TCGA_TCGA-A6-3808 | 2652.247 | 4074.378 | 6726.625 |
| TCGA_TCGA-AA-A010 | 186.8248 | 2598.666 | 2785.49 |
| TCGA_TCGA-AA-A02J | -1820.13 | 236.8461 | -1583.28 |
| TCGA_TCGA-AA-3837 | 981.6176 | 2495.321 | 3476.939 |
| TCGA_TCGA-CK-4947 | 1643.961 | 3734.612 | 5378.572 |
| TCGA_TCGA-CA-5256 | 456.1434 | 2729.578 | 3185.722 |
| TCGA_TCGA-CM-6167 | 3438.281 | 3926.24 | 7364.521 |
| TCGA_TCGA-4N-A93T | -1937.55 | 953.4249 | -984.128 |
| TCGA_TCGA-AA-3521 | 959.1134 | 2390.363 | 3349.477 |
| TCGA_TCGA-CA-5797 | 1383.904 | 2478.789 | 3862.694 |
| TCGA_TCGA-CM-6166 | 342.4494 | 599.5663 | 942.0158 |
| TCGA_TCGA-CK-4951 | 2146.858 | 4113.828 | 6260.686 |
| TCGA_TCGA-CA-6719 | 2187.168 | 3190.083 | 5377.251 |
| TCGA_TCGA-AA-A02R | 1507.803 | 4440.167 | 5947.97 |
| TCGA_TCGA-AA-A02O | -254.868 | 2248.786 | 1993.917 |
| TCGA_TCGA-A6-5666 | -697.451 | 1210.294 | 512.8434 |
| TCGA_TCGA-AY-5543 | -319.391 | 2730.015 | 2410.624 |
| TCGA_TCGA-AA-3845 | 1161.204 | 3733.376 | 4894.58 |
| TCGA_TCGA-F4-6704 | 3085.247 | 3485.088 | 6570.335 |
| TCGA_TCGA-AA-3856 | 887.5012 | 4004.599 | 4892.1 |
| TCGA_TCGA-AA-A00Q | 549.8993 | 2322.378 | 2872.278 |
| TCGA_TCGA-A6-6781 | 3934.615 | 5002.801 | 8937.415 |
| TCGA_TCGA-AA-A00F | 1034.028 | 2187.346 | 3221.374 |
| TCGA_TCGA-D5-6920 | 667.6909 | 3421.304 | 4088.995 |
| TCGA_TCGA-AA-3815 | 1071.989 | 4465.425 | 5537.414 |
| TCGA_TCGA-D5-5538 | 2285.467 | 3992.423 | 6277.89 |
| TCGA_TCGA-AA-3867 | 2070.214 | 2780.666 | 4850.88 |
| TCGA_TCGA-A6-6782 | 2600.224 | 3793.528 | 6393.751 |
| TCGA_TCGA-A6-6654 | 3359.237 | 4949.274 | 8308.512 |
| TCGA_TCGA-AA-A01D | 1954.604 | 3157.73 | 5112.333 |
| TCGA_TCGA-G4-6311 | 1593.502 | 2976.415 | 4569.917 |
| TCGA_TCGA-A6-3807 | 1431.38 | 3391.831 | 4823.211 |
| TCGA_TCGA-AZ-5403 | 1812.494 | 2473.989 | 4286.483 |
| TCGA_TCGA-AD-6888 | -2506.62 | 956.238 | -1550.38 |
| TCGA_TCGA-G4-6628 | 1718.527 | 4733.486 | 6452.012 |
| TCGA_TCGA-DM-A1D6 | -1634.21 | -325.656 | -1959.87 |
| TCGA_TCGA-CM-5341 | 2653.55 | 4221.046 | 6874.597 |
| TCGA_TCGA-AA-3862 | 872.1491 | 3686.976 | 4559.125 |
| TCGA_TCGA-AA-3939 | 978.1859 | 3226.574 | 4204.76 |
| TCGA_TCGA-AA-3514 | 1746.411 | 2612.238 | 4358.649 |
| TCGA_TCGA-AA-3660 | 737.3017 | 2318.635 | 3055.937 |
| TCGA_TCGA-AA-3833 | 1806.627 | 3786.524 | 5593.151 |
| TCGA_TCGA-AA-A01P | 2270.199 | 4795.284 | 7065.483 |
| TCGA_TCGA-AA-A00Z | -28.6443 | 2078.98 | 2050.336 |
| TCGA_TCGA-CM-5863 | 1939.031 | 2946.482 | 4885.512 |
| TCGA_TCGA-AZ-6598 | 558.1043 | 3357.43 | 3915.534 |
| TCGA_TCGA-AA-3679 | 713.4835 | 2600.168 | 3313.651 |
| TCGA_TCGA-G4-6304 | -1463.69 | 2116.002 | 652.3125 |
| TCGA_TCGA-DM-A285 | 622.2571 | 632.501 | 1254.758 |
| TCGA_TCGA-G4-6294 | -38.3565 | 1990.372 | 1952.016 |
| TCGA_TCGA-A6-4107 | 1726.455 | 3290.071 | 5016.526 |
| TCGA_TCGA-DM-A1D4 | -1839.88 | 1530.149 | -309.726 |
| TCGA_TCGA-CA-6716 | 681.5232 | 940.4723 | 1621.996 |
| TCGA_TCGA-CK-4952 | 47.56635 | 2192.832 | 2240.399 |
| TCGA_TCGA-AA-A01V | -1410.55 | 2525.222 | 1114.672 |
| TCGA_TCGA-AA-3844 | 174.7655 | 2829.842 | 3004.608 |
| TCGA_TCGA-D5-6541 | 2441.07 | 3943.21 | 6384.28 |
| TCGA_TCGA-AA-A01G | -665.516 | 1361.544 | 696.0283 |
| TCGA_TCGA-AA-3870 | 2256.554 | 3817.913 | 6074.467 |
| TCGA_TCGA-D5-6532 | -293.518 | 1266.663 | 973.1453 |
| TCGA_TCGA-CM-6169 | 3192.704 | 4562.383 | 7755.087 |
| TCGA_TCGA-D5-5541 | 1858.054 | 3313.144 | 5171.197 |
| TCGA_TCGA-DM-A1DB | -1770.73 | 1826.067 | 55.34143 |
| TCGA_TCGA-AA-A00A | 1054.446 | 3132.399 | 4186.845 |
| TCGA_TCGA-D5-6530 | 827.3482 | 3915.285 | 4742.633 |
| TCGA_TCGA-AA-3855 | 621.193 | 3359.105 | 3980.299 |
| TCGA_TCGA-CM-5868 | 1083.209 | 1872.769 | 2955.978 |
| TCGA_TCGA-AA-3519 | 559.795 | 2736.297 | 3296.092 |
| TCGA_TCGA-AA-3526 | 1166.018 | 3425.375 | 4591.393 |
| TCGA_TCGA-DM-A28M | -1989.54 | 878.4395 | -1111.1 |
| TCGA_TCGA-D5-6926 | 2278.262 | 3216.131 | 5494.393 |
| TCGA_TCGA-AA-3534 | 884.0273 | 2138.909 | 3022.936 |
| TCGA_TCGA-G4-6317 | -1229.66 | 130.3563 | -1099.3 |
| TCGA_TCGA-AA-A03J | 839.314 | 3240.556 | 4079.87 |
| TCGA_TCGA-CM-6680 | 1870.483 | 3527.098 | 5397.581 |
| TCGA_TCGA-AA-A01K | 1615.689 | 2977.028 | 4592.717 |
| TCGA_TCGA-AA-3984 | 1280.793 | 3192.697 | 4473.491 |
| TCGA_TCGA-D5-6536 | 1086.066 | 2644.344 | 3730.41 |
| TCGA_TCGA-AD-A5EK | 152.8954 | 1144.637 | 1297.532 |
| TCGA_TCGA-AA-3663 | 269.2995 | 2724.151 | 2993.45 |
| TCGA_TCGA-AZ-6607 | 3108.511 | 3829.88 | 6938.391 |
| TCGA_TCGA-AA-3520 | 1895.954 | 3187.413 | 5083.366 |
| TCGA_TCGA-AA-3543 | 1315.518 | 4335.274 | 5650.792 |
| TCGA_TCGA-AZ-5407 | -661.188 | 2852.006 | 2190.818 |
| TCGA_TCGA-G4-6314 | 2254.802 | 2598.202 | 4853.004 |
| TCGA_TCGA-CM-5348 | 2800.076 | 3743.12 | 6543.196 |
| TCGA_TCGA-CK-6751 | 1475.692 | 2595.137 | 4070.828 |
| TCGA_TCGA-D5-6929 | 1882.078 | 3074.71 | 4956.788 |
| TCGA_TCGA-AA-3675 | 437.4809 | 2266.285 | 2703.766 |
| TCGA_TCGA-A6-2681 | 2132.072 | 3245.789 | 5377.861 |
| TCGA_TCGA-AA-3869 | 1413.154 | 3736.602 | 5149.756 |
| TCGA_TCGA-DM-A282 | -22.5222 | 784.9735 | 762.4513 |
| TCGA_TCGA-D5-7000 | 1405.795 | 3097.172 | 4502.967 |
| TCGA_TCGA-AM-5821 | 1188.232 | 3967.362 | 5155.594 |
| TCGA_TCGA-G4-6298 | 285.9235 | 348.5556 | 634.4791 |
| TCGA_TCGA-AA-A01S | -1818.06 | 485.571 | -1332.49 |
| TCGA_TCGA-NH-A8F8 | 1400.605 | 2382.046 | 3782.651 |
| TCGA_TCGA-AA-3966 | 2323.455 | 4847.841 | 7171.295 |
| TCGA_TCGA-AA-3516 | 726.7988 | 3408.738 | 4135.537 |
| TCGA_TCGA-A6-2676 | 1513.494 | 4342.895 | 5856.39 |
| TCGA_TCGA-AA-3846 | 757.4202 | 3165.542 | 3922.962 |
| TCGA_TCGA-AA-3544 | 2076.651 | 4402.487 | 6479.138 |
| TCGA_TCGA-AA-3956 | 1292.205 | 3015.598 | 4307.804 |
| TCGA_TCGA-AA-3532 | 1896.871 | 4155.802 | 6052.673 |
| TCGA_TCGA-AA-3955 | 96.08225 | 2098.334 | 2194.416 |
| TCGA_TCGA-CK-5912 | 706.2732 | 1796.544 | 2502.817 |
| TCGA_TCGA-AD-6895 | 1291.211 | 3388.895 | 4680.106 |
| TCGA_TCGA-AA-3556 | 707.4427 | 2908.865 | 3616.308 |
| TCGA_TCGA-CM-6678 | 291.5247 | 1217.439 | 1508.964 |
| TCGA_TCGA-AA-3851 | 1062.932 | 3514.757 | 4577.689 |
| TCGA_TCGA-CM-5860 | 2439.617 | 3113.356 | 5552.974 |
| TCGA_TCGA-A6-5661 | 1207.135 | 3339.967 | 4547.103 |
| TCGA_TCGA-AA-A02F | -22.6636 | 455.9992 | 433.3356 |
| TCGA_TCGA-AA-3861 | -779.755 | 3319.738 | 2539.983 |
| TCGA_TCGA-DM-A1DA | -958.121 | 1173.849 | 215.7281 |
| TCGA_TCGA-CM-6162 | 3575.24 | 4716.733 | 8291.973 |
| TCGA_TCGA-AA-3522 | -64.2877 | 2817.442 | 2753.155 |
| TCGA_TCGA-AA-A00U | -57.6798 | 1908.658 | 1850.978 |
| TCGA_TCGA-F4-6807 | 3095.238 | 4296.422 | 7391.66 |
| TCGA_TCGA-AA-3930 | 1282.462 | 3550.498 | 4832.96 |
| TCGA_TCGA-AZ-6599 | -2351.29 | 760.3145 | -1590.97 |
| TCGA_TCGA-AA-3947 | 1792.963 | 3202.544 | 4995.507 |
| TCGA_TCGA-AA-3841 | 1187.161 | 3480.465 | 4667.626 |
| TCGA_TCGA-CM-6171 | 467.4716 | 2834.482 | 3301.954 |
| TCGA_TCGA-CM-4743 | 254.3763 | 3016.064 | 3270.44 |
| TCGA_TCGA-AA-3814 | 2130.017 | 3968.548 | 6098.564 |
| TCGA_TCGA-NH-A6GA | 174.9279 | 2010.138 | 2185.066 |
| TCGA_TCGA-A6-2675 | 2566.221 | 3702.722 | 6268.943 |
| TCGA_TCGA-G4-6297 | 2336.253 | 3358.793 | 5695.046 |
| TCGA_TCGA-A6-5656 | 1337.534 | 1985.612 | 3323.147 |
| TCGA_TCGA-DM-A0X9 | -103.924 | 2311.365 | 2207.441 |
| TCGA_TCGA-AA-3553 | 1489.392 | 3652.724 | 5142.116 |
| TCGA_TCGA-DM-A0XD | 193.1659 | 1526.046 | 1719.212 |
| TCGA_TCGA-AA-3511 | 1687.454 | 2383.587 | 4071.04 |
| TCGA_TCGA-A6-A56B | 973.0847 | 1335.443 | 2308.528 |
| TCGA_TCGA-A6-5667 | 1126.461 | 1841.519 | 2967.98 |
| TCGA_TCGA-AA-3866 | 2402.175 | 4523.557 | 6925.732 |
| TCGA_TCGA-AA-A017 | 780.727 | 2315.393 | 3096.12 |
| TCGA_TCGA-CM-6674 | 1542.652 | 3203.388 | 4746.039 |
| TCGA_TCGA-DM-A1D8 | -831.452 | 821.3428 | -10.1093 |
| TCGA_TCGA-CM-6170 | 1549.019 | 2970.776 | 4519.795 |
| TCGA_TCGA-AZ-4616 | 593.2943 | 2766.679 | 3359.974 |
| TCGA_TCGA-AA-3994 | 992.3296 | 2718.018 | 3710.347 |
| TCGA_TCGA-AA-3877 | 1920.689 | 4052.815 | 5973.504 |
| TCGA_TCGA-WS-AB45 | 4173.599 | 5298.678 | 9472.278 |
| TCGA_TCGA-AA-A00L | -527.085 | 1208.661 | 681.5755 |
| TCGA_TCGA-CM-5864 | 74.44321 | 2286.375 | 2360.818 |
| TCGA_TCGA-CA-5254 | 951.6227 | 1867.475 | 2819.098 |
| TCGA_TCGA-D5-6898 | 1985.3 | 3263.295 | 5248.595 |
| TCGA_TCGA-AA-3696 | 404.0412 | 1416.41 | 1820.451 |
| TCGA_TCGA-DM-A280 | 209.8142 | 1832.088 | 2041.902 |
| TCGA_TCGA-AA-A00O | 1745.459 | 2565.395 | 4310.854 |
| TCGA_TCGA-QG-A5YW | 750.1095 | 3171.409 | 3921.518 |
| TCGA_TCGA-AD-6965 | 397.5032 | 1690.867 | 2088.371 |
| TCGA_TCGA-CK-4948 | 1504.864 | 2797.137 | 4302.001 |
| TCGA_TCGA-DM-A1HA | -2411.12 | 826.7402 | -1584.38 |
| TCGA_TCGA-5M-AAT5 | -1139.41 | 823.8307 | -315.576 |
| TCGA_TCGA-F4-6809 | 2204.225 | 3605.262 | 5809.487 |
| TCGA_TCGA-CM-4748 | 1191.41 | 2631.055 | 3822.465 |
| TCGA_TCGA-AA-3858 | 1164.996 | 2755.471 | 3920.467 |
| TCGA_TCGA-D5-6531 | 1647.841 | 3296.706 | 4944.547 |
| TCGA_TCGA-AA-3662 | 1330.35 | 3538.403 | 4868.753 |
| TCGA_TCGA-AM-5820 | 980.8505 | 1773.92 | 2754.771 |
| TCGA_TCGA-AA-A02K | -714.314 | 1260.78 | 546.4664 |
| TCGA_TCGA-G4-6310 | 567.3417 | 342.7081 | 910.0498 |
| TCGA_TCGA-AD-6889 | -148.464 | 2001.844 | 1853.381 |
| TCGA_TCGA-AA-3872 | 2824.076 | 3645.571 | 6469.647 |
| TCGA_TCGA-AA-A01R | 525.6744 | 4200.959 | 4726.633 |
| TCGA_TCGA-AA-3975 | 1118.94 | 2988.941 | 4107.88 |
| TCGA_TCGA-5M-AAT6 | 2317.222 | 4397.217 | 6714.439 |
| TCGA_TCGA-D5-6931 | 1430.365 | 3448.87 | 4879.235 |
| TCGA_TCGA-D5-6924 | 2565.603 | 3900.785 | 6466.388 |
| TCGA_TCGA-AA-3860 | 1801.216 | 3699.042 | 5500.258 |
| TCGA_TCGA-A6-5665 | 397.7832 | 2806.996 | 3204.779 |
| TCGA_TCGA-5M-AATA | 722.0074 | 2528.475 | 3250.482 |
| TCGA_TCGA-D5-6540 | 500.1612 | 2863.982 | 3364.144 |
| TCGA_TCGA-CA-6718 | 1573.506 | 4416.19 | 5989.696 |
| TCGA_TCGA-AA-3986 | 1116.471 | 4494.548 | 5611.019 |
| TCGA_TCGA-G4-6323 | -1287.03 | 3403.868 | 2116.835 |
| TCGA_TCGA-A6-A566 | 3848.564 | 4710.896 | 8559.46 |
| TCGA_TCGA-AZ-6608 | -2444.58 | 700.4422 | -1744.13 |
| TCGA_TCGA-AA-3712 | 1446.777 | 2473.154 | 3919.931 |
| TCGA_TCGA-A6-5662 | 681.8407 | 1321.609 | 2003.449 |
| TCGA_TCGA-AA-3831 | 912.0697 | 3261.168 | 4173.238 |
| TCGA_TCGA-AA-A02H | 306.6763 | 1806.497 | 2113.173 |
| TCGA_TCGA-CM-6676 | 962.135 | 1393.719 | 2355.854 |
| TCGA_TCGA-AD-6899 | 2510.625 | 4024.94 | 6535.565 |
| TCGA_TCGA-AA-3685 | 1423.659 | 3971.889 | 5395.548 |
| TCGA_TCGA-CK-5915 | -725.359 | 450.6736 | -274.685 |
| TCGA_TCGA-AA-3548 | 933.3023 | 3200.1 | 4133.402 |
| TCGA_TCGA-F4-6569 | 3390.453 | 3957.79 | 7348.243 |
| TCGA_TCGA-F4-6461 | 1797.114 | 3078.412 | 4875.527 |
| TCGA_TCGA-F4-6854 | 1505.143 | 2691.685 | 4196.828 |
| TCGA_TCGA-F4-6856 | 368.4283 | 2376.601 | 2745.029 |
| TCGA_TCGA-AY-A54L | -2530.85 | 364.5619 | -2166.29 |
| TCGA_TCGA-AA-3538 | 1308.695 | 2435.854 | 3744.549 |
| TCGA_TCGA-AA-3842 | 1689.016 | 2776.626 | 4465.642 |
| TCGA_TCGA-AZ-4308 | 1640.426 | 2770.541 | 4410.967 |
| TCGA_TCGA-CK-6746 | -17.7898 | 3817.905 | 3800.115 |
| TCGA_TCGA-AA-3509 | 978.5283 | 3006.326 | 3984.855 |
| TCGA_TCGA-AA-3680 | 287.1626 | 3029.347 | 3316.509 |
| TCGA_TCGA-AA-3542 | -20.5436 | 1942.727 | 1922.183 |
| TCGA_TCGA-AD-6964 | 2961.864 | 4982.008 | 7943.872 |
| TCGA_TCGA-A6-6650 | -146.828 | 1346.89 | 1200.062 |
| TCGA_TCGA-A6-6140 | -517.366 | 2261.022 | 1743.656 |
| TCGA_TCGA-AA-A024 | -899.315 | 1671.815 | 772.5003 |
| TCGA_TCGA-A6-2682 | 1729.799 | 3236.338 | 4966.137 |
| TCGA_TCGA-CM-6675 | -14.7863 | 1569.793 | 1555.007 |
| TCGA_TCGA-DM-A1D7 | -83.1862 | 1777.889 | 1694.702 |
| TCGA_TCGA-4T-AA8H | -2226.28 | 679.892 | -1546.38 |
| TCGA_TCGA-A6-6653 | 818.3128 | 2756.581 | 3574.894 |
| TCGA_TCGA-AA-3666 | 632.9244 | 3228.353 | 3861.278 |
| TCGA_TCGA-F4-6460 | 1836.321 | 2865.712 | 4702.033 |
| TCGA_TCGA-AA-3655 | 1134.427 | 2625.581 | 3760.007 |
| TCGA_TCGA-AA-3673 | 845.4009 | 2607.861 | 3453.262 |
| TCGA_TCGA-AA-3952 | 1568.019 | 2567.057 | 4135.076 |
| TCGA_TCGA-T9-A92H | -985.485 | 1168.484 | 182.9985 |
| TCGA_TCGA-AY-4070 | 750.6391 | 2773.504 | 3524.143 |
| TCGA_TCGA-AY-A8YK | -326.91 | 1747.414 | 1420.504 |
| TCGA_TCGA-CK-4950 | 1003.805 | 3759.457 | 4763.262 |
| TCGA_TCGA-F4-6570 | 2417.554 | 4601.802 | 7019.356 |
| TCGA_TCGA-AZ-6600 | 2107.906 | 3399.469 | 5507.375 |
| TCGA_TCGA-AA-A00J | 1191.432 | 2606.363 | 3797.795 |
| TCGA_TCGA-AA-3667 | 463.4947 | 2926.405 | 3389.899 |
| TCGA_TCGA-AA-3554 | 2290.405 | 4521.426 | 6811.831 |
| TCGA_TCGA-AZ-6606 | -1050.24 | 1563.577 | 513.3412 |
| TCGA_TCGA-AA-3852 | 1628.042 | 3403.808 | 5031.85 |
| TCGA_TCGA-D5-6922 | 2156.725 | 2661.371 | 4818.096 |
| TCGA_TCGA-A6-6137 | 420.6082 | 3294.494 | 3715.102 |
| TCGA_TCGA-AA-3495 | 630.1224 | 3151.877 | 3782 |
| TCGA_TCGA-AA-3489 | 3565.905 | 5014.529 | 8580.435 |
| TCGA_TCGA-G4-6322 | 204.5799 | 2191.05 | 2395.63 |
| TCGA_TCGA-SS-A7HO | -924.445 | 459.0304 | -465.414 |
| TCGA_TCGA-AA-3976 | 1001.449 | 2441.132 | 3442.581 |
| TCGA_TCGA-AA-3811 | 1064.523 | 3212.61 | 4277.134 |
| TCGA_TCGA-AD-A5EJ | 316.6013 | 2076.53 | 2393.131 |
| TCGA_TCGA-AA-3819 | 249.4925 | 2293.317 | 2542.81 |
| TCGA_TCGA-AA-A02Y | -2278.33 | 2367.158 | 88.82392 |
| TCGA_TCGA-AZ-6603 | 1813.128 | 3202.338 | 5015.466 |
| TCGA_TCGA-AA-A022 | 1076.969 | 3958.578 | 5035.547 |
| TCGA_TCGA-G4-6626 | -1096.18 | 980.0631 | -116.117 |
| TCGA_TCGA-AA-3875 | 1188.686 | 3642.64 | 4831.326 |
| TCGA_TCGA-G4-6306 | -2071.66 | 1856.464 | -215.2 |
| TCGA_TCGA-G4-6320 | -1254.39 | 1645.911 | 391.5189 |
| TCGA_TCGA-CK-6748 | 2454.139 | 2955.624 | 5409.762 |
| TCGA_TCGA-AA-3864 | 1211.068 | 2626.841 | 3837.91 |
| TCGA_TCGA-A6-6142 | 2553.148 | 2885.26 | 5438.408 |
| TCGA_TCGA-A6-2678 | 1180.03 | 3078.655 | 4258.686 |
| TCGA_TCGA-CA-5796 | -293.753 | 2735.06 | 2441.307 |
| TCGA_TCGA-A6-A567 | 321.7737 | 1195.08 | 1516.854 |
| TCGA_TCGA-AA-3713 | 780.9171 | 3788.799 | 4569.717 |
| TCGA_TCGA-A6-6648 | -344.421 | 1715.448 | 1371.027 |
| TCGA_TCGA-A6-A565 | 2070.449 | 4740.829 | 6811.278 |
| TCGA_TCGA-A6-5660 | 1296.021 | 2434.957 | 3730.977 |
| TCGA_TCGA-AZ-6601 | 1996.317 | 4288.682 | 6284.999 |
| TCGA_TCGA-5M-AATE | -214.513 | 1478.712 | 1264.199 |
| TCGA_TCGA-CM-6161 | 1294.516 | 3343.251 | 4637.767 |
| TCGA_TCGA-AA-A01Q | -219.934 | 3063.349 | 2843.415 |
| TCGA_TCGA-AZ-4313 | -306.184 | 1599.377 | 1293.194 |
| TCGA_TCGA-A6-6652 | -731.668 | 799.7453 | 68.07693 |
| TCGA_TCGA-AA-A00E | 937.1495 | 3815.868 | 4753.018 |
| TCGA_TCGA-F4-6808 | -697.247 | 1133.605 | 436.3581 |
| TCGA_TCGA-AA-A02W | 11.29421 | 1476.159 | 1487.453 |
| TCGA_TCGA-DM-A28C | -1165.78 | 428.2155 | -737.56 |
| TCGA_TCGA-D5-6927 | 1878.918 | 4093.831 | 5972.748 |
| TCGA_TCGA-AA-3980 | 1025.973 | 3906.046 | 4932.019 |
| TCGA_TCGA-AA-3681 | 686.1797 | 3596.145 | 4282.325 |
| TCGA_TCGA-AA-3492 | 1064.853 | 3118.115 | 4182.968 |
| TCGA_TCGA-A6-2671 | 2064.012 | 2896.002 | 4960.013 |
| TCGA_TCGA-D5-6535 | 1014.36 | 3224.91 | 4239.27 |
| TCGA_TCGA-AA-A029 | -1040.3 | 1429.489 | 389.1877 |
| TCGA_TCGA-AD-6963 | 65.45473 | 3274.096 | 3339.55 |
| TCGA_TCGA-CM-4747 | 837.749 | 2134.926 | 2972.675 |
| TCGA_TCGA-NH-A50T | -791.117 | 513.1623 | -277.954 |
| TCGA_TCGA-CM-6168 | 3036.63 | 4060.216 | 7096.845 |
| TCGA_TCGA-AA-3518 | 504.0874 | 3602.909 | 4106.996 |
| TCGA_TCGA-AA-A01T | -879.07 | 1550.271 | 671.2018 |
| TCGA_TCGA-RU-A8FL | -2971.53 | 77.5076 | -2894.02 |
| TCGA_TCGA-A6-6651 | 3559.367 | 4679.781 | 8239.147 |
| TCGA_TCGA-AA-3850 | 1431.253 | 3924.904 | 5356.157 |
| TCGA_TCGA-AA-3972 | 668.5617 | 1932.515 | 2601.076 |
| TCGA_TCGA-AA-3812 | 1921.797 | 3300.514 | 5222.311 |
| TCGA_TCGA-AY-A69D | -83.5236 | 1733.608 | 1650.084 |
| TCGA_TCGA-AA-A01Z | -782.231 | 729.2577 | -52.9732 |
| TCGA_TCGA-A6-3810 | 2557.7 | 3429.918 | 5987.619 |
| TCGA_TCGA-DM-A28H | -674.1 | 633.1017 | -40.9979 |
| TCGA_TCGA-G4-6586 | -1090.35 | 2965.002 | 1874.653 |
| TCGA_TCGA-D5-6928 | 3227.131 | 6079.087 | 9306.217 |
| TCGA_TCGA-G4-6315 | -1362.78 | 901.2277 | -461.551 |
| TCGA_TCGA-D5-6534 | 3821.028 | 5315.346 | 9136.374 |
| TCGA_TCGA-AA-3664 | -244.321 | 2338.425 | 2094.104 |
| TCGA_TCGA-G4-6303 | 1522.28 | 2395.456 | 3917.735 |
| TCGA_TCGA-AA-3692 | 1227.38 | 3554.437 | 4781.817 |
| TCGA_TCGA-AA-3968 | 1721.743 | 3171.924 | 4893.667 |
| TCGA_TCGA-F4-6805 | 2754.67 | 3823.885 | 6578.554 |
| TCGA_TCGA-AA-3560 | 1149.123 | 3172.234 | 4321.357 |
| TCGA_TCGA-D5-5537 | 921.128 | 1784.334 | 2705.462 |
| TCGA_TCGA-CM-5862 | 857.4357 | 905.1589 | 1762.595 |
| TCGA_TCGA-D5-6932 | 1478.42 | 2774.313 | 4252.733 |
| TCGA_TCGA-AA-3982 | 1636.505 | 3907.615 | 5544.121 |
| TCGA_TCGA-DM-A28A | 699.0388 | 1438.763 | 2137.801 |
| TCGA_TCGA-CM-6172 | 1102.785 | 2272.289 | 3375.074 |
| TCGA_TCGA-D5-6930 | 2016.206 | 3824.513 | 5840.719 |
| TCGA_TCGA-D5-6529 | 2498.745 | 4432.323 | 6931.068 |
| TCGA_TCGA-A6-2683 | -391.697 | 1578.264 | 1186.567 |
| TCGA_TCGA-CK-5913 | 1256.162 | 3320.468 | 4576.63 |
| TCGA_TCGA-AA-3977 | 1228.862 | 3453.153 | 4682.016 |
| TCGA_TCGA-AA-3496 | 2226.806 | 4027.348 | 6254.154 |
| TCGA_TCGA-D5-5540 | 197.8757 | 1987.642 | 2185.518 |
| TCGA_TCGA-AD-6901 | 2611.13 | 3639.496 | 6250.626 |
| TCGA_TCGA-AA-A01F | -1827.63 | 1161.61 | -666.017 |
| TCGA_TCGA-AA-3552 | 952.7602 | 3942.985 | 4895.745 |
| TCGA_TCGA-F4-6855 | 2793.671 | 2816.852 | 5610.524 |
| TCGA_TCGA-AD-5900 | 1660.467 | 3726.627 | 5387.094 |
| TCGA_TCGA-AA-3979 | -588.735 | 1425.326 | 836.5917 |
| TCGA_TCGA-AA-A01I | -341.85 | 2200.711 | 1858.861 |
| TCGA_TCGA-AA-A03F | -580.386 | 2740.064 | 2159.677 |
| TCGA_TCGA-3L-AA1B | 885.0309 | 2603.231 | 3488.262 |
| TCGA_TCGA-AA-3693 | 103.3909 | 1970.322 | 2073.713 |
| TCGA_TCGA-AA-3502 | -908.231 | 2892.874 | 1984.642 |
| TCGA_TCGA-AA-A02E | -348.588 | 1891.812 | 1543.224 |
| TCGA_TCGA-CM-4746 | -953.093 | 1772.114 | 819.0209 |
| TCGA_TCGA-G4-6295 | 587.3834 | 3617.288 | 4204.672 |
| TCGA_TCGA-AA-A01C | 1278.057 | 2150.4 | 3428.457 |
| TCGA_TCGA-G4-6588 | 647.4288 | 2753.027 | 3400.456 |
| TCGA_TCGA-A6-2680 | 994.5249 | 2906.353 | 3900.878 |
| TCGA_TCGA-QG-A5YV | -575.008 | 1780.684 | 1205.676 |
| TCGA_TCGA-AA-3561 | 177.7414 | 2238.771 | 2416.512 |
| TCGA_TCGA-AA-3525 | 59.60106 | 2937.938 | 2997.539 |
| TCGA_TCGA-AA-3531 | -254.976 | 1641.627 | 1386.651 |
| TCGA_TCGA-AA-3973 | 626.5191 | 1658.122 | 2284.641 |
| TCGA_TCGA-AA-3527 | 1680.288 | 3250.041 | 4930.329 |
| TCGA_TCGA-AA-3555 | 1346.546 | 2635.009 | 3981.555 |
| TCGA_TCGA-AG-A025 | 456.3201 | 1902.393 | 2358.713 |
| TCGA_TCGA-EI-6884 | 1775.404 | 3578.455 | 5353.859 |
| TCGA_TCGA-AG-3582 | 1239.333 | 2891.807 | 4131.14 |
| TCGA_TCGA-DC-6154 | 430.9653 | 1907.971 | 2338.936 |
| TCGA_TCGA-AG-3611 | -323.544 | 2871.406 | 2547.862 |
| TCGA_TCGA-DY-A1DD | -271.31 | 843.7467 | 572.4364 |
| TCGA_TCGA-AG-3609 | 2035.696 | 3337.303 | 5372.999 |
| TCGA_TCGA-AG-A01L | -561.915 | 1510.733 | 948.8181 |
| TCGA_TCGA-AG-4022 | 2095.124 | 3602.561 | 5697.684 |
| TCGA_TCGA-AG-A01W | -271.009 | 2328.71 | 2057.7 |
| TCGA_TCGA-AG-A02N | -799.262 | 2666.402 | 1867.141 |
| TCGA_TCGA-AH-6549 | 1568.438 | 2256.484 | 3824.922 |
| TCGA_TCGA-AH-6903 | -830.079 | 1481.889 | 651.8097 |
| TCGA_TCGA-AG-3727 | 1052.495 | 2342.856 | 3395.35 |
| TCGA_TCGA-F5-6863 | 226.4026 | 659.8756 | 886.2782 |
| TCGA_TCGA-AG-3887 | 638.5283 | 2884.971 | 3523.5 |
| TCGA_TCGA-AG-A01J | -753.541 | 1806.238 | 1052.697 |
| TCGA_TCGA-AF-5654 | -483.82 | 1155.831 | 672.0105 |
| TCGA_TCGA-DY-A1DE | 960.7467 | 2536.697 | 3497.443 |
| TCGA_TCGA-AF-3913 | 1422.394 | 2014.576 | 3436.97 |
| TCGA_TCGA-AG-A014 | -363.777 | 1235.336 | 871.5597 |
| TCGA_TCGA-AG-4015 | 222.1797 | 2091.335 | 2313.515 |
| TCGA_TCGA-G5-6572 | 1684.676 | 2637.997 | 4322.673 |
| TCGA_TCGA-AG-A011 | 535.1098 | 2202.383 | 2737.492 |
| TCGA_TCGA-EI-6511 | 2207.7 | 4583.36 | 6791.06 |
| TCGA_TCGA-AG-4008 | 2020.998 | 2938.955 | 4959.953 |
| TCGA_TCGA-AG-3731 | 2906.458 | 4322.208 | 7228.666 |
| TCGA_TCGA-AG-A032 | -24.6487 | 1962.326 | 1937.677 |
| TCGA_TCGA-AH-6544 | -1631.55 | 358.1044 | -1273.44 |
| TCGA_TCGA-AG-A020 | -1579.39 | 1000.469 | -578.922 |
| TCGA_TCGA-AG-A026 | 238.3269 | 864.0405 | 1102.367 |
| TCGA_TCGA-DY-A1H8 | -2349.25 | 766.0475 | -1583.21 |
| TCGA_TCGA-AG-3883 | 2466.322 | 3762.565 | 6228.887 |
| TCGA_TCGA-EI-7004 | 3877.385 | 4003.307 | 7880.692 |
| TCGA_TCGA-AF-6136 | 365.742 | 2173.751 | 2539.493 |
| TCGA_TCGA-DC-4745 | 947.2591 | 2426.11 | 3373.37 |
| TCGA_TCGA-EI-6883 | 278.7865 | 2532.428 | 2811.215 |
| TCGA_TCGA-EI-6917 | 2431.175 | 4411.135 | 6842.31 |
| TCGA_TCGA-F5-6861 | 406.6331 | 1832.697 | 2239.33 |
| TCGA_TCGA-EI-6508 | 508.6755 | 1725.091 | 2233.766 |
| TCGA_TCGA-F5-6465 | 2825.739 | 4136.934 | 6962.672 |
| TCGA_TCGA-AF-6672 | -122.629 | 2028.197 | 1905.568 |
| TCGA_TCGA-AG-3885 | 511.4897 | 3046.82 | 3558.309 |
| TCGA_TCGA-EI-6509 | 666.6961 | 1245.447 | 1912.143 |
| TCGA_TCGA-DC-5337 | 458.022 | 2906.943 | 3364.965 |
| TCGA_TCGA-AG-3893 | 1655.238 | 2719.871 | 4375.109 |
| TCGA_TCGA-AG-A02G | -1114.96 | 1663.407 | 548.4512 |
| TCGA_TCGA-AG-3901 | 3165.343 | 4434.386 | 7599.73 |
| TCGA_TCGA-AG-3726 | 757.0143 | 2486.986 | 3244 |
| TCGA_TCGA-AG-3896 | 1350.395 | 2840.203 | 4190.598 |
| TCGA_TCGA-DC-6160 | 134.5503 | 3140.759 | 3275.309 |
| TCGA_TCGA-AG-3581 | 1026.459 | 3070.537 | 4096.996 |
| TCGA_TCGA-G5-6641 | -2546.09 | 202.1857 | -2343.9 |
| TCGA_TCGA-F5-6812 | 2514.923 | 3574.422 | 6089.345 |
| TCGA_TCGA-AG-4001 | 2417.382 | 3247.19 | 5664.572 |
| TCGA_TCGA-AF-2687 | 3291.448 | 4211.344 | 7502.792 |
| TCGA_TCGA-AH-6644 | 2236.434 | 2968.907 | 5205.341 |
| TCGA_TCGA-AG-3586 | 405.1782 | 2680.804 | 3085.982 |
| TCGA_TCGA-AG-3909 | 844.9616 | 2695.838 | 3540.8 |
| TCGA_TCGA-AG-3878 | 2337.043 | 4067.926 | 6404.969 |
| TCGA_TCGA-AG-3592 | 891.5194 | 2671.703 | 3563.222 |
| TCGA_TCGA-F5-6814 | 1185.542 | 3070.641 | 4256.183 |
| TCGA_TCGA-AG-3605 | -461.117 | 2741.003 | 2279.887 |
| TCGA_TCGA-EI-6513 | 771.7123 | 2244.396 | 3016.108 |
| TCGA_TCGA-DC-6158 | 2829.741 | 3791.496 | 6621.237 |
| TCGA_TCGA-EI-6512 | 459.0784 | 2585.287 | 3044.366 |
| TCGA_TCGA-AG-3601 | 795.3596 | 1880.513 | 2675.872 |
| TCGA_TCGA-CI-6622 | -145.523 | 1423.871 | 1278.348 |
| TCGA_TCGA-BM-6198 | 1851.652 | 3948.018 | 5799.671 |
| TCGA_TCGA-AG-3881 | 2620.775 | 4042.402 | 6663.177 |
| TCGA_TCGA-DY-A1DC | 1002.012 | 2175.7 | 3177.711 |
| TCGA_TCGA-AG-3612 | 1720.782 | 3120.522 | 4841.304 |
| TCGA_TCGA-AG-3600 | 887.4259 | 3140.328 | 4027.754 |
| TCGA_TCGA-AG-3599 | 169.9977 | 3061.699 | 3231.697 |
| TCGA_TCGA-AG-3584 | 1348.83 | 2984.903 | 4333.733 |
| TCGA_TCGA-DC-6156 | 3130.952 | 4487.289 | 7618.241 |
| TCGA_TCGA-DT-5265 | 2248.445 | 2999.126 | 5247.572 |
| TCGA_TCGA-AG-3902 | 676.3596 | 4212.628 | 4888.987 |
| TCGA_TCGA-AG-A036 | 831.9229 | 2250.488 | 3082.411 |
| TCGA_TCGA-AG-3593 | 680.7825 | 3562.117 | 4242.9 |
| TCGA_TCGA-AG-A01Y | 960.5941 | 2884.842 | 3845.436 |
| TCGA_TCGA-AG-3580 | 175.4924 | 3214.842 | 3390.335 |
| TCGA_TCGA-AG-3742 | 1176.282 | 1847.141 | 3023.423 |
| TCGA_TCGA-AG-A002 | -842.032 | 469.7939 | -372.239 |
| TCGA_TCGA-DC-6683 | 1082.093 | 2020.517 | 3102.611 |
| TCGA_TCGA-AF-6655 | 1951.154 | 2425.767 | 4376.921 |
| TCGA_TCGA-AG-A023 | 1319.862 | 2632.113 | 3951.975 |
| TCGA_TCGA-AG-3728 | 2176.096 | 4154.942 | 6331.038 |
| TCGA_TCGA-AF-3400 | 3324.79 | 5188.191 | 8512.982 |
| TCGA_TCGA-AF-2691 | 1457.993 | 3583.675 | 5041.668 |
| TCGA_TCGA-CL-5918 | -917.033 | 1894.909 | 977.8767 |
| TCGA_TCGA-F5-6864 | 2435.162 | 3121.335 | 5556.497 |
| TCGA_TCGA-AF-A56K | 2049.775 | 3204.659 | 5254.434 |
| TCGA_TCGA-AG-4021 | 1870.206 | 2260.458 | 4130.664 |
| TCGA_TCGA-AG-A01N | -271.078 | 962.095 | 691.0171 |
| TCGA_TCGA-DC-5869 | 1269.985 | 2136.43 | 3406.415 |
| TCGA_TCGA-AG-3608 | 863.3602 | 3559.198 | 4422.558 |
| TCGA_TCGA-DY-A0XA | 120.3552 | 1528.971 | 1649.326 |
| TCGA_TCGA-AG-3594 | 782.2695 | 4422.354 | 5204.623 |
| TCGA_TCGA-EF-5831 | 1452.446 | 2381.153 | 3833.599 |
| TCGA_TCGA-AG-3725 | -495.849 | 2107.466 | 1611.616 |
| TCGA_TCGA-EI-6514 | 1462.277 | 2241.152 | 3703.429 |
| TCGA_TCGA-CI-6624 | 2034.778 | 3544.129 | 5578.907 |
| TCGA_TCGA-CI-6620 | 1082.116 | 2236.515 | 3318.631 |
| TCGA_TCGA-CI-6621 | 1777.885 | 3555.411 | 5333.296 |
| TCGA_TCGA-EI-6510 | -1527.74 | 2122.37 | 594.6326 |
| TCGA_TCGA-DY-A1DG | -2012.24 | 756.6397 | -1255.6 |
| TCGA_TCGA-AG-A02X | -1129.57 | 1223.661 | 94.08731 |
| TCGA_TCGA-AG-3587 | 900.2572 | 2548.373 | 3448.63 |
| TCGA_TCGA-AG-3894 | 475.6566 | 2217.429 | 2693.086 |
| TCGA_TCGA-AG-3999 | 736.9722 | 1921.416 | 2658.388 |
| TCGA_TCGA-AG-A00H | 1315.161 | 1804.918 | 3120.08 |
| TCGA_TCGA-AF-A56N | 1179.574 | 2052.732 | 3232.306 |
| TCGA_TCGA-AG-A00C | -1585.95 | 1909.45 | 323.4997 |
| TCGA_TCGA-EI-6885 | 1980.839 | 3310.635 | 5291.475 |
| TCGA_TCGA-AG-3732 | 451.3526 | 3664.841 | 4116.193 |
| TCGA_TCGA-AG-4007 | 1644.701 | 3522.176 | 5166.877 |
| TCGA_TCGA-AG-A016 | -882.333 | 325.5527 | -556.78 |
| TCGA_TCGA-EI-6882 | 556.6349 | 2790.406 | 3347.041 |
| TCGA_TCGA-AG-3575 | 2002.812 | 3482 | 5484.812 |
| TCGA_TCGA-AF-2693 | 1219.607 | 3172.715 | 4392.322 |
| TCGA_TCGA-DC-6157 | 655.3221 | 1841.237 | 2496.559 |
| TCGA_TCGA-AF-4110 | 2120.739 | 4034.43 | 6155.169 |
| TCGA_TCGA-AG-3591 | 65.18597 | 2578.847 | 2644.033 |
| TCGA_TCGA-EI-6881 | -305.912 | 2347.896 | 2041.983 |
| TCGA_TCGA-AG-A008 | -1542.13 | 1589.816 | 47.68193 |
| TCGA_TCGA-AG-A015 | -998.052 | 1268.777 | 270.7243 |
| TCGA_TCGA-AG-3892 | 459.1569 | 4080.22 | 4539.377 |
| TCGA_TCGA-G5-6233 | 705.6331 | 2251.482 | 2957.115 |
| TCGA_TCGA-AG-3898 | 1442.38 | 3349 | 4791.38 |
| TCGA_TCGA-AG-3578 | 717.6296 | 2941.985 | 3659.614 |
| TCGA_TCGA-DC-6682 | -221.5 | 1702.516 | 1481.016 |
| TCGA_TCGA-F5-6813 | 1654.562 | 3082.237 | 4736.799 |
| TCGA_TCGA-F5-6702 | 3401.84 | 3479.465 | 6881.304 |
| TCGA_TCGA-CL-5917 | 498.1766 | 1234.703 | 1732.88 |
| TCGA_TCGA-CL-4957 | 376.8208 | 1518.504 | 1895.325 |
| TCGA_TCGA-EI-6507 | 1787.366 | 4030.882 | 5818.248 |
| TCGA_TCGA-DC-4749 | 39.90129 | 1591.63 | 1631.532 |
| TCGA_TCGA-AF-2690 | 3937.58 | 5166.578 | 9104.158 |
| TCGA_TCGA-EF-5830 | -92.7822 | 2031.989 | 1939.207 |
| TCGA_TCGA-AF-3911 | 1377.28 | 2529.939 | 3907.219 |
| TCGA_TCGA-DC-6155 | 186.9018 | 2424.085 | 2610.986 |
| TCGA_TCGA-DC-6681 | 1692.755 | 2425.733 | 4118.488 |
| TCGA_TCGA-AF-A56L | 799.2387 | 1799.356 | 2598.595 |
| TCGA_TCGA-CI-6623 | 523.4065 | 2509.402 | 3032.808 |
| TCGA_TCGA-AH-6897 | -742.594 | 1175.662 | 433.0677 |
| TCGA_TCGA-AG-3574 | 572.7673 | 2402.24 | 2975.007 |
| TCGA_TCGA-G5-6235 | -1786.19 | 1090.695 | -695.499 |
| TCGA_TCGA-AG-4005 | 1309.404 | 2199.056 | 3508.46 |
| TCGA_TCGA-AG-3583 | -144.448 | 2959.556 | 2815.108 |
| TCGA_TCGA-AH-6547 | 2596.678 | 4850.653 | 7447.331 |
| TCGA_TCGA-EI-7002 | 1549.13 | 2406.304 | 3955.434 |
| TCGA_TCGA-AG-A00Y | -412.946 | 2677.573 | 2264.627 |
| TCGA_TCGA-EI-6506 | 669.0039 | 3363.916 | 4032.92 |
| TCGA_TCGA-AH-6643 | 613.2699 | 1864.523 | 2477.793 |
| TCGA_TCGA-AG-3890 | 1103.829 | 3587.081 | 4690.91 |
| TCGA_TCGA-DY-A1DF | 153.389 | 775.7574 | 929.1464 |
| TCGA_TCGA-F5-6571 | 2409.289 | 4019.215 | 6428.504 |
| TCGA_TCGA-AG-3882 | 1470.57 | 4492.975 | 5963.545 |
| TCGA_TCGA-AG-3598 | 441.2988 | 3250.612 | 3691.91 |
| TCGA_TCGA-F5-6811 | 2373.477 | 3091.877 | 5465.354 |
| TCGA_TCGA-F5-6810 | 1959.873 | 2111.646 | 4071.519 |
| TCGA_TCGA-F5-6464 | 3365.654 | 4246.769 | 7612.423 |
| TCGA_TCGA-CI-6619 | 948.8133 | 3144.938 | 4093.751 |
| TCGA_TCGA-AG-3602 | 1330.853 | 3210.405 | 4541.258 |
| TCGA_TCGA-AF-2692 | 768.3579 | 2509.016 | 3277.373 |
| GSE39582_GSM971957 | 1387.434 | 2783.133 | 4170.567 |
| GSE39582_GSM971958 | 2395.849 | 3056.051 | 5451.9 |
| GSE39582_GSM971959 | -777.457 | 1377.612 | 600.155 |
| GSE39582_GSM971960 | -818.187 | 2158.854 | 1340.667 |
| GSE39582_GSM971961 | 3707.078 | 4521.956 | 8229.034 |
| GSE39582_GSM971962 | 3926.594 | 4296.682 | 8223.276 |
| GSE39582_GSM971963 | 2508.544 | 6697.033 | 9205.578 |
| GSE39582_GSM971964 | -185.248 | 2184.95 | 1999.702 |
| GSE39582_GSM971965 | 898.103 | 2161.038 | 3059.141 |
| GSE39582_GSM971966 | 1173.706 | 2279.809 | 3453.515 |
| GSE39582_GSM971967 | -530.293 | 1258.91 | 728.6172 |
| GSE39582_GSM971968 | 2856.422 | 4291.757 | 7148.179 |
| GSE39582_GSM971969 | 911.2419 | 4498.677 | 5409.919 |
| GSE39582_GSM971970 | -688.169 | 1043.57 | 355.4007 |
| GSE39582_GSM971971 | 539.7882 | 4240.133 | 4779.921 |
| GSE39582_GSM971972 | 579.7846 | 2213.362 | 2793.147 |
| GSE39582_GSM971973 | -494.97 | 1096.258 | 601.2884 |
| GSE39582_GSM971974 | 651.6232 | 2161.694 | 2813.318 |
| GSE39582_GSM971975 | -399.93 | 2269.031 | 1869.101 |
| GSE39582_GSM971976 | -549.615 | 619.0735 | 69.459 |
| GSE39582_GSM971977 | 2384.466 | 4067.238 | 6451.704 |
| GSE39582_GSM971978 | 823.4614 | 3067.037 | 3890.498 |
| GSE39582_GSM971979 | 1145.971 | 2828.596 | 3974.567 |
| GSE39582_GSM971980 | 885.0244 | 4038.889 | 4923.914 |
| GSE39582_GSM971981 | 2554.089 | 4283.01 | 6837.099 |
| GSE39582_GSM971982 | 2269.554 | 4385.797 | 6655.351 |
| GSE39582_GSM971983 | 2224.522 | 4926.651 | 7151.173 |
| GSE39582_GSM971984 | 1712.047 | 3635.57 | 5347.616 |
| GSE39582_GSM971985 | -826.36 | 2282.477 | 1456.117 |
| GSE39582_GSM971986 | 1559.92 | 3408.511 | 4968.431 |
| GSE39582_GSM971987 | 2849.583 | 4214.187 | 7063.77 |
| GSE39582_GSM971988 | -74.446 | 1497.846 | 1423.4 |
| GSE39582_GSM971989 | -966.893 | 1058.925 | 92.03225 |
| GSE39582_GSM971990 | 378.562 | 3651.711 | 4030.273 |
| GSE39582_GSM971991 | 1673.939 | 2738.092 | 4412.031 |
| GSE39582_GSM971992 | -866.093 | 2285.281 | 1419.188 |
| GSE39582_GSM971993 | 507.468 | 3207.479 | 3714.947 |
| GSE39582_GSM971994 | 439.8745 | 1156.63 | 1596.505 |
| GSE39582_GSM971995 | 747.1138 | 3145.744 | 3892.858 |
| GSE39582_GSM971996 | 4071.95 | 5494.884 | 9566.833 |
| GSE39582_GSM971997 | 1553.159 | 6116.53 | 7669.688 |
| GSE39582_GSM971998 | 857.0758 | 3090.037 | 3947.113 |
| GSE39582_GSM971999 | 1581.791 | 4067.294 | 5649.085 |
| GSE39582_GSM972000 | 933.383 | 1840.957 | 2774.34 |
| GSE39582_GSM972001 | -647.112 | 2700.701 | 2053.589 |
| GSE39582_GSM972002 | 56.16011 | 1714.184 | 1770.344 |
| GSE39582_GSM972003 | -910.39 | 1518.888 | 608.4979 |
| GSE39582_GSM972004 | 20.07642 | 2110.894 | 2130.97 |
| GSE39582_GSM972005 | -2629.26 | -13.7333 | -2642.99 |
| GSE39582_GSM972006 | 1610.699 | 2674.387 | 4285.086 |
| GSE39582_GSM972007 | 2955.31 | 3950.556 | 6905.866 |
| GSE39582_GSM972008 | 2273.886 | 3698.829 | 5972.715 |
| GSE39582_GSM972009 | 1214.26 | 2605.524 | 3819.785 |
| GSE39582_GSM972010 | 1717.64 | 2878.684 | 4596.324 |
| GSE39582_GSM972011 | 4191.215 | 3686.432 | 7877.647 |
| GSE39582_GSM972012 | -844.538 | 1945.159 | 1100.621 |
| GSE39582_GSM972013 | 739.1146 | 905.946 | 1645.061 |
| GSE39582_GSM972014 | -29.4535 | 1011.575 | 982.121 |
| GSE39582_GSM972015 | -84.2023 | 1665.078 | 1580.876 |
| GSE39582_GSM972016 | 887.3179 | 2593.039 | 3480.357 |
| GSE39582_GSM972017 | 585.4996 | 2886.122 | 3471.621 |
| GSE39582_GSM972018 | 1639.036 | 3913.548 | 5552.584 |
| GSE39582_GSM972019 | 2346.082 | 4650.901 | 6996.982 |
| GSE39582_GSM972020 | 968.0527 | 2827.719 | 3795.772 |
| GSE39582_GSM972021 | -906.167 | 808.3146 | -97.8521 |
| GSE39582_GSM972022 | 676.7599 | 2741.286 | 3418.046 |
| GSE39582_GSM972023 | 461.9961 | 2193.152 | 2655.148 |
| GSE39582_GSM972024 | -972.411 | 1120.967 | 148.5563 |
| GSE39582_GSM972025 | 192.0303 | 2379.385 | 2571.415 |
| GSE39582_GSM972026 | 2386.74 | 3692.694 | 6079.435 |
| GSE39582_GSM972027 | -643.518 | 850.1989 | 206.6809 |
| GSE39582_GSM972028 | 553.1254 | 3442.638 | 3995.763 |
| GSE39582_GSM972029 | 1802.925 | 2490.691 | 4293.617 |
| GSE39582_GSM972030 | 2115.969 | 2260.305 | 4376.274 |
| GSE39582_GSM972031 | 865.6305 | 1486.355 | 2351.986 |
| GSE39582_GSM972032 | 357.5234 | 1822.559 | 2180.082 |
| GSE39582_GSM972033 | -2017.47 | 425.2112 | -1592.26 |
| GSE39582_GSM972034 | -1506.85 | 1178.59 | -328.265 |
| GSE39582_GSM972035 | -1578.02 | 687.5728 | -890.448 |
| GSE39582_GSM972036 | -383.972 | 1534.074 | 1150.102 |
| GSE39582_GSM972037 | -1312.34 | 1825.093 | 512.7533 |
| GSE39582_GSM972038 | -538.656 | 1504.385 | 965.7294 |
| GSE39582_GSM972039 | 3123.723 | 3706.589 | 6830.312 |
| GSE39582_GSM972040 | -172.191 | 1584.696 | 1412.505 |
| GSE39582_GSM972041 | 359.5265 | 1320.742 | 1680.268 |
| GSE39582_GSM972042 | -727.902 | 2270.001 | 1542.099 |
| GSE39582_GSM972043 | -162.662 | 1104.312 | 941.6498 |
| GSE39582_GSM972044 | -2018.06 | 749.7774 | -1268.28 |
| GSE39582_GSM972045 | -819.984 | 1143.337 | 323.3526 |
| GSE39582_GSM972046 | 2034.127 | 3994.75 | 6028.876 |
| GSE39582_GSM972047 | 554.1221 | 971.8564 | 1525.979 |
| GSE39582_GSM972048 | -333.033 | 1261.788 | 928.7554 |
| GSE39582_GSM972049 | -1323.48 | 741.1638 | -582.321 |
| GSE39582_GSM972050 | -882.624 | 1967.741 | 1085.117 |
| GSE39582_GSM972051 | 2875.967 | 2278.968 | 5154.936 |
| GSE39582_GSM972052 | 680.6204 | 1827.097 | 2507.717 |
| GSE39582_GSM972053 | -912.182 | 645.5924 | -266.59 |
| GSE39582_GSM972054 | 2532.4 | 3537.791 | 6070.192 |
| GSE39582_GSM972055 | 931.2242 | 3047.28 | 3978.504 |
| GSE39582_GSM972056 | -166.78 | 1929.533 | 1762.753 |
| GSE39582_GSM972057 | -492.249 | 1822.137 | 1329.888 |
| GSE39582_GSM972058 | 2034.837 | 4649.868 | 6684.705 |
| GSE39582_GSM972059 | 2263.66 | 4664.639 | 6928.298 |
| GSE39582_GSM972060 | 1236.051 | 3243.169 | 4479.22 |
| GSE39582_GSM972061 | 27.75367 | 2273.414 | 2301.167 |
| GSE39582_GSM972062 | 794.3745 | 2623.622 | 3417.997 |
| GSE39582_GSM972063 | 2072.512 | 3328.813 | 5401.325 |
| GSE39582_GSM972064 | 2737.3 | 4241.371 | 6978.671 |
| GSE39582_GSM972065 | 1312.473 | 2863.142 | 4175.615 |
| GSE39582_GSM972066 | 868.1421 | 3151.69 | 4019.832 |
| GSE39582_GSM972067 | 2172.213 | 3315.491 | 5487.704 |
| GSE39582_GSM972068 | 4129.027 | 4664.556 | 8793.583 |
| GSE39582_GSM972069 | 1152.129 | 1948.925 | 3101.054 |
| GSE39582_GSM972070 | 805.8228 | 3212.241 | 4018.064 |
| GSE39582_GSM972071 | 1379.166 | 2224.532 | 3603.698 |
| GSE39582_GSM972072 | 2645.021 | 4687.345 | 7332.366 |
| GSE39582_GSM972073 | 2844.651 | 4217.842 | 7062.493 |
| GSE39582_GSM972074 | 819.2227 | 3398.79 | 4218.012 |
| GSE39582_GSM972075 | 856.8258 | 3191.694 | 4048.52 |
| GSE39582_GSM972076 | 2063.5 | 3994.088 | 6057.588 |
| GSE39582_GSM972077 | 1802.715 | 3629.599 | 5432.314 |
| GSE39582_GSM972078 | 1941.272 | 2418.127 | 4359.399 |
| GSE39582_GSM972079 | 2290.223 | 4541.911 | 6832.134 |
| GSE39582_GSM972080 | 1154.874 | 2731.12 | 3885.994 |
| GSE39582_GSM972081 | 747.4977 | 2333.211 | 3080.708 |
| GSE39582_GSM972082 | 3086.721 | 3696.751 | 6783.472 |
| GSE39582_GSM972083 | 1361.319 | 3544.633 | 4905.952 |
| GSE39582_GSM972084 | 2201.34 | 4439.602 | 6640.942 |
| GSE39582_GSM972085 | 1205.345 | 3433.353 | 4638.698 |
| GSE39582_GSM972086 | -148.616 | 1265.168 | 1116.552 |
| GSE39582_GSM972087 | -737.15 | 2782.627 | 2045.476 |
| GSE39582_GSM972088 | 1882.083 | 3692.565 | 5574.648 |
| GSE39582_GSM972089 | 2699.515 | 3112.956 | 5812.471 |
| GSE39582_GSM972090 | -1558.43 | 562.109 | -996.321 |
| GSE39582_GSM972091 | -1548.3 | 1524.366 | -23.9306 |
| GSE39582_GSM972092 | 2319.581 | 3092.67 | 5412.251 |
| GSE39582_GSM972093 | 1967.244 | 2210.456 | 4177.7 |
| GSE39582_GSM972094 | 1702.287 | 3494.888 | 5197.175 |
| GSE39582_GSM972095 | 1596.739 | 2332.199 | 3928.938 |
| GSE39582_GSM972096 | -1861.37 | 349.5159 | -1511.85 |
| GSE39582_GSM972097 | 2713.343 | 2634.309 | 5347.652 |
| GSE39582_GSM972098 | -1571.62 | 1081.57 | -490.049 |
| GSE39582_GSM972099 | -860.68 | 1121.156 | 260.4762 |
| GSE39582_GSM972100 | -485.072 | 1860.505 | 1375.433 |
| GSE39582_GSM972101 | 282.2746 | 3001.263 | 3283.538 |
| GSE39582_GSM972102 | 2149.78 | 5284.851 | 7434.631 |
| GSE39582_GSM972103 | 1304.451 | 3523.428 | 4827.879 |
| GSE39582_GSM972104 | 3117.788 | 3926.545 | 7044.333 |
| GSE39582_GSM972105 | 125.9072 | 1315.927 | 1441.835 |
| GSE39582_GSM972106 | 323.8725 | 1410.066 | 1733.939 |
| GSE39582_GSM972107 | 750.878 | 3125.736 | 3876.614 |
| GSE39582_GSM972108 | -76.269 | 2500.654 | 2424.385 |
| GSE39582_GSM972109 | -272.726 | 2485.751 | 2213.025 |
| GSE39582_GSM972110 | -308.092 | 2711.1 | 2403.009 |
| GSE39582_GSM972111 | 182.8531 | 2941.518 | 3124.371 |
| GSE39582_GSM972112 | 3057.157 | 4401.87 | 7459.028 |
| GSE39582_GSM972113 | 3665.441 | 4002.412 | 7667.854 |
| GSE39582_GSM972114 | -30.3731 | 2096.608 | 2066.234 |
| GSE39582_GSM972115 | 131.2888 | 1861.592 | 1992.881 |
| GSE39582_GSM972116 | 715.7699 | 2543.463 | 3259.233 |
| GSE39582_GSM972117 | 1449.089 | 5375.821 | 6824.911 |
| GSE39582_GSM972118 | -1110.54 | 956.8685 | -153.67 |
| GSE39582_GSM972119 | 108.8061 | 1246.119 | 1354.925 |
| GSE39582_GSM972120 | 1658.04 | 3520.896 | 5178.936 |
| GSE39582_GSM972121 | 48.26789 | 2891.446 | 2939.714 |
| GSE39582_GSM972122 | -1151.01 | 767.1276 | -383.879 |
| GSE39582_GSM972123 | -1036.77 | 1559.229 | 522.4611 |
| GSE39582_GSM972124 | 849.7991 | 2946.799 | 3796.598 |
| GSE39582_GSM972125 | 1944.385 | 2895.329 | 4839.714 |
| GSE39582_GSM972126 | -1134.64 | 656.3056 | -478.332 |
| GSE39582_GSM972127 | 1407.609 | 2789.547 | 4197.156 |
| GSE39582_GSM972128 | 1722.552 | 3370.912 | 5093.464 |
| GSE39582_GSM972129 | 140.9829 | 2925.179 | 3066.162 |
| GSE39582_GSM972130 | -1019.19 | 1255.623 | 236.4318 |
| GSE39582_GSM972131 | 2793.165 | 3602.321 | 6395.486 |
| GSE39582_GSM972132 | 1018.116 | 1818.275 | 2836.39 |
| GSE39582_GSM972133 | 2283.416 | 3515.388 | 5798.803 |
| GSE39582_GSM972134 | 1180.267 | 3021.89 | 4202.157 |
| GSE39582_GSM972135 | -1803.5 | 683.4756 | -1120.03 |
| GSE39582_GSM972136 | -50.782 | 1888.61 | 1837.828 |
| GSE39582_GSM972137 | -342.785 | 1596.438 | 1253.653 |
| GSE39582_GSM972138 | -708.639 | 1448.077 | 739.4381 |
| GSE39582_GSM972139 | -699.205 | 1281.602 | 582.3974 |
| GSE39582_GSM972140 | 3053.198 | 3355.392 | 6408.591 |
| GSE39582_GSM972141 | 1804.767 | 3142.932 | 4947.699 |
| GSE39582_GSM972142 | 152.0595 | 1800.777 | 1952.837 |
| GSE39582_GSM972143 | 980.3022 | 2513.599 | 3493.901 |
| GSE39582_GSM972144 | -601.154 | 1604.584 | 1003.43 |
| GSE39582_GSM972145 | 620.469 | 2799.585 | 3420.054 |
| GSE39582_GSM972146 | 1725.09 | 2390.289 | 4115.379 |
| GSE39582_GSM972147 | 427.4937 | 1761.932 | 2189.426 |
| GSE39582_GSM972148 | 461.1834 | 2232.124 | 2693.307 |
| GSE39582_GSM972149 | -480.968 | 1597.493 | 1116.525 |
| GSE39582_GSM972150 | 597.877 | 2854.096 | 3451.973 |
| GSE39582_GSM972151 | -950.496 | 1811.971 | 861.4746 |
| GSE39582_GSM972152 | -652.993 | 1640.6 | 987.6066 |
| GSE39582_GSM972153 | 470.0112 | 2510.727 | 2980.738 |
| GSE39582_GSM972154 | -224.395 | 1670.737 | 1446.342 |
| GSE39582_GSM972155 | -970.446 | 971.0082 | 0.562632 |
| GSE39582_GSM972156 | 535.2631 | 2147.84 | 2683.103 |
| GSE39582_GSM972157 | 964.7453 | 2007.584 | 2972.33 |
| GSE39582_GSM972158 | 2728.277 | 4560.918 | 7289.196 |
| GSE39582_GSM972159 | -15.1019 | 1901.221 | 1886.12 |
| GSE39582_GSM972160 | 799.8759 | 2905.061 | 3704.937 |
| GSE39582_GSM972161 | -243.199 | 870.39 | 627.1914 |
| GSE39582_GSM972162 | 1539.58 | 3106.078 | 4645.659 |
| GSE39582_GSM972163 | 542.4047 | 2648.971 | 3191.376 |
| GSE39582_GSM972164 | 1626.853 | 2599.488 | 4226.342 |
| GSE39582_GSM972165 | 1820.633 | 2646.739 | 4467.372 |
| GSE39582_GSM972166 | 395.1983 | 1869.572 | 2264.77 |
| GSE39582_GSM972167 | 613.9316 | 3349.56 | 3963.491 |
| GSE39582_GSM972168 | 1502.066 | 3516.367 | 5018.433 |
| GSE39582_GSM972169 | -220.059 | 2142.642 | 1922.583 |
| GSE39582_GSM972170 | 1486.534 | 2849.537 | 4336.071 |
| GSE39582_GSM972171 | 2290.627 | 5282.296 | 7572.923 |
| GSE39582_GSM972172 | -1153.13 | 1570.454 | 417.3237 |
| GSE39582_GSM972173 | 65.96357 | 1716.156 | 1782.119 |
| GSE39582_GSM972174 | -419.636 | 1676.258 | 1256.622 |
| GSE39582_GSM972175 | -72.9394 | 1599.353 | 1526.413 |
| GSE39582_GSM972176 | 1961.737 | 3163.172 | 5124.909 |
| GSE39582_GSM972177 | 112.5954 | 2451.697 | 2564.293 |
| GSE39582_GSM972178 | -815.556 | 2175.26 | 1359.704 |
| GSE39582_GSM972179 | 1865.227 | 5131.888 | 6997.115 |
| GSE39582_GSM972180 | 87.98731 | 4361.753 | 4449.741 |
| GSE39582_GSM972181 | 3596.571 | 4018.836 | 7615.406 |
| GSE39582_GSM972182 | -665.569 | 1541.051 | 875.4825 |
| GSE39582_GSM972183 | -1808.66 | 687.8983 | -1120.76 |
| GSE39582_GSM972184 | 2591.732 | 3278.496 | 5870.228 |
| GSE39582_GSM972185 | -348.682 | 752.8799 | 404.1984 |
| GSE39582_GSM972186 | 3090.465 | 4041.943 | 7132.408 |
| GSE39582_GSM972187 | 2416.796 | 3201.004 | 5617.8 |
| GSE39582_GSM972188 | -153.254 | 2036.055 | 1882.802 |
| GSE39582_GSM972189 | 525.1546 | 4469.391 | 4994.545 |
| GSE39582_GSM972190 | -372.717 | 1376.034 | 1003.317 |
| GSE39582_GSM972191 | 34.68532 | 1334.085 | 1368.77 |
| GSE39582_GSM972192 | 837.7287 | 3270.079 | 4107.807 |
| GSE39582_GSM972193 | -674.915 | 1809.715 | 1134.8 |
| GSE39582_GSM972194 | -293.368 | 2491.506 | 2198.138 |
| GSE39582_GSM972195 | -570.036 | 2636.739 | 2066.703 |
| GSE39582_GSM972196 | -789.319 | 2672.394 | 1883.075 |
| GSE39582_GSM972197 | -359.304 | 1777.202 | 1417.898 |
| GSE39582_GSM972198 | -849.324 | 1544.791 | 695.4672 |
| GSE39582_GSM972199 | -340.662 | 1618.763 | 1278.1 |
| GSE39582_GSM972200 | 663.8299 | 2065.83 | 2729.66 |
| GSE39582_GSM972201 | 2547.247 | 3945.346 | 6492.592 |
| GSE39582_GSM972202 | 6.768644 | 2476.882 | 2483.651 |
| GSE39582_GSM972203 | 893.6265 | 3311.541 | 4205.167 |
| GSE39582_GSM972204 | 2246.872 | 3703.927 | 5950.798 |
| GSE39582_GSM972205 | 1097.418 | 4474 | 5571.419 |
| GSE39582_GSM972206 | 1559.962 | 2326.033 | 3885.995 |
| GSE39582_GSM972207 | 950.8701 | 2399.208 | 3350.078 |
| GSE39582_GSM972208 | 3547.633 | 4859.71 | 8407.343 |
| GSE39582_GSM972209 | 932.6816 | 4347.172 | 5279.854 |
| GSE39582_GSM972210 | 1526.129 | 2143.785 | 3669.914 |
| GSE39582_GSM972211 | 707.0554 | 1336.641 | 2043.697 |
| GSE39582_GSM972212 | -575.467 | 2411.581 | 1836.114 |
| GSE39582_GSM972213 | 440.6493 | 936.7932 | 1377.442 |
| GSE39582_GSM972214 | -1206.41 | 1504.201 | 297.7866 |
| GSE39582_GSM972215 | 1285.076 | 3408.61 | 4693.686 |
| GSE39582_GSM972216 | -510.415 | 2250.781 | 1740.365 |
| GSE39582_GSM972217 | 344.4436 | 1709.297 | 2053.74 |
| GSE39582_GSM972218 | -1293.58 | 1678.768 | 385.1873 |
| GSE39582_GSM972219 | 51.51426 | 1202.864 | 1254.378 |
| GSE39582_GSM972220 | 1790.376 | 5133.375 | 6923.752 |
| GSE39582_GSM972221 | -377.866 | 2313.018 | 1935.153 |
| GSE39582_GSM972222 | 227.9135 | 2127.956 | 2355.87 |
| GSE39582_GSM972223 | 1911.263 | 3695.579 | 5606.842 |
| GSE39582_GSM972224 | 2460.195 | 4957.942 | 7418.137 |
| GSE39582_GSM972225 | 752.8422 | 3133.217 | 3886.059 |
| GSE39582_GSM972226 | 362.8213 | 2538.843 | 2901.665 |
| GSE39582_GSM972227 | -675.25 | 2185.142 | 1509.892 |
| GSE39582_GSM972228 | -540.127 | 2716.712 | 2176.586 |
| GSE39582_GSM972229 | 1378.504 | 4016.512 | 5395.016 |
| GSE39582_GSM972230 | -18.8654 | 1306.516 | 1287.651 |
| GSE39582_GSM972231 | 3030.434 | 4170.142 | 7200.575 |
| GSE39582_GSM972232 | 2413.68 | 4027.733 | 6441.412 |
| GSE39582_GSM972233 | 969.9541 | 3611.338 | 4581.293 |
| GSE39582_GSM972234 | 3777.221 | 3541.806 | 7319.027 |
| GSE39582_GSM972235 | 2103.57 | 2841.978 | 4945.547 |
| GSE39582_GSM972236 | 489.584 | 2012.526 | 2502.11 |
| GSE39582_GSM972237 | 0.139861 | 1815.055 | 1815.195 |
| GSE39582_GSM972238 | 1760.883 | 2757.619 | 4518.503 |
| GSE39582_GSM972239 | 1922.666 | 4291.119 | 6213.785 |
| GSE39582_GSM972240 | 2257.856 | 4262.378 | 6520.233 |
| GSE39582_GSM972241 | 7.87666 | 2847.786 | 2855.663 |
| GSE39582_GSM972242 | 2300.826 | 3486.444 | 5787.27 |
| GSE39582_GSM972243 | 1037.704 | 3566.789 | 4604.493 |
| GSE39582_GSM972244 | 1157.413 | 3991.154 | 5148.566 |
| GSE39582_GSM972245 | 454.4621 | 2393.32 | 2847.782 |
| GSE39582_GSM972246 | 349.1516 | 1185.037 | 1534.189 |
| GSE39582_GSM972247 | -352.082 | 2278.741 | 1926.659 |
| GSE39582_GSM972248 | 120.3412 | 2318.536 | 2438.877 |
| GSE39582_GSM972249 | 986.5865 | 2735.894 | 3722.481 |
| GSE39582_GSM972250 | 765.1545 | 1704.628 | 2469.782 |
| GSE39582_GSM972251 | 2280.663 | 2223.029 | 4503.692 |
| GSE39582_GSM972252 | 1879.505 | 3200.407 | 5079.913 |
| GSE39582_GSM972253 | -30.8509 | 1978.501 | 1947.65 |
| GSE39582_GSM972254 | 1942.833 | 2816.794 | 4759.626 |
| GSE39582_GSM972255 | 2161.466 | 1884.539 | 4046.005 |
| GSE39582_GSM972256 | 327.3785 | 2094.418 | 2421.797 |
| GSE39582_GSM972257 | -122.073 | 2617.784 | 2495.711 |
| GSE39582_GSM972258 | 423.5105 | 1835.563 | 2259.073 |
| GSE39582_GSM972259 | 743.6596 | 3180.786 | 3924.446 |
| GSE39582_GSM972260 | -94.8782 | 1989.371 | 1894.493 |
| GSE39582_GSM972261 | 585.8566 | 3405.917 | 3991.774 |
| GSE39582_GSM972262 | 4634.674 | 5141.509 | 9776.184 |
| GSE39582_GSM972263 | 1173.36 | 3850.653 | 5024.013 |
| GSE39582_GSM972264 | -160.35 | 2948.19 | 2787.839 |
| GSE39582_GSM972265 | 546.4941 | 2277.737 | 2824.231 |
| GSE39582_GSM972266 | -1434.44 | 463.4061 | -971.032 |
| GSE39582_GSM972267 | 547.6803 | 2081.048 | 2628.728 |
| GSE39582_GSM972268 | -411.453 | 1753.374 | 1341.92 |
| GSE39582_GSM972269 | -290.604 | 3490.503 | 3199.898 |
| GSE39582_GSM972270 | 505.9678 | 3430.705 | 3936.673 |
| GSE39582_GSM972271 | -605.046 | 1334.566 | 729.5201 |
| GSE39582_GSM972272 | -793.832 | 1653.1 | 859.2684 |
| GSE39582_GSM972273 | -701.347 | 2483.615 | 1782.268 |
| GSE39582_GSM972274 | 1451.729 | 3892.619 | 5344.348 |
| GSE39582_GSM972275 | 2828.35 | 5702.986 | 8531.336 |
| GSE39582_GSM972276 | -244.694 | 2801.23 | 2556.536 |
| GSE39582_GSM972277 | -685.412 | 1493.035 | 807.6229 |
| GSE39582_GSM972278 | 290.4836 | 3385.018 | 3675.501 |
| GSE39582_GSM972279 | 2577.125 | 3402.763 | 5979.888 |
| GSE39582_GSM972280 | 1370.313 | 3746.143 | 5116.456 |
| GSE39582_GSM972281 | 838.9541 | 3366.193 | 4205.147 |
| GSE39582_GSM972282 | -71.3162 | 2271.474 | 2200.158 |
| GSE39582_GSM972283 | 906.0467 | 3464.483 | 4370.529 |
| GSE39582_GSM972284 | -1431.94 | 1408.717 | -23.2226 |
| GSE39582_GSM972285 | 133.941 | 1750.197 | 1884.138 |
| GSE39582_GSM972286 | 1020.077 | 3062.388 | 4082.464 |
| GSE39582_GSM972287 | 2299.241 | 4470.311 | 6769.552 |
| GSE39582_GSM972288 | -659.247 | 1292.54 | 633.2929 |
| GSE39582_GSM972289 | -43.7982 | 1746.555 | 1702.757 |
| GSE39582_GSM972290 | 2411.96 | 4538.856 | 6950.816 |
| GSE39582_GSM972291 | 1509.066 | 2581.808 | 4090.874 |
| GSE39582_GSM972292 | 192.0584 | 2959.631 | 3151.69 |
| GSE39582_GSM972293 | 1716.768 | 3493.521 | 5210.289 |
| GSE39582_GSM972294 | 1684.632 | 5566.786 | 7251.419 |
| GSE39582_GSM972295 | -991.994 | 1005.097 | 13.10235 |
| GSE39582_GSM972296 | -763.61 | 3019.852 | 2256.242 |
| GSE39582_GSM972297 | 2466.363 | 4303.691 | 6770.054 |
| GSE39582_GSM972298 | 167.1322 | 2446.923 | 2614.055 |
| GSE39582_GSM972299 | 1645.195 | 2657.873 | 4303.068 |
| GSE39582_GSM972300 | 60.37315 | 2550.335 | 2610.708 |
| GSE39582_GSM972301 | 2591.446 | 4915.228 | 7506.674 |
| GSE39582_GSM972302 | -257.075 | 1666.186 | 1409.111 |
| GSE39582_GSM972303 | -752.888 | 1384.412 | 631.5237 |
| GSE39582_GSM972304 | -1512.26 | 2026.479 | 514.2165 |
| GSE39582_GSM972305 | 1747.024 | 5618.597 | 7365.621 |
| GSE39582_GSM972306 | -613.931 | 1272.949 | 659.0176 |
| GSE39582_GSM972307 | -909.091 | 2468.802 | 1559.711 |
| GSE39582_GSM972308 | 781.549 | 4201.322 | 4982.871 |
| GSE39582_GSM972309 | -1788.51 | 710.6336 | -1077.88 |
| GSE39582_GSM972310 | 768.1668 | 2263.533 | 3031.699 |
| GSE39582_GSM972311 | -124.377 | 668.0215 | 543.6444 |
| GSE39582_GSM972312 | -1196.95 | 1080.052 | -116.901 |
| GSE39582_GSM972313 | -1042.89 | 297.1628 | -745.73 |
| GSE39582_GSM972314 | 282.7318 | 4082.13 | 4364.862 |
| GSE39582_GSM972315 | -591.751 | 2610.351 | 2018.599 |
| GSE39582_GSM972316 | 253.9255 | 2539.305 | 2793.231 |
| GSE39582_GSM972317 | 895.9006 | 2801.434 | 3697.334 |
| GSE39582_GSM972318 | 586.7607 | 2196.578 | 2783.338 |
| GSE39582_GSM972319 | -543.625 | 1507.433 | 963.8082 |
| GSE39582_GSM972320 | -214.878 | 1495.58 | 1280.702 |
| GSE39582_GSM972321 | 712.4348 | 2542.762 | 3255.197 |
| GSE39582_GSM972322 | 1060.446 | 4395.449 | 5455.895 |
| GSE39582_GSM972323 | -395.067 | 1259.227 | 864.1602 |
| GSE39582_GSM972324 | -18.4304 | 1950.445 | 1932.015 |
| GSE39582_GSM972325 | 704.5785 | 3484.514 | 4189.093 |
| GSE39582_GSM972326 | -1081.47 | 2138.616 | 1057.148 |
| GSE39582_GSM972327 | 2807.731 | 4990.452 | 7798.183 |
| GSE39582_GSM972328 | 805.8779 | 1155.128 | 1961.006 |
| GSE39582_GSM972329 | 1830.108 | 3548.246 | 5378.354 |
| GSE39582_GSM972330 | 1810.942 | 3832.8 | 5643.742 |
| GSE39582_GSM972331 | 660.6449 | 2525.499 | 3186.144 |
| GSE39582_GSM972332 | 967.1659 | 1840.911 | 2808.077 |
| GSE39582_GSM972333 | 244.3299 | 1952.274 | 2196.604 |
| GSE39582_GSM972334 | 3211.947 | 5484.023 | 8695.969 |
| GSE39582_GSM972335 | 238.9084 | 2394.399 | 2633.307 |
| GSE39582_GSM972336 | 540.7152 | 2003.206 | 2543.921 |
| GSE39582_GSM972337 | 1845.331 | 6314.156 | 8159.488 |
| GSE39582_GSM972338 | -93.0828 | 2912.883 | 2819.8 |
| GSE39582_GSM972339 | 722.9767 | 2168.219 | 2891.196 |
| GSE39582_GSM972340 | 25.68733 | 2583.676 | 2609.363 |
| GSE39582_GSM972341 | 719.2167 | 3144.372 | 3863.589 |
| GSE39582_GSM972342 | 1040.408 | 2324.076 | 3364.484 |
| GSE39582_GSM972343 | 280.0743 | 2420.747 | 2700.821 |
| GSE39582_GSM972344 | -280.464 | 1380.898 | 1100.433 |
| GSE39582_GSM972345 | 2651.446 | 3030.659 | 5682.104 |
| GSE39582_GSM972346 | -612.427 | 966.9335 | 354.5066 |
| GSE39582_GSM972347 | 337.5691 | 2600.494 | 2938.063 |
| GSE39582_GSM972348 | -308.683 | 2228.258 | 1919.575 |
| GSE39582_GSM972349 | 4525.351 | 4320.432 | 8845.783 |
| GSE39582_GSM972350 | -1812.39 | 1225.633 | -586.76 |
| GSE39582_GSM972351 | 78.55379 | 1469.642 | 1548.196 |
| GSE39582_GSM972352 | 3232.102 | 2694.558 | 5926.66 |
| GSE39582_GSM972353 | 1084.86 | 3400.886 | 4485.747 |
| GSE39582_GSM972354 | -44.0645 | 2507.879 | 2463.815 |
| GSE39582_GSM972355 | 1904.042 | 2326.819 | 4230.861 |
| GSE39582_GSM972356 | -171.683 | 2821.562 | 2649.879 |
| GSE39582_GSM972357 | -317.206 | 2769.178 | 2451.972 |
| GSE39582_GSM972358 | 1078.915 | 3181.76 | 4260.675 |
| GSE39582_GSM972359 | 3042.988 | 4384.907 | 7427.894 |
| GSE39582_GSM972360 | 1528.44 | 2799.077 | 4327.517 |
| GSE39582_GSM972361 | 3844.38 | 6490.141 | 10334.52 |
| GSE39582_GSM972362 | 76.26538 | 2082.093 | 2158.358 |
| GSE39582_GSM972363 | 831.4696 | 3095.6 | 3927.07 |
| GSE39582_GSM972364 | -251.284 | 899.1629 | 647.8789 |
| GSE39582_GSM972365 | 2385.4 | 4249.778 | 6635.177 |
| GSE39582_GSM972366 | 1827.245 | 3582.749 | 5409.994 |
| GSE39582_GSM972367 | 3191.754 | 3779.098 | 6970.852 |
| GSE39582_GSM972368 | -1335.58 | 2039.545 | 703.9696 |
| GSE39582_GSM972369 | 3660.864 | 4865.93 | 8526.793 |
| GSE39582_GSM972370 | 473.8323 | 2820.764 | 3294.597 |
| GSE39582_GSM972371 | 204.6515 | 1906.866 | 2111.517 |
| GSE39582_GSM972372 | 1174.916 | 2951.385 | 4126.302 |
| GSE39582_GSM972373 | -507.677 | 359.1842 | -148.493 |
| GSE39582_GSM972374 | 572.1649 | 3242.432 | 3814.597 |
| GSE39582_GSM972375 | 1690.079 | 2906.378 | 4596.457 |
| GSE39582_GSM972376 | 1316.498 | 1673.617 | 2990.115 |
| GSE39582_GSM972377 | 2624.723 | 4227.511 | 6852.234 |
| GSE39582_GSM972378 | 867.0853 | 2375.169 | 3242.254 |
| GSE39582_GSM972379 | -1111.91 | 2862.959 | 1751.046 |
| GSE39582_GSM972380 | 2236.243 | 3932.08 | 6168.322 |
| GSE39582_GSM972381 | 1191.724 | 1751.968 | 2943.691 |
| GSE39582_GSM972382 | 122.8704 | 1912.034 | 2034.905 |
| GSE39582_GSM972383 | -359.76 | 2364.011 | 2004.252 |
| GSE39582_GSM972384 | 690.1316 | 1547.665 | 2237.797 |
| GSE39582_GSM972385 | 2713.015 | 4951.182 | 7664.197 |
| GSE39582_GSM972386 | -243.726 | 1796.332 | 1552.606 |
| GSE39582_GSM972387 | -789.19 | 1657.974 | 868.7844 |
| GSE39582_GSM972388 | -702.193 | 307.9965 | -394.196 |
| GSE39582_GSM972389 | -190.811 | 2896.719 | 2705.908 |
| GSE39582_GSM972390 | -54.2267 | 2186.65 | 2132.423 |
| GSE39582_GSM972391 | 948.3826 | 3115.302 | 4063.685 |
| GSE39582_GSM972392 | 196.7995 | 2359.156 | 2555.956 |
| GSE39582_GSM972393 | 648.4065 | 2356.305 | 3004.712 |
| GSE39582_GSM972394 | 1776.574 | 2819.75 | 4596.324 |
| GSE39582_GSM972395 | 1008.16 | 2935.323 | 3943.483 |
| GSE39582_GSM972396 | -1392.49 | 257.0774 | -1135.41 |
| GSE39582_GSM972397 | -592.942 | 2650.823 | 2057.881 |
| GSE39582_GSM972398 | -15.6981 | 3358.194 | 3342.496 |
| GSE39582_GSM972399 | 443.2695 | 1719.983 | 2163.252 |
| GSE39582_GSM972400 | 790.5764 | 2859.888 | 3650.465 |
| GSE39582_GSM972401 | 3533.968 | 6173.34 | 9707.308 |
| GSE39582_GSM972402 | 1878 | 2329.635 | 4207.635 |
| GSE39582_GSM972403 | 614.1256 | 2935.651 | 3549.777 |
| GSE39582_GSM972404 | 1015.262 | 2760.93 | 3776.192 |
| GSE39582_GSM972405 | 1242.706 | 2913.877 | 4156.583 |
| GSE39582_GSM972406 | 1883.953 | 4206.39 | 6090.343 |
| GSE39582_GSM972407 | 1358.43 | 2145.231 | 3503.661 |
| GSE39582_GSM972408 | 824.4476 | 3544.273 | 4368.72 |
| GSE39582_GSM972409 | 4547.302 | 6293.647 | 10840.95 |
| GSE39582_GSM972410 | 2709.329 | 3475.132 | 6184.461 |
| GSE39582_GSM972411 | -488.994 | 1797.776 | 1308.782 |
| GSE39582_GSM972412 | 670.4645 | 2512.467 | 3182.932 |
| GSE39582_GSM972413 | 4353.016 | 4192.066 | 8545.082 |
| GSE39582_GSM972414 | 308.2464 | 2105.62 | 2413.866 |
| GSE39582_GSM972415 | -1116.31 | 1081.62 | -34.6895 |
| GSE39582_GSM972416 | 1364.122 | 1519.204 | 2883.327 |
| GSE39582_GSM972417 | 911.7267 | 2160.566 | 3072.293 |
| GSE39582_GSM972418 | 500.6688 | 1702.893 | 2203.562 |
| GSE39582_GSM972419 | 1431.133 | 1876.246 | 3307.379 |
| GSE39582_GSM972420 | 1488.768 | 4466.474 | 5955.241 |
| GSE39582_GSM972421 | 1851.399 | 2440.602 | 4292.001 |
| GSE39582_GSM972422 | 3441.486 | 4828.151 | 8269.637 |
| GSE39582_GSM972423 | 917.6684 | 2886.699 | 3804.367 |
| GSE39582_GSM972424 | -367.868 | 1038.29 | 670.422 |
| GSE39582_GSM972425 | 1816.586 | 2447.934 | 4264.52 |
| GSE39582_GSM972426 | 1514.549 | 3902.131 | 5416.68 |
| GSE39582_GSM972427 | 715.2894 | 1809.96 | 2525.25 |
| GSE39582_GSM972428 | 1837.492 | 3065.535 | 4903.027 |
| GSE39582_GSM972429 | -43.4841 | 3088.314 | 3044.83 |
| GSE39582_GSM972430 | 1255.586 | 4737.323 | 5992.909 |
| GSE39582_GSM972431 | 2001.184 | 4094.937 | 6096.121 |
| GSE39582_GSM972432 | 1150.383 | 1912.754 | 3063.136 |
| GSE39582_GSM972433 | 3832.676 | 4152.951 | 7985.627 |
| GSE39582_GSM972434 | -745.501 | 2154.922 | 1409.421 |
| GSE39582_GSM972435 | 610.0284 | 1690.405 | 2300.433 |
| GSE39582_GSM972436 | 629.7348 | 2339.405 | 2969.139 |
| GSE39582_GSM972437 | 2981.231 | 3660.504 | 6641.735 |
| GSE39582_GSM972438 | 4046.808 | 4206.239 | 8253.047 |
| GSE39582_GSM972439 | 1080.722 | 1556.529 | 2637.251 |
| GSE39582_GSM972440 | 2576.168 | 4002.475 | 6578.643 |
| GSE39582_GSM972441 | 2684.65 | 4795.485 | 7480.135 |
| GSE39582_GSM972442 | 865.0977 | 4951.821 | 5816.919 |
| GSE39582_GSM972443 | -487.192 | 2604.844 | 2117.652 |
| GSE39582_GSM972444 | 2861.783 | 4417.763 | 7279.546 |
| GSE39582_GSM972445 | 846.3167 | 3505.518 | 4351.835 |
| GSE39582_GSM972446 | 944.2418 | 2235.396 | 3179.638 |
| GSE39582_GSM972447 | 1141.83 | 3780.532 | 4922.362 |
| GSE39582_GSM972448 | 310.3432 | 725.8131 | 1036.156 |
| GSE39582_GSM972449 | 1354.078 | 2862.672 | 4216.75 |
| GSE39582_GSM972450 | -207.04 | 2352.368 | 2145.328 |
| GSE39582_GSM972451 | -770.054 | 1463.945 | 693.8917 |
| GSE39582_GSM972452 | 2716.65 | 6018.781 | 8735.431 |
| GSE39582_GSM972453 | -339.137 | 2340.184 | 2001.048 |
| GSE39582_GSM972454 | 563.5482 | 3601.197 | 4164.745 |
| GSE39582_GSM972455 | 3507.587 | 5155.817 | 8663.404 |
| GSE39582_GSM972456 | 1184.159 | 1981.733 | 3165.893 |
| GSE39582_GSM972457 | -526.717 | 2434.412 | 1907.695 |
| GSE39582_GSM972458 | 1795.959 | 5472.982 | 7268.941 |
| GSE39582_GSM972459 | -1.85853 | 1798.328 | 1796.469 |
| GSE39582_GSM972460 | 1129.501 | 2339.572 | 3469.073 |
| GSE39582_GSM972461 | 83.06645 | 1994.258 | 2077.325 |
| GSE39582_GSM972462 | -13.3735 | 2035.291 | 2021.918 |
| GSE39582_GSM972463 | -503.767 | 2237.083 | 1733.315 |
| GSE39582_GSM972464 | 1855.706 | 3683.111 | 5538.816 |
| GSE39582_GSM972465 | 210.9678 | 1197.802 | 1408.769 |
| GSE39582_GSM972466 | -589.451 | 2040.18 | 1450.73 |
| GSE39582_GSM972467 | 3876.506 | 3298.697 | 7175.203 |
| GSE39582_GSM972468 | 714.1966 | 2548.481 | 3262.677 |
| GSE39582_GSM972469 | 813.7404 | 2495.306 | 3309.046 |
| GSE39582_GSM972470 | 4125.168 | 6783.726 | 10908.89 |
| GSE39582_GSM972471 | 701.1678 | 3578.755 | 4279.923 |
| GSE39582_GSM972472 | 702.5182 | 2044.278 | 2746.796 |
| GSE39582_GSM972473 | 645.8533 | 3562.867 | 4208.72 |
| GSE39582_GSM972474 | 1451.69 | 2831.254 | 4282.944 |
| GSE39582_GSM972475 | -635.534 | 2488.633 | 1853.099 |
| GSE39582_GSM972476 | 3962.115 | 3557.793 | 7519.908 |
| GSE39582_GSM972477 | -1260.58 | 1780.338 | 519.7539 |
| GSE39582_GSM972478 | 344.9885 | 2410.334 | 2755.323 |
| GSE39582_GSM972479 | 5211.478 | 4284.759 | 9496.237 |
| GSE39582_GSM972480 | -957.877 | 2018.512 | 1060.635 |
| GSE39582_GSM972481 | 2342.638 | 2548.12 | 4890.759 |
| GSE39582_GSM972482 | 1897.653 | 1879.071 | 3776.724 |
| GSE39582_GSM972483 | 915.1068 | 1199.345 | 2114.452 |
| GSE39582_GSM972484 | 1706.038 | 2457.781 | 4163.82 |
| GSE39582_GSM972485 | 1190.727 | 2871.173 | 4061.899 |
| GSE39582_GSM972486 | 1140.778 | 1786.48 | 2927.258 |
| GSE39582_GSM972487 | -251.114 | 2443.761 | 2192.647 |
| GSE39582_GSM972488 | -54.7006 | 3232.374 | 3177.673 |
| GSE39582_GSM972489 | 600.7321 | 2631.015 | 3231.747 |
| GSE39582_GSM972490 | 129.1655 | 1549.79 | 1678.955 |
| GSE39582_GSM972491 | 789.9269 | 1608.747 | 2398.674 |
| GSE39582_GSM972492 | 384.585 | 2578.761 | 2963.346 |
| GSE39582_GSM972493 | 1523.082 | 2447.9 | 3970.982 |
| GSE39582_GSM972494 | 1146.088 | 4007.892 | 5153.98 |
| GSE39582_GSM972495 | -108.82 | 2092.081 | 1983.261 |
| GSE39582_GSM972496 | -832.481 | 4098.365 | 3265.885 |
| GSE39582_GSM972497 | 466.7327 | 1295.181 | 1761.914 |
| GSE39582_GSM972498 | -314.803 | 1161.517 | 846.7142 |
| GSE39582_GSM972499 | -208.21 | 2932.087 | 2723.877 |
| GSE39582_GSM972500 | 2559.901 | 3703.371 | 6263.272 |
| GSE39582_GSM972501 | 754.6279 | 3881.626 | 4636.254 |
| GSE39582_GSM972502 | -1323.68 | 1165.815 | -157.861 |
| GSE39582_GSM972503 | 2285.757 | 4435.262 | 6721.02 |
| GSE39582_GSM972504 | 1687.301 | 2914.113 | 4601.415 |
| GSE39582_GSM972505 | 373.2217 | 2895.582 | 3268.804 |
| GSE39582_GSM972506 | 1501.659 | 3565.893 | 5067.552 |
| GSE39582_GSM972507 | 594.374 | 3230.385 | 3824.759 |
| GSE39582_GSM972508 | 318.9727 | 3171.722 | 3490.695 |
| GSE39582_GSM972509 | -116.625 | 1922.192 | 1805.568 |
| GSE39582_GSM972510 | -1089.17 | 1789.627 | 700.4534 |
| GSE39582_GSM972511 | 681.2298 | 2035.265 | 2716.495 |
| GSE39582_GSM972512 | -374.509 | 2453.26 | 2078.751 |
| GSE39582_GSM972513 | 264.0617 | 1199.564 | 1463.626 |
| GSE39582_GSM972514 | 453.0652 | 2478.649 | 2931.714 |
| GSE39582_GSM972515 | -557.582 | 1394.802 | 837.2204 |
| GSE39582_GSM972516 | -1019.58 | 1288.792 | 269.2071 |
| GSE39582_GSM972517 | 1050.649 | 2234.922 | 3285.571 |
| GSE39582_GSM972518 | 1442.211 | 3229.376 | 4671.587 |
| GSE39582_GSM972519 | 1316.89 | 2600.375 | 3917.266 |
| GSE39582_GSM972520 | 884.1006 | 3418.508 | 4302.608 |
| GSE39582_GSM972521 | 2294.433 | 3515.512 | 5809.945 |
| GSE39582_GSM972522 | 2364.47 | 5306.246 | 7670.716 |

**Supplementary Table S9.** The IPS of TCGA samples.

| barcode | ips_ctla4_neg_pd1_neg | ips_ctla4_neg_pd1_pos | ips_ctla4_pos_pd1_neg | ips_ctla4_pos_pd1_pos |
| --- | --- | --- | --- | --- |
| TCGA-QG-A5YX | 10 | 8 | 10 | 8 |
| TCGA-G4-6306 | 10 | 9 | 10 | 9 |
| TCGA-AA-A02E | 10 | 8 | 9 | 8 |
| TCGA-AA-A00L | 10 | 8 | 10 | 8 |
| TCGA-G4-6626 | 10 | 8 | 9 | 8 |
| TCGA-AZ-4308 | 10 | 8 | 9 | 8 |
| TCGA-DM-A28K | 10 | 8 | 10 | 8 |
| TCGA-AA-3531 | 10 | 9 | 10 | 8 |
| TCGA-G4-6294 | 10 | 8 | 9 | 7 |
| TCGA-AD-6963 | 10 | 9 | 10 | 9 |
| TCGA-A6-5661 | 10 | 8 | 9 | 8 |
| TCGA-AA-3530 | 10 | 8 | 9 | 8 |
| TCGA-AA-3851 | 10 | 8 | 9 | 8 |
| TCGA-A6-2679 | 10 | 10 | 10 | 10 |
| TCGA-AA-3837 | 10 | 8 | 9 | 7 |
| TCGA-AA-3971 | 10 | 8 | 9 | 8 |
| TCGA-AZ-4313 | 10 | 9 | 10 | 8 |
| TCGA-AA-3552 | 10 | 9 | 10 | 8 |
| TCGA-AA-3527 | 10 | 10 | 10 | 9 |
| TCGA-D5-6930 | 10 | 9 | 10 | 9 |
| TCGA-AA-A03F | 10 | 8 | 10 | 8 |
| TCGA-AA-3688 | 10 | 8 | 9 | 8 |
| TCGA-DM-A28F | 10 | 8 | 9 | 7 |
| TCGA-AY-4071 | 10 | 9 | 10 | 9 |
| TCGA-AA-3862 | 10 | 9 | 10 | 9 |
| TCGA-NH-A6GC | 10 | 8 | 9 | 7 |
| TCGA-AA-3519 | 10 | 8 | 9 | 8 |
| TCGA-AA-3502 | 10 | 8 | 9 | 7 |
| TCGA-AY-5543 | 10 | 8 | 9 | 8 |
| TCGA-DM-A1D4 | 10 | 8 | 9 | 7 |
| TCGA-QG-A5Z2 | 10 | 9 | 10 | 9 |
| TCGA-AA-A01S | 10 | 8 | 9 | 7 |
| TCGA-DM-A28E | 10 | 8 | 9 | 7 |
| TCGA-4N-A93T | 10 | 8 | 9 | 7 |
| TCGA-DM-A1D8 | 10 | 9 | 10 | 8 |
| TCGA-AA-A01I | 10 | 8 | 10 | 8 |
| TCGA-A6-5656 | 10 | 8 | 9 | 7 |
| TCGA-AA-3848 | 10 | 8 | 9 | 8 |
| TCGA-AA-A029 | 10 | 8 | 9 | 8 |
| TCGA-AA-3856 | 10 | 9 | 9 | 8 |
| TCGA-G4-6323 | 10 | 8 | 9 | 8 |
| TCGA-CM-5861 | 10 | 9 | 10 | 8 |
| TCGA-AA-A01R | 10 | 10 | 10 | 10 |
| TCGA-D5-6532 | 10 | 8 | 9 | 7 |
| TCGA-AA-3518 | 10 | 9 | 9 | 8 |
| TCGA-CK-5912 | 10 | 8 | 9 | 7 |
| TCGA-AA-3516 | 10 | 10 | 10 | 10 |
| TCGA-AA-3846 | 10 | 9 | 10 | 9 |
| TCGA-G4-6320 | 10 | 8 | 9 | 7 |
| TCGA-DM-A1DA | 10 | 8 | 9 | 7 |
| TCGA-AA-3672 | 10 | 10 | 10 | 10 |
| TCGA-DM-A28M | 10 | 8 | 9 | 7 |
| TCGA-AY-6197 | 10 | 8 | 9 | 8 |
| TCGA-AA-A01T | 10 | 9 | 10 | 9 |
| TCGA-AA-A00W | 10 | 8 | 9 | 7 |
| TCGA-AA-A01G | 10 | 8 | 9 | 7 |
| TCGA-AZ-4681 | 10 | 8 | 9 | 7 |
| TCGA-A6-2683 | 10 | 8 | 9 | 8 |
| TCGA-AZ-6599 | 10 | 8 | 9 | 7 |
| TCGA-CM-6675 | 10 | 8 | 9 | 7 |
| TCGA-CK-6747 | 10 | 8 | 9 | 8 |
| TCGA-DM-A0X9 | 10 | 8 | 9 | 8 |
| TCGA-AA-A022 | 10 | 10 | 10 | 10 |
| TCGA-CK-6751 | 10 | 8 | 9 | 8 |
| TCGA-CM-4752 | 10 | 9 | 10 | 9 |
| TCGA-AD-6895 | 10 | 9 | 9 | 9 |
| TCGA-AA-3844 | 10 | 8 | 9 | 8 |
| TCGA-T9-A92H | 10 | 9 | 10 | 8 |
| TCGA-A6-3807 | 10 | 9 | 9 | 8 |
| TCGA-CA-5255 | 10 | 8 | 9 | 8 |
| TCGA-DM-A1HA | 10 | 9 | 10 | 8 |
| TCGA-CK-5914 | 10 | 9 | 10 | 8 |
| TCGA-AA-3861 | 10 | 8 | 9 | 8 |
| TCGA-RU-A8FL | 10 | 8 | 9 | 7 |
| TCGA-D5-6929 | 10 | 8 | 9 | 8 |
| TCGA-F4-6856 | 10 | 8 | 9 | 8 |
| TCGA-CA-5796 | 10 | 8 | 9 | 8 |
| TCGA-A6-6653 | 10 | 9 | 10 | 8 |
| TCGA-G4-6321 | 10 | 9 | 10 | 9 |
| TCGA-AA-3855 | 10 | 8 | 9 | 8 |
| TCGA-DM-A0XD | 10 | 8 | 9 | 7 |
| TCGA-DM-A1D6 | 10 | 8 | 9 | 7 |
| TCGA-AA-3679 | 10 | 8 | 9 | 7 |
| TCGA-AA-A004 | 10 | 9 | 10 | 9 |
| TCGA-D5-5537 | 10 | 8 | 9 | 7 |
| TCGA-CM-4750 | 10 | 9 | 10 | 8 |
| TCGA-F4-6806 | 10 | 9 | 10 | 8 |
| TCGA-AZ-6606 | 10 | 8 | 9 | 8 |
| TCGA-CM-4743 | 10 | 10 | 10 | 10 |
| TCGA-NH-A6GB | 10 | 8 | 9 | 8 |
| TCGA-QL-A97D | 10 | 9 | 10 | 9 |
| TCGA-A6-4107 | 10 | 8 | 9 | 7 |
| TCGA-AA-A01Z | 10 | 8 | 9 | 7 |
| TCGA-D5-6535 | 10 | 9 | 10 | 9 |
| TCGA-CM-4751 | 10 | 9 | 9 | 9 |
| TCGA-AZ-4682 | 10 | 8 | 9 | 7 |
| TCGA-AA-3525 | 10 | 9 | 10 | 8 |
| TCGA-AA-3684 | 10 | 9 | 9 | 8 |
| TCGA-AA-3522 | 10 | 8 | 9 | 8 |
| TCGA-CM-4744 | 10 | 10 | 10 | 9 |
| TCGA-AA-A03J | 10 | 9 | 10 | 9 |
| TCGA-AZ-4616 | 10 | 9 | 9 | 9 |
| TCGA-G4-6322 | 10 | 8 | 9 | 8 |
| TCGA-CM-4748 | 10 | 8 | 9 | 7 |
| TCGA-AA-A02Y | 10 | 9 | 9 | 8 |
| TCGA-A6-2680 | 10 | 8 | 9 | 8 |
| TCGA-AG-A023 | 10 | 8 | 10 | 8 |
| TCGA-EI-6883 | 10 | 8 | 9 | 8 |
| TCGA-AG-A020 | 10 | 8 | 9 | 7 |
| TCGA-DC-5337 | 10 | 8 | 9 | 8 |
| TCGA-DC-6682 | 10 | 8 | 9 | 8 |
| TCGA-AG-3599 | 10 | 9 | 10 | 8 |
| TCGA-EI-6510 | 10 | 8 | 9 | 8 |
| TCGA-AG-A01L | 10 | 8 | 9 | 7 |
| TCGA-AG-3728 | 10 | 9 | 10 | 9 |
| TCGA-AG-3882 | 10 | 9 | 10 | 9 |
| TCGA-AG-A02G | 10 | 8 | 9 | 8 |
| TCGA-AG-A032 | 10 | 8 | 10 | 8 |
| TCGA-AG-3602 | 10 | 9 | 10 | 8 |
| TCGA-AG-A036 | 10 | 8 | 10 | 8 |
| TCGA-G5-6235 | 10 | 8 | 9 | 8 |
| TCGA-AG-3608 | 10 | 9 | 10 | 8 |
| TCGA-AG-3598 | 10 | 9 | 9 | 8 |
| TCGA-AG-3611 | 10 | 9 | 10 | 8 |
| TCGA-AG-3909 | 10 | 8 | 9 | 7 |
| TCGA-DY-A1DG | 10 | 9 | 10 | 8 |
| TCGA-AG-3887 | 10 | 8 | 9 | 8 |
| TCGA-AG-4007 | 10 | 8 | 9 | 8 |
| TCGA-AF-2691 | 10 | 8 | 9 | 8 |
| TCGA-AH-6544 | 10 | 9 | 10 | 8 |
| TCGA-AG-3890 | 10 | 8 | 10 | 8 |
| TCGA-CI-6622 | 10 | 8 | 9 | 7 |
| TCGA-AG-A01J | 10 | 8 | 9 | 7 |
| TCGA-DC-4749 | 10 | 8 | 9 | 7 |
| TCGA-AG-3591 | 10 | 8 | 9 | 8 |
| TCGA-CL-4957 | 10 | 8 | 9 | 7 |
| TCGA-F5-6812 | 10 | 8 | 9 | 8 |
| TCGA-AG-3885 | 10 | 9 | 9 | 8 |
| TCGA-AG-A02X | 10 | 8 | 9 | 7 |
| TCGA-AG-A00C | 10 | 9 | 10 | 8 |
| TCGA-A6-6137 | 9 | 8 | 9 | 8 |
| TCGA-AU-6004 | 9 | 8 | 9 | 8 |
| TCGA-AZ-4614 | 9 | 7 | 9 | 7 |
| TCGA-AA-3693 | 9 | 7 | 8 | 6 |
| TCGA-AA-3510 | 9 | 8 | 9 | 8 |
| TCGA-AY-6386 | 9 | 8 | 9 | 7 |
| TCGA-5M-AAT5 | 9 | 7 | 9 | 7 |
| TCGA-CK-5913 | 9 | 8 | 9 | 8 |
| TCGA-AA-3980 | 9 | 8 | 9 | 8 |
| TCGA-A6-6649 | 9 | 7 | 8 | 7 |
| TCGA-AA-3869 | 9 | 8 | 9 | 8 |
| TCGA-CA-5797 | 9 | 7 | 8 | 7 |
| TCGA-A6-6782 | 9 | 7 | 8 | 7 |
| TCGA-AA-3675 | 9 | 7 | 8 | 7 |
| TCGA-AA-3858 | 9 | 8 | 9 | 7 |
| TCGA-AA-3812 | 9 | 8 | 9 | 7 |
| TCGA-A6-A5ZU | 9 | 8 | 8 | 7 |
| TCGA-NH-A50T | 9 | 7 | 9 | 7 |
| TCGA-F4-6808 | 9 | 7 | 9 | 7 |
| TCGA-CM-5868 | 9 | 7 | 9 | 7 |
| TCGA-A6-6138 | 9 | 9 | 9 | 9 |
| TCGA-AA-A00A | 9 | 8 | 9 | 8 |
| TCGA-NH-A8F8 | 9 | 7 | 8 | 7 |
| TCGA-AA-A02K | 9 | 7 | 8 | 6 |
| TCGA-AA-3872 | 9 | 7 | 8 | 7 |
| TCGA-AA-3562 | 9 | 7 | 9 | 7 |
| TCGA-AA-A01F | 9 | 8 | 9 | 7 |
| TCGA-AA-3850 | 9 | 8 | 9 | 8 |
| TCGA-A6-6140 | 9 | 8 | 9 | 7 |
| TCGA-G4-6299 | 9 | 8 | 9 | 8 |
| TCGA-AA-3821 | 9 | 8 | 9 | 8 |
| TCGA-AA-A00E | 9 | 8 | 9 | 8 |
| TCGA-A6-5666 | 9 | 7 | 8 | 6 |
| TCGA-CA-5254 | 9 | 8 | 9 | 7 |
| TCGA-G4-6293 | 9 | 8 | 9 | 8 |
| TCGA-CM-6172 | 9 | 8 | 9 | 7 |
| TCGA-AA-3506 | 9 | 7 | 8 | 7 |
| TCGA-F4-6461 | 9 | 8 | 9 | 8 |
| TCGA-F4-6460 | 9 | 7 | 8 | 7 |
| TCGA-AA-3712 | 9 | 8 | 9 | 7 |
| TCGA-G4-6628 | 9 | 9 | 9 | 9 |
| TCGA-AA-3560 | 9 | 8 | 9 | 7 |
| TCGA-CM-5341 | 9 | 7 | 8 | 7 |
| TCGA-AA-3548 | 9 | 8 | 9 | 7 |
| TCGA-AA-3561 | 9 | 7 | 9 | 7 |
| TCGA-AA-3655 | 9 | 7 | 8 | 7 |
| TCGA-AA-3866 | 9 | 8 | 8 | 8 |
| TCGA-AA-3524 | 9 | 8 | 9 | 8 |
| TCGA-AA-3877 | 9 | 8 | 9 | 8 |
| TCGA-AA-3986 | 9 | 8 | 9 | 8 |
| TCGA-AA-A00D | 9 | 9 | 9 | 9 |
| TCGA-AA-A00U | 9 | 7 | 8 | 7 |
| TCGA-AA-A01P | 9 | 9 | 9 | 9 |
| TCGA-AD-6888 | 9 | 7 | 8 | 7 |
| TCGA-DM-A288 | 9 | 7 | 8 | 6 |
| TCGA-G4-6310 | 9 | 7 | 8 | 6 |
| TCGA-AA-3976 | 9 | 7 | 8 | 7 |
| TCGA-AZ-6605 | 9 | 8 | 8 | 7 |
| TCGA-A6-A567 | 9 | 7 | 8 | 7 |
| TCGA-AA-3495 | 9 | 7 | 8 | 7 |
| TCGA-AA-3973 | 9 | 8 | 9 | 7 |
| TCGA-G4-6588 | 9 | 7 | 8 | 7 |
| TCGA-F4-6807 | 9 | 8 | 9 | 8 |
| TCGA-AA-3972 | 9 | 7 | 8 | 7 |
| TCGA-AA-3511 | 9 | 7 | 9 | 7 |
| TCGA-CA-5256 | 9 | 8 | 9 | 7 |
| TCGA-AA-3526 | 9 | 8 | 9 | 8 |
| TCGA-AD-6889 | 9 | 8 | 9 | 7 |
| TCGA-A6-5662 | 9 | 8 | 8 | 7 |
| TCGA-AA-A01V | 9 | 9 | 10 | 9 |
| TCGA-AA-A00K | 9 | 7 | 9 | 7 |
| TCGA-CK-6746 | 9 | 9 | 9 | 9 |
| TCGA-AA-A01K | 9 | 8 | 9 | 8 |
| TCGA-G4-6627 | 9 | 8 | 9 | 7 |
| TCGA-AA-3819 | 9 | 7 | 8 | 6 |
| TCGA-A6-6652 | 9 | 7 | 9 | 7 |
| TCGA-CM-5860 | 9 | 8 | 9 | 8 |
| TCGA-CM-6677 | 9 | 7 | 8 | 7 |
| TCGA-A6-5659 | 9 | 7 | 8 | 6 |
| TCGA-AA-3975 | 9 | 7 | 9 | 7 |
| TCGA-AA-3532 | 9 | 9 | 9 | 9 |
| TCGA-DM-A28A | 9 | 7 | 8 | 7 |
| TCGA-AA-3681 | 9 | 8 | 8 | 7 |
| TCGA-AA-3860 | 9 | 8 | 9 | 8 |
| TCGA-AY-A69D | 9 | 7 | 8 | 7 |
| TCGA-AZ-4323 | 9 | 8 | 9 | 8 |
| TCGA-F4-6704 | 9 | 8 | 8 | 7 |
| TCGA-AY-A8YK | 9 | 7 | 9 | 7 |
| TCGA-AZ-6608 | 9 | 7 | 9 | 7 |
| TCGA-AD-6548 | 9 | 8 | 8 | 7 |
| TCGA-AA-3952 | 9 | 7 | 8 | 6 |
| TCGA-AA-3811 | 9 | 8 | 9 | 8 |
| TCGA-AY-A54L | 9 | 7 | 8 | 6 |
| TCGA-A6-2677 | 9 | 7 | 8 | 6 |
| TCGA-AA-3814 | 9 | 7 | 8 | 7 |
| TCGA-AA-3680 | 9 | 7 | 9 | 7 |
| TCGA-CK-4950 | 9 | 8 | 9 | 8 |
| TCGA-AA-3662 | 9 | 8 | 8 | 7 |
| TCGA-AA-3710 | 9 | 9 | 8 | 8 |
| TCGA-AA-3517 | 9 | 7 | 9 | 7 |
| TCGA-A6-5657 | 9 | 8 | 9 | 8 |
| TCGA-D5-6920 | 9 | 8 | 9 | 7 |
| TCGA-AA-3685 | 9 | 8 | 9 | 8 |
| TCGA-CM-6164 | 9 | 8 | 9 | 7 |
| TCGA-AY-4070 | 9 | 7 | 9 | 7 |
| TCGA-A6-A565 | 9 | 8 | 9 | 8 |
| TCGA-AA-3697 | 9 | 8 | 9 | 8 |
| TCGA-AA-3514 | 9 | 7 | 9 | 7 |
| TCGA-DM-A28C | 9 | 7 | 8 | 6 |
| TCGA-A6-6650 | 9 | 7 | 9 | 7 |
| TCGA-CA-6718 | 9 | 9 | 9 | 9 |
| TCGA-AA-3660 | 9 | 7 | 9 | 7 |
| TCGA-D5-6533 | 9 | 7 | 8 | 7 |
| TCGA-AA-A02O | 9 | 7 | 9 | 7 |
| TCGA-AA-3852 | 9 | 8 | 9 | 7 |
| TCGA-DM-A1D7 | 9 | 7 | 9 | 7 |
| TCGA-AU-3779 | 9 | 8 | 9 | 8 |
| TCGA-G4-6302 | 9 | 7 | 8 | 7 |
| TCGA-G4-6295 | 9 | 8 | 9 | 8 |
| TCGA-CM-6680 | 9 | 8 | 8 | 7 |
| TCGA-AA-3556 | 9 | 8 | 9 | 8 |
| TCGA-AA-3818 | 9 | 7 | 8 | 7 |
| TCGA-AA-3970 | 9 | 8 | 9 | 8 |
| TCGA-AA-A02W | 9 | 7 | 8 | 6 |
| TCGA-AA-A02J | 9 | 7 | 8 | 6 |
| TCGA-CK-4947 | 9 | 8 | 9 | 8 |
| TCGA-AA-3994 | 9 | 8 | 9 | 8 |
| TCGA-AA-A02R | 9 | 9 | 9 | 8 |
| TCGA-CM-6679 | 9 | 7 | 8 | 7 |
| TCGA-CA-6715 | 9 | 7 | 8 | 6 |
| TCGA-AA-3854 | 9 | 7 | 8 | 7 |
| TCGA-AA-3956 | 9 | 8 | 9 | 7 |
| TCGA-A6-2681 | 9 | 7 | 8 | 7 |
| TCGA-AA-3841 | 9 | 8 | 9 | 8 |
| TCGA-A6-2676 | 9 | 9 | 9 | 9 |
| TCGA-A6-3808 | 9 | 8 | 9 | 8 |
| TCGA-AA-3842 | 9 | 8 | 9 | 7 |
| TCGA-CM-6163 | 9 | 7 | 8 | 7 |
| TCGA-AA-A01C | 9 | 8 | 9 | 7 |
| TCGA-AA-3939 | 9 | 7 | 9 | 7 |
| TCGA-AA-3930 | 9 | 8 | 9 | 7 |
| TCGA-NH-A6GA | 9 | 7 | 9 | 7 |
| TCGA-AA-3941 | 9 | 7 | 9 | 7 |
| TCGA-AA-3875 | 9 | 7 | 8 | 7 |
| TCGA-CM-6674 | 9 | 8 | 9 | 7 |
| TCGA-G4-6304 | 9 | 8 | 9 | 7 |
| TCGA-AZ-6603 | 9 | 7 | 8 | 7 |
| TCGA-A6-6141 | 9 | 8 | 8 | 7 |
| TCGA-AA-3982 | 9 | 8 | 9 | 7 |
| TCGA-CM-5864 | 9 | 8 | 9 | 7 |
| TCGA-AA-3955 | 9 | 7 | 8 | 7 |
| TCGA-D5-6931 | 9 | 8 | 8 | 7 |
| TCGA-G4-6625 | 9 | 9 | 9 | 9 |
| TCGA-AA-3520 | 9 | 8 | 9 | 7 |
| TCGA-AA-A00F | 9 | 7 | 8 | 7 |
| TCGA-G4-6297 | 9 | 8 | 9 | 8 |
| TCGA-AA-3673 | 9 | 7 | 8 | 7 |
| TCGA-AA-A00Q | 9 | 7 | 8 | 7 |
| TCGA-A6-6648 | 9 | 8 | 9 | 7 |
| TCGA-DM-A280 | 9 | 7 | 8 | 7 |
| TCGA-AA-A02H | 9 | 7 | 9 | 7 |
| TCGA-F4-6809 | 9 | 8 | 9 | 7 |
| TCGA-AA-3544 | 9 | 8 | 9 | 8 |
| TCGA-AD-6890 | 9 | 8 | 9 | 8 |
| TCGA-AD-A5EK | 9 | 7 | 8 | 6 |
| TCGA-CM-5344 | 9 | 7 | 8 | 6 |
| TCGA-G4-6317 | 9 | 7 | 8 | 7 |
| TCGA-4T-AA8H | 9 | 7 | 9 | 7 |
| TCGA-AD-6899 | 9 | 8 | 8 | 7 |
| TCGA-AZ-5407 | 9 | 7 | 9 | 7 |
| TCGA-A6-3810 | 9 | 8 | 9 | 7 |
| TCGA-DM-A0XF | 9 | 7 | 8 | 7 |
| TCGA-AA-3553 | 9 | 8 | 9 | 8 |
| TCGA-NH-A50U | 9 | 7 | 8 | 7 |
| TCGA-F4-6569 | 9 | 7 | 8 | 7 |
| TCGA-QG-A5YV | 9 | 7 | 8 | 7 |
| TCGA-AD-6965 | 9 | 8 | 9 | 7 |
| TCGA-CM-6167 | 9 | 7 | 8 | 7 |
| TCGA-QG-A5YW | 9 | 8 | 9 | 7 |
| TCGA-AA-3845 | 9 | 9 | 8 | 9 |
| TCGA-AA-3715 | 9 | 9 | 9 | 9 |
| TCGA-AZ-6600 | 9 | 8 | 9 | 7 |
| TCGA-AA-3979 | 9 | 8 | 9 | 7 |
| TCGA-AA-A00Z | 9 | 8 | 9 | 7 |
| TCGA-AY-A71X | 9 | 7 | 9 | 7 |
| TCGA-AA-A024 | 9 | 8 | 9 | 8 |
| TCGA-AA-3989 | 9 | 8 | 8 | 7 |
| TCGA-AA-3667 | 9 | 8 | 9 | 7 |
| TCGA-D5-6539 | 9 | 8 | 9 | 7 |
| TCGA-G4-6315 | 9 | 7 | 8 | 6 |
| TCGA-AA-3968 | 9 | 8 | 8 | 7 |
| TCGA-AZ-4684 | 9 | 7 | 8 | 7 |
| TCGA-AA-3870 | 9 | 8 | 8 | 8 |
| TCGA-DM-A1D9 | 9 | 7 | 8 | 6 |
| TCGA-AZ-4615 | 9 | 9 | 9 | 8 |
| TCGA-CM-6678 | 9 | 7 | 8 | 6 |
| TCGA-DM-A1DB | 9 | 8 | 9 | 7 |
| TCGA-G4-6309 | 9 | 8 | 9 | 7 |
| TCGA-D5-6531 | 9 | 8 | 8 | 8 |
| TCGA-D5-6540 | 9 | 8 | 9 | 7 |
| TCGA-CM-5863 | 9 | 7 | 8 | 6 |
| TCGA-AA-3549 | 9 | 8 | 8 | 7 |
| TCGA-AA-3666 | 9 | 8 | 9 | 8 |
| TCGA-5M-AATE | 9 | 7 | 9 | 7 |
| TCGA-DM-A282 | 9 | 7 | 8 | 6 |
| TCGA-5M-AATA | 9 | 7 | 8 | 7 |
| TCGA-DC-6154 | 9 | 7 | 8 | 6 |
| TCGA-CI-6624 | 9 | 7 | 8 | 7 |
| TCGA-EI-6881 | 9 | 8 | 9 | 8 |
| TCGA-AG-A011 | 9 | 7 | 8 | 7 |
| TCGA-DC-6681 | 9 | 8 | 9 | 7 |
| TCGA-EI-6514 | 9 | 8 | 9 | 7 |
| TCGA-AG-3593 | 9 | 7 | 8 | 7 |
| TCGA-AH-6903 | 9 | 8 | 9 | 7 |
| TCGA-AG-A01N | 9 | 6 | 8 | 6 |
| TCGA-DY-A1DC | 9 | 7 | 9 | 7 |
| TCGA-AG-3592 | 9 | 8 | 9 | 7 |
| TCGA-AG-3726 | 9 | 7 | 8 | 7 |
| TCGA-EI-6512 | 9 | 7 | 8 | 7 |
| TCGA-DC-6160 | 9 | 8 | 9 | 7 |
| TCGA-AF-2693 | 9 | 8 | 9 | 8 |
| TCGA-AG-3581 | 9 | 7 | 8 | 7 |
| TCGA-AG-A026 | 9 | 7 | 8 | 6 |
| TCGA-AG-4015 | 9 | 8 | 9 | 7 |
| TCGA-AG-4022 | 9 | 7 | 9 | 7 |
| TCGA-AG-A015 | 9 | 7 | 8 | 7 |
| TCGA-G5-6641 | 9 | 7 | 9 | 7 |
| TCGA-F5-6571 | 9 | 8 | 8 | 8 |
| TCGA-AG-3612 | 9 | 7 | 8 | 7 |
| TCGA-DC-6683 | 9 | 7 | 8 | 6 |
| TCGA-AG-4005 | 9 | 7 | 8 | 7 |
| TCGA-AF-3911 | 9 | 8 | 9 | 7 |
| TCGA-EF-5830 | 9 | 7 | 8 | 7 |
| TCGA-AG-3605 | 9 | 7 | 8 | 7 |
| TCGA-AG-3732 | 9 | 8 | 9 | 8 |
| TCGA-EI-6513 | 9 | 7 | 8 | 7 |
| TCGA-AG-3586 | 9 | 8 | 9 | 8 |
| TCGA-F5-6813 | 9 | 8 | 9 | 8 |
| TCGA-AG-3896 | 9 | 8 | 9 | 7 |
| TCGA-CI-6623 | 9 | 7 | 8 | 7 |
| TCGA-AH-6547 | 9 | 8 | 8 | 7 |
| TCGA-AG-A016 | 9 | 7 | 9 | 7 |
| TCGA-AG-3582 | 9 | 8 | 8 | 7 |
| TCGA-CL-5918 | 9 | 7 | 9 | 7 |
| TCGA-AG-3892 | 9 | 9 | 9 | 9 |
| TCGA-AG-3725 | 9 | 8 | 9 | 8 |
| TCGA-AG-3878 | 9 | 8 | 9 | 8 |
| TCGA-AG-4008 | 9 | 7 | 8 | 7 |
| TCGA-AG-A01W | 9 | 8 | 9 | 7 |
| TCGA-F5-6861 | 9 | 7 | 8 | 6 |
| TCGA-EF-5831 | 9 | 7 | 8 | 7 |
| TCGA-AG-A02N | 9 | 8 | 9 | 7 |
| TCGA-DY-A1DD | 9 | 7 | 8 | 6 |
| TCGA-AG-3609 | 9 | 8 | 9 | 8 |
| TCGA-AG-A014 | 9 | 7 | 8 | 7 |
| TCGA-DY-A1DE | 9 | 8 | 9 | 8 |
| TCGA-AG-3580 | 9 | 8 | 9 | 7 |
| TCGA-AG-3898 | 9 | 8 | 9 | 7 |
| TCGA-AG-3583 | 9 | 8 | 9 | 7 |
| TCGA-AF-6136 | 9 | 8 | 9 | 7 |
| TCGA-AG-3575 | 9 | 9 | 9 | 8 |
| TCGA-AF-4110 | 9 | 8 | 9 | 8 |
| TCGA-AF-6672 | 9 | 8 | 9 | 7 |
| TCGA-DC-6157 | 9 | 7 | 8 | 7 |
| TCGA-DC-4745 | 9 | 7 | 9 | 7 |
| TCGA-AG-3881 | 9 | 8 | 9 | 8 |
| TCGA-AG-3883 | 9 | 8 | 9 | 8 |
| TCGA-AG-A025 | 9 | 8 | 9 | 7 |
| TCGA-AG-3893 | 9 | 8 | 9 | 7 |
| TCGA-AG-A00Y | 9 | 8 | 9 | 7 |
| TCGA-AG-A00H | 9 | 7 | 8 | 6 |
| TCGA-EI-6882 | 9 | 8 | 8 | 7 |
| TCGA-EI-6506 | 9 | 8 | 9 | 8 |
| TCGA-AG-3902 | 9 | 9 | 9 | 9 |
| TCGA-AF-A56L | 9 | 7 | 8 | 7 |
| TCGA-AG-3587 | 9 | 8 | 9 | 7 |
| TCGA-AG-3594 | 9 | 9 | 9 | 8 |
| TCGA-DY-A1DF | 9 | 7 | 8 | 6 |
| TCGA-F5-6465 | 9 | 8 | 8 | 8 |
| TCGA-CI-6620 | 9 | 7 | 8 | 7 |
| TCGA-EI-6884 | 9 | 8 | 9 | 7 |
| TCGA-F5-6811 | 9 | 8 | 9 | 7 |
| TCGA-AG-3727 | 9 | 8 | 8 | 7 |
| TCGA-DC-5869 | 9 | 7 | 8 | 7 |
| TCGA-BM-6198 | 9 | 8 | 9 | 8 |
| TCGA-AG-A008 | 9 | 8 | 9 | 7 |
| TCGA-G5-6233 | 9 | 8 | 9 | 7 |
| TCGA-AA-3977 | 8 | 7 | 8 | 7 |
| TCGA-CM-6161 | 8 | 7 | 8 | 6 |
| TCGA-CK-5916 | 8 | 7 | 7 | 7 |
| TCGA-D5-6541 | 8 | 7 | 8 | 7 |
| TCGA-A6-2678 | 8 | 7 | 8 | 6 |
| TCGA-CM-6676 | 8 | 6 | 7 | 5 |
| TCGA-A6-2684 | 8 | 7 | 8 | 7 |
| TCGA-AA-A01Q | 8 | 7 | 8 | 7 |
| TCGA-A6-5665 | 8 | 7 | 8 | 6 |
| TCGA-AA-3833 | 8 | 7 | 8 | 7 |
| TCGA-D5-5538 | 8 | 7 | 8 | 7 |
| TCGA-A6-5660 | 8 | 6 | 8 | 6 |
| TCGA-A6-6651 | 8 | 7 | 7 | 6 |
| TCGA-DM-A1D0 | 8 | 6 | 7 | 5 |
| TCGA-F4-6855 | 8 | 6 | 7 | 6 |
| TCGA-AD-5900 | 8 | 7 | 8 | 7 |
| TCGA-5M-AAT4 | 8 | 7 | 8 | 6 |
| TCGA-D5-6932 | 8 | 7 | 8 | 7 |
| TCGA-CM-5349 | 8 | 6 | 7 | 6 |
| TCGA-F4-6854 | 8 | 7 | 8 | 6 |
| TCGA-G4-6303 | 8 | 6 | 7 | 6 |
| TCGA-AM-5820 | 8 | 6 | 7 | 6 |
| TCGA-A6-5667 | 8 | 6 | 8 | 6 |
| TCGA-AY-6196 | 8 | 8 | 8 | 8 |
| TCGA-CK-4951 | 8 | 7 | 8 | 7 |
| TCGA-AA-A01X | 8 | 6 | 7 | 5 |
| TCGA-AA-A00O | 8 | 7 | 8 | 6 |
| TCGA-D5-5540 | 8 | 6 | 7 | 6 |
| TCGA-A6-2675 | 8 | 7 | 8 | 7 |
| TCGA-AA-3713 | 8 | 7 | 8 | 7 |
| TCGA-CM-6168 | 8 | 7 | 8 | 7 |
| TCGA-CM-6166 | 8 | 6 | 7 | 5 |
| TCGA-AZ-5403 | 8 | 6 | 7 | 6 |
| TCGA-AA-3492 | 8 | 7 | 8 | 7 |
| TCGA-A6-3809 | 8 | 8 | 8 | 8 |
| TCGA-AA-3543 | 8 | 8 | 8 | 7 |
| TCGA-D5-5539 | 8 | 7 | 8 | 6 |
| TCGA-D5-6922 | 8 | 7 | 8 | 7 |
| TCGA-D5-5541 | 8 | 7 | 7 | 6 |
| TCGA-A6-6780 | 8 | 8 | 8 | 8 |
| TCGA-CA-6716 | 8 | 6 | 7 | 5 |
| TCGA-A6-4105 | 8 | 7 | 8 | 7 |
| TCGA-AZ-6598 | 8 | 7 | 7 | 6 |
| TCGA-3L-AA1B | 8 | 7 | 8 | 6 |
| TCGA-DM-A285 | 8 | 6 | 8 | 5 |
| TCGA-AA-3696 | 8 | 6 | 7 | 5 |
| TCGA-G4-6314 | 8 | 6 | 8 | 6 |
| TCGA-AA-3538 | 8 | 6 | 7 | 5 |
| TCGA-AA-3678 | 8 | 7 | 8 | 7 |
| TCGA-CK-5915 | 8 | 6 | 7 | 5 |
| TCGA-D5-6529 | 8 | 8 | 8 | 7 |
| TCGA-QG-A5Z1 | 8 | 6 | 7 | 5 |
| TCGA-CA-6719 | 8 | 7 | 8 | 7 |
| TCGA-CM-4746 | 8 | 7 | 8 | 6 |
| TCGA-AA-3984 | 8 | 7 | 8 | 7 |
| TCGA-CM-6165 | 8 | 7 | 8 | 6 |
| TCGA-AA-A02F | 8 | 6 | 7 | 5 |
| TCGA-AZ-4315 | 8 | 7 | 7 | 6 |
| TCGA-AA-3534 | 8 | 6 | 7 | 5 |
| TCGA-A6-5664 | 8 | 7 | 8 | 7 |
| TCGA-D5-6530 | 8 | 7 | 7 | 7 |
| TCGA-AA-3488 | 8 | 7 | 8 | 6 |
| TCGA-A6-A56B | 8 | 6 | 7 | 5 |
| TCGA-G4-6586 | 8 | 7 | 8 | 7 |
| TCGA-A6-2682 | 8 | 6 | 7 | 6 |
| TCGA-G4-6311 | 8 | 7 | 8 | 6 |
| TCGA-AA-3509 | 8 | 7 | 8 | 6 |
| TCGA-CM-6169 | 8 | 7 | 8 | 7 |
| TCGA-A6-2671 | 8 | 7 | 8 | 7 |
| TCGA-G4-6307 | 8 | 6 | 8 | 6 |
| TCGA-CM-4747 | 8 | 7 | 8 | 6 |
| TCGA-A6-2674 | 8 | 8 | 8 | 7 |
| TCGA-CK-4948 | 8 | 6 | 8 | 6 |
| TCGA-AD-6964 | 8 | 8 | 8 | 8 |
| TCGA-AM-5821 | 8 | 9 | 8 | 8 |
| TCGA-AA-3496 | 8 | 8 | 8 | 7 |
| TCGA-NH-A8F7 | 8 | 6 | 8 | 6 |
| TCGA-AA-3494 | 8 | 7 | 8 | 6 |
| TCGA-D5-6536 | 8 | 6 | 8 | 6 |
| TCGA-A6-6781 | 8 | 7 | 7 | 7 |
| TCGA-A6-2672 | 8 | 8 | 8 | 8 |
| TCGA-AD-6901 | 8 | 7 | 8 | 7 |
| TCGA-CM-5348 | 8 | 6 | 7 | 6 |
| TCGA-CM-6170 | 8 | 7 | 8 | 7 |
| TCGA-AA-3542 | 8 | 6 | 8 | 6 |
| TCGA-AA-3521 | 8 | 7 | 8 | 6 |
| TCGA-D5-6534 | 8 | 8 | 8 | 8 |
| TCGA-D5-6537 | 8 | 7 | 8 | 6 |
| TCGA-CM-6162 | 8 | 8 | 8 | 7 |
| TCGA-AA-3489 | 8 | 8 | 8 | 7 |
| TCGA-A6-6142 | 8 | 6 | 7 | 6 |
| TCGA-AA-3554 | 8 | 8 | 8 | 7 |
| TCGA-NH-A50V | 8 | 7 | 8 | 7 |
| TCGA-AA-3864 | 8 | 7 | 8 | 6 |
| TCGA-F4-6570 | 8 | 7 | 7 | 7 |
| TCGA-D5-6928 | 8 | 9 | 8 | 9 |
| TCGA-CM-6171 | 8 | 6 | 7 | 6 |
| TCGA-AA-A017 | 8 | 6 | 8 | 6 |
| TCGA-AA-3692 | 8 | 7 | 8 | 6 |
| TCGA-AA-3831 | 8 | 6 | 7 | 6 |
| TCGA-D5-7000 | 8 | 7 | 8 | 7 |
| TCGA-AA-A00R | 8 | 8 | 8 | 8 |
| TCGA-F4-6459 | 8 | 6 | 7 | 6 |
| TCGA-AA-3664 | 8 | 7 | 8 | 7 |
| TCGA-D5-6924 | 8 | 7 | 8 | 7 |
| TCGA-D5-6538 | 8 | 6 | 7 | 5 |
| TCGA-AA-A00N | 8 | 7 | 8 | 7 |
| TCGA-AA-3529 | 8 | 6 | 8 | 6 |
| TCGA-AA-3867 | 8 | 7 | 8 | 6 |
| TCGA-AA-3949 | 8 | 9 | 8 | 8 |
| TCGA-F4-6463 | 8 | 6 | 8 | 6 |
| TCGA-CM-5862 | 8 | 7 | 8 | 6 |
| TCGA-A6-2685 | 8 | 7 | 8 | 7 |
| TCGA-D5-6923 | 8 | 6 | 7 | 6 |
| TCGA-5M-AAT6 | 8 | 8 | 8 | 8 |
| TCGA-F4-6805 | 8 | 7 | 8 | 7 |
| TCGA-DM-A28G | 8 | 7 | 8 | 6 |
| TCGA-F5-6864 | 8 | 7 | 8 | 6 |
| TCGA-AF-2690 | 8 | 7 | 8 | 7 |
| TCGA-CI-6619 | 8 | 7 | 8 | 7 |
| TCGA-DT-5265 | 8 | 6 | 8 | 6 |
| TCGA-AH-6549 | 8 | 7 | 8 | 6 |
| TCGA-DC-6156 | 8 | 7 | 7 | 6 |
| TCGA-F5-6810 | 8 | 6 | 7 | 6 |
| TCGA-EI-7002 | 8 | 7 | 8 | 6 |
| TCGA-G5-6572 | 8 | 6 | 7 | 6 |
| TCGA-DC-6158 | 8 | 7 | 8 | 7 |
| TCGA-AF-3913 | 8 | 6 | 7 | 6 |
| TCGA-AG-3578 | 8 | 6 | 7 | 6 |
| TCGA-AG-3894 | 8 | 7 | 8 | 6 |
| TCGA-CI-6621 | 8 | 7 | 8 | 7 |
| TCGA-AG-3600 | 8 | 7 | 8 | 7 |
| TCGA-AH-6897 | 8 | 7 | 8 | 6 |
| TCGA-EI-6917 | 8 | 7 | 8 | 7 |
| TCGA-AG-A01Y | 8 | 7 | 8 | 7 |
| TCGA-F5-6702 | 8 | 7 | 7 | 6 |
| TCGA-AG-4001 | 8 | 7 | 8 | 6 |
| TCGA-EI-6508 | 8 | 6 | 8 | 6 |
| TCGA-AH-6644 | 8 | 6 | 8 | 6 |
| TCGA-AF-2687 | 8 | 7 | 8 | 7 |
| TCGA-AF-2692 | 8 | 7 | 8 | 6 |
| TCGA-AG-3731 | 8 | 7 | 8 | 7 |
| TCGA-AG-3742 | 8 | 6 | 8 | 6 |
| TCGA-F5-6863 | 8 | 6 | 7 | 5 |
| TCGA-CL-5917 | 8 | 6 | 7 | 5 |
| TCGA-F5-6464 | 8 | 7 | 7 | 7 |
| TCGA-AF-A56N | 8 | 7 | 8 | 6 |
| TCGA-AF-5654 | 8 | 6 | 7 | 6 |
| TCGA-AG-3999 | 8 | 6 | 7 | 6 |
| TCGA-EI-6509 | 8 | 6 | 8 | 6 |
| TCGA-EI-6511 | 8 | 8 | 8 | 7 |
| TCGA-DC-6155 | 8 | 7 | 8 | 6 |
| TCGA-AG-3601 | 8 | 6 | 8 | 6 |
| TCGA-DY-A0XA | 8 | 7 | 8 | 6 |
| TCGA-AH-6643 | 8 | 7 | 9 | 7 |
| TCGA-AF-A56K | 8 | 6 | 8 | 6 |
| TCGA-AG-3584 | 8 | 7 | 8 | 6 |
| TCGA-EI-7004 | 8 | 7 | 8 | 7 |
| TCGA-AF-6655 | 8 | 7 | 8 | 7 |
| TCGA-AA-3555 | 7 | 6 | 7 | 5 |
| TCGA-D5-6926 | 7 | 7 | 7 | 6 |
| TCGA-A6-2686 | 7 | 7 | 7 | 7 |
| TCGA-CA-6717 | 7 | 6 | 7 | 6 |
| TCGA-D5-6898 | 7 | 6 | 7 | 5 |
| TCGA-AA-A010 | 7 | 6 | 8 | 6 |
| TCGA-F4-6703 | 7 | 7 | 7 | 7 |
| TCGA-G4-6298 | 7 | 5 | 6 | 5 |
| TCGA-AA-3663 | 7 | 6 | 7 | 6 |
| TCGA-A6-A566 | 7 | 6 | 7 | 6 |
| TCGA-AZ-6601 | 7 | 7 | 7 | 7 |
| TCGA-CK-6748 | 7 | 6 | 7 | 5 |
| TCGA-DM-A1HB | 7 | 6 | 7 | 5 |
| TCGA-A6-6654 | 7 | 7 | 7 | 7 |
| TCGA-CK-4952 | 7 | 6 | 7 | 5 |
| TCGA-AA-A00J | 7 | 6 | 7 | 5 |
| TCGA-AZ-6607 | 7 | 7 | 7 | 7 |
| TCGA-AA-A01D | 7 | 5 | 7 | 5 |
| TCGA-AA-3966 | 7 | 6 | 7 | 6 |
| TCGA-DM-A28H | 7 | 6 | 7 | 5 |
| TCGA-AA-3815 | 7 | 7 | 7 | 7 |
| TCGA-NH-A5IV | 7 | 6 | 6 | 5 |
| TCGA-AA-3950 | 7 | 7 | 7 | 7 |
| TCGA-AG-3574 | 7 | 6 | 7 | 5 |
| TCGA-EI-6885 | 7 | 6 | 6 | 6 |
| TCGA-F5-6814 | 7 | 7 | 7 | 7 |
| TCGA-AG-3901 | 7 | 6 | 7 | 6 |
| TCGA-DY-A1H8 | 7 | 6 | 7 | 5 |
| TCGA-AG-4021 | 7 | 5 | 6 | 5 |
| TCGA-AG-A002 | 7 | 5 | 7 | 5 |
| TCGA-AF-3400 | 7 | 7 | 7 | 7 |
| TCGA-D5-6927 | 6 | 6 | 6 | 6 |
| TCGA-WS-AB45 | 6 | 6 | 6 | 6 |
| TCGA-SS-A7HO | 6 | 5 | 6 | 5 |
| TCGA-AD-A5EJ | 6 | 5 | 6 | 5 |
| TCGA-AA-3947 | 6 | 5 | 6 | 5 |
| TCGA-EI-6507 | 6 | 6 | 6 | 6 |

**Supplementary Table S10.** The cluster group of TCGA samples.

| ID | Cluster |
| --- | --- |
| TCGA-AA-3818 | C1 |
| TCGA-AA-3543 | C2 |
| TCGA-AA-A00R | C2 |
| TCGA-AA-A01S | C2 |
| TCGA-AA-3496 | C2 |
| TCGA-AA-3494 | C1 |
| TCGA-AG-A020 | C1 |
| TCGA-AG-3883 | C2 |
| TCGA-AG-3896 | C1 |
| TCGA-AZ-4323 | C1 |
| TCGA-F4-6704 | C2 |
| TCGA-AG-A026 | C1 |
| TCGA-AA-3966 | C2 |
| TCGA-AA-3930 | C1 |
| TCGA-AG-3574 | C1 |
| TCGA-EF-5830 | C1 |
| TCGA-AG-4021 | C1 |
| TCGA-4N-A93T | C1 |
| TCGA-D5-6537 | C1 |
| TCGA-AA-A02J | C2 |
| TCGA-AZ-4616 | C1 |
| TCGA-AZ-6605 | C2 |
| TCGA-AZ-4614 | C1 |
| TCGA-AD-6899 | C1 |
| TCGA-AG-A00C | C1 |
| TCGA-CI-6619 | C1 |
| TCGA-F4-6570 | C2 |
| TCGA-AA-3511 | C2 |
| TCGA-AA-3663 | C2 |
| TCGA-D5-6898 | C2 |
| TCGA-DM-A280 | C2 |
| TCGA-AG-3726 | C2 |
| TCGA-F4-6459 | C1 |
| TCGA-EI-6882 | C2 |
| TCGA-AA-3519 | C2 |
| TCGA-D5-6927 | C1 |
| TCGA-NH-A6GA | C1 |
| TCGA-CM-6679 | C1 |
| TCGA-AA-3811 | C1 |
| TCGA-CA-6718 | C2 |
| TCGA-AD-6964 | C2 |
| TCGA-NH-A50U | C1 |
| TCGA-DC-6158 | C1 |
| TCGA-CM-6172 | C2 |
| TCGA-CM-6678 | C2 |
| TCGA-AA-3680 | C1 |
| TCGA-CM-6677 | C1 |
| TCGA-F4-6461 | C2 |
| TCGA-D5-6932 | C1 |
| TCGA-EI-6883 | C2 |
| TCGA-D5-6928 | C2 |
| TCGA-AZ-6606 | C2 |
| TCGA-EI-7002 | C1 |
| TCGA-CM-6162 | C2 |
| TCGA-D5-6931 | C1 |
| TCGA-CM-6680 | C1 |
| TCGA-AD-5900 | C2 |
| TCGA-CA-6716 | C2 |
| TCGA-CA-5256 | C2 |
| TCGA-F5-6864 | C1 |
| TCGA-CA-5797 | C2 |
| TCGA-CA-6715 | C1 |
| TCGA-CM-6168 | C2 |
| TCGA-AA-3939 | C1 |
| TCGA-CM-4752 | C2 |
| TCGA-CM-6169 | C2 |
| TCGA-AG-3892 | C2 |
| TCGA-CM-6675 | C1 |
| TCGA-AA-3973 | C1 |
| TCGA-F4-6809 | C1 |
| TCGA-DM-A1D7 | C1 |
| TCGA-A6-2682 | C1 |
| TCGA-CL-4957 | C1 |
| TCGA-AA-A02K | C1 |
| TCGA-AA-3667 | C2 |
| TCGA-CM-6171 | C2 |
| TCGA-AG-4005 | C2 |
| TCGA-D5-6924 | C2 |
| TCGA-CA-6719 | C2 |
| TCGA-AU-3779 | C2 |
| TCGA-A6-4105 | C1 |
| TCGA-AA-3844 | C1 |
| TCGA-CM-6167 | C1 |
| TCGA-AA-A017 | C2 |
| TCGA-CM-5861 | C2 |
| TCGA-D5-6535 | C1 |
| TCGA-D5-6541 | C2 |
| TCGA-NH-A6GB | C1 |
| TCGA-EI-6511 | C2 |
| TCGA-AA-3833 | C2 |
| TCGA-AG-A014 | C2 |
| TCGA-CM-6165 | C2 |
| TCGA-D5-6540 | C2 |
| TCGA-A6-A565 | C1 |
| TCGA-EI-6514 | C2 |
| TCGA-EI-6513 | C1 |
| TCGA-AD-A5EK | C1 |
| TCGA-NH-A8F8 | C1 |
| TCGA-EI-6509 | C1 |
| TCGA-CK-6751 | C2 |
| TCGA-AG-3890 | C2 |
| TCGA-D5-6538 | C1 |
| TCGA-AY-A54L | C1 |
| TCGA-EI-6917 | C1 |
| TCGA-AH-6549 | C1 |
| TCGA-CK-4947 | C2 |
| TCGA-D5-6531 | C2 |
| TCGA-D5-6536 | C2 |
| TCGA-AA-3554 | C2 |
| TCGA-AA-3831 | C1 |
| TCGA-AA-A00J | C1 |
| TCGA-NH-A50T | C1 |
| TCGA-DM-A1D6 | C2 |
| TCGA-AY-A8YK | C1 |
| TCGA-AA-A00D | C2 |
| TCGA-AA-3688 | C1 |
| TCGA-AA-3715 | C2 |
| TCGA-AA-3713 | C2 |
| TCGA-AA-3526 | C1 |
| TCGA-AY-A71X | C1 |
| TCGA-A6-6781 | C1 |
| TCGA-EI-6507 | C1 |
| TCGA-AA-3562 | C2 |
| TCGA-AA-3560 | C2 |
| TCGA-CM-4744 | C2 |
| TCGA-A6-6780 | C1 |
| TCGA-D5-6529 | C2 |
| TCGA-D5-6530 | C2 |
| TCGA-EI-6506 | C1 |
| TCGA-A6-6650 | C1 |
| TCGA-AA-3549 | C2 |
| TCGA-AD-6548 | C1 |
| TCGA-AY-6197 | C1 |
| TCGA-AA-A00Z | C1 |
| TCGA-AA-3968 | C1 |
| TCGA-AA-A02R | C2 |
| TCGA-A6-5665 | C1 |
| TCGA-CM-5348 | C1 |
| TCGA-AA-3556 | C2 |
| TCGA-CM-4743 | C2 |
| TCGA-A6-6654 | C2 |
| TCGA-AA-3553 | C2 |
| TCGA-AA-3979 | C1 |
| TCGA-AG-3584 | C2 |
| TCGA-AA-3520 | C2 |
| TCGA-A6-6140 | C1 |
| TCGA-DM-A0XD | C1 |
| TCGA-A6-A566 | C1 |
| TCGA-AA-3977 | C2 |
| TCGA-AA-3819 | C1 |
| TCGA-D5-6533 | C2 |
| TCGA-CM-4748 | C2 |
| TCGA-G4-6588 | C2 |
| TCGA-G4-6320 | C1 |
| TCGA-CK-6747 | C2 |
| TCGA-AA-3982 | C2 |
| TCGA-G4-6294 | C1 |
| TCGA-AA-3534 | C1 |
| TCGA-AA-3532 | C1 |
| TCGA-CM-5341 | C2 |
| TCGA-A6-5660 | C2 |
| TCGA-QG-A5YW | C1 |
| TCGA-AF-4110 | C2 |
| TCGA-AG-3728 | C2 |
| TCGA-AA-3861 | C2 |
| TCGA-AA-A01I | C1 |
| TCGA-AA-3877 | C2 |
| TCGA-DC-5869 | C2 |
| TCGA-AA-3858 | C2 |
| TCGA-AA-3860 | C1 |
| TCGA-A6-5657 | C2 |
| TCGA-F4-6460 | C1 |
| TCGA-AA-A01F | C2 |
| TCGA-A6-5666 | C1 |
| TCGA-A6-3809 | C1 |
| TCGA-AZ-4615 | C2 |
| TCGA-CI-6620 | C2 |
| TCGA-AA-3975 | C1 |
| TCGA-F4-6805 | C2 |
| TCGA-A6-3807 | C1 |
| TCGA-AA-A010 | C1 |
| TCGA-AA-3502 | C2 |
| TCGA-AA-3812 | C2 |
| TCGA-A6-2680 | C2 |
| TCGA-F4-6856 | C2 |
| TCGA-AA-3854 | C2 |
| TCGA-AA-3970 | C2 |
| TCGA-AG-A015 | C1 |
| TCGA-A6-3810 | C1 |
| TCGA-AA-3841 | C2 |
| TCGA-AG-3887 | C1 |
| TCGA-A6-2686 | C1 |
| TCGA-AA-3842 | C2 |
| TCGA-AA-A01Z | C2 |
| TCGA-AA-3495 | C1 |
| TCGA-AA-3522 | C2 |
| TCGA-A6-2684 | C1 |
| TCGA-A6-2685 | C2 |
| TCGA-AF-3911 | C1 |
| TCGA-AA-A00L | C1 |
| TCGA-AA-A01P | C2 |
| TCGA-DM-A0XF | C1 |
| TCGA-RU-A8FL | C1 |
| TCGA-AG-A02G | C1 |
| TCGA-5M-AATE | C1 |
| TCGA-AA-A02F | C1 |
| TCGA-AA-A03J | C2 |
| TCGA-AG-A02X | C2 |
| TCGA-A6-2678 | C1 |
| TCGA-F5-6571 | C2 |
| TCGA-F4-6807 | C2 |
| TCGA-A6-2674 | C1 |
| TCGA-DM-A1DB | C1 |
| TCGA-G4-6306 | C1 |
| TCGA-A6-2681 | C2 |
| TCGA-AG-4022 | C1 |
| TCGA-A6-2672 | C1 |
| TCGA-AA-3678 | C1 |
| TCGA-AA-3675 | C2 |
| TCGA-AG-3898 | C1 |
| TCGA-AG-A025 | C2 |
| TCGA-AA-3673 | C1 |
| TCGA-AA-3972 | C2 |
| TCGA-CK-5913 | C2 |
| TCGA-DY-A1DG | C1 |
| TCGA-AG-A023 | C1 |
| TCGA-G4-6304 | C2 |
| TCGA-D5-5538 | C1 |
| TCGA-G4-6307 | C1 |
| TCGA-D5-5541 | C2 |
| TCGA-DM-A28G | C2 |
| TCGA-AA-3655 | C1 |
| TCGA-A6-A567 | C1 |
| TCGA-G4-6310 | C1 |
| TCGA-AA-3510 | C2 |
| TCGA-G4-6303 | C1 |
| TCGA-CK-4951 | C2 |
| TCGA-G4-6627 | C2 |
| TCGA-G4-6299 | C2 |
| TCGA-G4-6628 | C2 |
| TCGA-G4-6297 | C1 |
| TCGA-G4-6625 | C1 |
| TCGA-DM-A1D4 | C1 |
| TCGA-DM-A28M | C2 |
| TCGA-AG-A036 | C1 |
| TCGA-DM-A28E | C1 |
| TCGA-DY-A1DE | C1 |
| TCGA-G4-6293 | C2 |
| TCGA-DM-A1HB | C2 |
| TCGA-DM-A282 | C1 |
| TCGA-CK-4948 | C2 |
| TCGA-AA-3856 | C2 |
| TCGA-AG-3878 | C2 |
| TCGA-AA-3518 | C2 |
| TCGA-AA-3821 | C1 |
| TCGA-AA-A01V | C2 |
| TCGA-AA-A01Q | C2 |
| TCGA-AA-3514 | C2 |
| TCGA-AG-A01J | C2 |
| TCGA-5M-AAT4 | C1 |
| TCGA-AZ-6608 | C1 |
| TCGA-AA-3952 | C1 |
| TCGA-AA-A02H | C1 |
| TCGA-CK-6748 | C2 |
| TCGA-AA-3666 | C2 |
| TCGA-AA-A02E | C1 |
| TCGA-AZ-6607 | C2 |
| TCGA-AA-A00N | C2 |
| TCGA-EF-5831 | C1 |
| TCGA-CM-5862 | C2 |
| TCGA-AA-3696 | C1 |
| TCGA-DM-A285 | C1 |
| TCGA-AA-3681 | C2 |
| TCGA-AA-3662 | C1 |
| TCGA-AZ-6599 | C2 |
| TCGA-AA-3489 | C1 |
| TCGA-DM-A1DA | C1 |
| TCGA-AA-3980 | C1 |
| TCGA-AA-3989 | C2 |
| TCGA-G4-6295 | C1 |
| TCGA-A6-6141 | C2 |
| TCGA-D5-6926 | C1 |
| TCGA-AG-A016 | C2 |
| TCGA-5M-AAT6 | C1 |
| TCGA-A6-A5ZU | C2 |
| TCGA-AA-3848 | C1 |
| TCGA-D5-6922 | C1 |
| TCGA-D5-7000 | C1 |
| TCGA-EI-6884 | C2 |
| TCGA-CM-6676 | C1 |
| TCGA-F5-6863 | C1 |
| TCGA-T9-A92H | C1 |
| TCGA-AA-A01G | C1 |
| TCGA-AG-3575 | C2 |
| TCGA-AZ-6600 | C2 |
| TCGA-CA-5255 | C2 |
| TCGA-CA-5796 | C2 |
| TCGA-D5-6920 | C2 |
| TCGA-D5-6923 | C1 |
| TCGA-D5-6539 | C2 |
| TCGA-DM-A1D8 | C1 |
| TCGA-DT-5265 | C2 |
| TCGA-4T-AA8H | C1 |
| TCGA-CA-5254 | C2 |
| TCGA-CA-6717 | C2 |
| TCGA-NH-A6GC | C1 |
| TCGA-CM-6674 | C2 |
| TCGA-AA-3542 | C2 |
| TCGA-AA-3516 | C2 |
| TCGA-AA-3552 | C1 |
| TCGA-D5-6930 | C2 |
| TCGA-D5-6929 | C1 |
| TCGA-AF-2692 | C1 |
| TCGA-EI-6885 | C1 |
| TCGA-G4-6323 | C1 |
| TCGA-CI-6621 | C1 |
| TCGA-AA-A004 | C2 |
| TCGA-AA-3561 | C2 |
| TCGA-AG-A008 | C1 |
| TCGA-AA-3544 | C2 |
| TCGA-AG-3894 | C1 |
| TCGA-CM-6163 | C2 |
| TCGA-DM-A288 | C1 |
| TCGA-AA-A00W | C1 |
| TCGA-CM-6170 | C2 |
| TCGA-CM-5863 | C2 |
| TCGA-AA-3679 | C2 |
| TCGA-AA-A01C | C1 |
| TCGA-CM-6161 | C1 |
| TCGA-CM-5864 | C2 |
| TCGA-AD-6888 | C1 |
| TCGA-3L-AA1B | C1 |
| TCGA-CK-4952 | C1 |
| TCGA-AG-3608 | C2 |
| TCGA-AA-3971 | C1 |
| TCGA-AY-4070 | C1 |
| TCGA-EI-6881 | C2 |
| TCGA-A6-2683 | C2 |
| TCGA-CM-5868 | C2 |
| TCGA-AA-A00U | C1 |
| TCGA-AA-3846 | C1 |
| TCGA-AA-3866 | C1 |
| TCGA-AF-2690 | C2 |
| TCGA-EI-6512 | C2 |
| TCGA-AY-6386 | C2 |
| TCGA-NH-A8F7 | C1 |
| TCGA-AY-A69D | C1 |
| TCGA-AG-3885 | C2 |
| TCGA-AA-A00K | C1 |
| TCGA-AA-3875 | C1 |
| TCGA-D5-6532 | C1 |
| TCGA-G5-6233 | C1 |
| TCGA-EI-6510 | C1 |
| TCGA-AA-3530 | C1 |
| TCGA-AA-3986 | C2 |
| TCGA-NH-A50V | C1 |
| TCGA-D5-5539 | C2 |
| TCGA-AG-3612 | C2 |
| TCGA-A6-6782 | C2 |
| TCGA-EI-6508 | C1 |
| TCGA-AA-3955 | C1 |
| TCGA-AG-A002 | C2 |
| TCGA-CK-5916 | C2 |
| TCGA-BM-6198 | C1 |
| TCGA-A6-6651 | C2 |
| TCGA-QL-A97D | C1 |
| TCGA-CK-5914 | C1 |
| TCGA-CM-6166 | C1 |
| TCGA-CM-5344 | C1 |
| TCGA-G4-6321 | C2 |
| TCGA-A6-5664 | C1 |
| TCGA-AD-6901 | C1 |
| TCGA-A6-6138 | C2 |
| TCGA-AG-A00Y | C1 |
| TCGA-A6-5662 | C2 |
| TCGA-AA-3941 | C2 |
| TCGA-AA-3950 | C2 |
| TCGA-AA-3867 | C2 |
| TCGA-A6-6649 | C1 |
| TCGA-A6-2677 | C1 |
| TCGA-A6-6653 | C2 |
| TCGA-AD-6890 | C1 |
| TCGA-A6-6652 | C2 |
| TCGA-CM-4747 | C2 |
| TCGA-AD-6895 | C2 |
| TCGA-A6-6142 | C2 |
| TCGA-A6-6648 | C1 |
| TCGA-AG-A00H | C2 |
| TCGA-AA-3538 | C1 |
| TCGA-AA-A01X | C2 |
| TCGA-AA-3949 | C2 |
| TCGA-G4-6322 | C2 |
| TCGA-DM-A28A | C1 |
| TCGA-AD-6965 | C1 |
| TCGA-AA-3710 | C2 |
| TCGA-AA-A00O | C1 |
| TCGA-AA-3994 | C2 |
| TCGA-AA-3869 | C2 |
| TCGA-CM-4751 | C2 |
| TCGA-A6-6137 | C1 |
| TCGA-AU-6004 | C2 |
| TCGA-AD-6963 | C1 |
| TCGA-CM-6164 | C1 |
| TCGA-A6-5667 | C2 |
| TCGA-AZ-6603 | C1 |
| TCGA-AA-3555 | C2 |
| TCGA-AA-3870 | C2 |
| TCGA-AA-A00E | C2 |
| TCGA-AA-3862 | C2 |
| TCGA-CM-5349 | C2 |
| TCGA-A6-5659 | C1 |
| TCGA-AA-A01K | C1 |
| TCGA-AG-A01N | C1 |
| TCGA-QG-A5Z2 | C1 |
| TCGA-CM-5860 | C2 |
| TCGA-AA-3855 | C1 |
| TCGA-A6-4107 | C2 |
| TCGA-DY-A1H8 | C1 |
| TCGA-A6-5656 | C1 |
| TCGA-QG-A5YX | C2 |
| TCGA-AY-5543 | C1 |
| TCGA-AA-3947 | C1 |
| TCGA-AA-3815 | C2 |
| TCGA-AA-A01T | C1 |
| TCGA-AA-3851 | C1 |
| TCGA-A6-3808 | C1 |
| TCGA-A6-5661 | C2 |
| TCGA-F4-6808 | C1 |
| TCGA-AA-3548 | C2 |
| TCGA-AA-3956 | C2 |
| TCGA-AA-A00F | C1 |
| TCGA-AA-3531 | C1 |
| TCGA-AA-A01R | C2 |
| TCGA-F4-6569 | C2 |
| TCGA-F4-6463 | C1 |
| TCGA-G4-6586 | C2 |
| TCGA-G4-6314 | C1 |
| TCGA-DM-A28F | C1 |
| TCGA-AA-3692 | C1 |
| TCGA-G4-6317 | C1 |
| TCGA-AA-3524 | C1 |
| TCGA-CM-4746 | C2 |
| TCGA-AG-3731 | C1 |
| TCGA-AG-A011 | C2 |
| TCGA-AA-3685 | C2 |
| TCGA-F5-6814 | C1 |
| TCGA-AA-A00A | C2 |
| TCGA-AG-A032 | C2 |
| TCGA-F5-6861 | C2 |
| TCGA-AA-3837 | C2 |
| TCGA-AA-3517 | C1 |
| TCGA-AA-A024 | C2 |
| TCGA-G4-6311 | C1 |
| TCGA-AA-A02Y | C2 |
| TCGA-AA-A02W | C1 |
| TCGA-F4-6806 | C2 |
| TCGA-AA-A00Q | C1 |
| TCGA-QG-A5YV | C1 |
| TCGA-A6-2676 | C2 |
| TCGA-D5-6534 | C2 |
| TCGA-A6-2675 | C2 |
| TCGA-A6-2671 | C2 |
| TCGA-CI-6622 | C2 |
| TCGA-A6-2679 | C2 |
| TCGA-D5-5537 | C1 |
| TCGA-AG-3587 | C1 |
| TCGA-F4-6855 | C1 |
| TCGA-CI-6623 | C2 |
| TCGA-F4-6703 | C2 |
| TCGA-CI-6624 | C2 |
| TCGA-CK-5912 | C2 |
| TCGA-AZ-6598 | C2 |
| TCGA-AA-A029 | C2 |
| TCGA-AA-3864 | C2 |
| TCGA-AA-3664 | C1 |
| TCGA-D5-5540 | C1 |
| TCGA-A6-A56B | C1 |
| TCGA-AA-3506 | C2 |
| TCGA-AZ-4315 | C2 |
| TCGA-SS-A7HO | C1 |
| TCGA-G4-6315 | C1 |
| TCGA-AG-A02N | C1 |
| TCGA-AZ-5403 | C2 |
| TCGA-AA-3509 | C1 |
| TCGA-WS-AB45 | C2 |
| TCGA-AZ-4313 | C2 |
| TCGA-AA-3660 | C2 |
| TCGA-DM-A28C | C2 |
| TCGA-AD-6889 | C1 |
| TCGA-AA-3697 | C1 |
| TCGA-CK-4950 | C2 |
| TCGA-DM-A1HA | C1 |
| TCGA-G4-6309 | C2 |
| TCGA-AZ-5407 | C2 |
| TCGA-DM-A28K | C2 |
| TCGA-AZ-6601 | C2 |
| TCGA-AZ-4308 | C2 |
| TCGA-DM-A28H | C1 |
| TCGA-DM-A0X9 | C1 |
| TCGA-DM-A1D0 | C1 |
| TCGA-DM-A1D9 | C1 |
